# DNMT3A facilitates breast cancer progression via regulating ADAMTS8 mediated EGFR-MEK-ERK activation

**DOI:** 10.1371/journal.pone.0321889

**Published:** 2025-05-05

**Authors:** Shan Yang, Meng Cheng, Shaonan Zhang, Haoqi Wang, Jiaxing Wang, Xi Zhang, Yunzhe Mi, Sainan Li

**Affiliations:** 1 Department of Breast Center, The Fourth Hospital of Hebei Medical University, Shijiazhuang, Hebei, China; 2 Hebei Key Laboratory of Breast Cancer Molecular Medicine, Shijiazhuang, Hebei, China; National Institute of Cancer Research, TAIWAN

## Abstract

ADAMTS8 inactivation by epigenetic modifications has been reported in various tumors, and the dysregulation of ADAMTS8 expression is associated with poor clinical outcomes, cancer cell invasion, and metastasis. De novo methylation, involving DNMT3A, plays an important role in cancer development; however, it remains unclear whether DNMT3A regulates the progressive expression of breast cancer by regulating ADAMTS8. Through published cancer-related datasets and clinical validation, we found that ADAMTS8 and DNMT3A expression negatively correlated in breast cancer, and both associated with patient prognosis. Related cell experiments have shown that DNMT3A overexpression promotes breast cancer cell proliferation, migration, invasion, and apoptosis, whereas silencing DNMT3A has the opposite effect. Through Co-IP experiments, we confirmed that DNMT3A binds directly to ADAMTS8. Methylation-specific PCR (MSP) experiments confirmed that DNMT3A mediates ADAMTS8 promoter methylation in breast cancer. In addition, DNMT3A activated the EGFR-MEK-ERK signaling pathway by effectively downregulating ADAMTS8, whereas silencing ADAMTS8 effectively inhibited this signaling pathway. Taken together, our findings suggest that DNMT3A activates the EGFR-MEK-ERK signaling pathway by silencing ADAMTS8 transcription through methylation, thereby promoting breast cancer development. Therefore, DNMT3A may serve as an inhibitory target in breast cancer-targeted therapy.

## Introduction

In recent years, the incidence of breast cancer has increased annually, and breast cancer has become the main cause of cancer-related deaths in women around the world [1]. Breast cancer is related to genetic and environmental factors, and complex molecular mechanisms underlie its development [2]. Therefore, different breast cancer subtypes and their mutual transformations lead to differences in tumor progression, metastasis, and disease prognosis among different individuals. Notably, 70–80% of patients with early stage breast cancer are curable, although treatment after advanced metastases remains challenging [3]. Therefore, different breast cancer subtypes and their mutual transformations lead to differences in tumor progression, metastasis, and disease prognosis among various individuals.

A disintegrin and metalloproteinase with thrombospondin motifs (ADAMTS) proteins with platelet-reactive protein motifs are complex extracellular proteases, and 19 ADAMTS proteins have been identified in the human proteome [4]. ADAMTS8, also known as METH-2, is a member of the ADAMTS family and was originally identified as an anti-angiogenic factor. ADAMTS proteins have been implicated in the development of various diseases, including osteoarthritis, cardiovascular disease, and platelet-clotting disease [5]. In addition, ADAMTS has tumor-suppressive and tumor-protective functions [6]. Previous studies have found that ADAMTS8 is down-regulated in colorectal cancer, lung cancer, esophageal cancer, hepatocellular carcinoma, thyroid cancer, breast cancer, and glioma, and is an anticancer gene in various cancers [7–14]. Additionally, ADAMTS8 promoter methylation is significantly elevated in lung and thyroid cancers, suggesting that epigenetic silencing is closely associated with tumorigenesis [15,16].

Epigenetic modifications of DNA can lead to changes in gene expression during aging and cancer development. In human cancers, epigenetic changes such as DNA methylation, histone modification, non-coding RNA, chromatin remodeling, and RNA modification control gene expression [17]. DNA methylation patterns often change during tumorigenesis, usually with CpG island hypermethylation and non-CPG islands hypomethylation [18]. Hypermethylation of tumor suppressor gene promoter regions leads to transcriptional silencing of tumor suppressor genes. Furthermore, the regulation of DNA sequence hypomethylation can increase the expression of proto-oncogenes and promote tumorigenesis [19]. The DNA methyltransferase (DNMT) family catalyzes DNA methylation and includes DNMT1, DNMT3A, and DNMT3B [20]. DNMT3A and DNMT3B are mainly responsible for de novo CpG island methylation, whereas DNMT1 maintains methylation during DNA replication [21]. In earlier studies, DNMT3A was found to be upregulated in melanoma and liver cancer and is considered an oncogene [22,23]. Although DNMT3A is mutated in leukemia, Mayle et al. showed that DNMT3A knockdown mice developed a range of diseases associated with hematological malignancies [24].

To explore the epigenetic regulatory mechanisms involved in breast cancer, we investigated the roles of DNMT3A and ADAMTS8 in breast cancer development and downstream signaling. We discovered that DNMT3A promoted the proliferation, migration, and invasion of breast cancer cells by activating ADAMTS8. Our experiments suggest that DNMT3A promotes breast cancer progression by activating the ADAMTS8-mediated EGFR-MEK-ERK signaling pathway.

## Materials and methods

### Expression analysis of genes

We used the Expression Analysis Module of GEPIA2 (http://gepia2.cancer-pku.cn), with data obtained from The Cancer Genome Atlas (TCGA) and Genotype-Tissue Expression (GTEx) databases, to prepare box plots of gene expression between these tumors and normal tissues. Moreover, GEPIA was used to explore DNMT3A and ADAMTS8 expression in different pathological stages of breast cancer using TCGA database.

### Survival prognosis analysis

The Kaplan-Meier Plotter (https://kmplot.com/analysis/) is an online database that contains microarray datasets. The prognostic value of DNMT3A and ADAMTS8 expression in breast cancer was evaluated based on overall survival (OS) and relapse-free survival (RFS). OS and RFS analyses were performed using the Kaplan–Meier method with a 50% (median) cut-off for gene expression and a 95% confidence interval (CI). Statistical significance was set at *p* < 0.05.

### Tissue specimens

Tissue samples (normal, n = 20; BC, n = 18) were obtained from patients at the Fourth Hospital of Hebei Medical University. According to the exclusion criteria, patients were histopathologically diagnosed with primary breast cancer, were 18–70 years of age, and had tissue stored in liquid nitrogen immediately after breast cancer tumor resection. The clinicopathological features of the patients are shown in S1 Table. This study strictly adhered to the ethical guidelines of the Declaration of Helsinki to ensure the protection of all participants’ rights and well-being and was approved by the Ethics Committee of the Fourth Hospital of Hebei Medical University (2022KS419).

### RNA extraction and quantitative RT-PCR (qRT-PCR)

The tissues were lysed using RedZol (SBS Genetech Co., Ltd. Beijing, China), chloroform was added, and cells were shaken and mixed for 2 min. The supernatant was then added to a centrifuge tube, the same volume of isopropanol was added, and the tube was gently turned upside down four times, mixed well, and allowed to stand for 15 min. The sample was then centrifuged at 12000 rpm for 10 min at low temperature. The supernatant was discarded after centrifugation, 75% ethanol was added, and the sample was washed, precipitated, and then centrifuged at 9000 rpm at 4°C for 5 min. The precipitate was removed and air-dried, and the supernatant was discarded and air-dried for 5 min. Then, 25 μL of enzyme-free water was added, cDNA was synthesized using the PrimeScript™ RT kit (Takara, Beijing, China), and the cDNA was synthesized using the 2×SYBR Green qPCR Master Mix (Servicebio, Wuhan, China) with the iQ5 Real-Time PCR Amplifier (Applied Biosystems, USA). RT-PCR was performed under the following conditions: 95°C for 3 min; 40 cycles of 95°C for 15 s, 60°C for 15 s, 72°C for 15 s; and melting curve analysis. Data were analyzed using the 2^−ΔΔCT^ method for relative quantification. The primer sequences are listed in S2 Table.

### Western blot

Tissues were rinsed in phosphate buffered saline (PBS) (ViviCell, Shanghai, China) and RIPA lysis solution (Zeocin Selection Reagent, Beijing, China) on ice. The supernatants were separated to produce protein extracts, and the samples were subjected to polyacrylamide gel electrophoresis (PAGE) using the BCA technique to determine the protein concentration. Proteins isolated from the gel were transferred to a PVDF membrane (Thermo Fisher Scientific, USA) and blocked with TBST buffer (Bost Biotech, Wuhan, China) mixed with 5% skim milk powder. After rinsing with TBST and incubating with the secondary antibody for 1 h, the sealed membrane was incubated with the primary antibody working solution overnight at 4°C. Protein bands were visualized using an exposure multifunctional imaging system (Shenhuabio, Hangzhou, China) with an ultrasensitive ECL chemiluminescent reagent (NCM Biotech, Suzhou, China). β-actin was used as an endogenous control, and the acquired images were analyzed using ImageJ software. The antibodies used in this study are listed in S3 Table.

### Cell line culture

Six human breast cancer cell lines were used in this study, among which MDA-MB-157, MDA-MB-231, and MDA-MB-453 cells were cultured in Leibovitz’s L-15+10% FBS+1% P/S complete medium at 37°C and 100% in air. MCF-7 cells were cultured in MEM + 10 μg/mL insulin + 10% FBS + 1% P/S growth medium at 37°C in an incubator with 5% CO_2_. T47D cell lines were cultured in RPMI-1640 + 10 μg/mL insulin + 10% FBS + 1% P/S growth medium at 37°C in an incubator with 5% CO_2_. All cell lines were purchased from Pricella (Shanghai, China).

### Cell transfection

DNMT3A resistant small interfering RNAs (si-DNMT3A-1, si-DNMT3A-2, and si-DNMT3A-3, si-ADAMTS8–1, si-ADAMTS8–2, si-ADAMTS8–3, si-NC) were synthesized by RiboBio Biotechnology Co., LTD (Guangzhou, China). Non-targeted sequences were used as negative controls (si-NC). pc-DNMT3A was constructed using pc-DNA3.1, and si-DNMT3A and pc-DNMT3A were transfected with the Lipo6000 Reagent (Beyotime, Shanghai, China). pc-NC and pc-DNMT3A were stably transfected into U87 cells, and the stably transfected cells were screened for G418. The corresponding primer sequences are listed in S4 Table.

### CCK-8 experiment

The cells were washed twice with 2 mL of PBS. Briefly, 1 mL of trypsin was added, and the sample was digested for 5 min. The cell suspension was removed and placed in a centrifuge tube. Complete medium (1 mL) was added, and the mixture was centrifuged at 1000 rpm for 5 min. Cell fluid (10 μL) was taken and counted, and 100 μL cell suspension (2000 cells/well) was added to the 96-well plate and incubated in a controlled incubator for 48 h. Subsequently, 10 μL of CCK-8 solution (Solarbio, Beijing, China) was added to each well, and the samples were incubated for 3 h and then counted at 450 nm. Finally, the absorbance of each sample was measured at 450 nm. Cell viability was calculated as follows: Cell viability (%) = (measured value − blank value)/(control value − blank value) × 100.

### Transwell assay

Matrigel (Solarbio, Beijing, China) was diluted with PBS at a ratio of 1:8 at 4°C, and 100 μL was evenly applied to the upper cavity and left for 1 h at 37°C. Subsequently, 500 μL of medium (containing 20% FBS) was added to the lower cavity of the 24-well plate, the transwell chamber (BIOFIL, China, Guangzhou) was placed in the 24-well plate with tweezers, and 200 μL of cell suspension was added to the upper pore chamber. The cells were then incubated for 48 h. To a new 24-well plate, 600 μL 4% paraformaldehyde was added, the sample was fixed for 20 min, the fixative was removed, and the cells were stained with 0.1%–0.2% crystal violet for 1min, washed with PBS three times, and air-dried. The cells were then observed with a high-power microscope, and finally, the cells were counted with ImageJ.

### Wound-healing assay

Approximately 5 × 10^5^ cells were inoculated into a 6-well cell culture plate (KIRGEN, Shanghai, China) and cultured overnight. The lines were inscribed on the plate using a straight edge. The cells were then washed with sterile PBS 3 times to remove scratched cells. The serum-free medium was replaced with fresh medium, and the cells were cultured in an incubator. After 48 h, the cells were removed, viewed under a microscope, and photographed. After the image was opened using ImageJ software, the gap area was circled to calculate the average distance between cells.

### Co-immunoprecipitation (Co-IP) assay

The MDA-MB-231 cell line was cultured as described above, and nuclear proteins were extracted from the culture dishes. Nuclear protein lysates were shaken with specific antibodies against DNMT3A (Abclonal, Wuhan, China), ADAMTS8 (Huabio, Hangzhou, China), or IgG negative control (Abcam, UK) at 4°C overnight. Then, 50 μL of protein A/G Magnetic Beads (Thermo Fisher Scientific, USA) was added, and the sample was washed using PBST followed by shaking at 4°C for 2 h. Subsequently, the magnetic beads were washed four times with PBST. Western blot was performed after separating the protein complexes from the magnetic beads.

### DNA extraction and methylation-specific PCR (MSP)

We utilized UCSU (http://genome.ucsc.edu/) to find the correct gene sequence (containing the upstream bases of the first exon) and then MethPrimer ADAMTS8 2.0 (http://www.urogene.org/methprimer/index1.html) to analyze the gene promoter CpG islands. We designed ADAMTS8 forward and reverse methyl specific primers, respectively: 5′-GTTGTTGTTGTTGTTGTTGTTGTC-3′, 5′-GCAACACGAAACCCTTACCG-3′. The ADAMTS8 forward and reverse non-methyl-specific primers were 5′-GTTGTTGTTGTTGTTGTTGTTGTT-3′ and 5′-ACACAACACAAAACCCTTACCA-3′, respectively. Genomic DNA was isolated from the cells using a Genomic DNA extraction kit (Tiangen, Beijing, China) according to the manufacturer’s instructions. Sodium bisulfite DNA precipitation was performed using the EpiArt DNA Methylation Bisulfite Kit (Vazyme, Nanjing, China). Subsequently, bisulfite-treated DNA was used as a template for methylation-specific PCR. The PCR conditions were as follows: 94°C for 3 min; and 35 cycles of 94°C for 30 s, 60°C for 15 s, 72°C for 15 s, and 72°C for 5 min. The PCR products were resolved by electrophoresis on 2% agarose gels containing ethidium bromide.

### Statistical analysis

To compare the survival curves, the log-rank test was used to calculate the HR and log-rank *P*-value in the Kaplan–Meier plotter. The correlation between gene expression levels was analyzed using Spearman’s correlation coefficients. Differences with values of *P* < 0.05 were considered statistically significant, and statistical analyses were performed using GraphPad Prism 9.0. The alpha level for all tests was set at 0.05.

## Results

### ADAMTS8 is down-regulated and highly methylated in breast cancer

**Fig 1:**
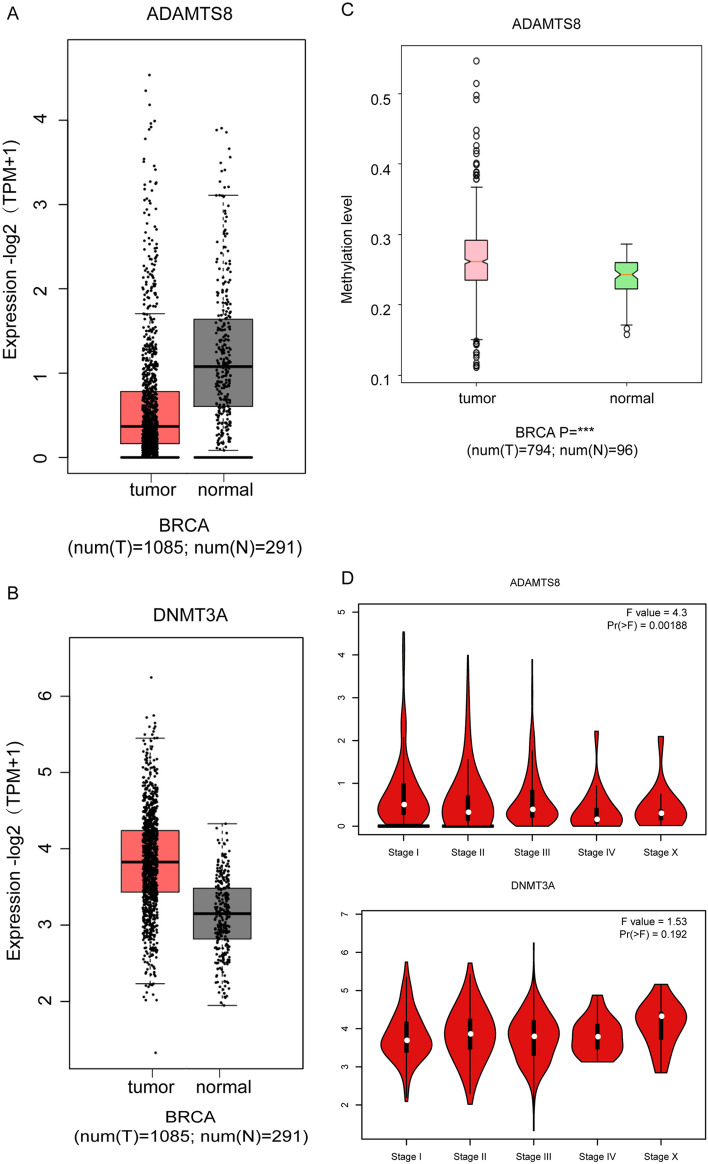
ADAMTS8 is down-regulated and highly methylated in breast cancer. (A) The expressions of ADAMTS8 in breast cancer tissues and normal breast tissues were analyzed based on the TCGA database. (B) The expressions of DNMT3A in breast cancer tissues and normal breast tissues were analyzed based on the TCGA database. (C) MethHC online analysis of ADAMTS8 methylation status in breast cancer. (D) Correlation between mRNA expression of DNMT3A and ADAMTS8 and case stage.

To investigate the association between DNMT3A and ADAMTS8 and breast cancer, we analyzed their expression using the TCGA database. ADAMTS8 was down-regulated in breast cancer tissues (Fig 1A), whereas DNMT3A was upregulated in breast cancer (Fig 1B). MethHC analysis revealed elevated ADAMTS8 methylation levels in breast cancer (Fig 1C). In addition, ADAMTS8 expression correlated with the pathological stage (*P* = 0.00188) (Fig 1D); however, there was no correlation between DNMT3A mRNA expression and the pathological stage of breast cancer.

### The prognostic value of DNMT3A and ADAMTS8 mRNA expression in breast cancer

**Fig 2:**
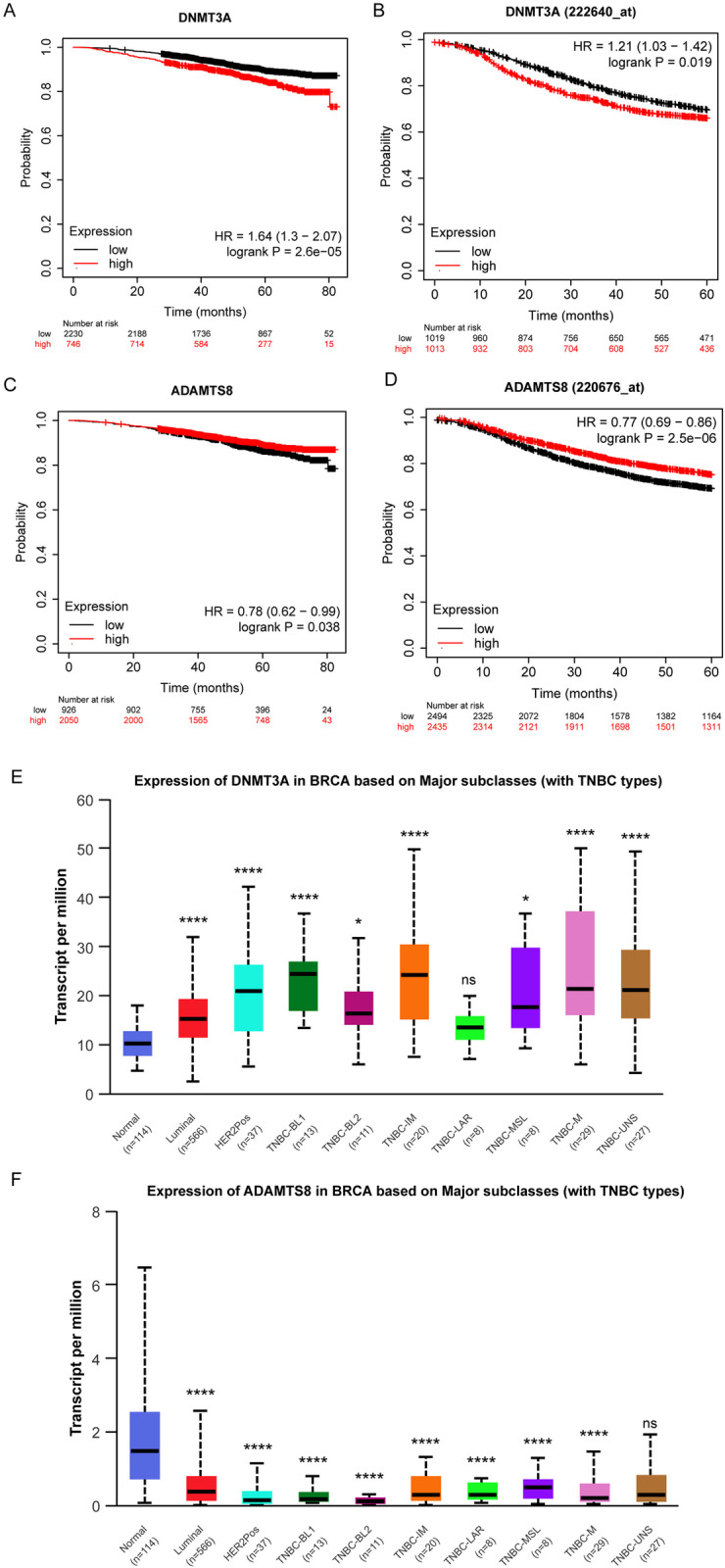
The expressions of DNMT3A and ADAMTS8 were negatively correlated in breast cancer tissues. (A) Correlation between mRNA expression of DNMT3A and OS. (B) Correlation between mRNA expression of DNMT3A and RFS. (C) Correlation between mRNA expression of ADAMTS8 and OS. (D) Correlation between mRNA expression of ADAMTS8 and RFS. (E, F) The expression levels of DNMT3A and ADAMTS8 in different breast cancer subtypes, including Normal, Luminal, HER2-positive and triple-negative breast cancer (TNBC) subtypes (BL1, BL2, IM, LAR, MSL, M, UNS), were analyzed using UALCAN database data. The data are presented as the mean ± S.E.M. *P < 0.01, ****P < 0.0001.

Kaplan-Meier Plotter was used to evaluate the prognostic value of DNMT3A and ADAMTS8 mRNA in breast cancer. Kaplan-Meier survival analysis showed that lower expression levels of DNMT3A mRNA were significantly associated with improved OS (*P *= 0.000026, HR = 1.64), compared to patients with breast cancer with high levels of DNMT3A mRNA expression (Fig 2A). Low DNMT3A mRNA expression was also correlated with improved RFS (*P *= 0.019, HR = 1.12) according to Kaplan-Meier plots (Fig 2B). In addition, high ADAMTS8 mRNA levels were significantly associated with favorable OS (*P*=0.038, HR=0.78) (Fig 2C) and improved RFS (*P *= 0.0000025, HR = 0.77) (Fig 2D). To further explore differences in the expression of DNMT3A and ADAMTS8 in different breast cancer subtypes, we utilized data from the UALCAN database (https://ualcan.path.uab.edu/). Expression levels of DNMT3A and ADAMTS8 were analyzed according to the major breast cancer subtypes, including Normal, Luminal, HER2-positive and triple-negative breast cancer (TNBC) subtypes (BL1, BL2, IM, LAR, MSL, M, UNS). Fig 2E shows the expression levels of DNMT3A in different breast cancer subtypes. We observed that the expression levels of DNMT3A were generally upregulated in all breast cancer subtypes, with the highest levels detected in the TNBC-IM and TNBC-M subtypes. This pattern of differential expression suggests a potential role for DNMT3A in the progression of specific breast cancer subtypes, particularly those that are more aggressive. Fig 2F shows ADAMTS8 expression levels in the same breast cancer subtype. Interestingly, ADAMTS8 expression was generally lower in the TNBC subtype compared to the normal and Luminal subtypes. Expression was lowest in the TNBC-IM subtype, which is known for poor prognosis and aggressive behavior. This finding is consistent with our previous findings showing that ADAMTS8 downregulation is associated with breast cancer progression.

### DNMT3A and ADAMTS8 expression negatively correlate in breast cancer

**Fig 3:**
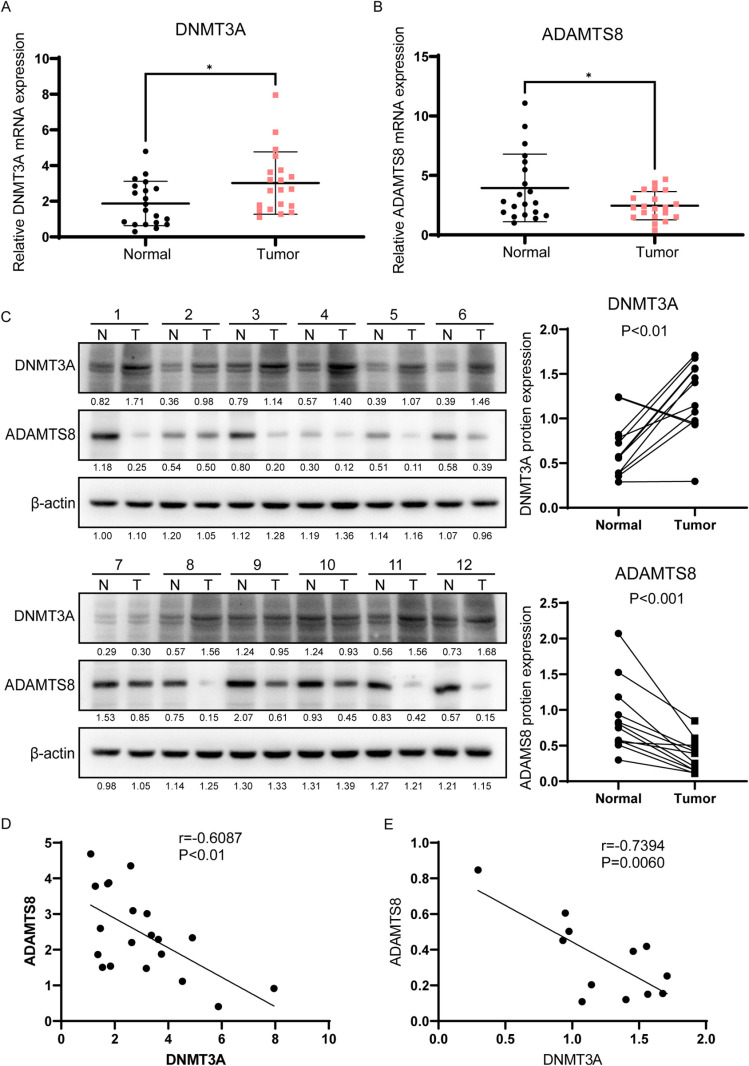
The expressions of DNMT3A and ADAMTS8 were negatively correlated in breast cancer tissues. (A) qRT-PCR analysis of the relative mRNA levels of DNMT3A in BC and paired adjacent normal tissues (n = 20). (B) qRT-PCR analysis of the relative mRNA levels of ADAMTS8 in BC and paired adjacent normal tissues (n = 20). (C) The protein levels of DNMT3A and ADAMTS8 in breast cancer and matched normal tissues were detected by western blot. Quantitative determination of relative protein levels using ImageJ software (n = 12). (D) Pearson correlation analysis of DNMT3A and ADAMTS8 expression at mRNA level in breast cancer tissues. (E) Pearson correlation analysis of DNMT3A and ADAMTS8 expression at protein level in breast cancer tissues. The data are presented as the mean ± S.E.M. *P < 0.05.

DNMT3A and ADAMTS8 expression levels were detected by qRT-PCR. The results showed that the mRNA expression of DNMT3A in breast cancer tissues was increased (Fig 3A), whereas the mRNA expression of ADAMTS8 was decreased compared to that in the neighboring normal tissues (Fig 3B). We further used western blot to evaluate the expression of DNMT3A and ADAMTS8 proteins in breast cancer Figsand paired normal tissues (Fig 3C), and the results were consistent with those of qRT-PCR. Statistical analysis revealed that the mRNA levels of DNMT3A and ADAMTS8 were negatively correlated in BC tissues (Fig 3D). Furthermore, correlation analysis showed that DNMT3A and ADAMTS8 were negatively expressed in breast cancer and adjacent tissues (Fig 3E).

### ADAMTS8 expression is down-regulated by DNMT3A

**Fig 4:**
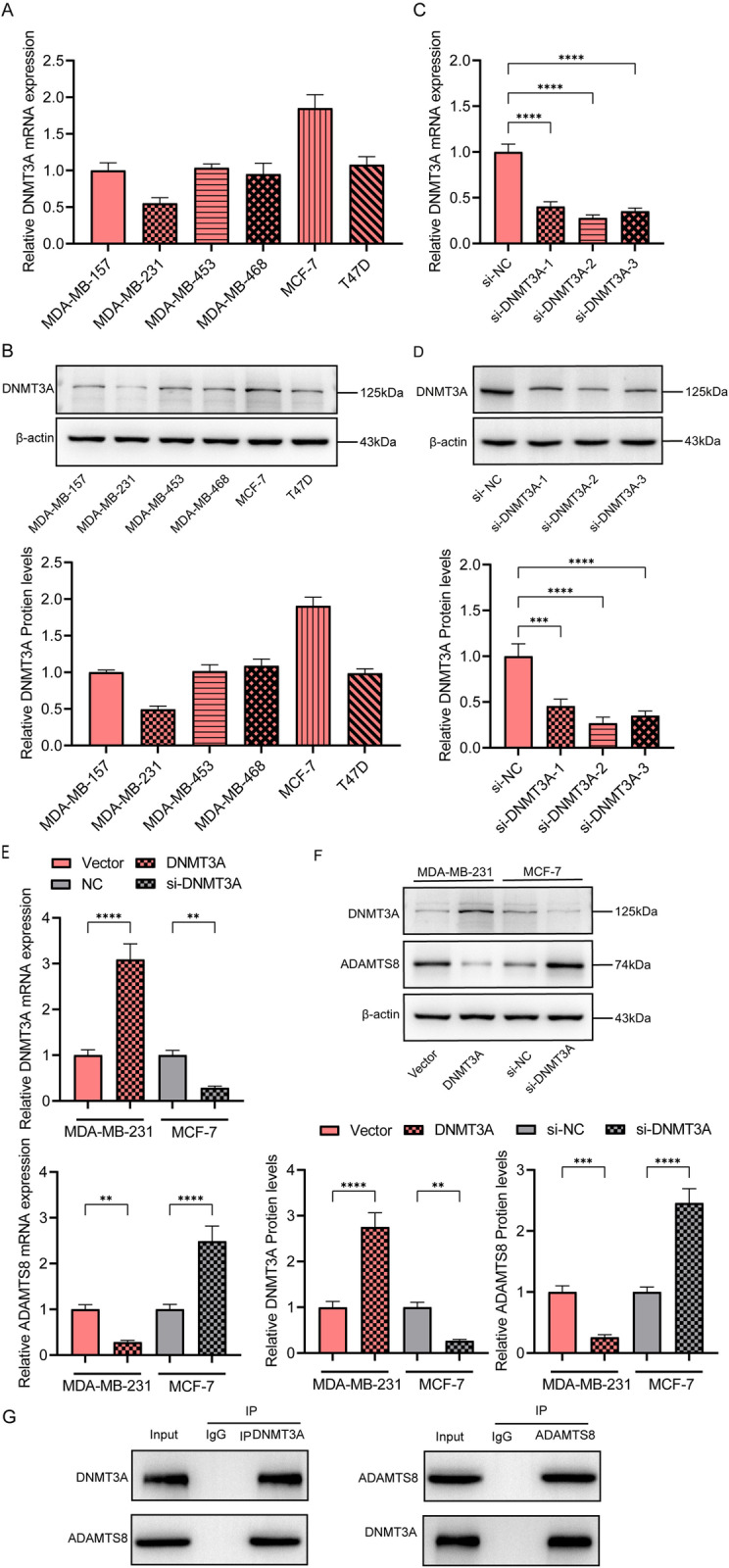
DNMT3A promotes proliferation, migration, invasion and apoptosis of breast cancer cells. (A) The relative expression of DNMT3A mRNA in breast cancer cell lines was detected by qRT-PCR. (B) The protein levels of DNMT3A protein in breast cancer cell lines was detected by western blot. (MCF-7 cell line was selected to silence DNMT3A, and MDA-MB-231 cells were selected to overexpression DNMT3A.) (C) The si-DNMT3A-1, si-DNMT3A-2 and si-DNMT3A-3 sequences were designed to silence DNMT3A. (D) Western blot was used to detect the silencing efficiency of 3 si-DNMT3A sequences (E) Gene expression levels of DNMT3A and ADAMTS8 in breast cancer cells were detected by qRT-PCR. (F) The protein expression levels of DNMT3A and ADAMTS8 in breast cancer cells were detected by western blot. (G) Co-IP assays were used to detect the direct interaction of DNMT3A and ADAMTS8. The data are presented as the mean ± S.E.M. **P < 0.01, ***P < 0.001,****P < 0.0001.

We examined the relative DNMT3A mRNA and protein expression levels in various breast cancer cell lines. We found that the relative expression level of DNMT3A was the lowest in the MDA-MB-231 cell line and the highest in the MCF-7 cell line (Fig 4A and 4B). To evaluate the pathological significance of DNMT3A, MCF-7 cells were selected to silence DNMT3A expression, whereas MDA-MB-231 cells were selected to overexpress DNMT3A. Fig 4C and 4D show the knockdown efficiency of DNMT3A. si-DNMT3A-2 was selected for subsequent knockdown experiments. We found that the relative expression levels of ADAMTS8 mRNA and protein in the DNMT3A overexpression group were significantly lower than those in the control groupFigs, whereas the opposite results were observed in the DNMT3A knockdown group (Fig 4E–F). Co-IP results showed that ADAMTS8 directly interacted with DNMT3A in cells (Fig 4G).

### DNMT3A promotes breast cancer cell proliferation, migration, invasion, and apoptosis

**Fig 5:**
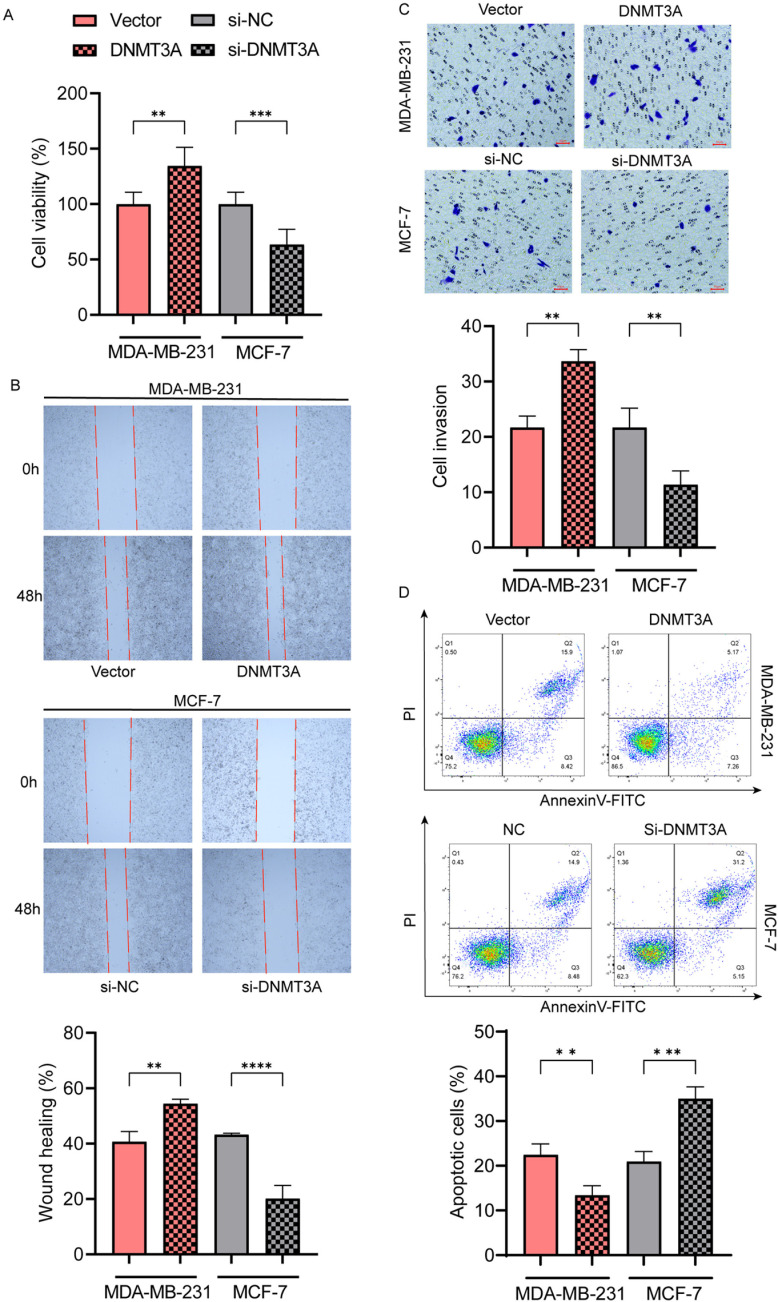
DNMT3A mediates ADAMTS8 methylation and regulates the EGFR-MEK-ERK pathway. (A) CCK8 assay was applied to evaluate the vitality of MCF-7 and MDA-MB-231cells transfected with DNMT3A siRNA or DNMT3A plasmids. (B) The cell wound-healing assay was used to test the cell migration capacity of MDA-MB-231 and MCF-7 cells. (C) The transwell assay was used to test the cell invasion capacity of MDA-MB-231 and MCF-7 cells. (D) Flow cytometry was performed to examine the apoptosis in breast cancer cells. The data were shown as the mean ± SEM. **P < 0.01, ***P < 0.001, ****P < 0.0001.

Subsequently, we used the CCK8 assay to investigate the effect of DNMT3A on breast cancer cell proliferation (Fig 5A). The results showed that DNMT3A overexpression significantly enhanced the viability of breast cancer cells compared to that of the negative control, whereas DNMT3A knockdown significantly weakened the viability of breast cancer cells. In addition, we conducted wound-healing and transwell assays to evaluate the effects of DNMT3A on breast cancer cell migration and invasion, respectively. The wound-healing assay showed that the healing rate of cells in the DNMT3A knockdown group was lower than that in the control group, whereas the healing rate of cells in the DNMT3A overexpression group was significantly higher than that in the control group (Fig 5B). The transwell assay was used to test cell invasion capacity and revealed that DNMT3A overexpression clearly increased the invasion capacity of MDA-MB-231 and MCF-7 cells, whereas the cell invasion ability of the DNMT3A knockdown group was the lowest (Fig 5C). Apoptotic cells were analyzed using annexin V-FITC and propidium iodide (PI) staining. Compared to the control group, the number of apoptotic breast cancer cells decreased upon DNMT3A silencing (Fig 5D).

### DNMT3A mediates ADAMTS8 methylation and regulates the downstream EGFR-MEK-ERK pathway

**Fig 6:**
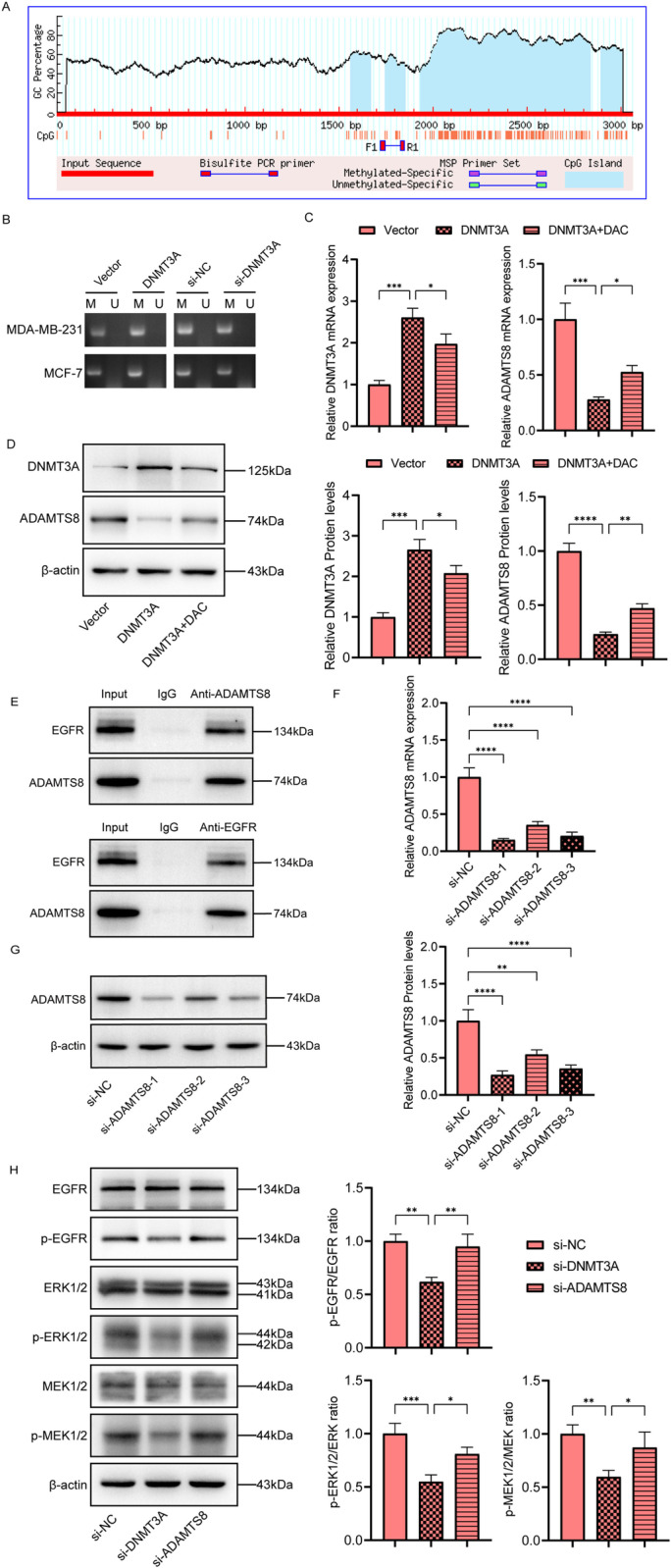
DNMT3A mediates ADAMTS8 methylation and regulates the EGFR-MEK-ERK pathway. (A) The online prediction of the CpG island in the ADAMTS8 promoter region. (B) MSP assay was used to software MethPrimer was used to predict the location detect the methylation level of ADAMTS8 promoter. (C) qRT-PCR analysis of the relative mRNA levels of DNMT3A and ADAMTS8 in MDA-MB-231 cells. (D) The protein levels of DNMT3A and ADAMTS8 in MDA-MB-231 cells. (E) Co-IP assays were used to detect the direct interaction of ADAMTS8 and EGFR. (F) The si-ADAMTS8-1, si-ADAMTS8-2 and si-ADAMTS8-3 sequences were designed to silence ADAMTS8. (G) Western blot was used to detect the silencing efficiency of 3 si-ADAMTS8 sequences. (H) The protein levels of EGFR-MEK-ERK pathway in MCF-7 cells. The data were shown as the mean ± SEM. *P < 0.05, **P < 0.01,***P < 0.001, ****P < 0.0001.

To determine whether DNA methylation is involved in ADAMTS8 repression in breast cancer, we analyzed the methylation status of the ADAMTS8 promoter using MSP. MethPrimer (http://www.urogene.org/methprimer/) was used to predict the promoter CpG island in ADAMTS8 (Fig 6A). The results showed that DNMT3A silencing attenuated ADAMTS8 promoter methylation, whereas DNMT3A overexpression enhanced ADAMTS8 methylation (Fig 6B). Furthermore, we found that the mRNA and protein levels of ADAMTS8 were significantly elevated in DNMT3A overexpression breast cancer cells. The application of the demethylating drug decitabine neutralized the inhibitory effect of DNMT3A on ADAMTS8 expression (Fig 6C and D). Overall, our data suggest that DNMT3A-mediated DNA methylation is responsible for ADAMTS8 inhibition.

The role of the EGFR-MEK-ERK pathway in promoting breast cancer has been preliminarily explored [25]. In addition, existing studies have shown that ADAMTS8 exhibits anti-tumor properties by antagonizing the EGFR-MEK-ERK signaling pathway in various cancers. For more details, please refer to [11,26]. Therefore, we decided to investigate its role in breast cancer in depth. We conducted co-immunoprecipitation assays using antibodies specific to ADAMTS8 and EGFR. The results revealed that both ADAMTS8 and EGFR were prominently expressed in the input samples. In the IP samples, the corresponding proteins were only detected when antibodies specific to ADAMTS8 or EGFR were used (Fig 6E). This finding indicates that there is a direct interaction between ADAMTS8 and EGFR. Subsequently, Subsequently, we evaluated the regulatory mechanisms of the EGFR-MEK-ERK pathway by knocking down DNMT3A and ADAMTS8 (Fig 6F and G). In MCF-7 cells, DNMT3A silencing decreased the activation of the EGFR-MEK-ERK pathway, as evidenced by reduced levels of p-EGFR, p-ERK1/2, and p-MEK1/2, while ADAMTS8 silencing reversed this effect, suggesting that DNMT3A and ADAMTS8 exert opposing regulatory roles on the pathway (Fig 6H).

## Discussion

Epigenetic modifications play an increasingly important role in the development of malignant tumors [27]. Abnormal DNA methylation is often associated with a range of human diseases such as cancer. In mammals, the methylation site is located on the CpG island of the gene promoter region, and hypermethylation of the promoter region ultimately leads to transcriptional silencing of tumor suppressor genes by blocking transcription factor recognition and binding [28]. In non-small cell lung cancer, KMT2C (a histone H3 lysine 4 methyltransferase) directly regulates DNMT3A expression through histone methylation, and forced DNMT3A overexpression inhibits cancer metastasis by reducing the activity of MEIS/HOX genes [29]. In addition, DNMT3A has been shown to target the MEIS1 promoter via lncRNA ELNF1-AS1 to epigenetically inhibit MEIS1 transcription and thus colorectal cancer cells proliferation [30]. Published cancer-related data show that the expression level of DNMT3A in breast cancer tissues is significantly higher than that in the corresponding adjacent tissues and is correlated with OS and RFS in breast cancer. Furthermore, our study showed that DNMT3A was significantly upregulated in both breast cancer tissues and cells. In addition, DNMT3A promotes the proliferation, migration, invasion, and apoptosis of breast cancer cells. Li et al. also reported that overexpression of DNMT3A can regulate the proliferation, metastasis, and glycolysis of tumors [31]. However, the potential carcinogenic mechanism of DNMT3A in breast cancer has not yet been elucidated. In our study, we found that the relative expression level of DNMT3A in the MCF-7 cell line was significantly higher than that in other cell lines. Therefore, we selected the MCF-7 cell line for DNMT3A silencing experiments, which enabled us to effectively evaluate the impact of DNMT3A knockdown on downstream signaling pathways and cellular behaviors. MCF-7 cells are widely used in breast cancer research due to their well-characterized estrogen receptor (ER)-positive status, which is representative of a significant subset of breast cancer cases. In addition, DNA methylation plays a crucial role in the recurrence of breast cancer. Studies have shown that ER-positive tumors typically exhibit higher levels of methylation, especially in primary tumors [32]. Moreover, DNMT3A is involved in the regulation of estrogen-responsive genes [33]. Therefore, by conducting DNMT3A silencing experiments using MCF-7 cells, we aim to elucidate the role of DNMT3A in a representative and significant subgroup of breast cancer cases.

Methen-2 promoter regions show hypermethylated alleles in most tumors, suggesting that DNA methylation causes METHEN-2 transcriptional inactivation in non-small cell lung cancer [15]. Rodriguez-Rodero et al. used a DNA methylation array to detect the methylation status of gene promoters in thyroid tumors and showed that ADAMTS8 was significantly hypermethylated and had an obvious tumor-inhibitory effect [16]. In addition, miRNAs inhibit ADAMTS8 expression via post-transcriptional regulation. For example, lncRNA OIP22-AS5 regulates ADAMTS8 expression by sponging miR-98, resulting in the proliferation, migration, and invasion of thyroid cancer cells [11]. However, it remains unclear whether DNMT3A epigenetically regulates ADAMTS8 expression in breast cancer has not been determined. In the present study, we observed a significant inverse association between DNMT3A and ADAMTS8 expression levels in breast cancer cells. We also explored whether DNMT3A regulates ADAMTS8 expression via DNA methylation. After DNMT3A overexpression in breast cancer cells, ADAMTS8 expression was significantly decreased, whereas DNMT3A knockdown restored ADAMTS8 expression. Furthermore, we determined the interaction between DNMT3A and ADAMTS8 using immunoprecipitation. In addition, DNA extraction and methylation-specific PCR experiments showed that DNMT3A silencing weakened the methylation status of the ADAMTS8 promoter, whereas DNMT3A overexpression enhanced ADAMTS8 methylation. Therefore, we preliminarily elucidated the mechanism by which DNMT3A regulates ADAMTS8 expression in breast cancer.

The EGFR-MEK-ERK pathway is a conserved signaling pathway that plays an important role in cell proliferation and differentiation. Previous studies have shown that RNF128 activates the EGFR-MEK-ERK signaling pathway to promote the progression of liver cancer [34]. In addition, the targeting of miRNAs leads to dysregulation of Med19 expression in breast cancer, which promotes breast cancer progression by interacting with EGFR and activating the MEK-ERK signaling pathway [25]. Our research findings reveal a negative correlation between DNMT3A and ADAMTS8 expression, and indicate that this relationship is associated with poor prognosis in breast cancer. Furthermore, our discoveries suggest that DNMT3A activates the EGFR-MEK-ERK signaling pathway by silencing ADAMTS8 transcription through methylation, thereby further promoting the development of breast cancer. This finding is consistent with previous research that showed DNMT3A activates MEK-ERK signaling in colorectal cancer [35]. These studies demonstrate that the EGFR-MEK-ERK signaling pathway plays a crucial role in cell proliferation and differentiation, and is activated in various cancer types to promote tumor progression. This also provides new insights into the molecular mechanisms of breast cancer and offers potential targets for the development of new therapeutic strategies. Although this study provides strong evidence for the role of DNMT3A in breast cancer, there are some limitations. Our research primarily focused on breast cancer cell lines, and future studies should consider the heterogeneity of different breast cancer subtypes and patient samples to gain a more comprehensive understanding of the roles of DNMT3A and ADAMTS8.

## Conclusion

In summary, our findings suggest that DNMT3A is inversely associated with ADAMTS8 expression in breast cancer and that both are associated with adverse outcomes. In addition, DNMT3A can silence ADAMTS8 transcription through methylation, thereby activating the EGFR-MEK-ERK signaling pathway and further promoting the development of breast cancer. Therefore, DNMT3A may serve as an inhibitory target in breast cancer-targeted therapy.

## Supporting information

S1 Table Clinicopathologic characteristics of patients. (DOCX)

S2 Table The primer sequences of qRT-PCR. (DOCX)

S3 Table Antibody information used for western blot. (DOCX)

S4 Table The primer sequences of siRNA and pcDNA3.1 DNMT3A sequence. (DOCX)

S1 Raw Images The original image of uncropped western blot. (PDF)

## References

[1] Xu Y, Gong M, Wang Y, Yang Y, Liu S, Zeng Q. Global trends and forecasts of breast cancer incidence and deaths. Sci Data. 2023;10(1):334. doi: 10.1038/s41597-023-02253-5 37244901 PMC10224917

[2] Nolan E, Lindeman GJ, Visvader JE. Deciphering breast cancer: from biology to the clinic. Cell. 2023;186(8):1708–28. doi: 10.1016/j.cell.2023.01.040 36931265

[3] Zannetti A. Breast cancer: from pathophysiology to novel therapeutic approaches 2.0. Int J Mol Sci. 2023;24(3):2542. doi: 10.3390/ijms24032542 36768866 PMC9916418

[4] Wang Z, Li W, Chen S, Tang XX. Role of ADAM and ADAMTS proteases in pathological tissue remodeling. Cell Death Discov. 2023;9(1):447. doi: 10.1038/s41420-023-01744-z 38071234 PMC10710407

[5] Mead TJ, Apte SS. ADAMTS proteins in human disorders. Matrix Biol. 2018;71–72:225–39. doi: 10.1016/j.matbio.2018.06.002 29885460 PMC6146047

[6] Cal S, López-Otín C. ADAMTS proteases and cancer. Matrix Biol. 2015;44–46:77–85. doi: 10.1016/j.matbio.2015.01.013 25636539

[7] Li L, Yuan S, Zhao X, Luo T. ADAMTS8 is frequently down-regulated in colorectal cancer and functions as a tumor suppressor. Biochem Biophys Res Commun. 2020;524(3):663–71. doi: 10.1016/j.bbrc.2020.01.020 32033751

[8] Zhang Y, Hu K, Qu Z, Xie Z, Tian F. ADAMTS8 inhibited lung cancer progression through suppressing VEGFA. Biochem Biophys Res Commun. 2022;598:1–8. doi: 10.1016/j.bbrc.2022.01.110 35149432

[9] Wu Z, Shi Y, Ren S, Ju Y, Hu Y, Wu J. ADAMTS8 inhibits progression of esophageal squamous cell carcinoma. DNA Cell Biol. 2020. doi: 10.1089/dna.2020.6053 33054388

[10] Zhao X, Yang C, Wu J, Nan Y. ADAMTS8 targets ERK to suppress cell proliferation, invasion, and metastasis of hepatocellular carcinoma. Onco Targets Ther. 2018;11:7569–78. doi: 10.2147/OTT.S173360 30464505 PMC6214590

[11] Zhang X, Li D, Jia C, Cai H, Lv Z, Wu B. METTL14 promotes tumorigenesis by regulating lncRNA OIP5-AS1/miR-98/ADAMTS8 signaling in papillary thyroid cancer. Cell Death Dis. 2021;12(6):617. doi: 10.1038/s41419-021-03891-6 34131102 PMC8206147

[12] Zhang K, Tian R, Wang G, Zhang J, Ma H, Hu X, et al. ADAMTS8 inhibits cell proliferation and invasion, and induces apoptosis in breast cancer. Onco Targets Ther. 2020;13:8373–82. doi: 10.2147/OTT.S248085 32904790 PMC7457586

[13] Li Y, Yang X, Sun J, Zhao Y, Zhou Q, Hua B. ADAMTS8 expression is a potential prognostic biomarker for postoperative metastasis in lymph node-negative early-stage invasive breast carcinoma patients. Pharmgenomics Pers Med. 2021;14:1701–13. doi: 10.2147/PGPM.S339919 35002288 PMC8722701

[14] Zhou B, Liu Y, Ma G, Wang X, Chang B, Lu H, et al. ADAMTS8 inhibits glioma development in vitro and in vivo. Folia Neuropathol. 2023;61(2):144–52. doi: 10.5114/fn.2023.129380 37587889

[15] Dunn JR, Panutsopulos D, Shaw MW, Heighway J, Dormer R, Salmo EN, et al. METH-2 silencing and promoter hypermethylation in NSCLC. Br J Cancer. 2004;91(6):1149–54. doi: 10.1038/sj.bjc.6602107 15328519 PMC2747718

[16] Rodríguez-Rodero S, Fernández AF, Fernández-Morera JL, Castro-Santos P, Bayon GF, Ferrero C, et al. DNA methylation signatures identify biologically distinct thyroid cancer subtypes. J Clin Endocrinol Metab. 2013;98(7):2811–21. doi: 10.1210/jc.2012-3566 23666970

[17] Ilango S, Paital B, Jayachandran P, Padma PR, Nirmaladevi R. Epigenetic alterations in cancer. Front Biosci (Landmark Ed). 2020;25(6):1058–109. doi: 10.2741/4847 32114424

[18] Chen B-F, Chan W-Y. The de novo DNA methyltransferase DNMT3A in development and cancer. Epigenetics. 2014;9(5):669–77. doi: 10.4161/epi.28324 24589714 PMC4063825

[19] Skvortsova K, Stirzaker C, Taberlay P. The DNA methylation landscape in cancer. Essays Biochem. 2019;63(6):797–811. doi: 10.1042/EBC20190037 31845735 PMC6923322

[20] Hoang N-M, Rui L. DNA methyltransferases in hematological malignancies. J Genet Genom. 2020;47(7):361–72. doi: 10.1016/j.jgg.2020.04.006 32994141 PMC7704698

[21] Zhang Z-M, Lu R, Wang P, Yu Y, Chen D, Gao L, et al. Structural basis for DNMT3A-mediated de novo DNA methylation. Nature. 2018;554(7692):387–91. doi: 10.1038/nature25477 29414941 PMC5814352

[22] Deng T, Kuang Y, Wang L, Li J, Wang Z, Fei J. An essential role for DNA methyltransferase 3a in melanoma tumorigenesis. Biochem Biophys Res Commun. 2009;387(3):611–6. doi: 10.1016/j.bbrc.2009.07.093 19632198

[23] Oh B-K, Kim H, Park H-J, Shim Y-H, Choi J, Park C, et al. DNA methyltransferase expression and DNA methylation in human hepatocellular carcinoma and their clinicopathological correlation. Int J Mol Med. 2007;20(1):65–73. 17549390

[24] Mayle A, Yang L, Rodriguez B, Zhou T, Chang E, Curry CV, et al. Dnmt3a loss predisposes murine hematopoietic stem cells to malignant transformation. Blood. 2015;125(4):629–38. doi: 10.1182/blood-2014-08-594648 25416277 PMC4304108

[25] Zhang X, Gao D, Fang K, Guo Z, Li L. Med19 is targeted by miR-101-3p/miR-422a and promotes breast cancer progression by regulating the EGFR/MEK/ERK signaling pathway. Cancer Lett. 2019;444:105–15. doi: 10.1016/j.canlet.2018.12.008 30583076

[26] Slotkin EK, Bowman AS, Levine MF, Dela Cruz F, Coutinho DF, Sanchez GI, et al. Comprehensive molecular profiling of desmoplastic small round cell tumor. Mol Cancer Res. 2021;19(7):1146–55. doi: 10.1158/1541-7786.MCR-20-0722 33753552 PMC8293793

[27] You JS, Jones PA. Cancer genetics and epigenetics: two sides of the same coin?. Cancer Cell. 2012;22(1):9–20. doi: 10.1016/j.ccr.2012.06.008 22789535 PMC3396881

[28] Deaton AM, Bird A. CpG islands and the regulation of transcription. Genes Dev. 2011;25(10):1010–22. doi: 10.1101/gad.2037511 21576262 PMC3093116

[29] Na F, Pan X, Chen J, Chen X, Wang M, Chi P, et al. KMT2C deficiency promotes small cell lung cancer metastasis through DNMT3A-mediated epigenetic reprogramming. Nat Cancer. 2022;3(6):753–67. doi: 10.1038/s43018-022-00361-6 35449309 PMC9969417

[30] Li Y, Gan Y, Liu J, Li J, Zhou Z, Tian R, et al. Downregulation of MEIS1 mediated by ELFN1-AS1/EZH2/DNMT3a axis promotes tumorigenesis and oxaliplatin resistance in colorectal cancer. Signal Transduct Target Ther. 2022;7(1):87. doi: 10.1038/s41392-022-00902-6 35351858 PMC8964798

[31] Li H, Gao P, Chen H, Zhao J, Zhang X, Li G, et al. HOXC13 promotes cell proliferation, metastasis and glycolysis in breast cancer by regulating DNMT3A. Exp Ther Med. 2023;26(3):439. doi: 10.3892/etm.2023.12138 37614427 PMC10443053

[32] Williams KE, Jawale RM, Schneider SS, Otis CN, Pentecost BT, Arcaro KF. DNA methylation in breast cancers: differences based on estrogen receptor status and recurrence. J Cell Biochem. 2019;120(1):738–55. doi: 10.1002/jcb.27431 30230580

[33] Yu Z, Xiao Q, Zhao L, Ren J, Bai X, Sun M, et al. DNA methyltransferase 1/3a overexpression in sporadic breast cancer is associated with reduced expression of estrogen receptor-alpha/breast cancer susceptibility gene 1 and poor prognosis. Mol Carcinog. 2015;54(9):707–19. doi: 10.1002/mc.22133 24464625

[34] Bai X-S, Zhang C, Peng R, Jiang G-Q, Jin S-J, Wang Q, et al. RNF128 promotes malignant behaviors via EGFR/MEK/ERK pathway in hepatocellular carcinoma. Onco Targets Ther. 2020;13:10129–41. doi: 10.2147/OTT.S269606 33116595 PMC7553654

[35] Zhou Y, Yang Z, Zhang H, Li H, Zhang M, Wang H, et al. DNMT3A facilitates colorectal cancer progression via regulating DAB2IP mediated MEK/ERK activation. Biochim Biophys Acta Mol Basis Dis. 2022;1868(4):166353. doi: 10.1016/j.bbadis.2022.166353 35063646

