# Supplementary material for: DNMT3A facilitates breast cancer progression via regulating ADAMTS8 mediated EGFR-MEK-ERK activation
Source: PLoS One. 2025 May 5;20(5):e0321889. doi: 10.1371/journal.pone.0321889 (PMC12052109; doi:10.1371/journal.pone.0321889)
Supplement: S1 Table — (DOCX) [file pone.0321889.s001.docx]

| Characteristics | ER+(n=9) | HER2+(n=4) | TNBC(n=7) |
| --- | --- | --- | --- |
| Age(mean±SD) | 57.8±8.4 | 57.5±9.0 | 62.7±10.4 |
| Histologic grade |  |  |  |
| 1-2 | 3 | 1 | 2 |
| 3 | 6 | 3 | 5 |
| TNM stage^a^ |  |  |  |
| I -II | 4 | 2 | 3 |
| III | 5 | 2 | 4 |
| Lymph node |  |  |  |
| Positive | 6 | 3 | 5 |
| Negative | 3 | 1 | 2 |
| Histologic type |  |  |  |
| Ductal | 8 | 3 | 6 |
| Lobular | 1 | 1 | 1 |

**S1 Table. Clinicopathologic characteristics of patients**
