# Supplementary material for: DNMT3A facilitates breast cancer progression via regulating ADAMTS8 mediated EGFR-MEK-ERK activation
Source: PLoS One. 2025 May 5;20(5):e0321889. doi: 10.1371/journal.pone.0321889 (PMC12052109; doi:10.1371/journal.pone.0321889)
Supplement: S2 Table — (DOCX) [file pone.0321889.s002.docx]

| Gene name | （5‘to 3’） | Sequence |
| --- | --- | --- |
| ADAMTS8 | Forward | GTGACCCCAACAAAAGCTGC |
|  | Reverse | GGACGTGCCCTAGTTCATGG |
| DNMT3A | Forward | CCGATGCTGGGGACAAGAAT |
|  | Reverse | CCCGTCATCCACCAAGACAC |
| GAPDH | Forward | GGAGCGAGATCCCTCCAAAAT |
|  | Reverse | GGCTGTTGTCATACTTCTCATGG |

**S2 Table. The primer sequences of qRT-PCR**
