# Supplementary material for: DNMT3A facilitates breast cancer progression via regulating ADAMTS8 mediated EGFR-MEK-ERK activation
Source: PLoS One. 2025 May 5;20(5):e0321889. doi: 10.1371/journal.pone.0321889 (PMC12052109; doi:10.1371/journal.pone.0321889)
Supplement: S3 Table — (DOCX) [file pone.0321889.s003.docx]

**S3 Table. Antibody information used for western blot**

| Antibody | Dilution ratio | Product number | Manufacturer |
| --- | --- | --- | --- |
| DNMT3A | 1/1000 | A2065 | Abclonal |
| ADAMTS8 | 1/1000 | ER1903-34 | Huabio |
| EGFR | 1/1000 | HY-P80116 | MedChemExpress |
| P-EGFR | 1/1000 | HY-P80808 | MedChemExpress |
| ERK1/2 | 1/1000 | ET1601-29 | Huabio |
| P-ERK1/2 | 1/1000 | AF1015 | Affinity |
| MEK1/2 | 1/1000 | 11049-1-AP | Proteintech |
| P-MEK1/2 | 1/1000 | sc-81503 | Santa cruz |
| β-actin | 1/10000 | T0022 | Affinity |
| Goat Anti-Rabbit IgG (H+L) HRP | 1/5000 | S0001 | Affinity |
| Goat Anti-Mouse IgG (H+L) HRP | 1/5000 | S0002 | Affinity |
