# Supplementary material for: DNMT3A facilitates breast cancer progression via regulating ADAMTS8 mediated EGFR-MEK-ERK activation
Source: PLoS One. 2025 May 5;20(5):e0321889. doi: 10.1371/journal.pone.0321889 (PMC12052109; doi:10.1371/journal.pone.0321889)
Supplement: S4 Table — (DOCX) [file pone.0321889.s004.docx]

| Gene name | Sequence |
| --- | --- |
| si-DNMT3A-1 | GCUGUAAACAUGUUAGUAA |
| si-DNMT3A-2 | CGAUUGCUAGACUGGGAUA |
| si-DNMT3A-3 | GAACAUUUCCAGAAUACAA |
| si-ADAMTS8-1 | GCAUCAAGAAUUCCAUCAACC |
| si-ADAMTS8-2 | GGAGCGAGUUCAAAGUGUUCG |
| si-ADAMTS8-3 | GCGAGUUCAAAGUGUUCGAGG |
| si-NC | CGAUUGCUAGACUGAGAUA |
| pcDNA3.1 DNMT3A（Forward） | TACCGAGCTCGGATCCATGCCCGCCATGCCCTC |
| pcDNA3.1 DNMT3A（Reverse） | GATATCTGCAGAATTCTCAGGGTATGCTGGTGGGC |

**S4 Table. The primer sequences of siRNA and pcDNA3.1 DNMT3A sequence**
