# Supplementary material for: DNMT3A facilitates breast cancer progression via regulating ADAMTS8 mediated EGFR-MEK-ERK activation
Source: PLoS One. 2025 May 5;20(5):e0321889. doi: 10.1371/journal.pone.0321889 (PMC12052109; doi:10.1371/journal.pone.0321889)
Supplement: S1 Raw Images — (PDF) [file pone.0321889.s005.pdf]

Figure 3C

DNMT3A

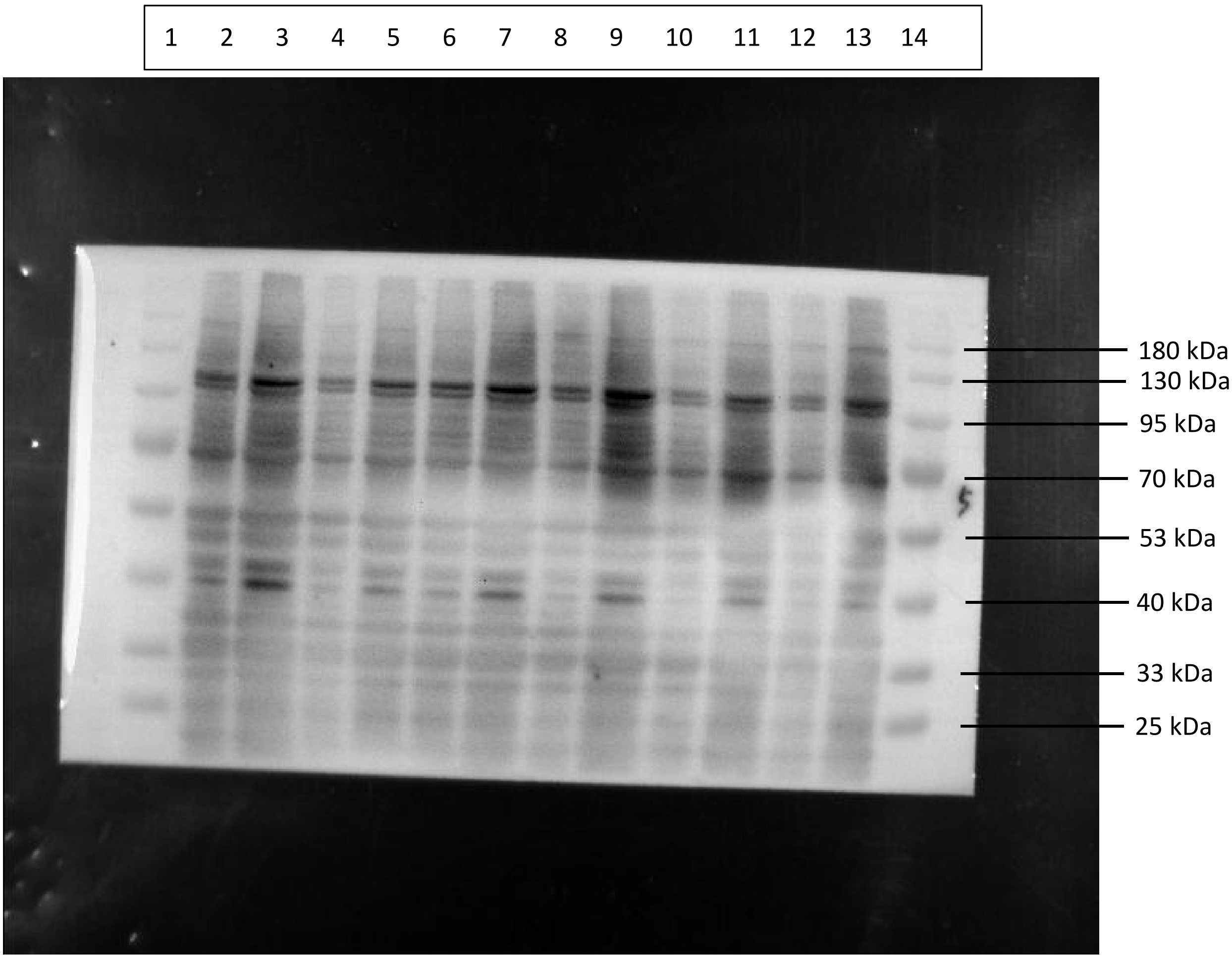

Lane1：Protein marker

Lane2：N

Lane3：T

Lane4：N

Lane5：T

Lane6：N

Lane7：T

Lane8：N

Lane9：T

Lane10：N

Lane11：T

Lane12：N

Lane13：T

Lane14：Protein marker

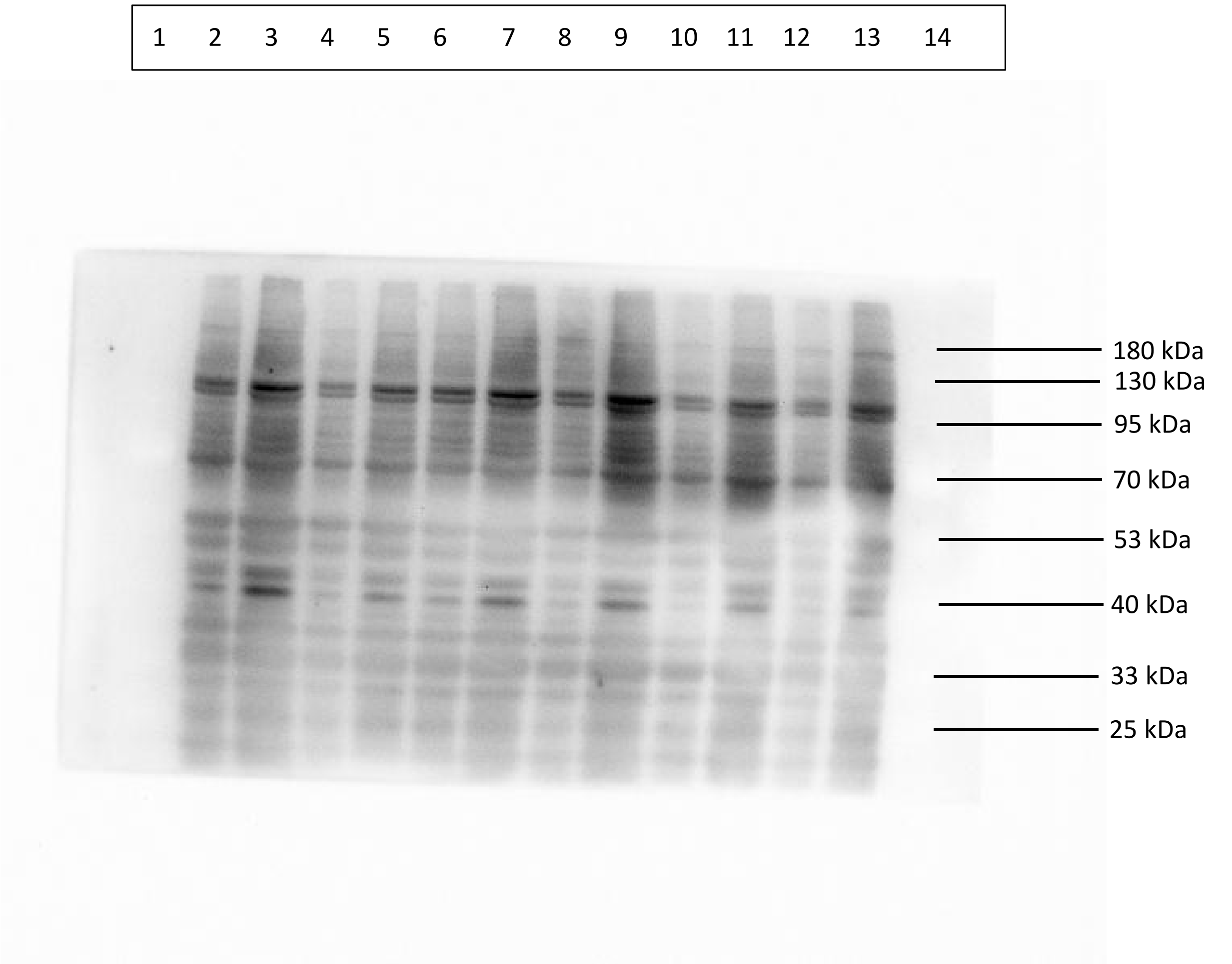

Lane1：Protein marker

Lane2：N

Lane3：T

Lane4：N

Lane5：T

Lane6：N

Lane7：T

Lane8：N

Lane9：T

Lane10：N

Lane11：T

Lane12：N

Lane13：T

Lane14：Protein marker

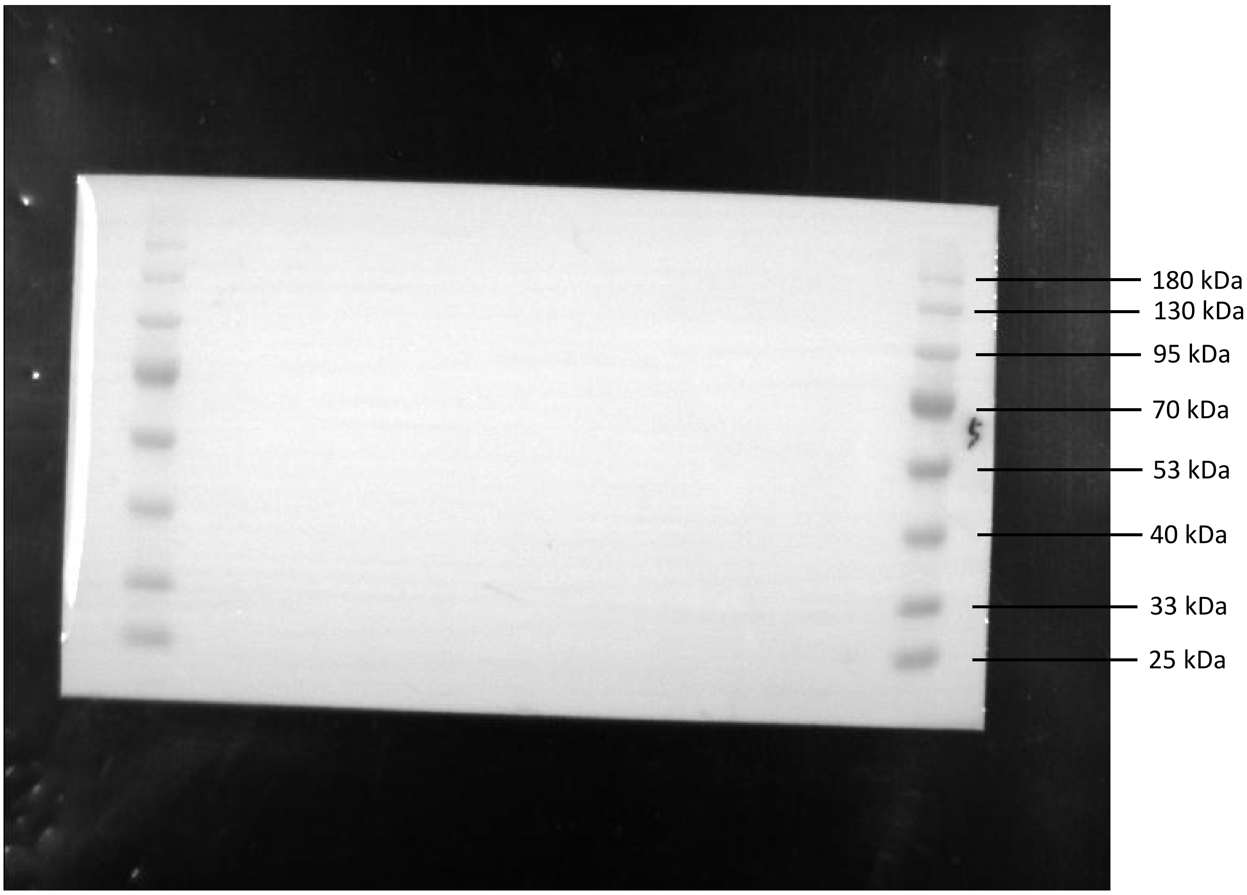

Lane1：Protein marker

Lane2：N

Lane3：T

Lane4：N

Lane5：T

Lane6：N

Lane7：T

Lane8：N

Lane9：T

Lane10：N

Lane11：T

Lane12：N

Lane13：T

Lane14：Protein marker

Figure 3C

ADAMTS8

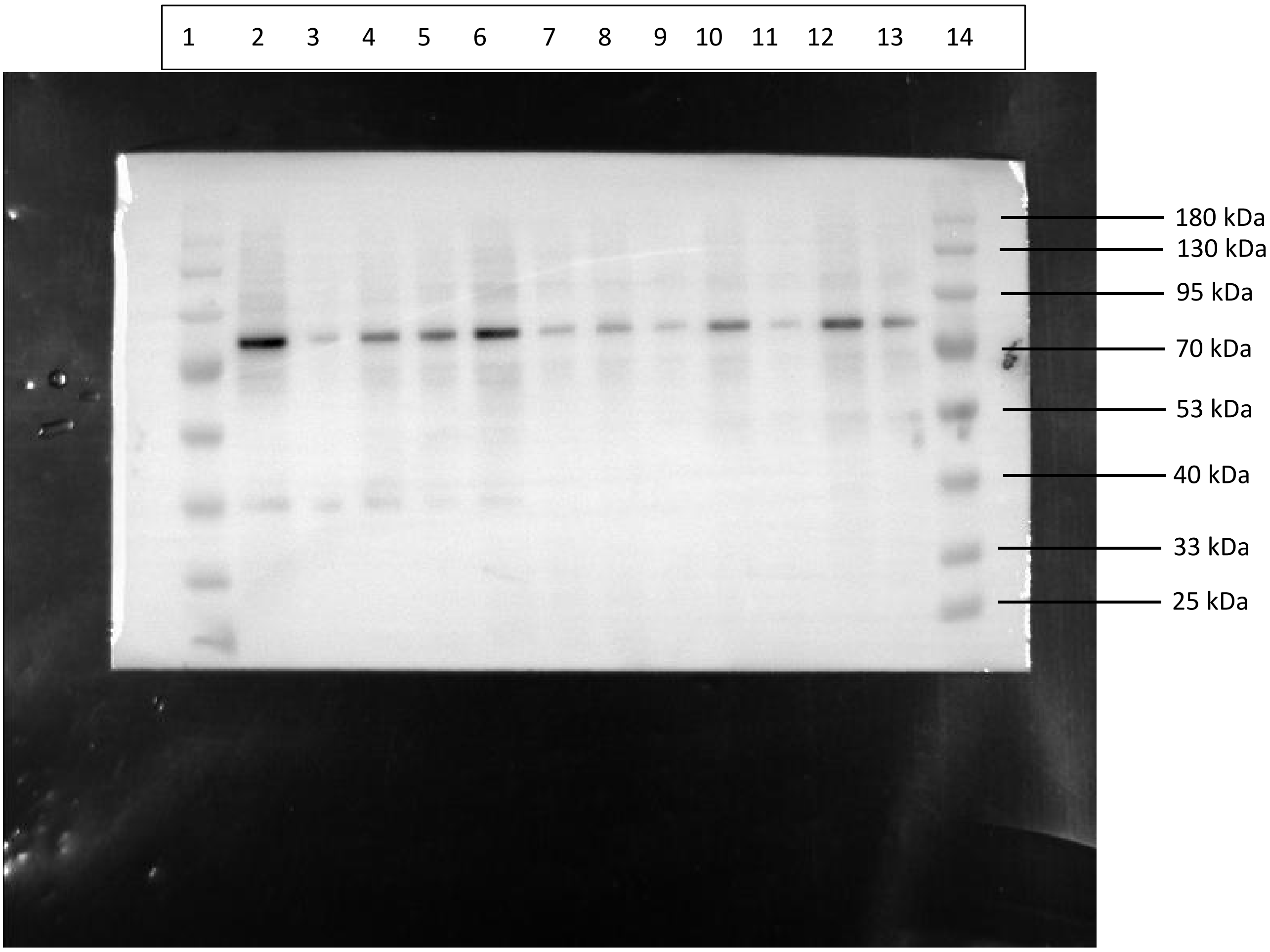

Lane1：Protein marker

Lane2：N

Lane3：T

Lane4：N

Lane5：T

Lane6：N

Lane7：T

Lane8：N

Lane9：T

Lane10：N

Lane11：T

Lane12：N

Lane13：T

Lane14：Protein marker

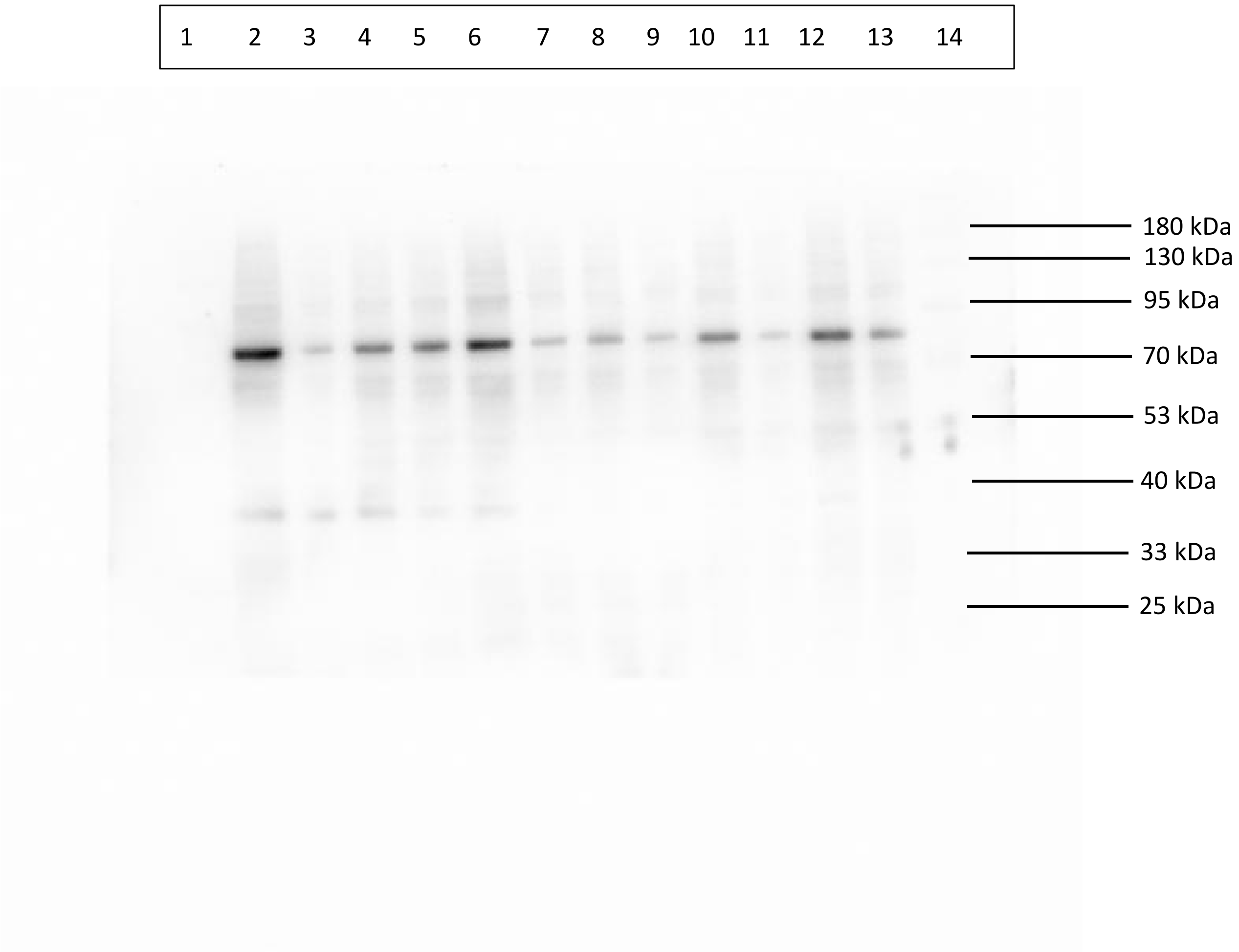

Lane1： Protein marker

Lane2： N

Lane3： T

Lane4： N

Lane5： T

Lane6： N

Lane7： T

Lane8： N

Lane9： T

Lane10： N

Lane11： T

Lane12： N

Lane13： T

Lane14： Protein marker

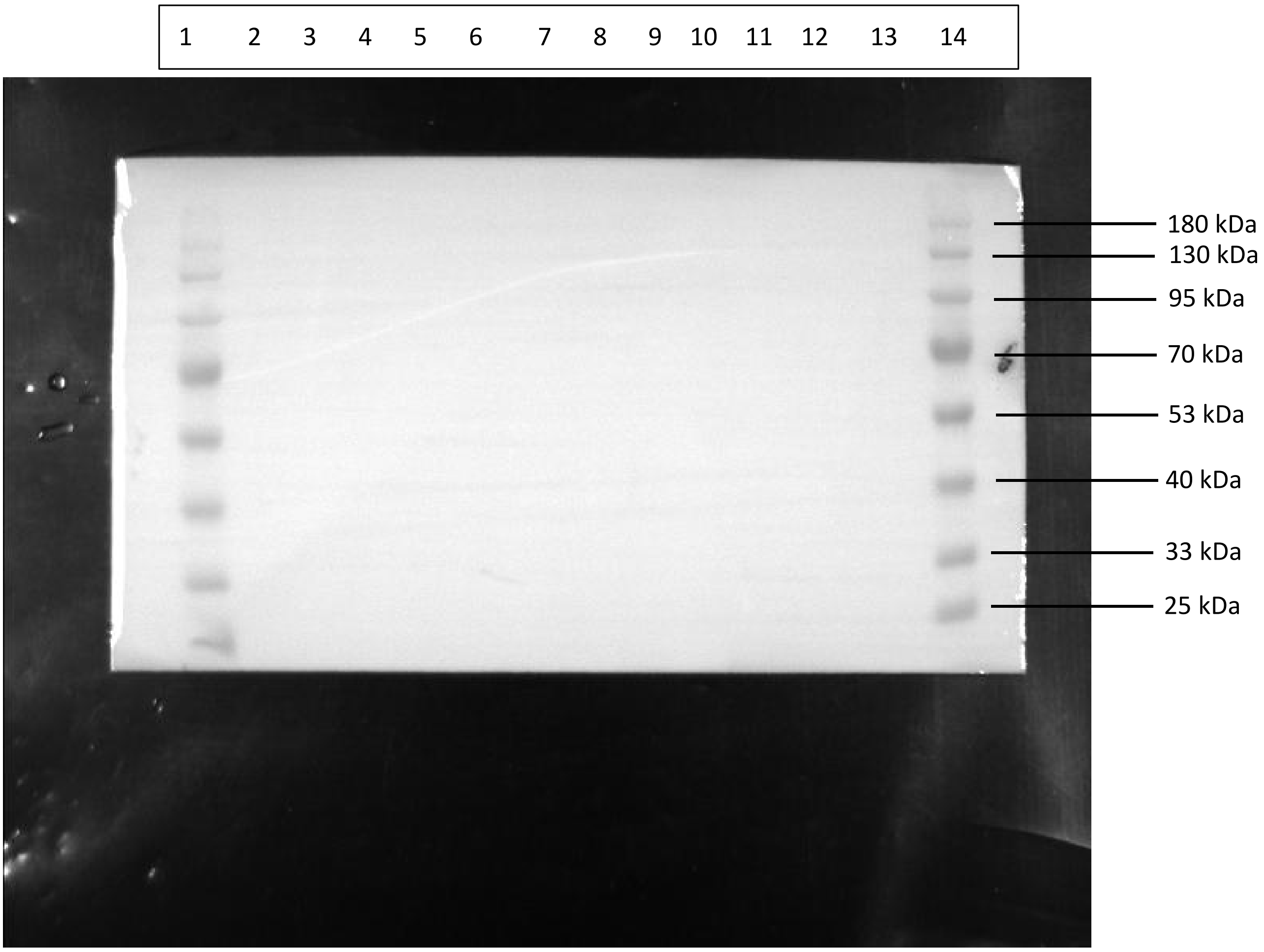

Lane1：Protein marker

Lane2：N

Lane3：T

Lane4：N

Lane5：T

Lane6：N

Lane7：T

Lane8：N

Lane9：T

Lane10：N

Lane11：T

Lane12：N

Lane13：T

Lane14：Protein marker

Figure 3C

β-actin

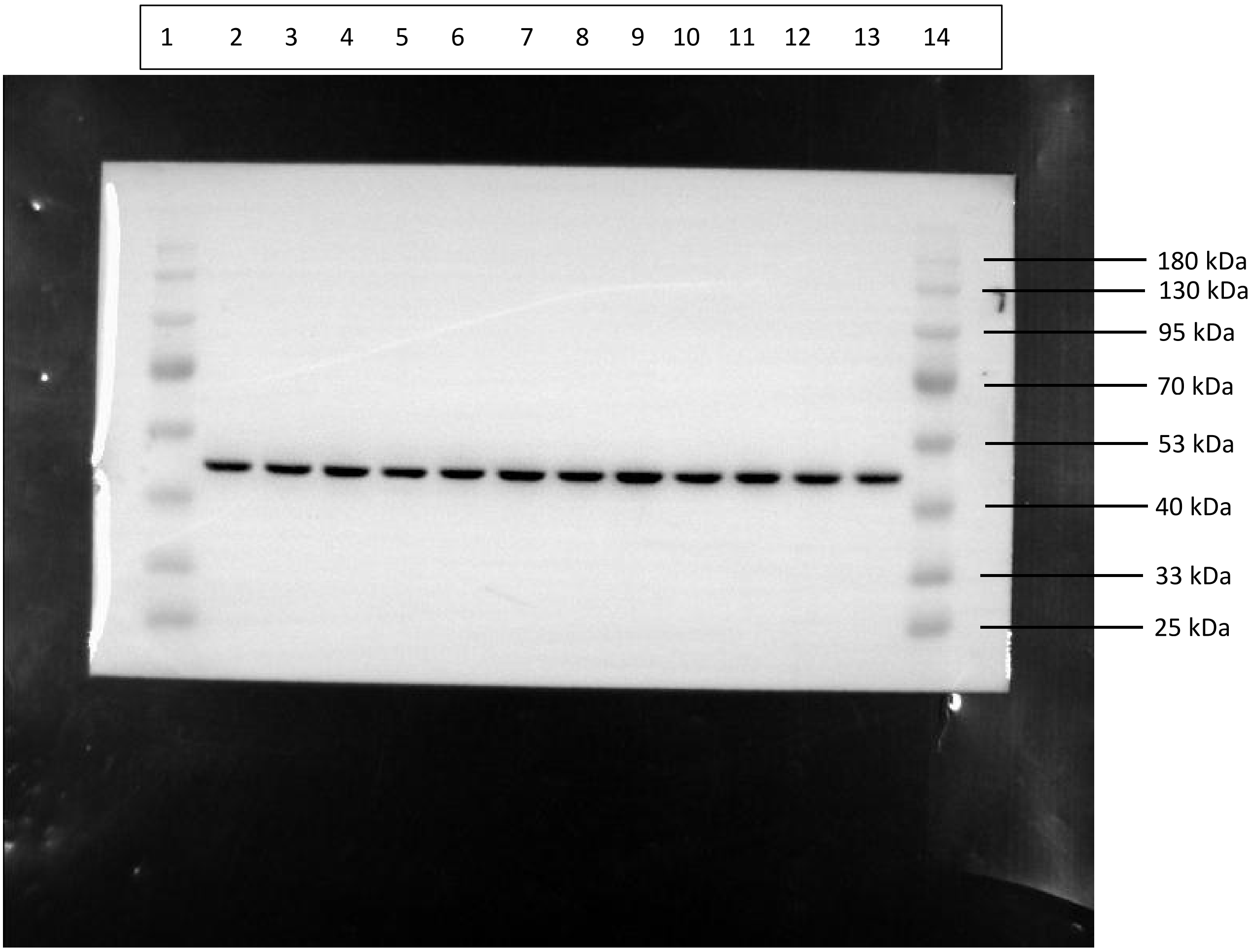

Lane1：Protein marker

Lane2：N

Lane3：T

Lane4：N

Lane5：T

Lane6：N

Lane7：T

Lane8：N

Lane9：T

Lane10：N

Lane11：T

Lane12：N

Lane13：T

Lane14：Protein marker

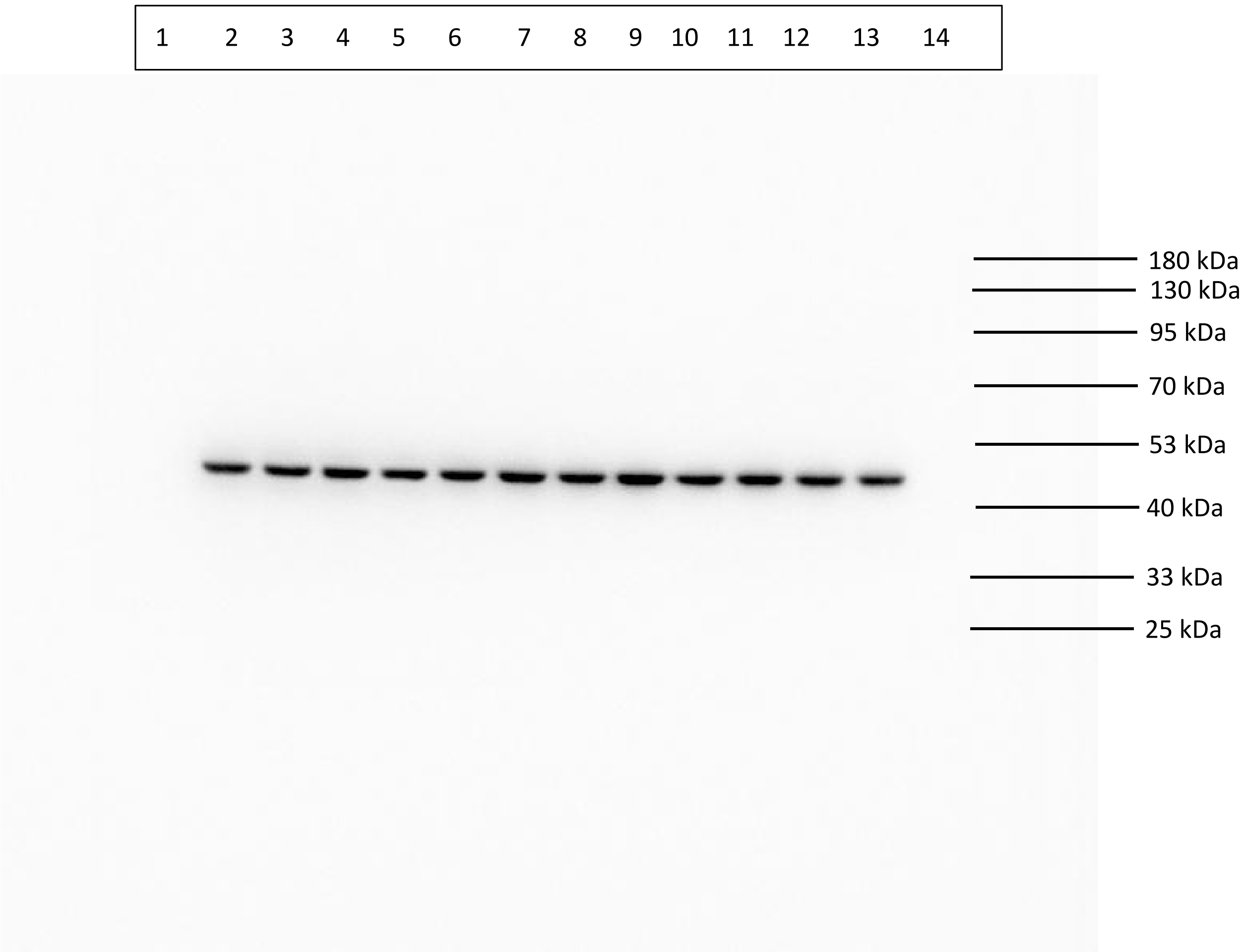

Lane1：Protein marker

Lane2：N

Lane3：T

Lane4：N

Lane5：T

Lane6：N

Lane7：T

Lane8：N

Lane9：T

Lane10：N

Lane11：T

Lane12：N

Lane13：T

Lane14：Protein marker

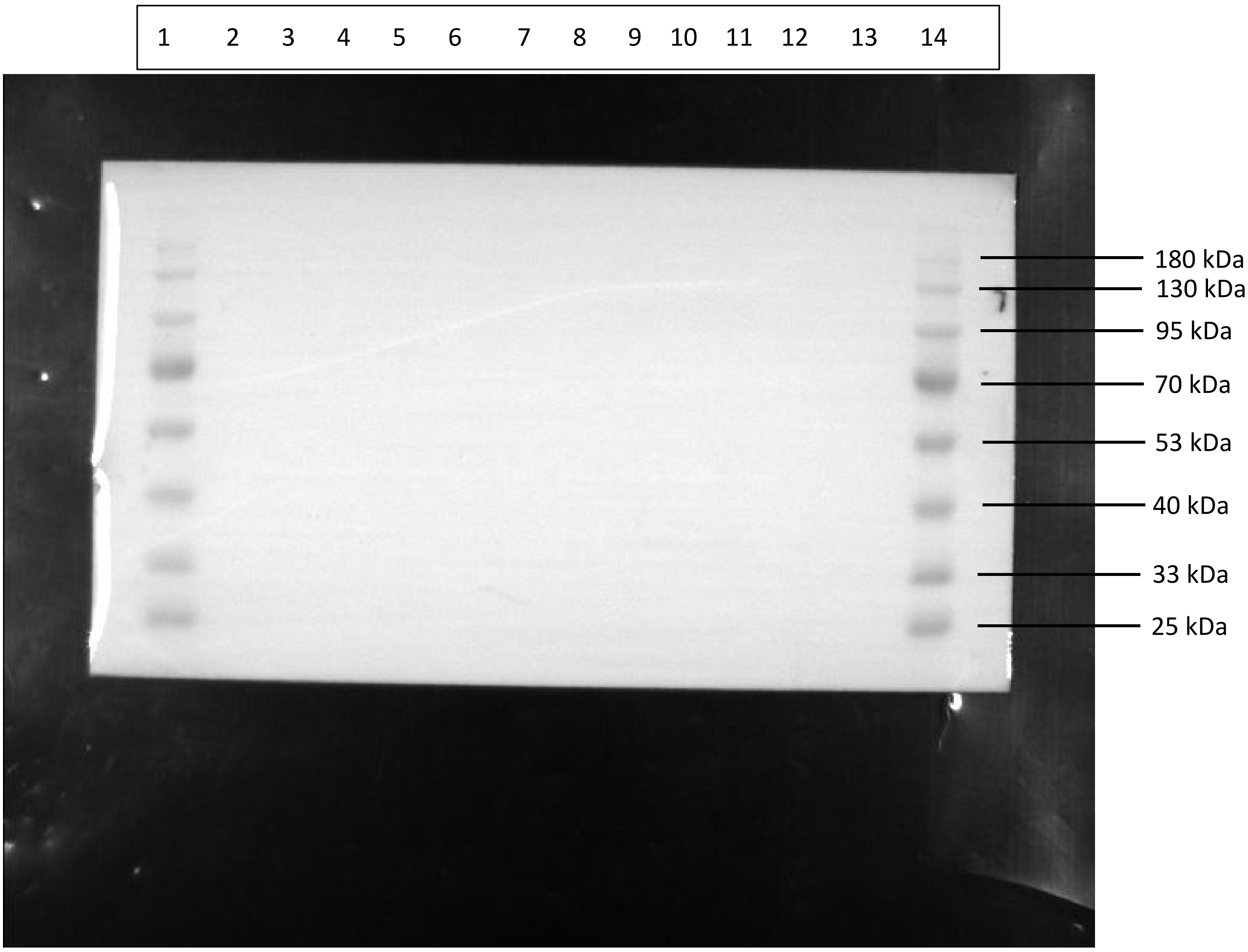

Lane1：Protein marker

Lane2：N

Lane3：T

Lane4：N

Lane5：T

Lane6：N

Lane7：T

Lane8：N

Lane9：T

Lane10：N

Lane11：T

Lane12：N

Lane13：T

Lane14：Protein marker

Figure 3C

DNMT3A

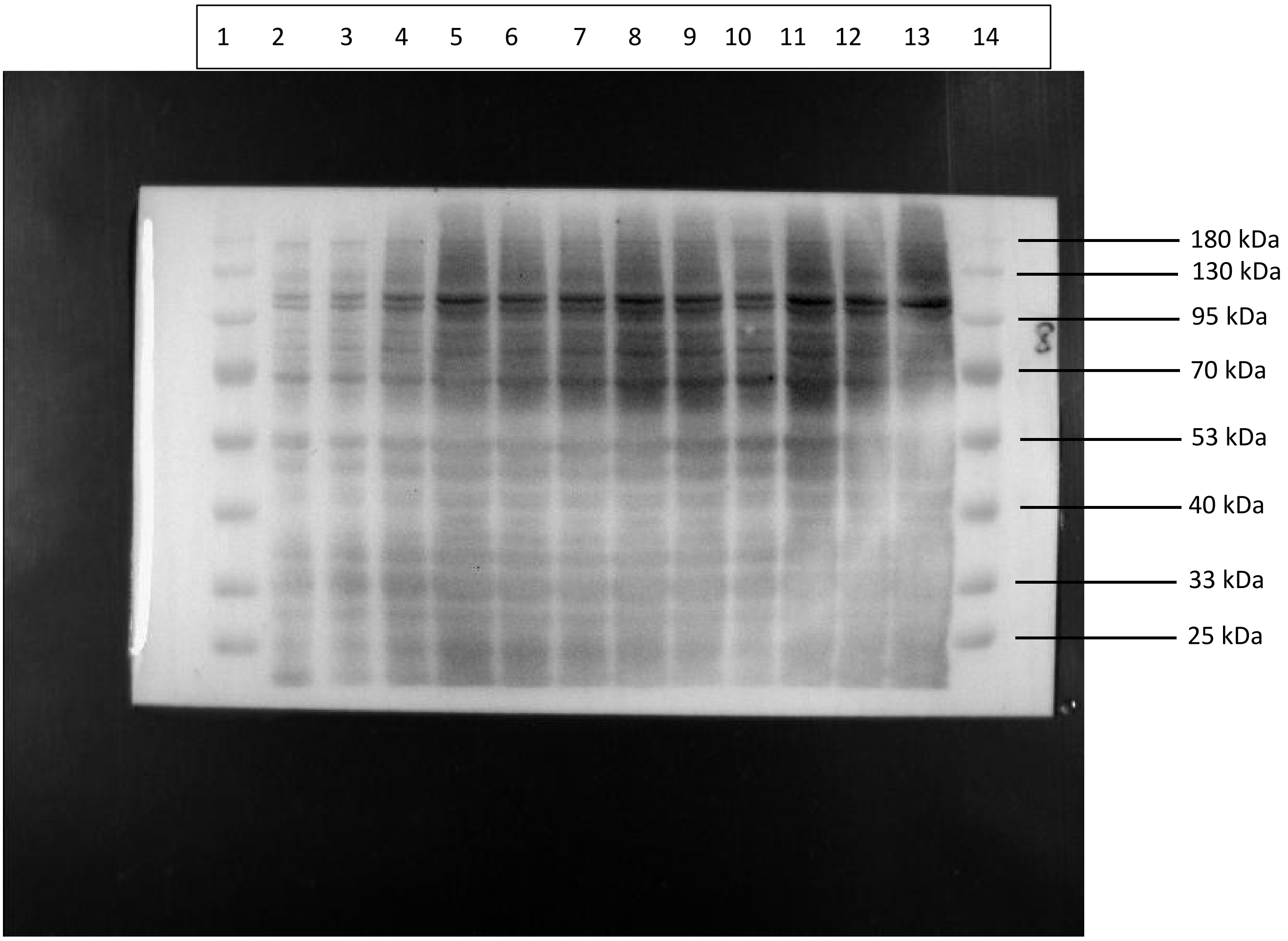

Lane1：Protein marker

Lane2：N

Lane3：T

Lane4：N

Lane5：T

Lane6：N

Lane7：T

Lane8：N

Lane9：T

Lane10：N

Lane11：T

Lane12：N

Lane13：T

Lane14：Protein marker

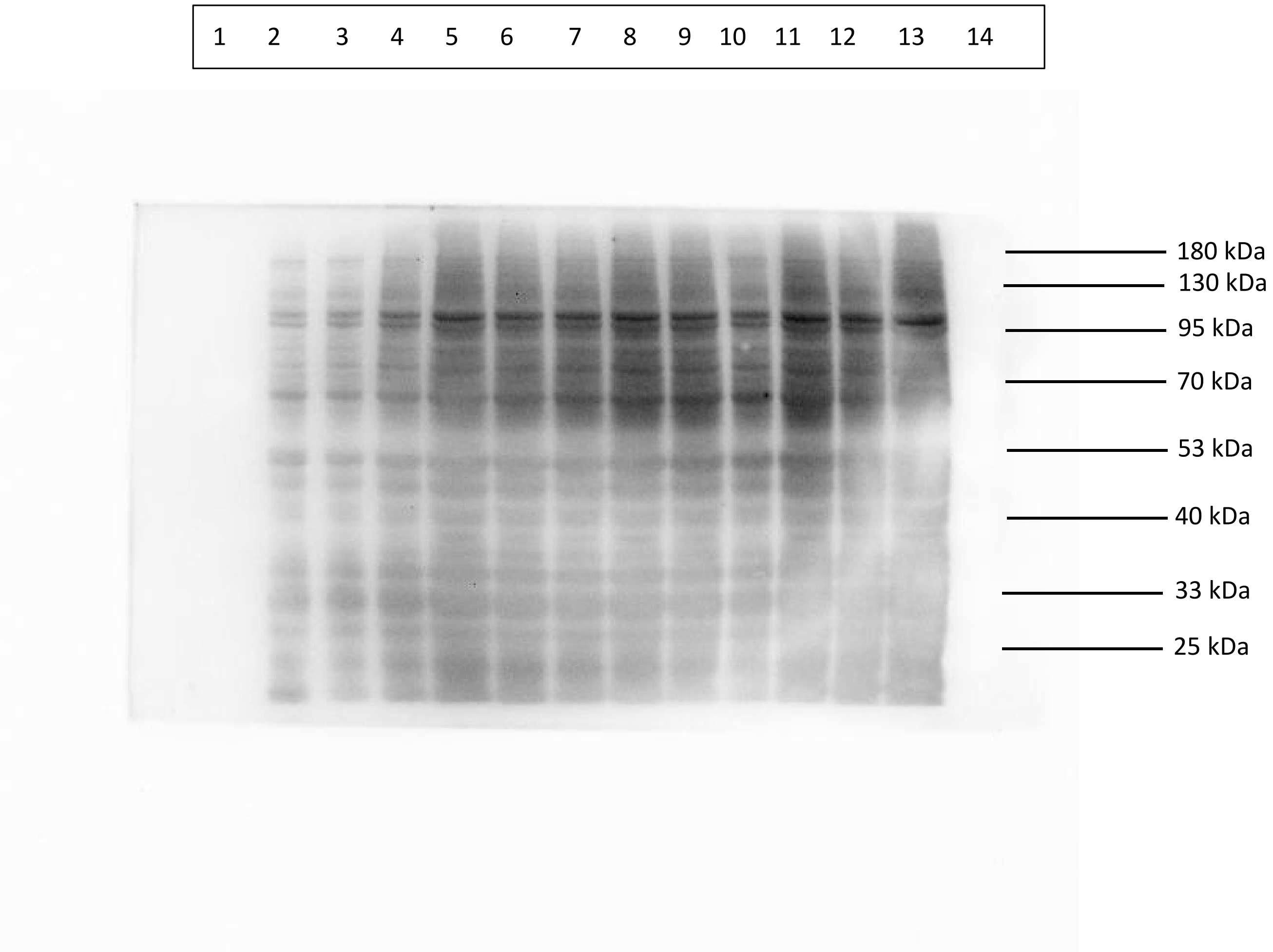

Lane1：Protein marker

Lane2：N

Lane3：T

Lane4：N

Lane5：T

Lane6：N

Lane7：T

Lane8：N

Lane9：T

Lane10：N

Lane11：T

Lane12：N

Lane13：T

Lane14：Protein marker

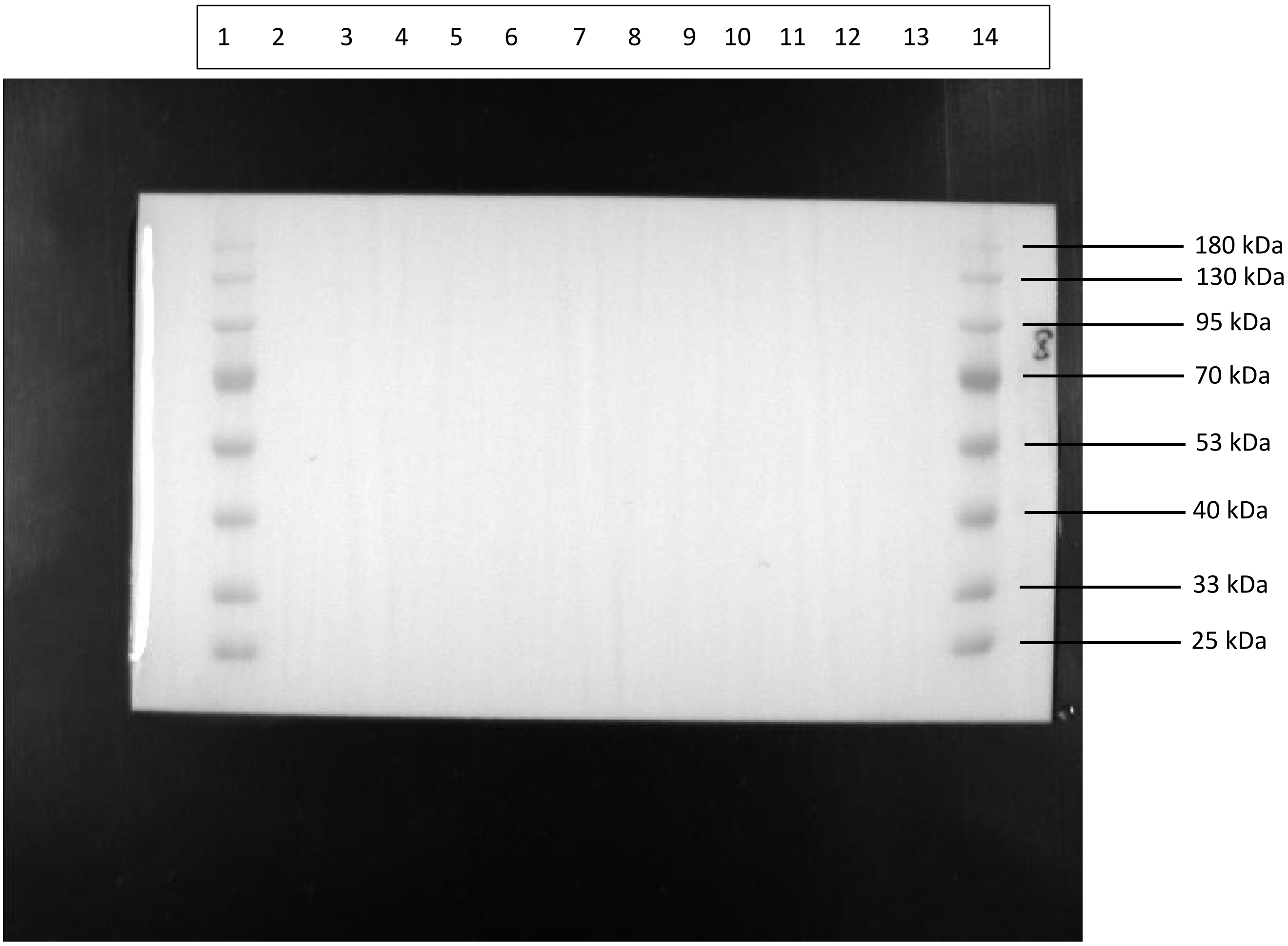

Lane1：Protein marker

Lane2：N

Lane3：T

Lane4：N

Lane5：T

Lane6：N

Lane7：T

Lane8：N

Lane9：T

Lane10：N

Lane11：T

Lane12：N

Lane13：T

Lane14：Protein marker

Figure 3C

ADAMTS8

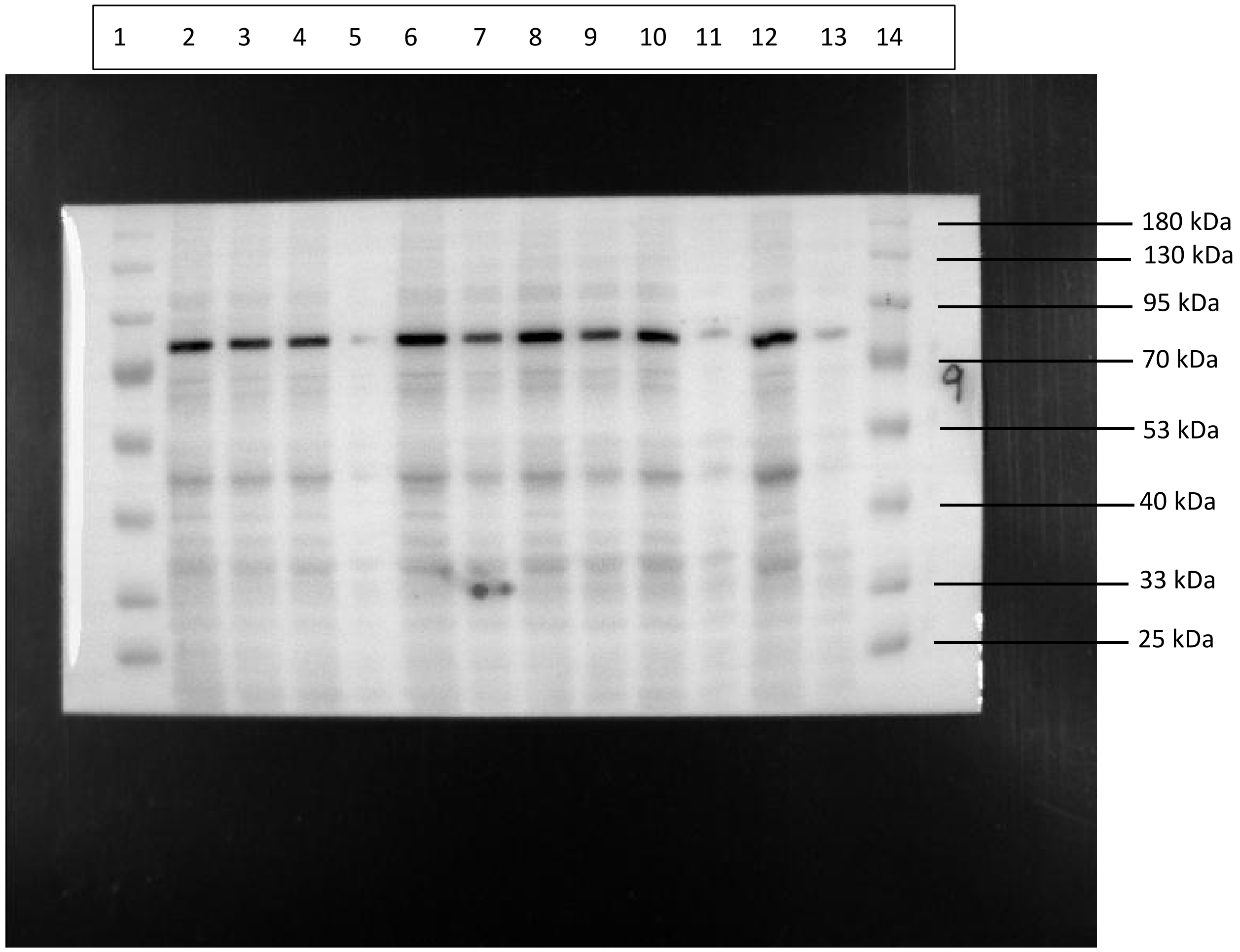

Lane1：Protein marker

Lane2：N

Lane3：T

Lane4：N

Lane5：T

Lane6：N

Lane7：T

Lane8：N

Lane9：T

Lane10：N

Lane11：T

Lane12：N

Lane13：T

Lane14：Protein marker

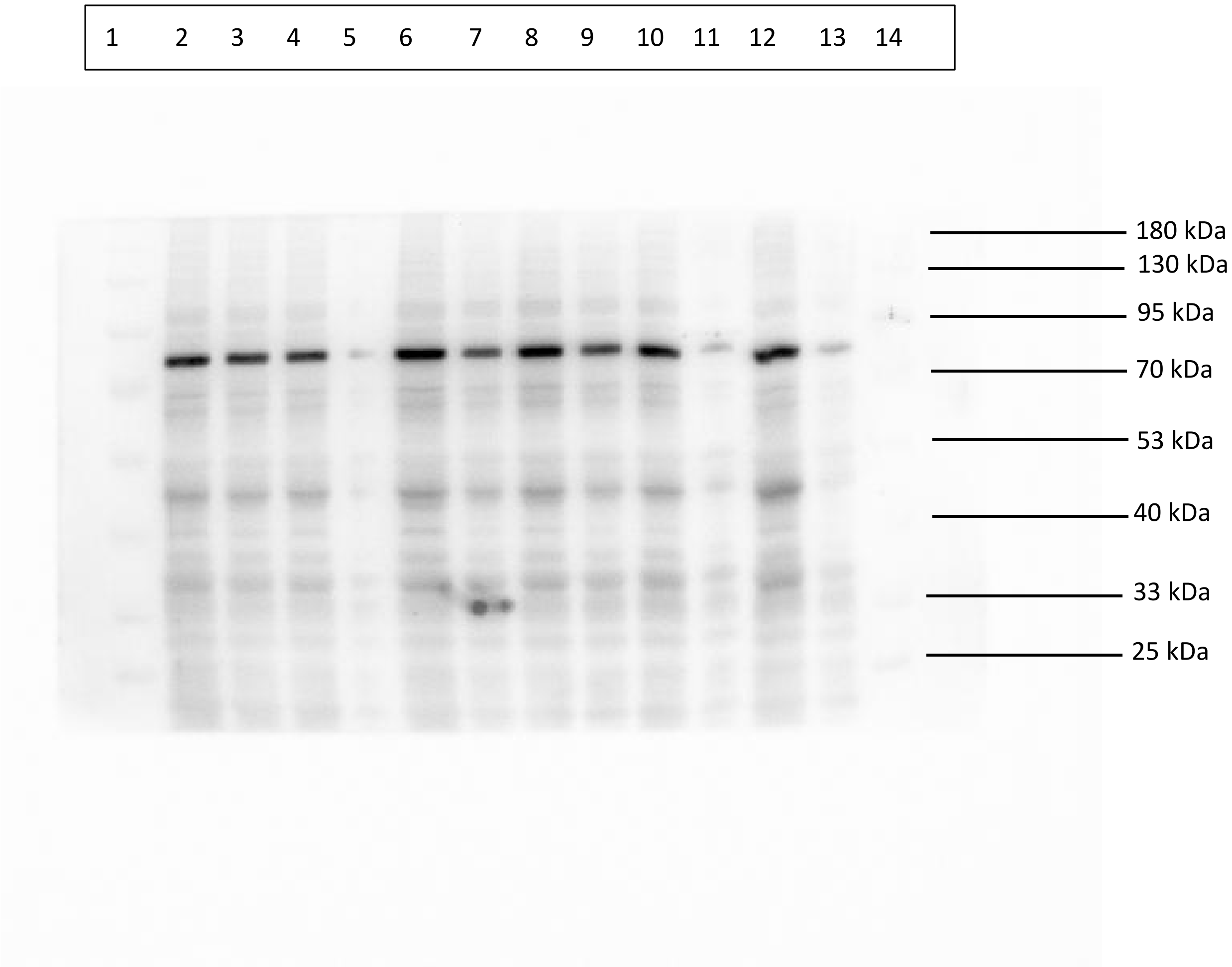

Lane1：Protein marker

Lane2：N

Lane3：T

Lane4：N

Lane5：T

Lane6：N

Lane7：T

Lane8：N

Lane9：T

Lane10：N

Lane11：T

Lane12：N

Lane13：T

Lane14：Protein marker

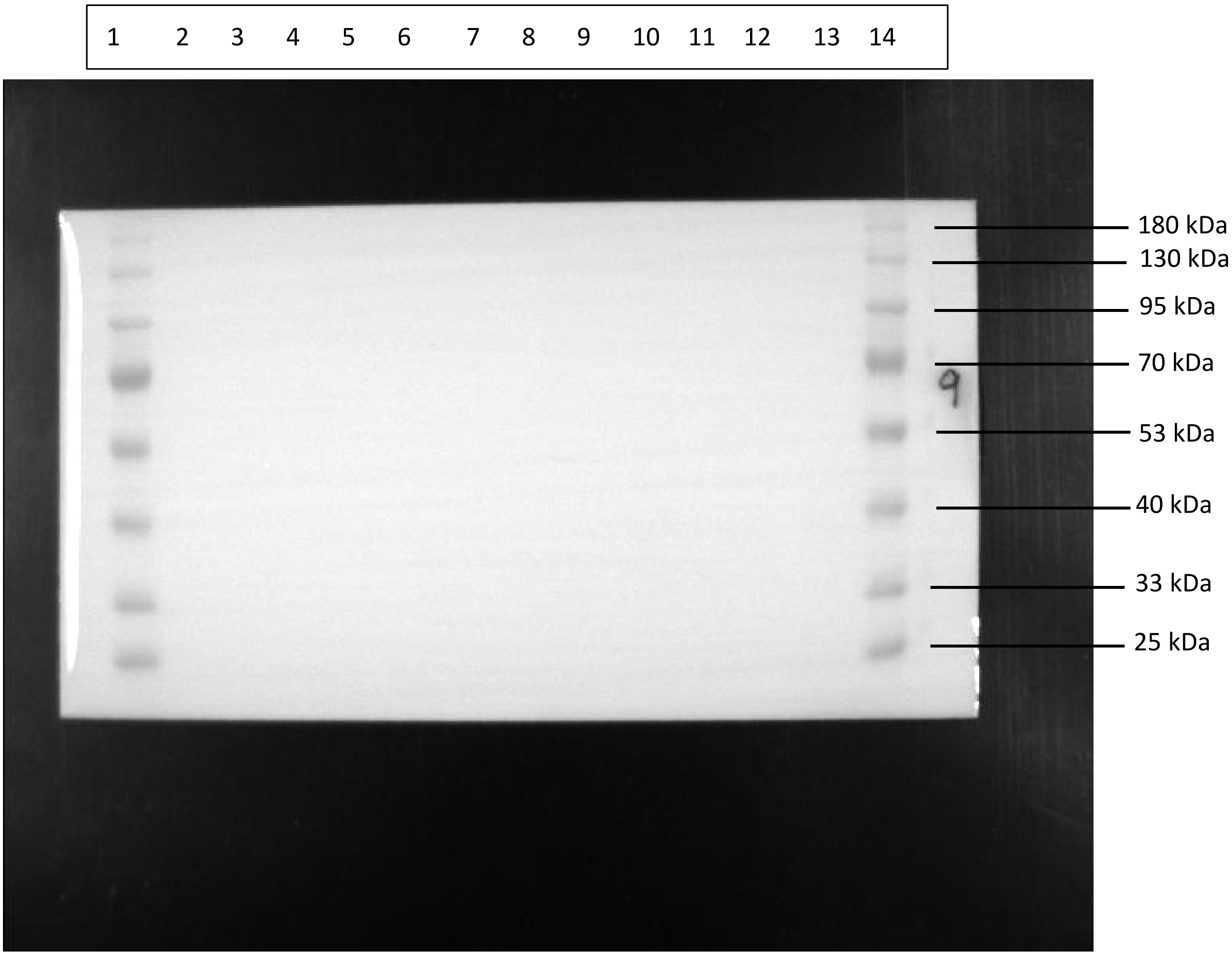

Lane1：Protein marker

Lane2：N

Lane3：T

Lane4：N

Lane5：T

Lane6：N

Lane7：T

Lane8：N

Lane9：T

Lane10：N

Lane11：T

Lane12：N

Lane13：T

Lane14：Protein marker

Figure 3C

β-actin

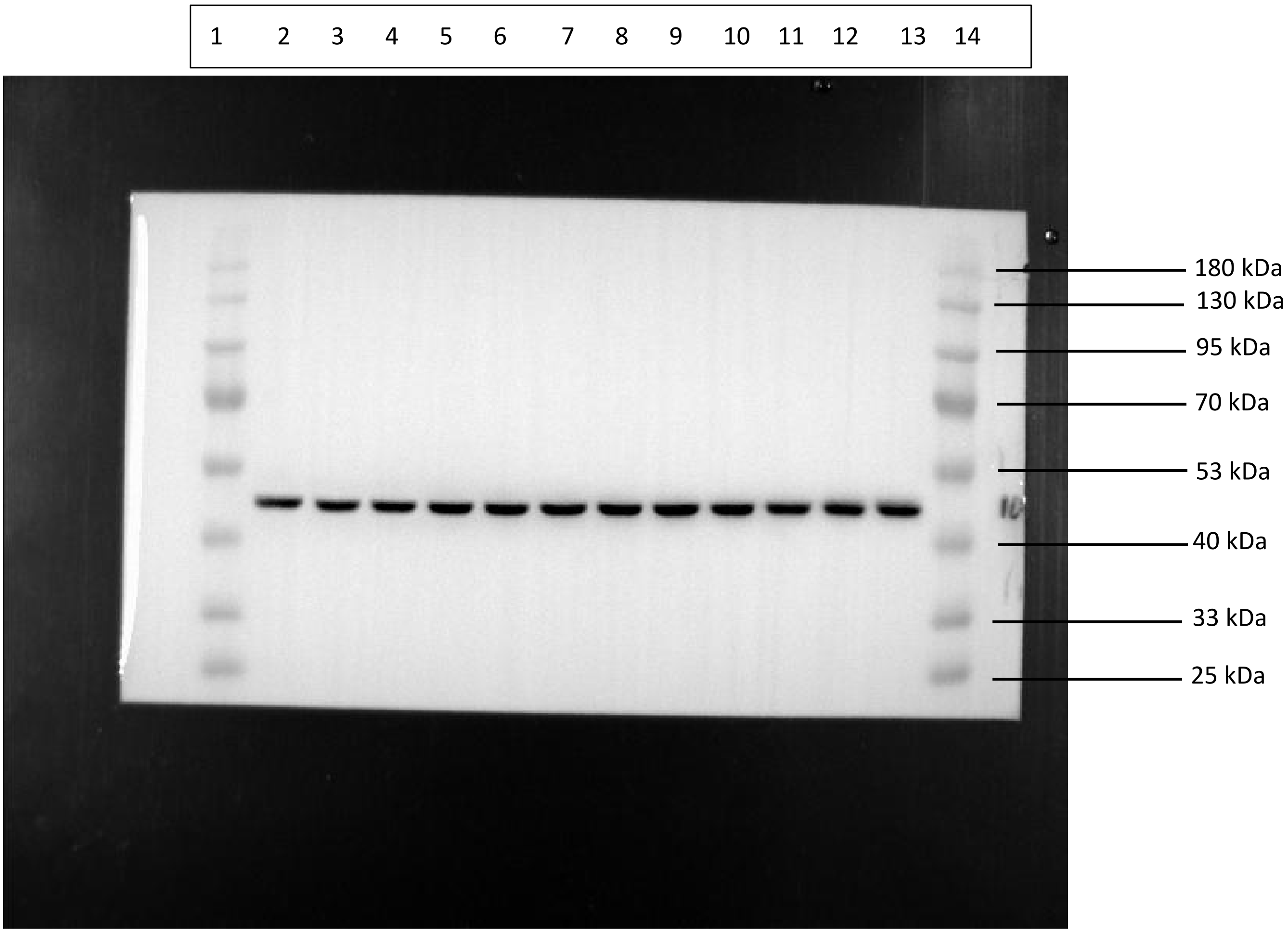

Lane1：Protein marker

Lane2：N

Lane3：T

Lane4：N

Lane5：T

Lane6：N

Lane7：T

Lane8：N

Lane9：T

Lane10：N

Lane11：T

Lane12：N

Lane13：T

Lane14：Protein marker

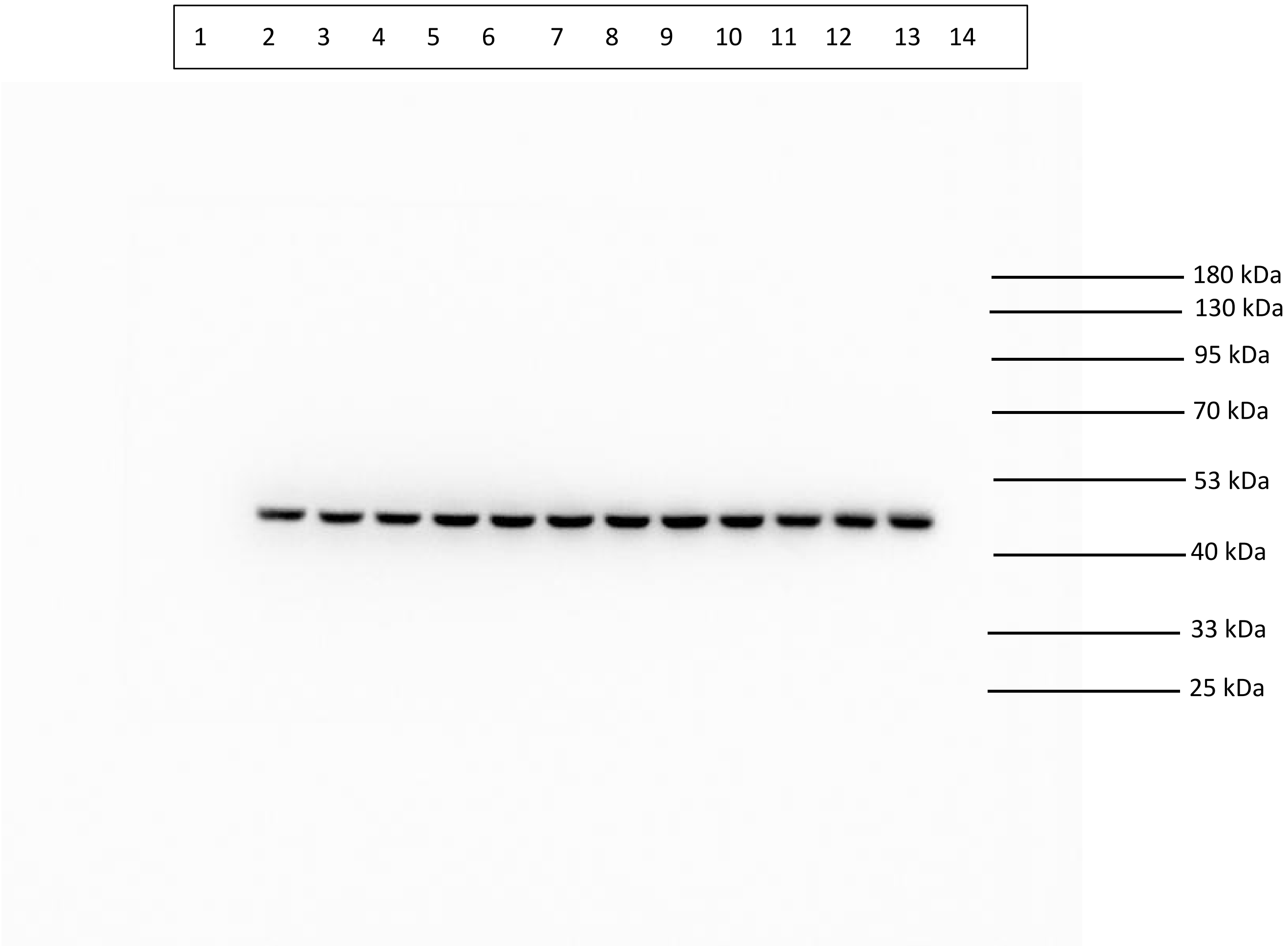

Lane1：Protein marker

Lane2：N

Lane3：T

Lane4：N

Lane5：T

Lane6：N

Lane7：T

Lane8：N

Lane9：T

Lane10：N

Lane11：T

Lane12：N

Lane13：T

Lane14：Protein marker

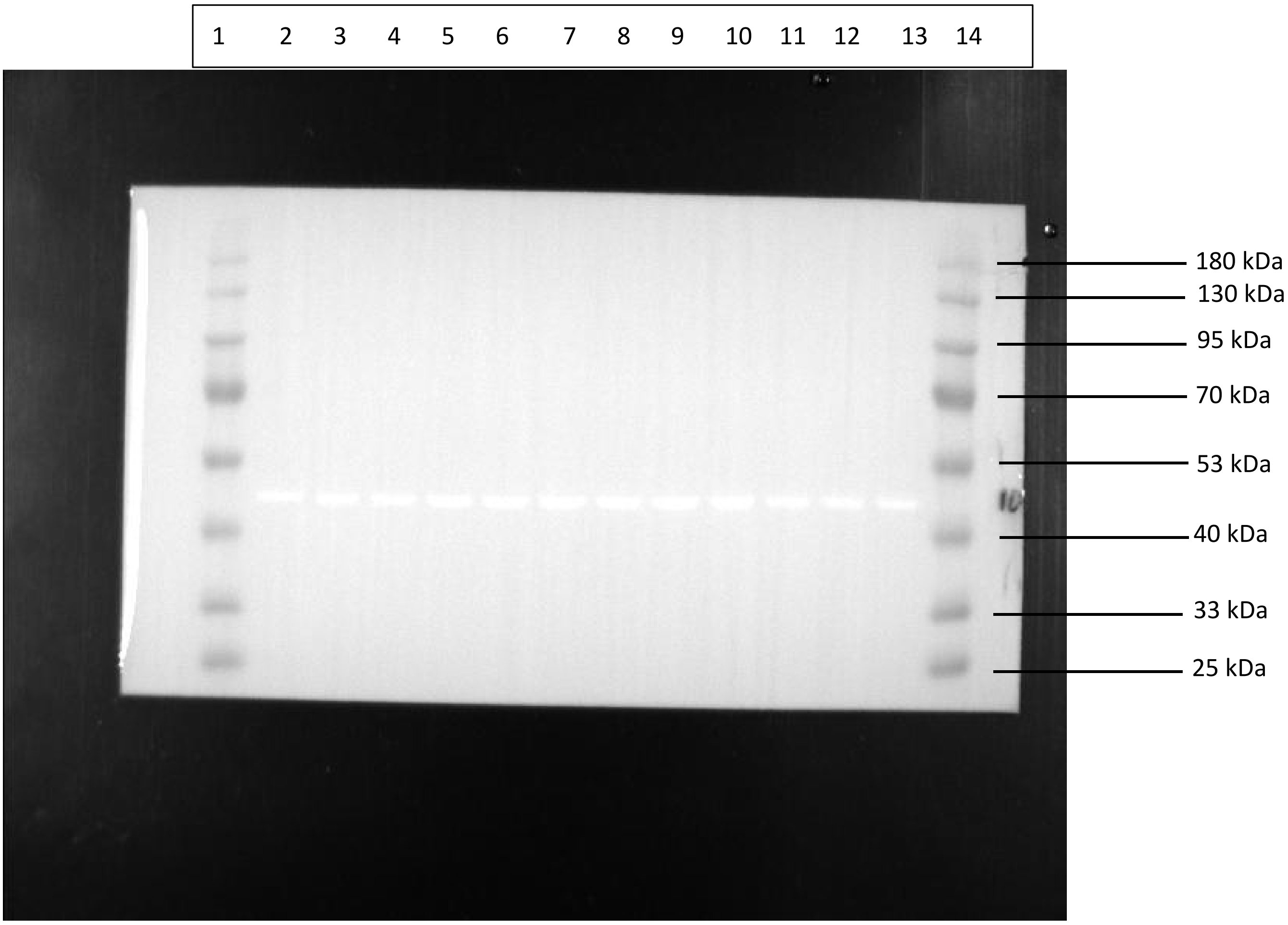

Lane1：Protein marker

Lane2：N

Lane3：T

Lane4：N

Lane5：T

Lane6：N

Lane7：T

Lane8：N

Lane9：T

Lane10：N

Lane11：T

Lane12：N

Lane13：T

Lane14：Protein marker

Figure 4B

DNMT3A

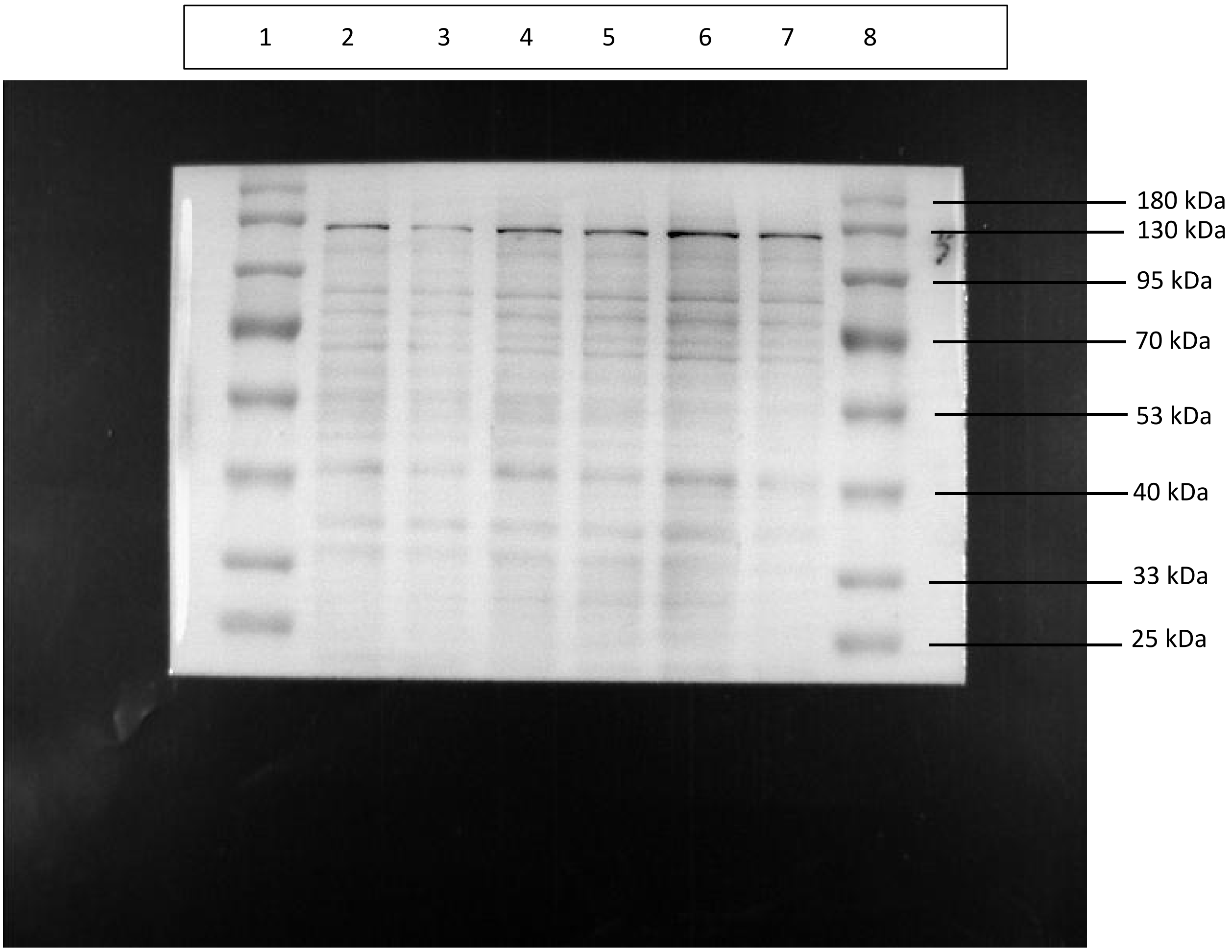

Lane1：Protein marker

Lane2：MDA-MB-157

Lane3：MDA-MB-231

Lane4：MDA-MB-453

Lane5：MDA-MB-468

Lane6：MCF-7

Lane7：T47D

Lane8：Protein marker

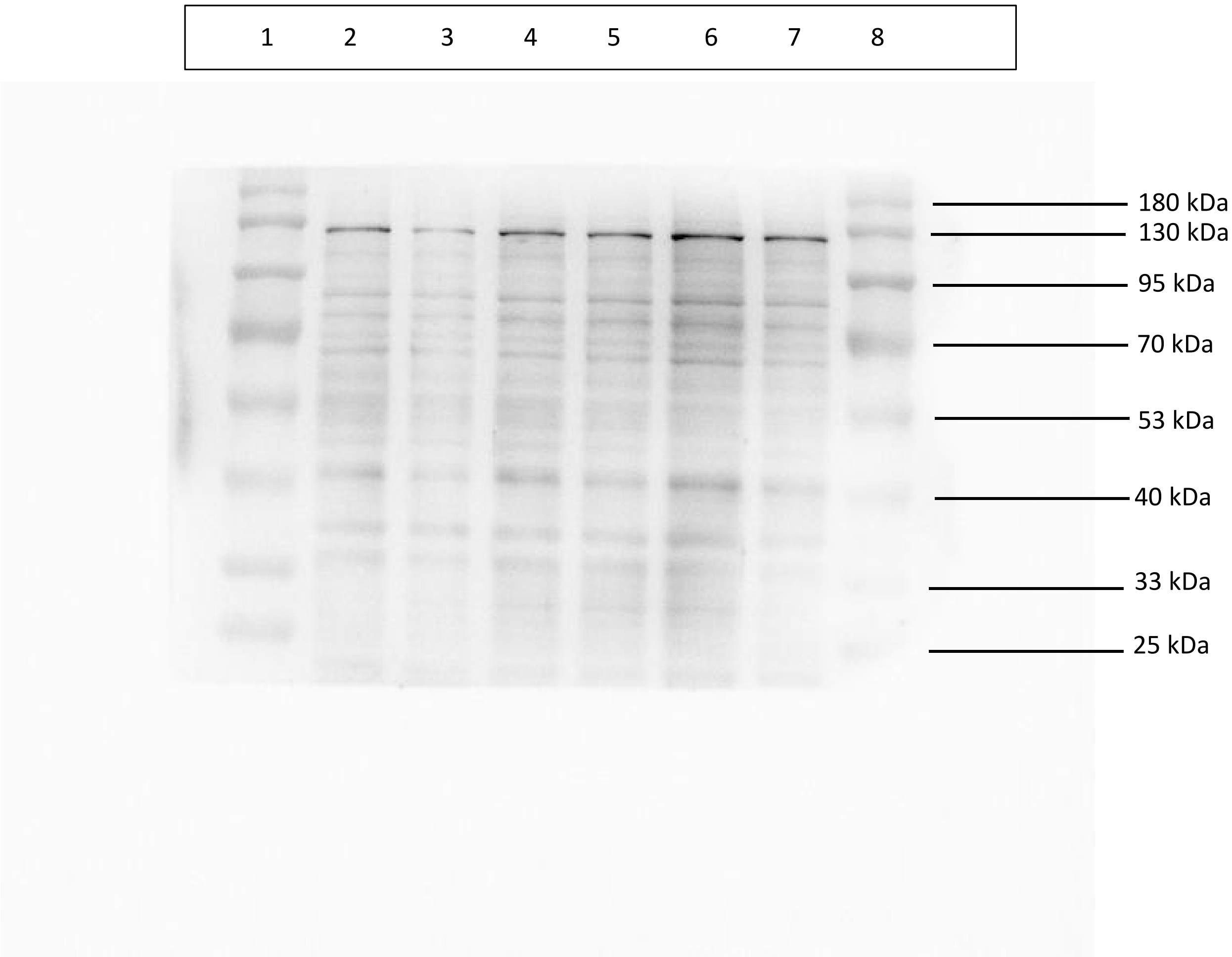

Lane1：Protein marker
Lane2：MDA-MB-157
Lane3：MDA-MB-231
Lane4：MDA-MB-453
Lane5：MDA-MB-468
Lane6：MCF-7
Lane7：T47D
Lane8：Protein marker

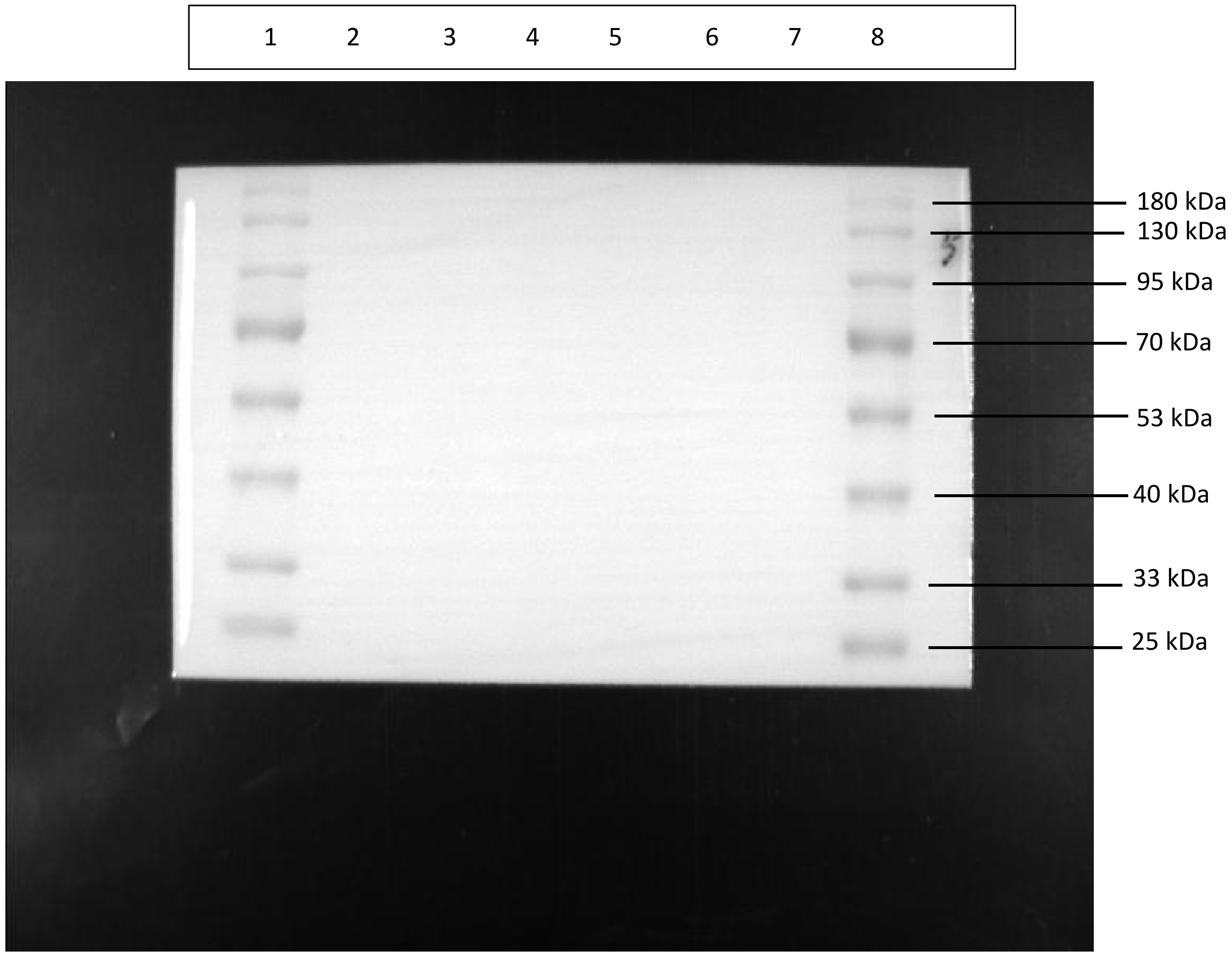

Lane1：Protein marker

Lane2：MDA-MB-157

Lane3：MDA-MB-231

Lane4：MDA-MB-453

Lane5：MDA-MB-468

Lane6：MCF-7

Lane7：T47D

Lane8：Protein marker

Figure 4B

β-actin

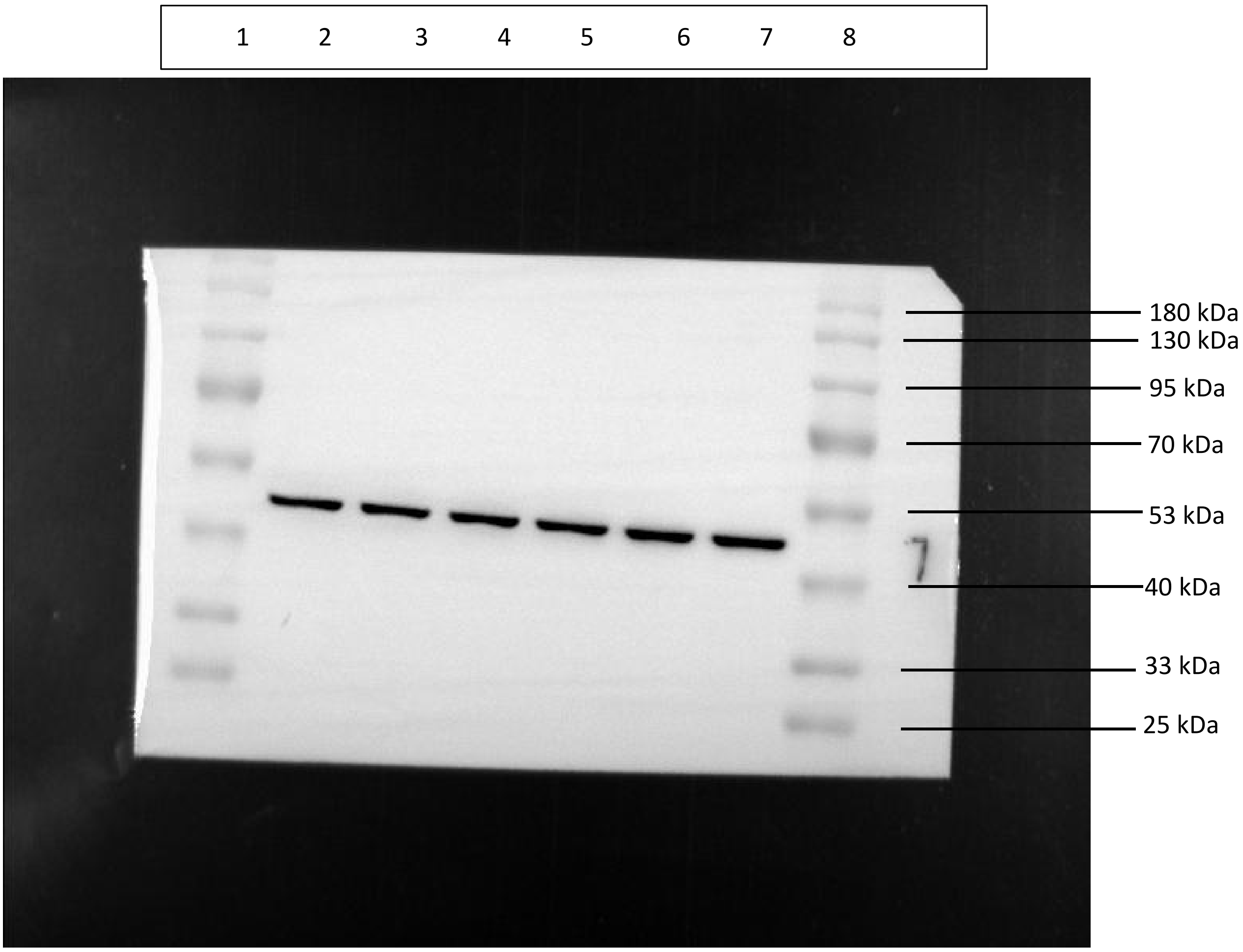

Lane1： Protein marker

Lane2： MDA-MB-157

Lane3： MDA-MB-231

Lane4： MDA-MB-453

Lane5： MDA-MB-468

Lane6： MCF-7

Lane7： T47D

Lane8： Protein marker

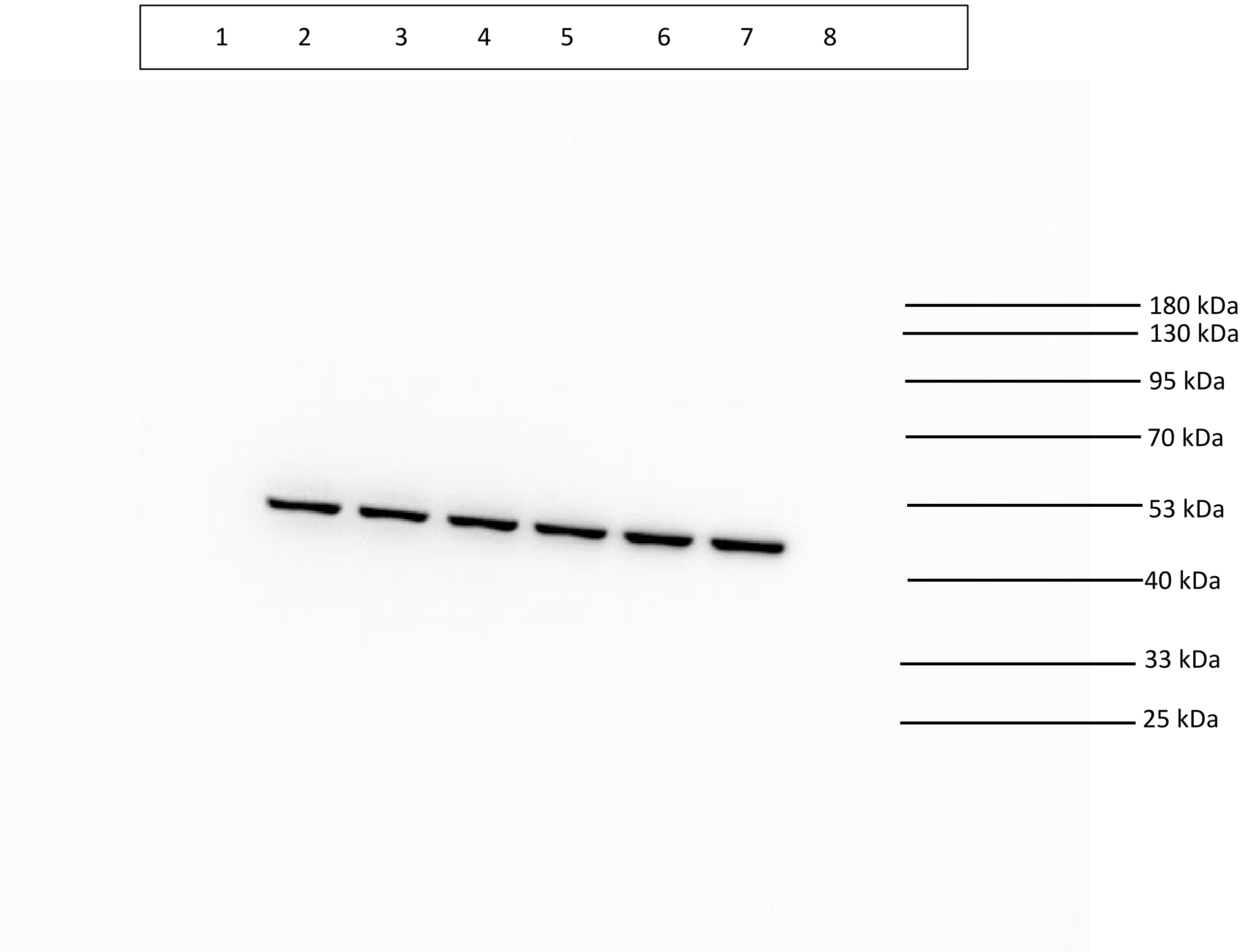

Lane1：Protein marker

Lane2：MDA-MB-157

Lane3：MDA-MB-231

Lane4：MDA-MB-453

Lane5：MDA-MB-468

Lane6：MCF-7

Lane7：T47D

Lane8：Protein marker

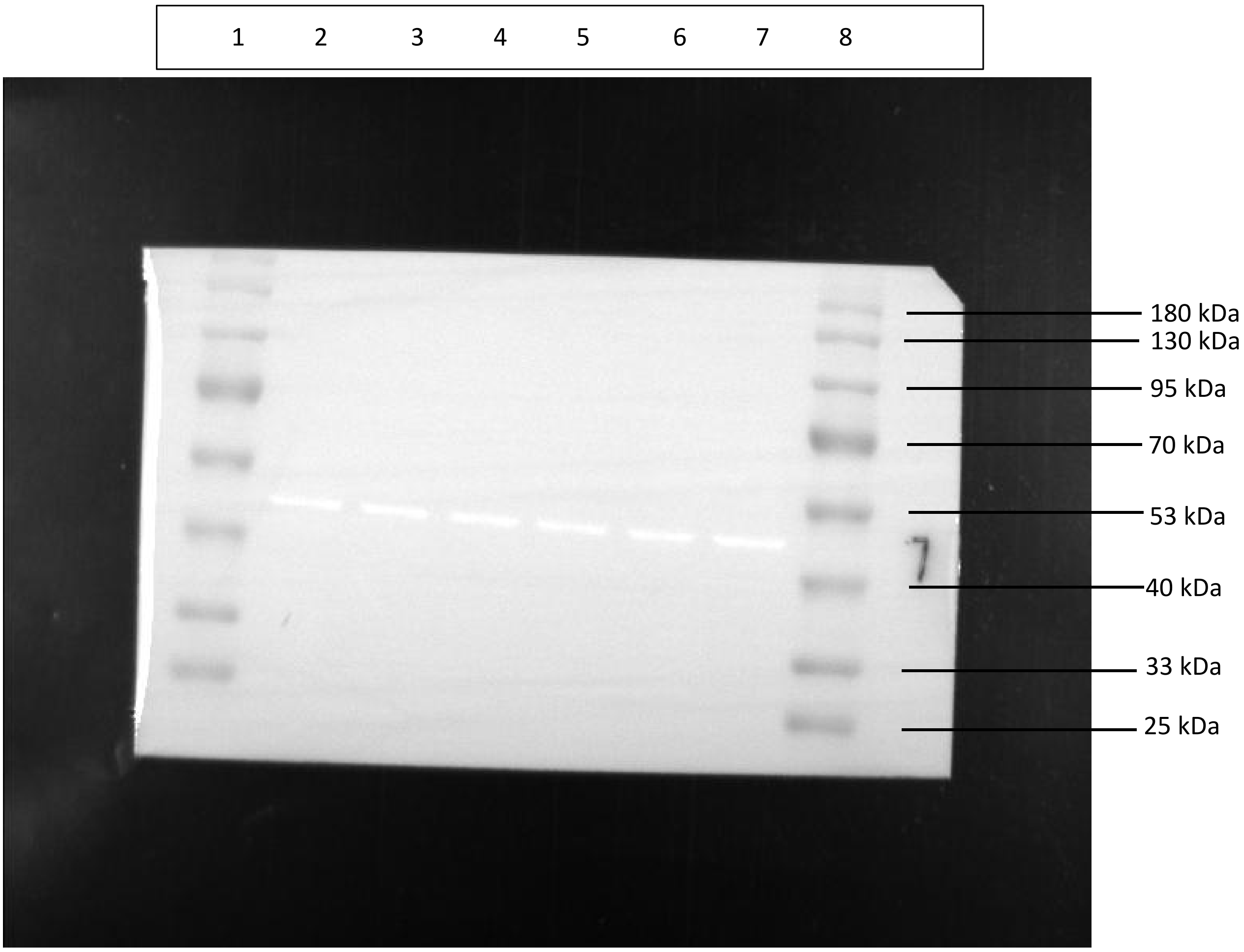

Lane1：Protein marker

Lane2：MDA-MB-157

Lane3：MDA-MB-231

Lane4：MDA-MB-453

Lane5：MDA-MB-468

Lane6：MCF-7

Lane7：T47D

Lane8：Protein marker

Figure 4D

DNMT3A

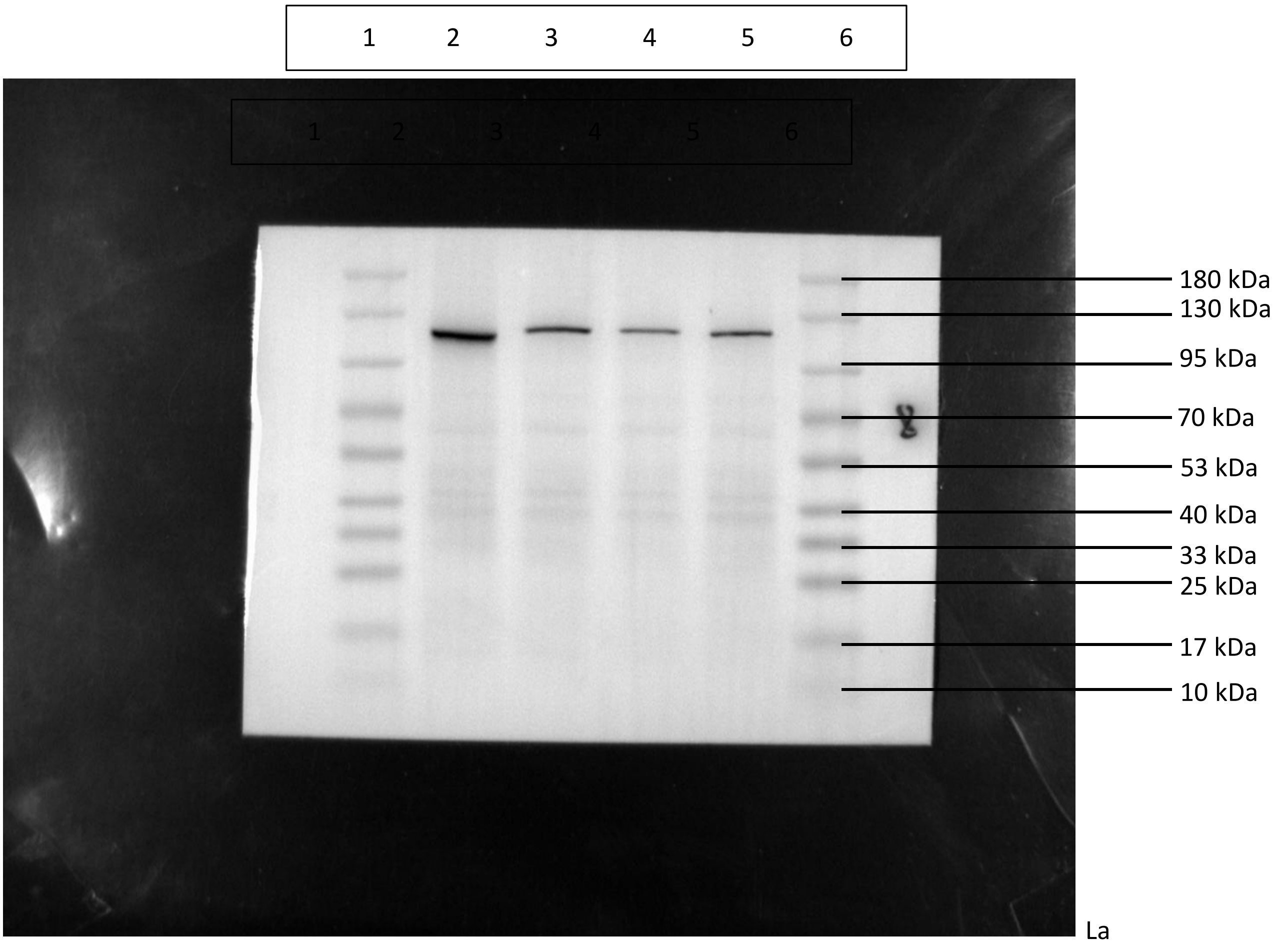

Lane1：Protein marker

Lane2：NC

Lane3：si-DNMT3A-1

Lane4：si-DNMT3A-2

Lane5：si-DNMT3A-3

Lane6：Protein marker

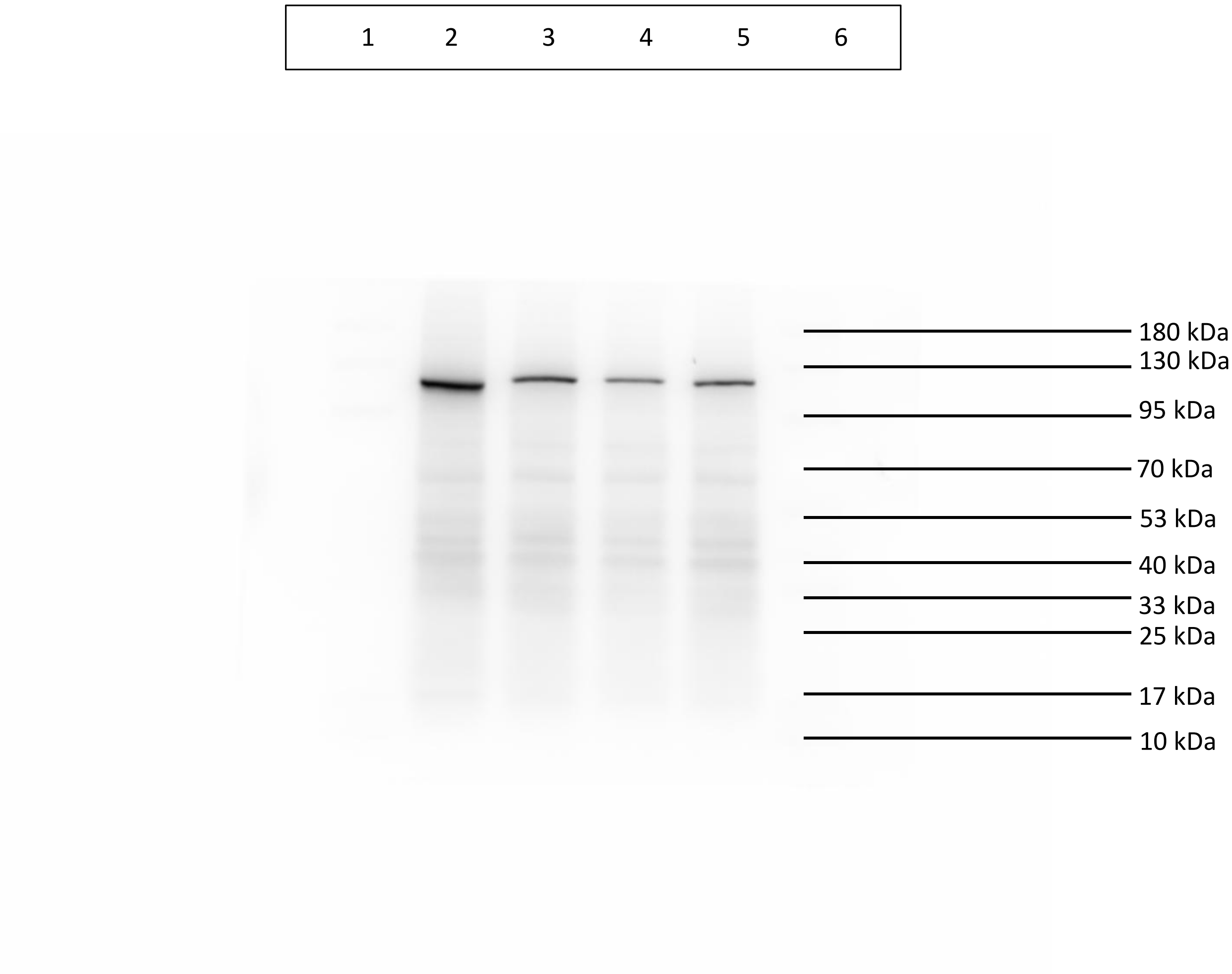

Lane1：Protein marker

Lane2：NC

Lane3：si-DNMT3A-1

Lane4：si-DNMT3A-2

Lane5：si-DNMT3A-3

Lane6：Protein marker

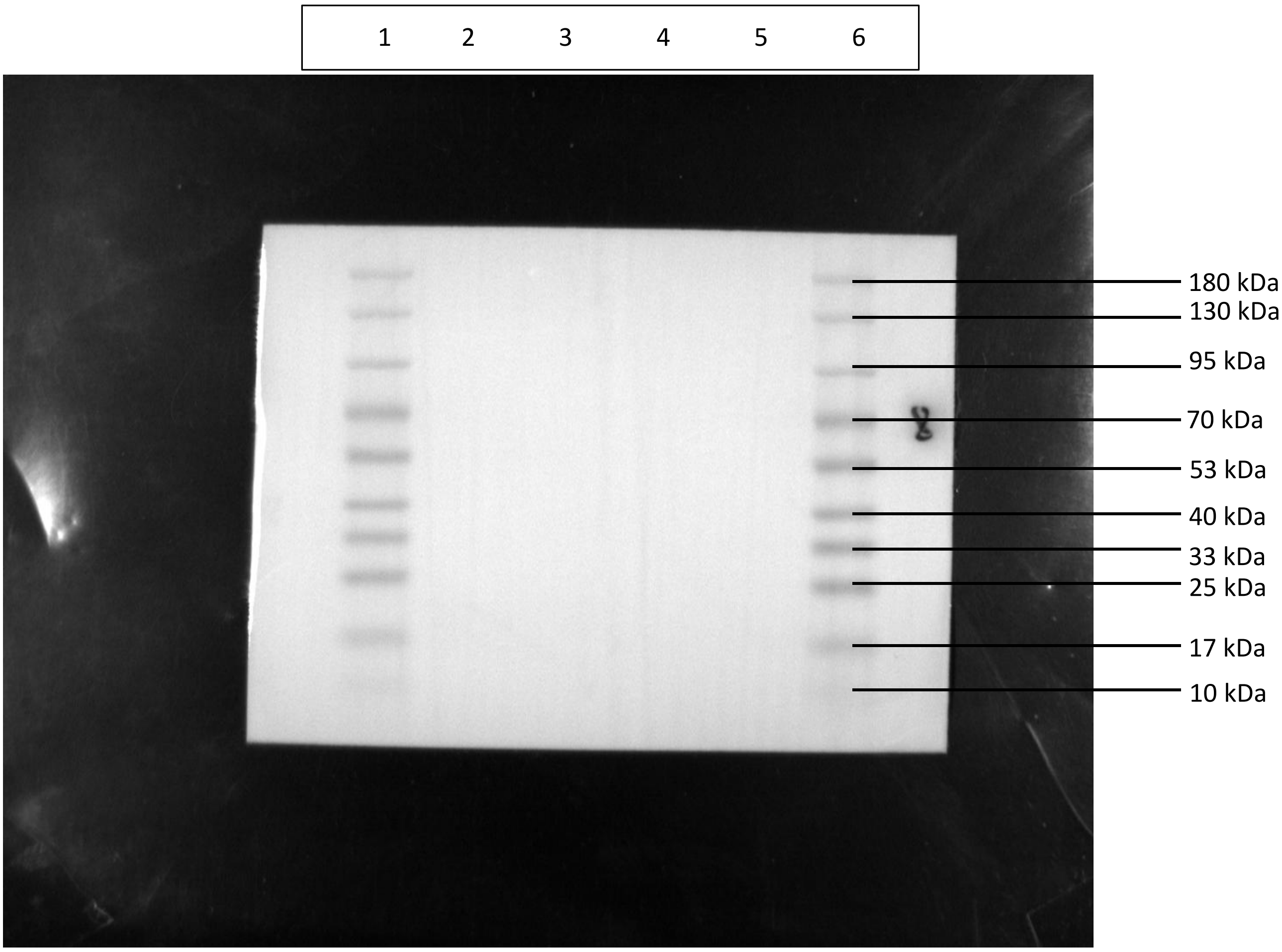

Lane1：Protein marker

Lane2：NC

Lane3：si-DNMT3A-1

Lane4：si-DNMT3A-2

Lane5：si-DNMT3A-3

Lane6：Protein marker

Figure 4D

β-actin

1	2	3	4	5	6

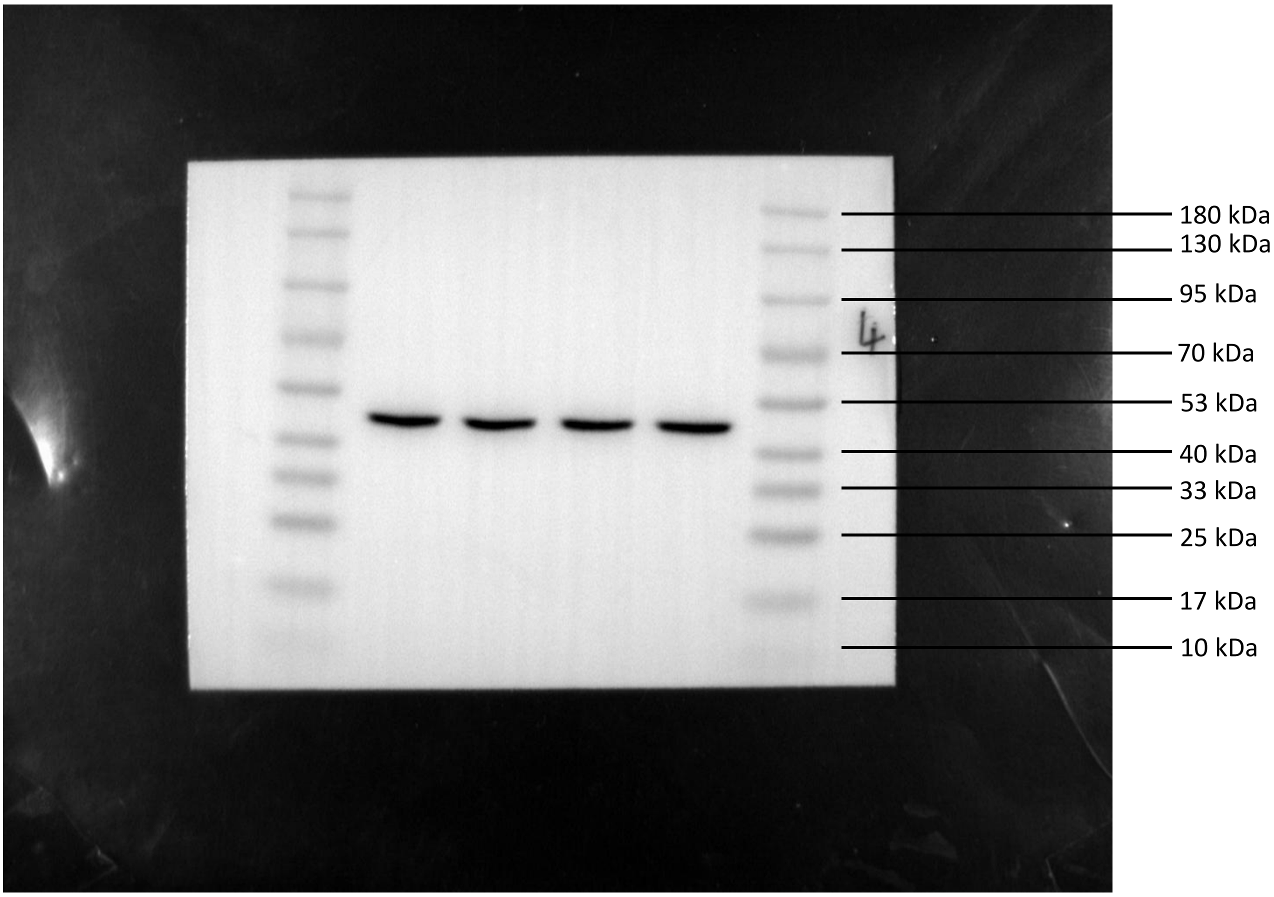

Lane1：Protein marker

Lane2：NC

Lane3：si-DNMT3A-1

Lane4：si-DNMT3A-2

Lane5：si-DNMT3A-3

Lane6：Protein marker

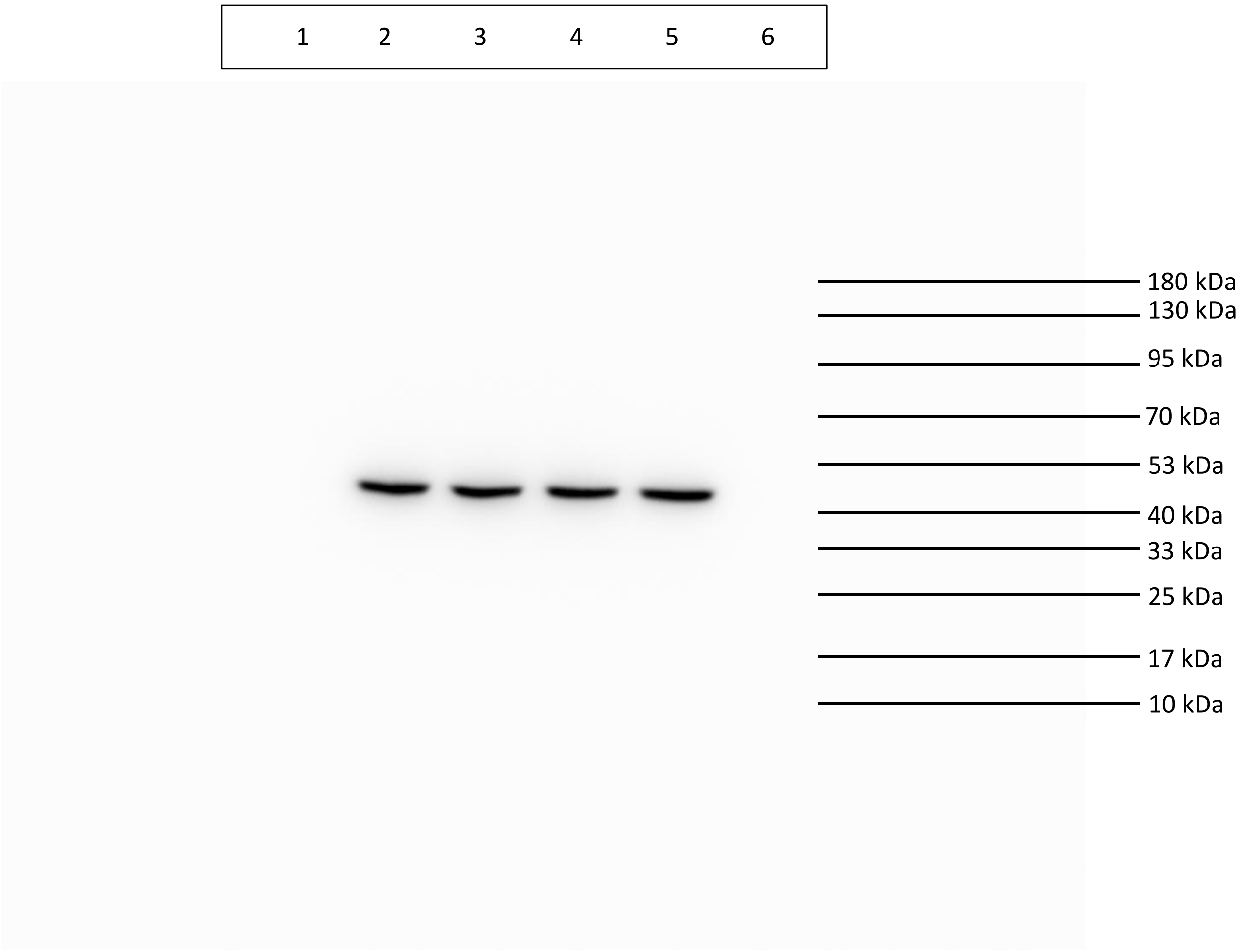

Lane1：Protein marker

Lane2：NC

Lane3：si-DNMT3A-1

Lane4：si-DNMT3A-2

Lane5：si-DNMT3A-3

Lane6：Protein marker

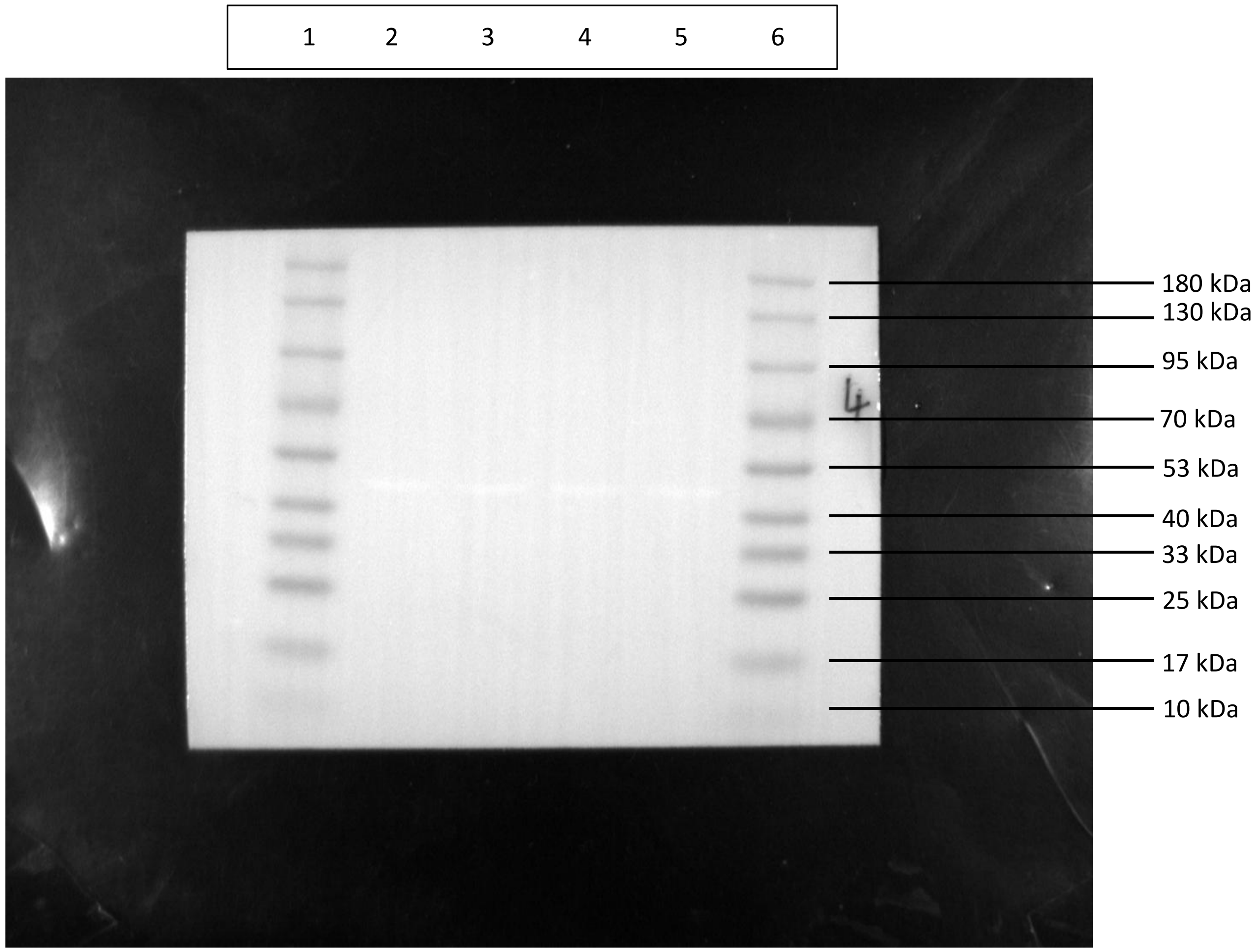

Lane1：Protein marker

Lane2：NC

Lane3：si-DNMT3A-1

Lane4：si-DNMT3A-2

Lane5：si-DNMT3A-3

Lane6：Protein marker

Figure 4F

DNMT3A

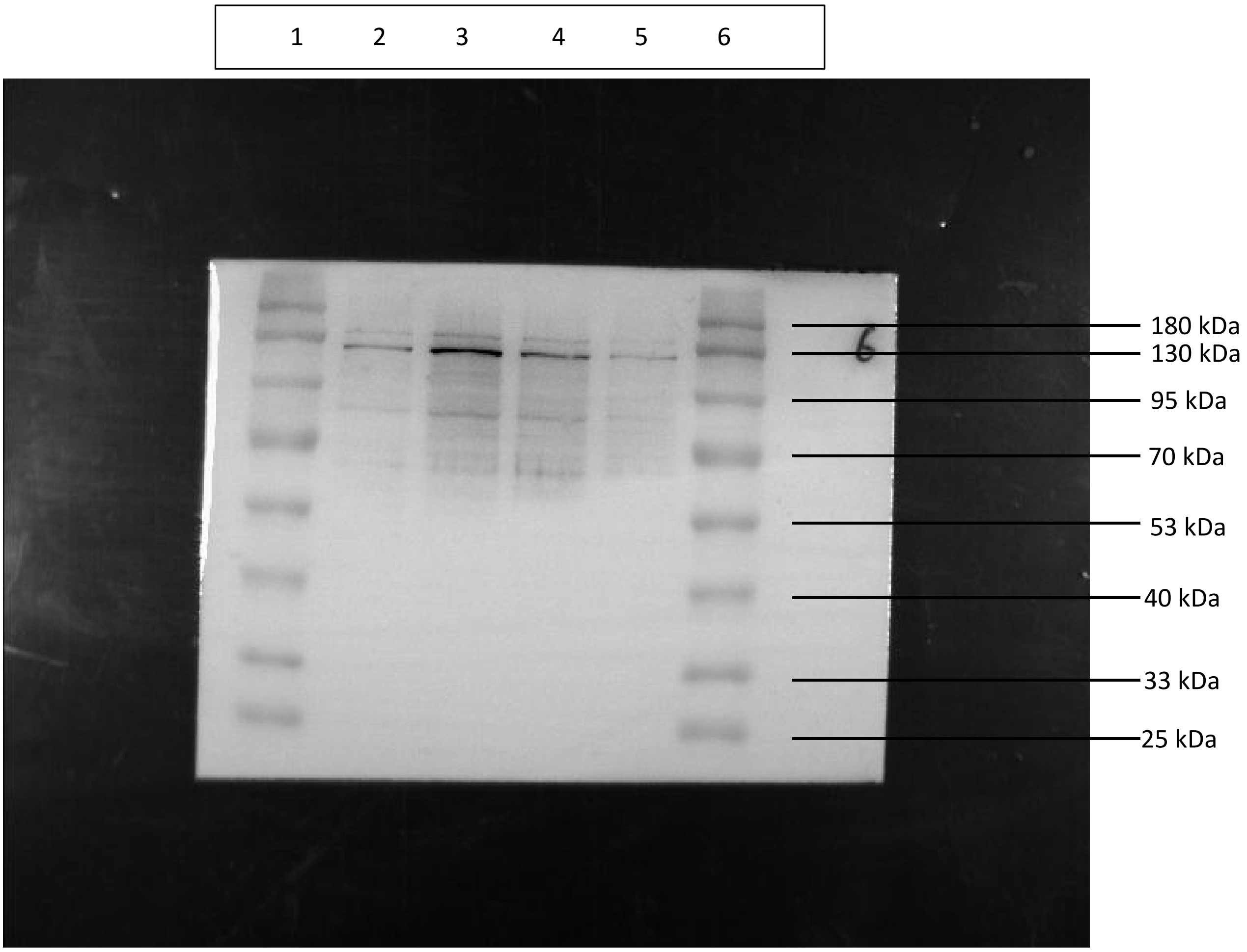

Lane1：Protein marker

Lane2：Vector

Lane3：DNMT3A

Lane4：si-NC

Lane5：si-DNMT3A

Lane6：Protein marker

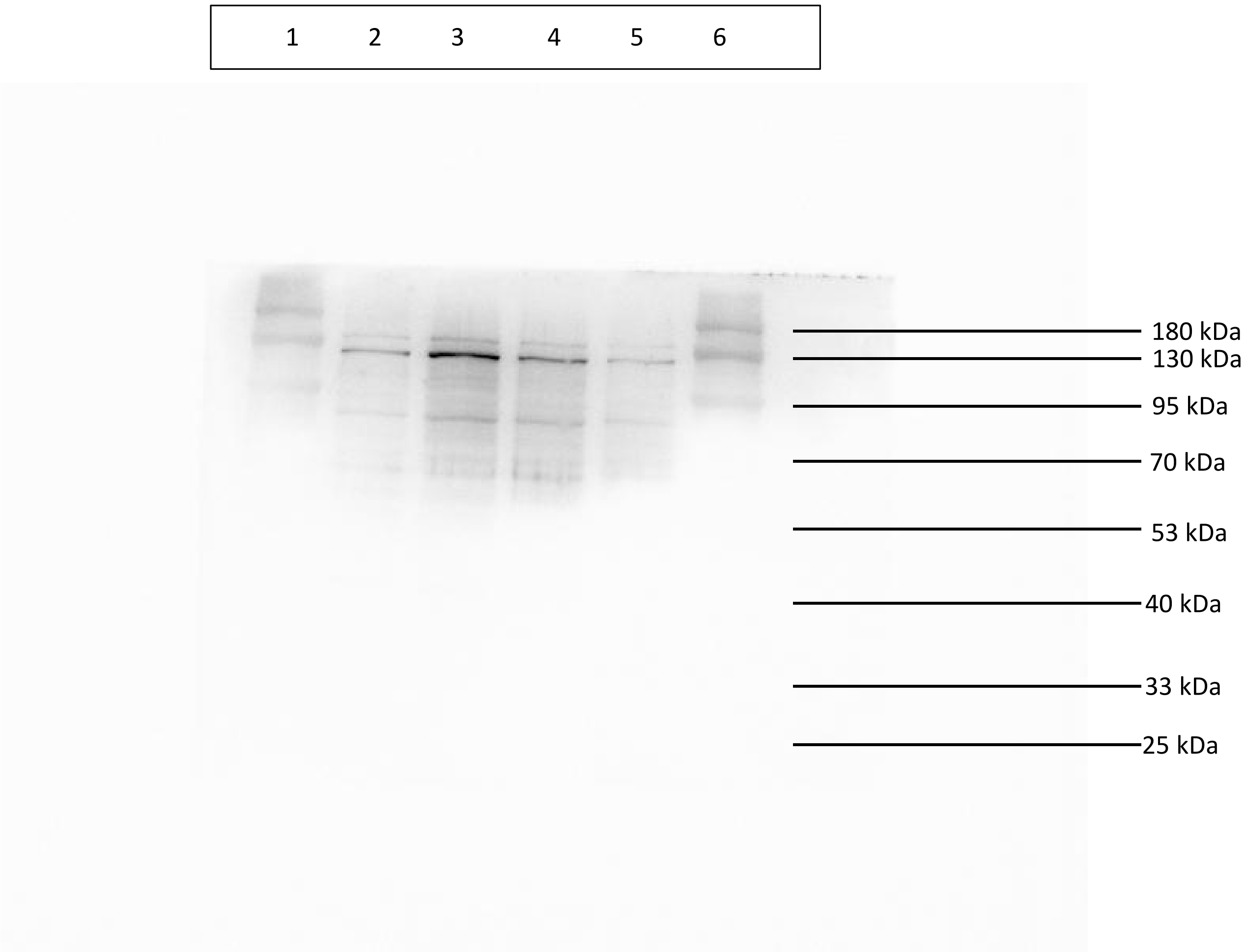

Lane1：Protein marker

Lane2：Vector

Lane3：DNMT3A

Lane4：si-NC

Lane5：si-DNMT3A

Lane6：Protein marker

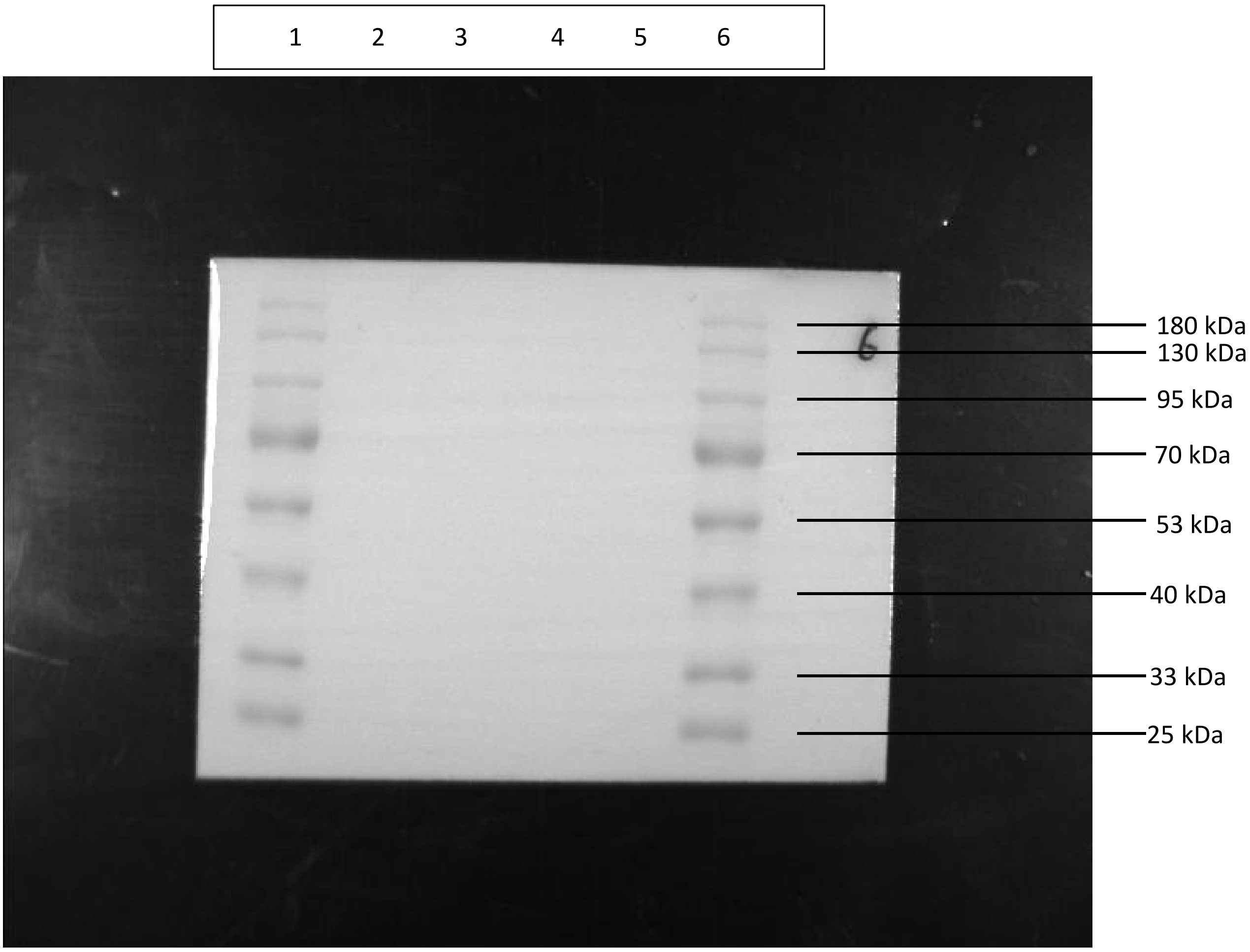

Lane1：Protein marker

Lane2：Vector

Lane3：DNMT3A

Lane4：si-NC

Lane5：si-DNMT3A

Lane6：Protein marker

Figure 4F

ADAMTS8

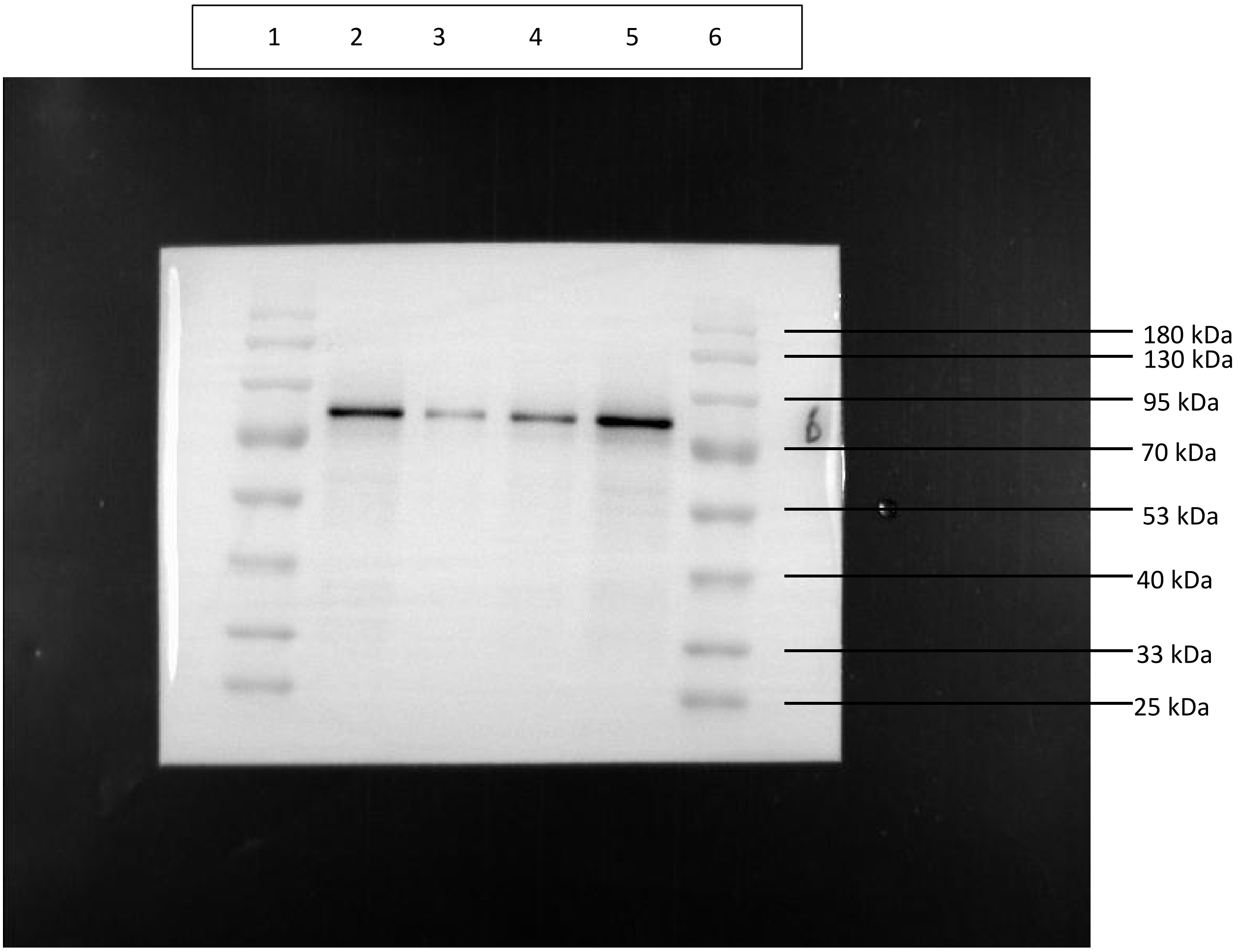

Lane1：Protein marker

Lane2：Vector

Lane3：DNMT3A

Lane4：si-NC

Lane5：si-DNMT3A

Lane6：Protein marker

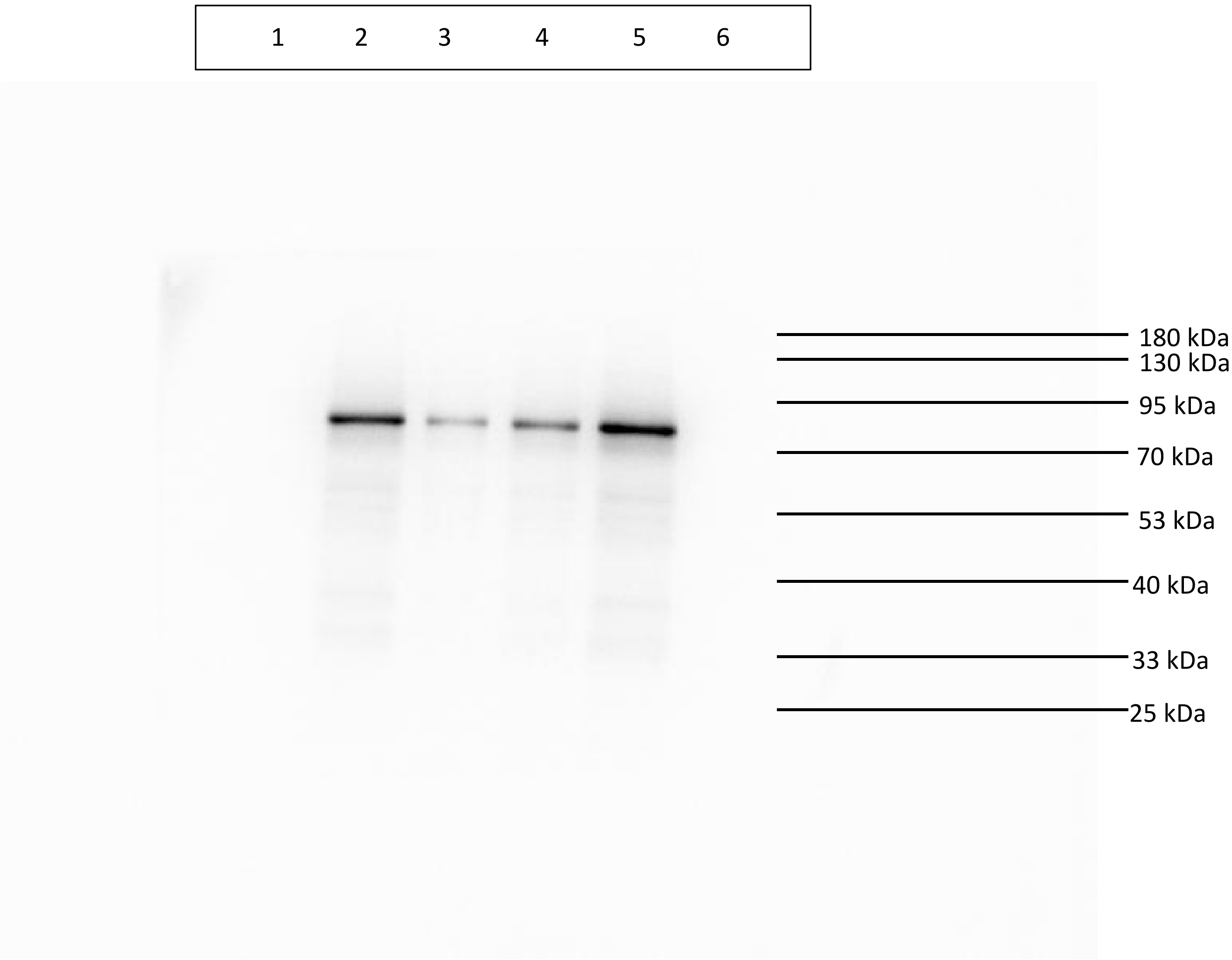

Lane1：Protein marker

Lane2：Vector

Lane3：DNMT3A

Lane4：si-NC

Lane5：si-DNMT3A

Lane6：Protein marker

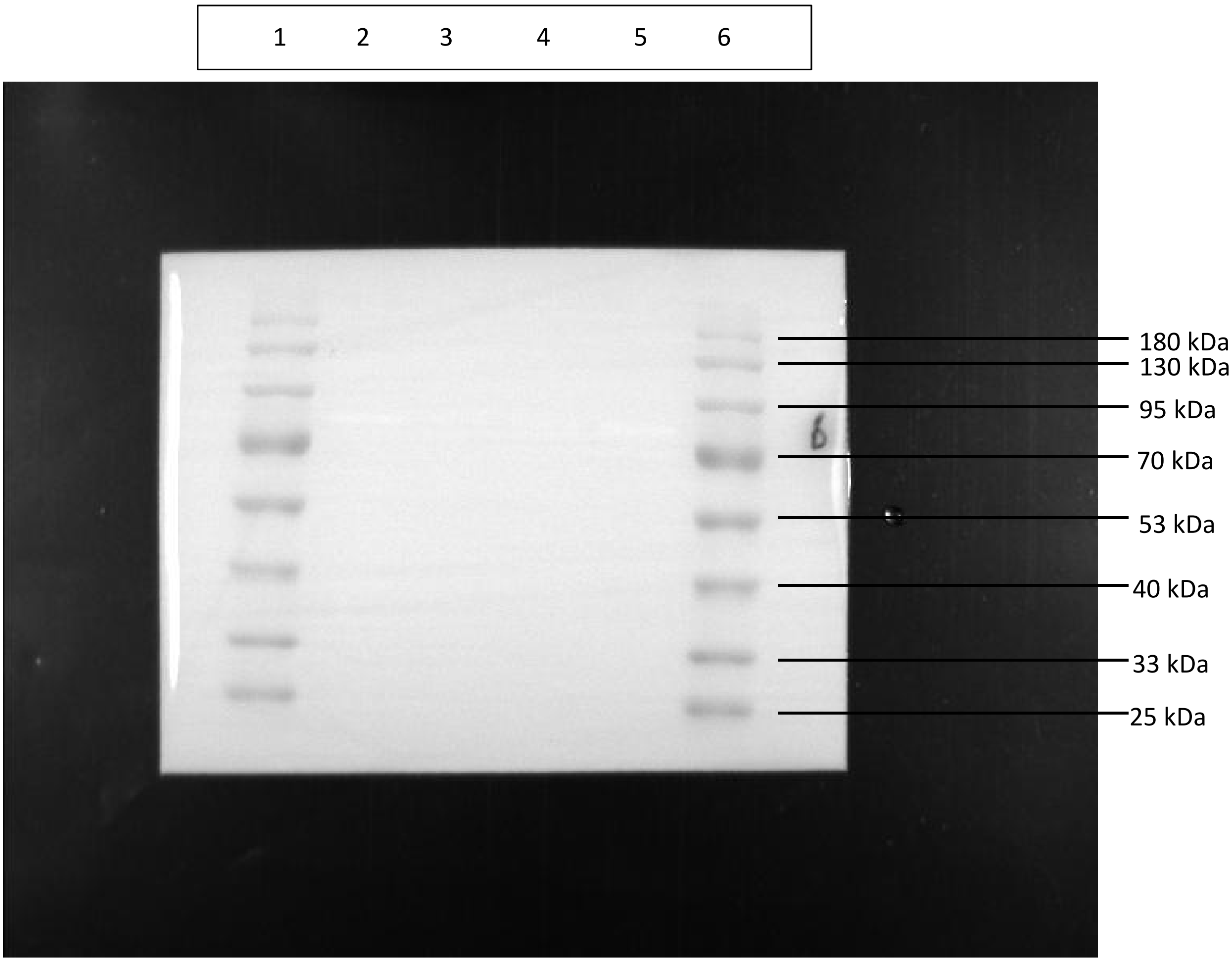

Lane1：Protein marker

Lane2：Vector

Lane3：DNMT3A

Lane4：si-NC

Lane5：si-DNMT3A

Lane6：Protein marker

Figure 4F

β-actin

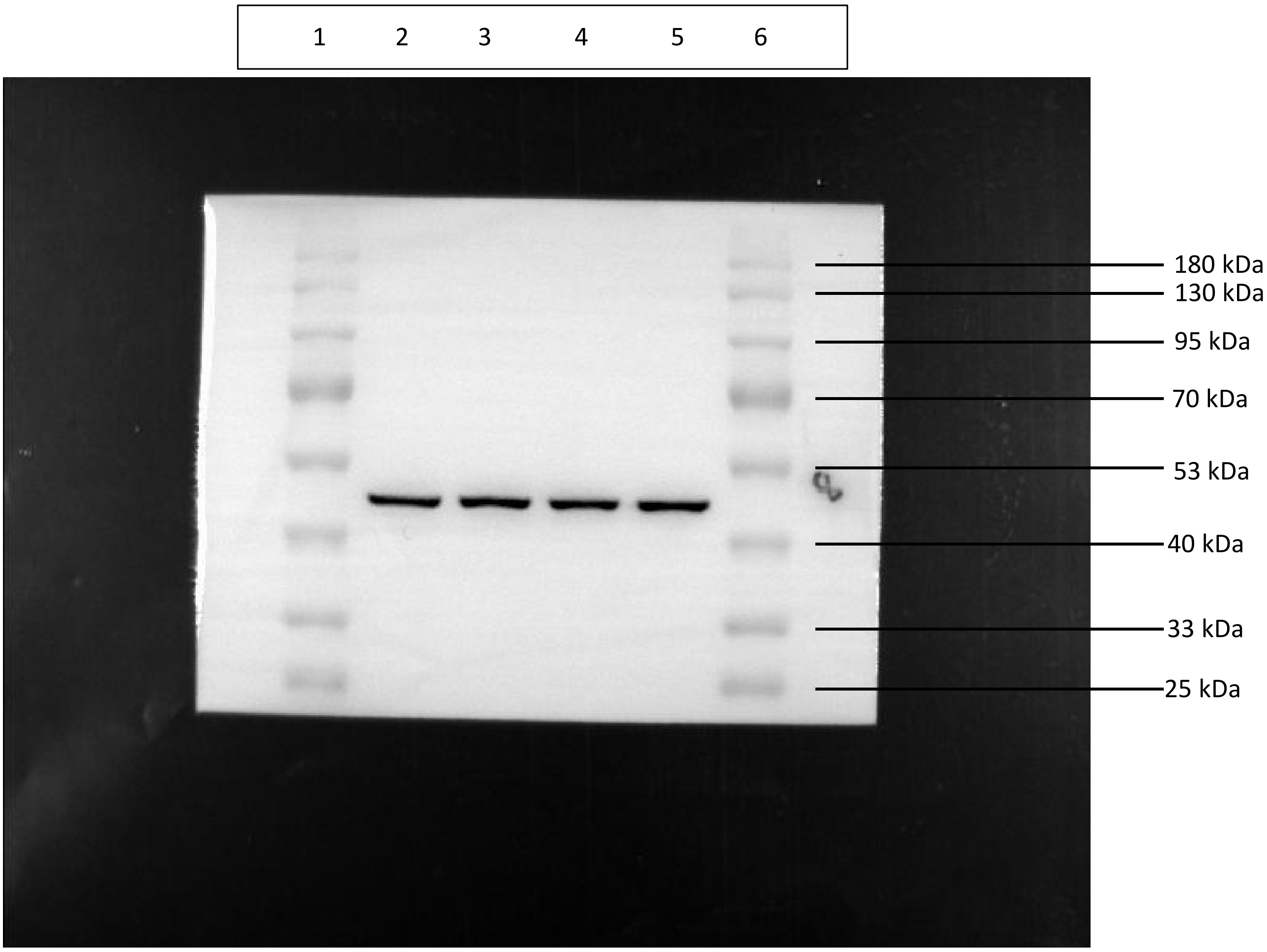

Lane1：Protein marker

Lane2：Vector

Lane3：DNMT3A

Lane4：si-NC

Lane5：si-DNMT3A

Lane6：Protein marker

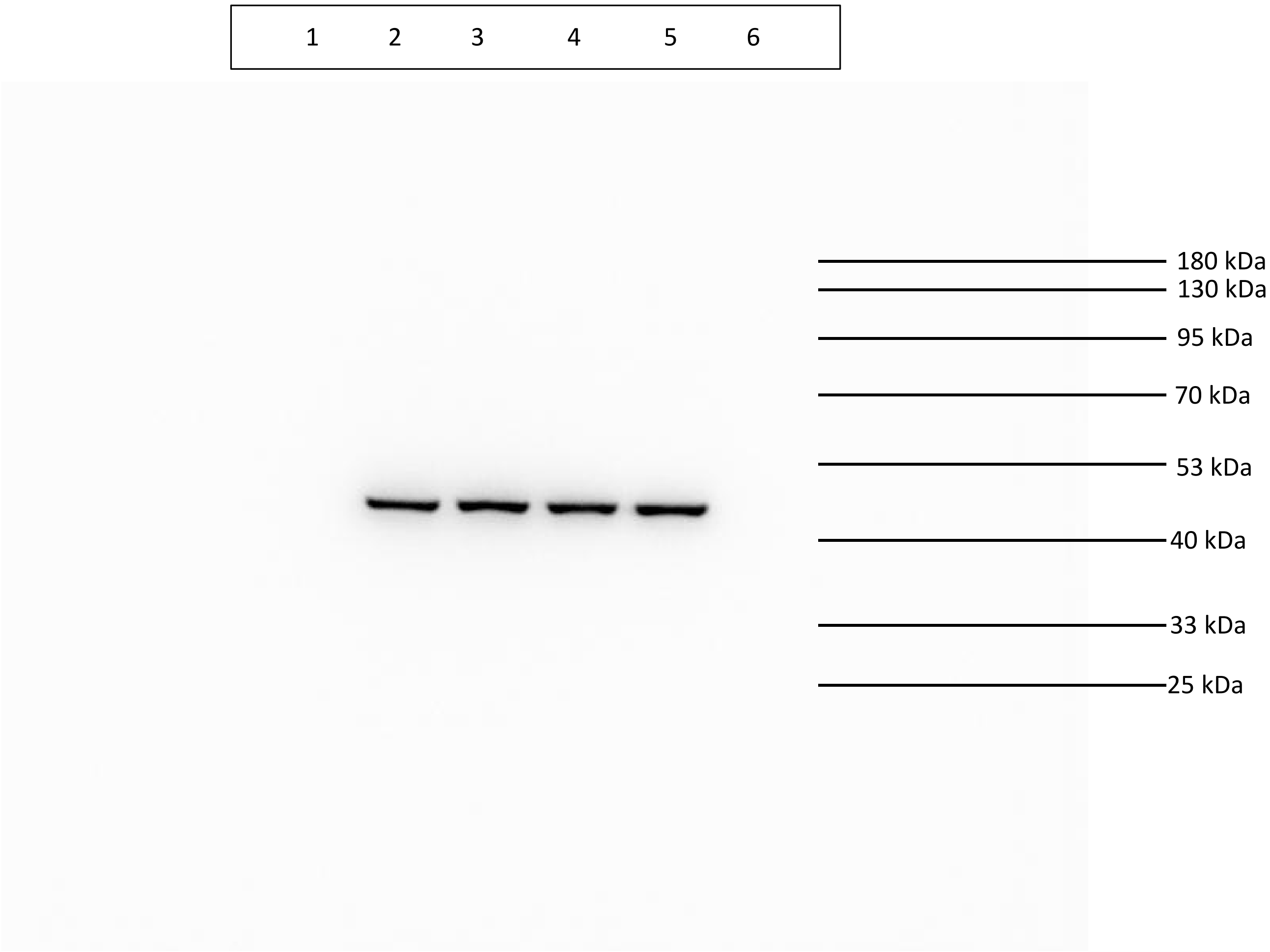

Lane1：Protein marker

Lane2：Vector

Lane3：DNMT3A

Lane4：si-NC

Lane5：si-DNMT3A

Lane6：Protein marker

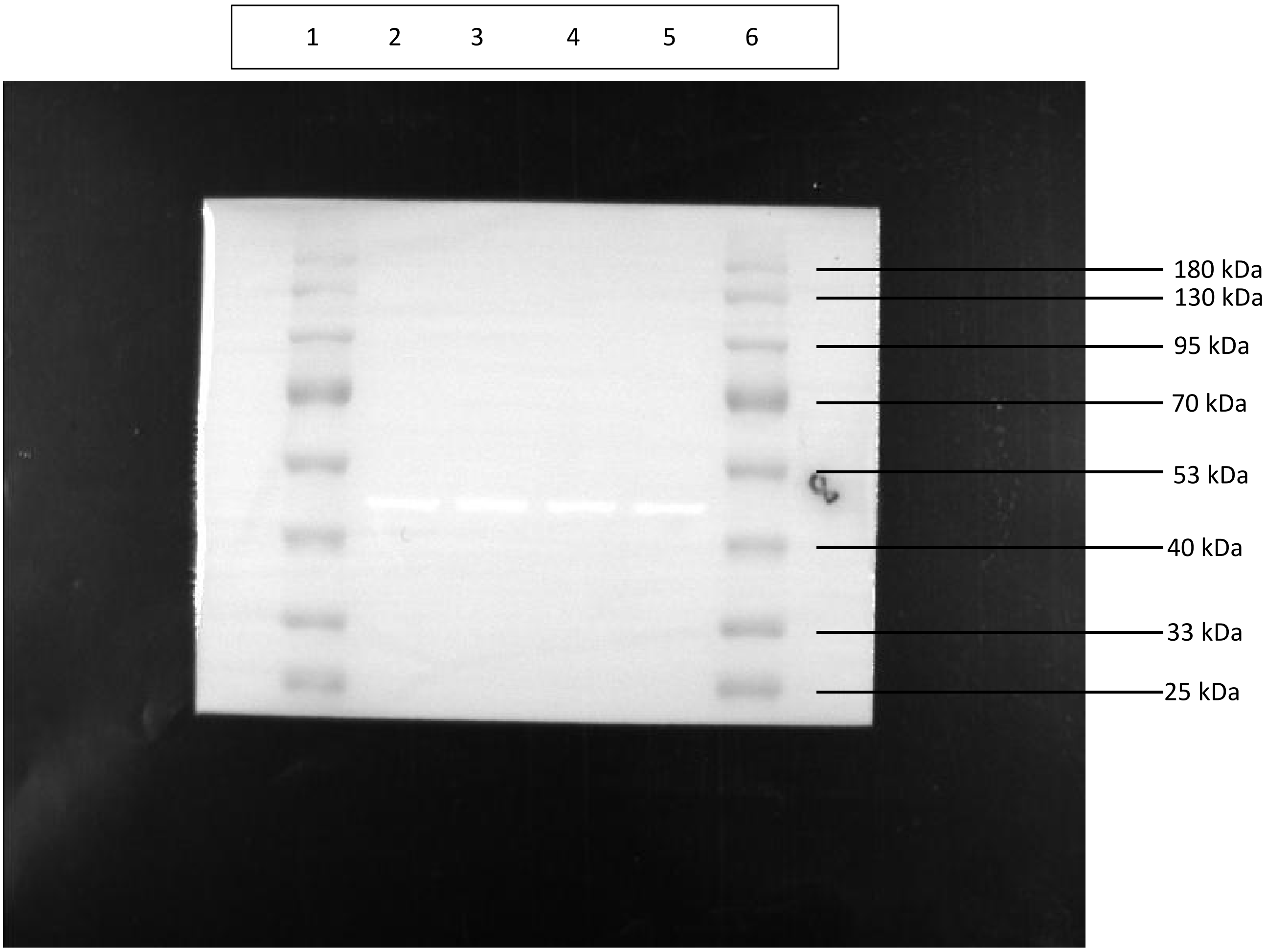

Lane1：Protein marker

Lane2：Vector

Lane3：DNMT3A

Lane4：si-NC

Lane5：si-DNMT3A

Lane6：Protein marker

Figure 4G

DNMT3A

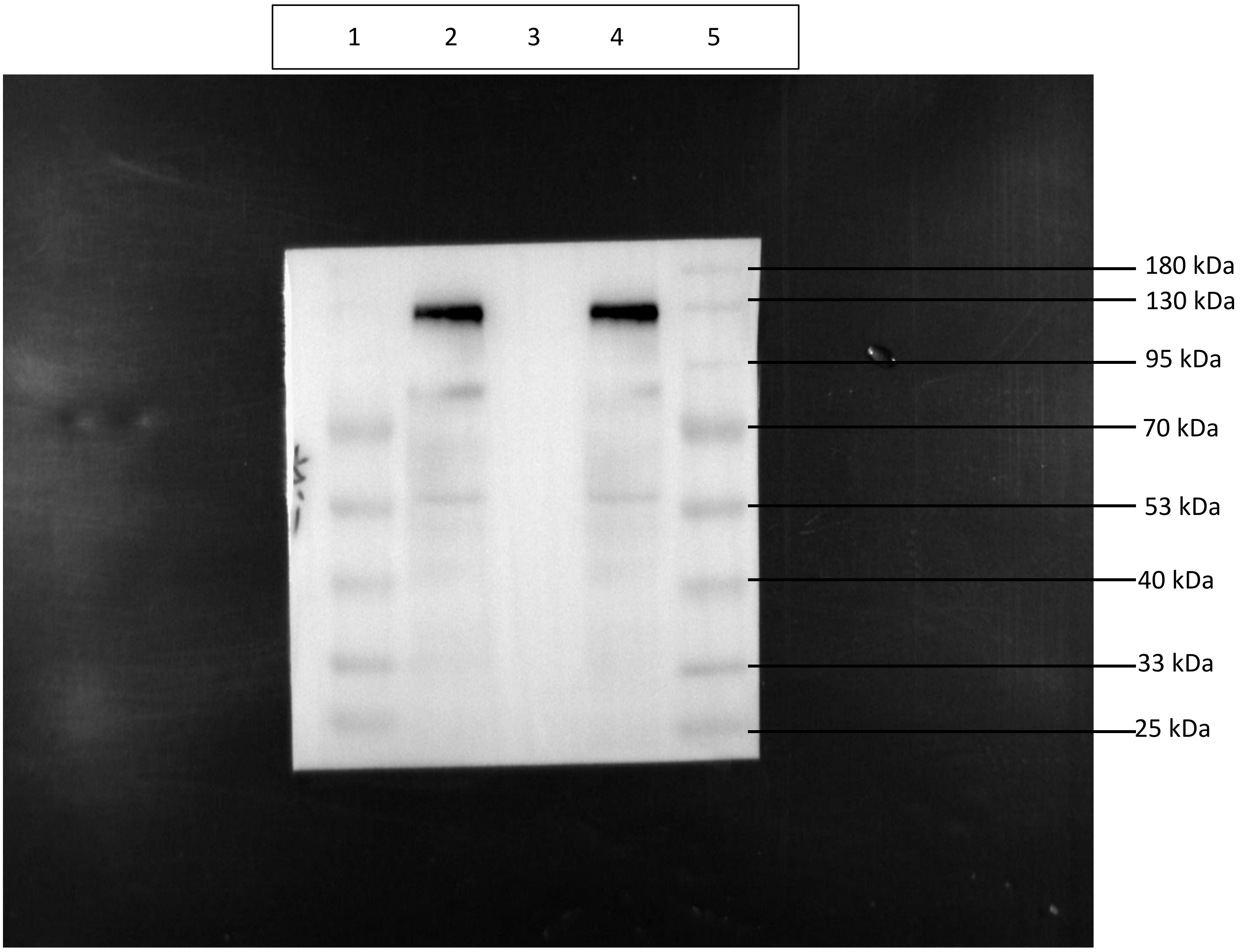

Lane1：Protein marker

Lane2：Input

Lane3：IgG

Lane4：DNMT3A

Lane5：Protein marker

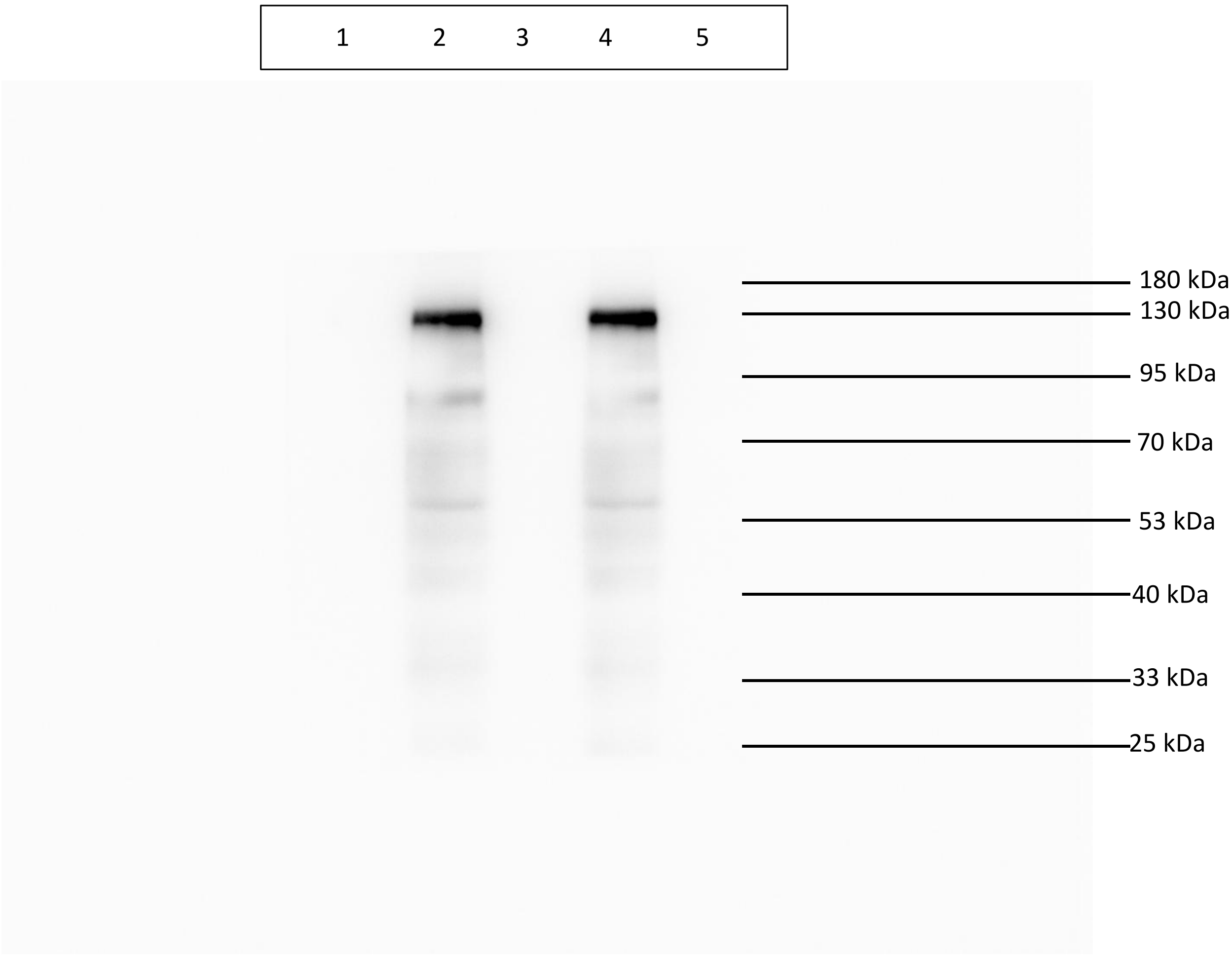

Lane1：Protein marker

Lane2：Input

Lane3：IgG

Lane4：DNMT3A

Lane5：Protein marker

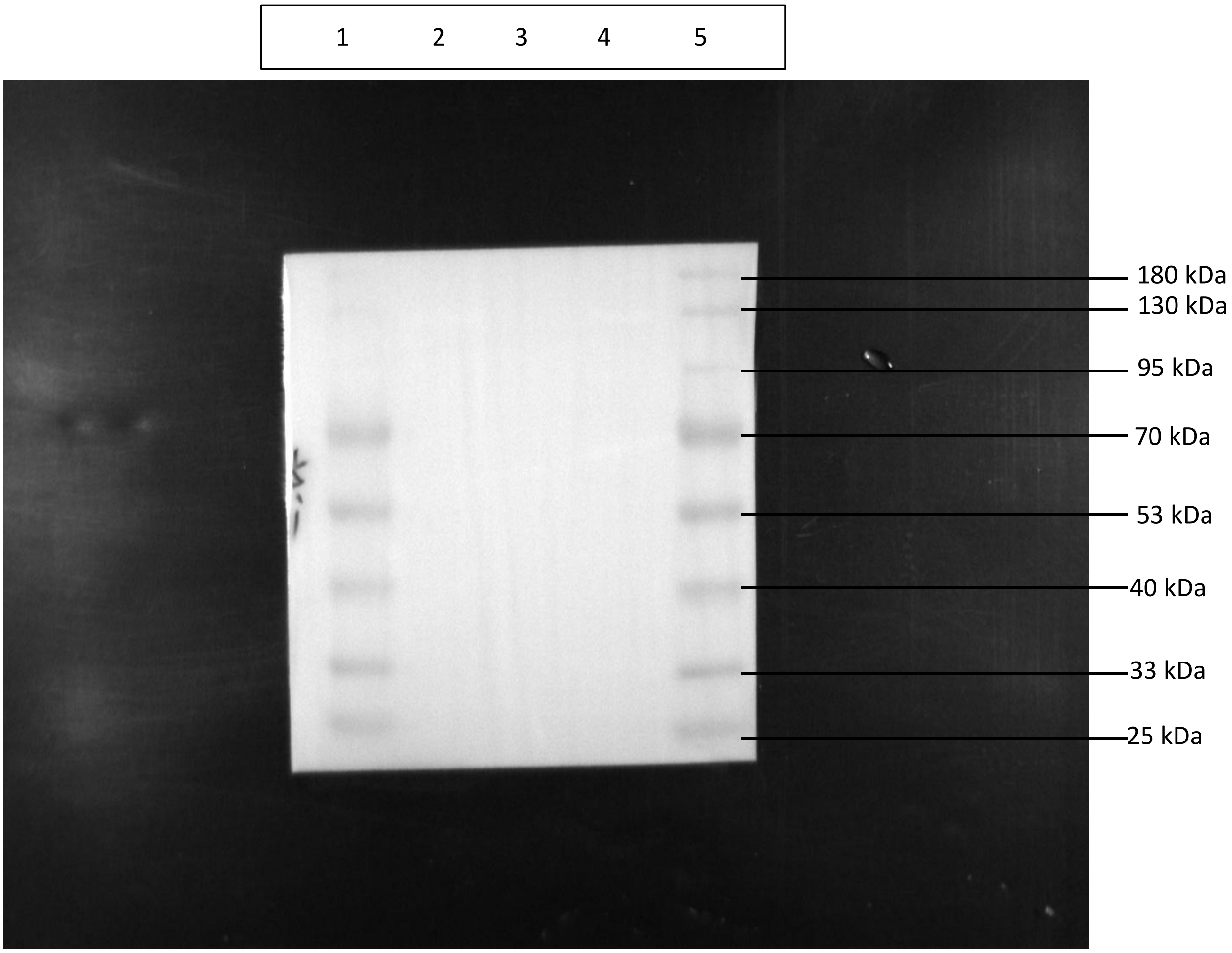

Lane1：Protein marker

Lane2：Input

Lane3：IgG

Lane4：DNMT3A

Lane5：Protein marker

Figure 4G

ADAMTS8

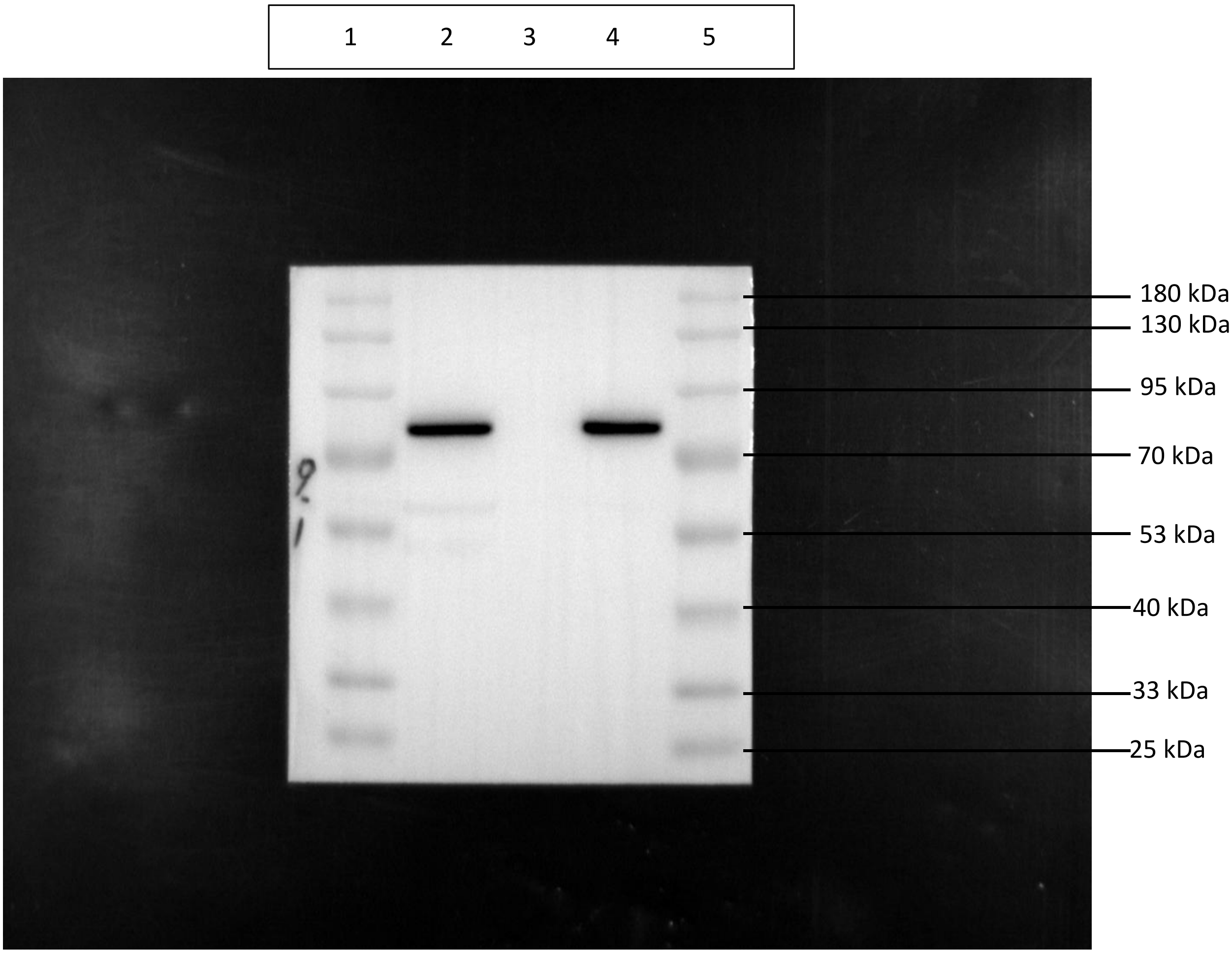

Lane1：Protein marker

Lane2：Input

Lane3：IgG

Lane4：DNMT3A

Lane5：Protein marker

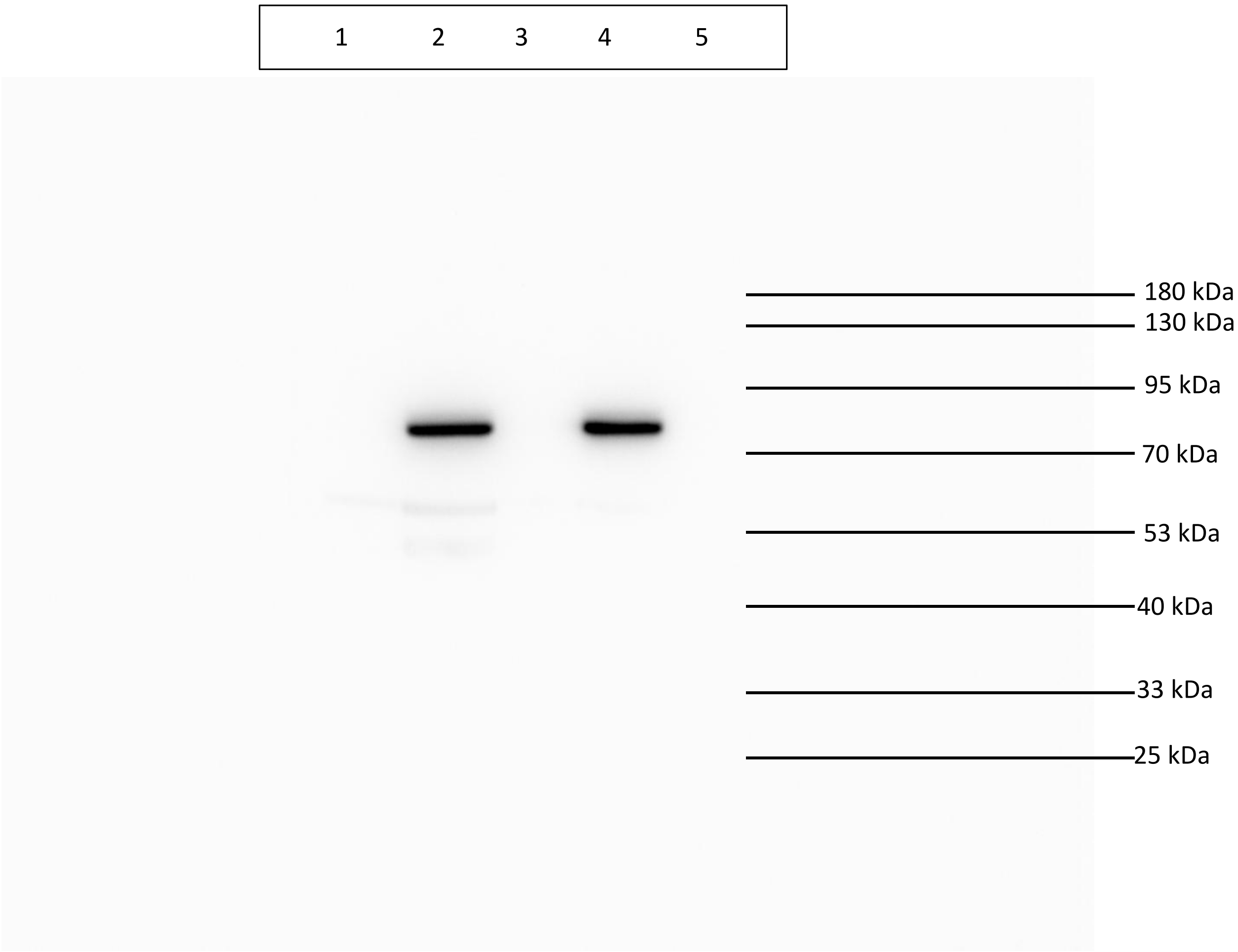

Lane1：Protein marker

Lane2：Input

Lane3：IgG

Lane4：DNMT3A

Lane5：Protein marker

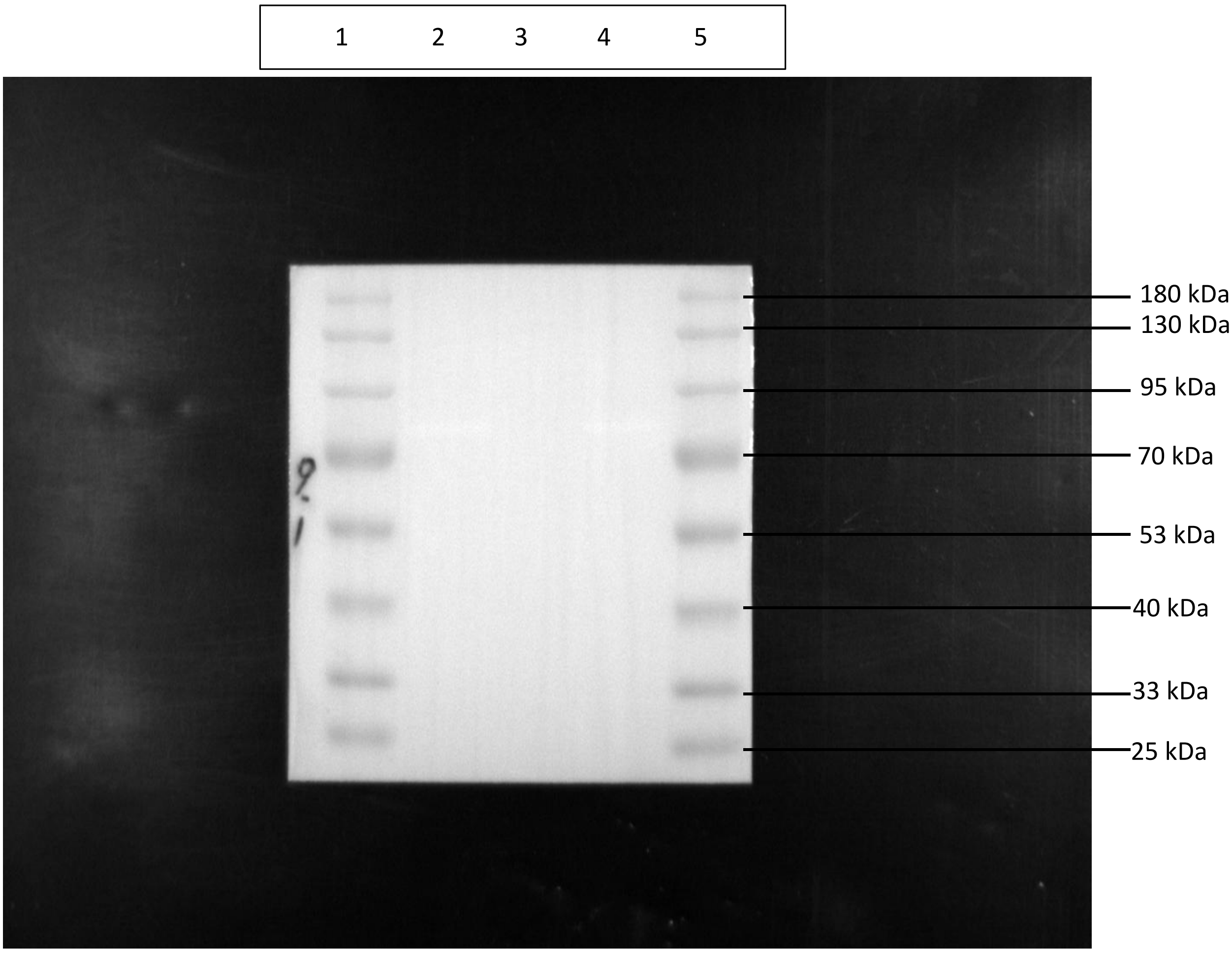

Lane1：Protein marker

Lane2：Input

Lane3：IgG

Lane4：DNMT3A

Lane5：Protein marker

Figure 4G

ADAMTS8

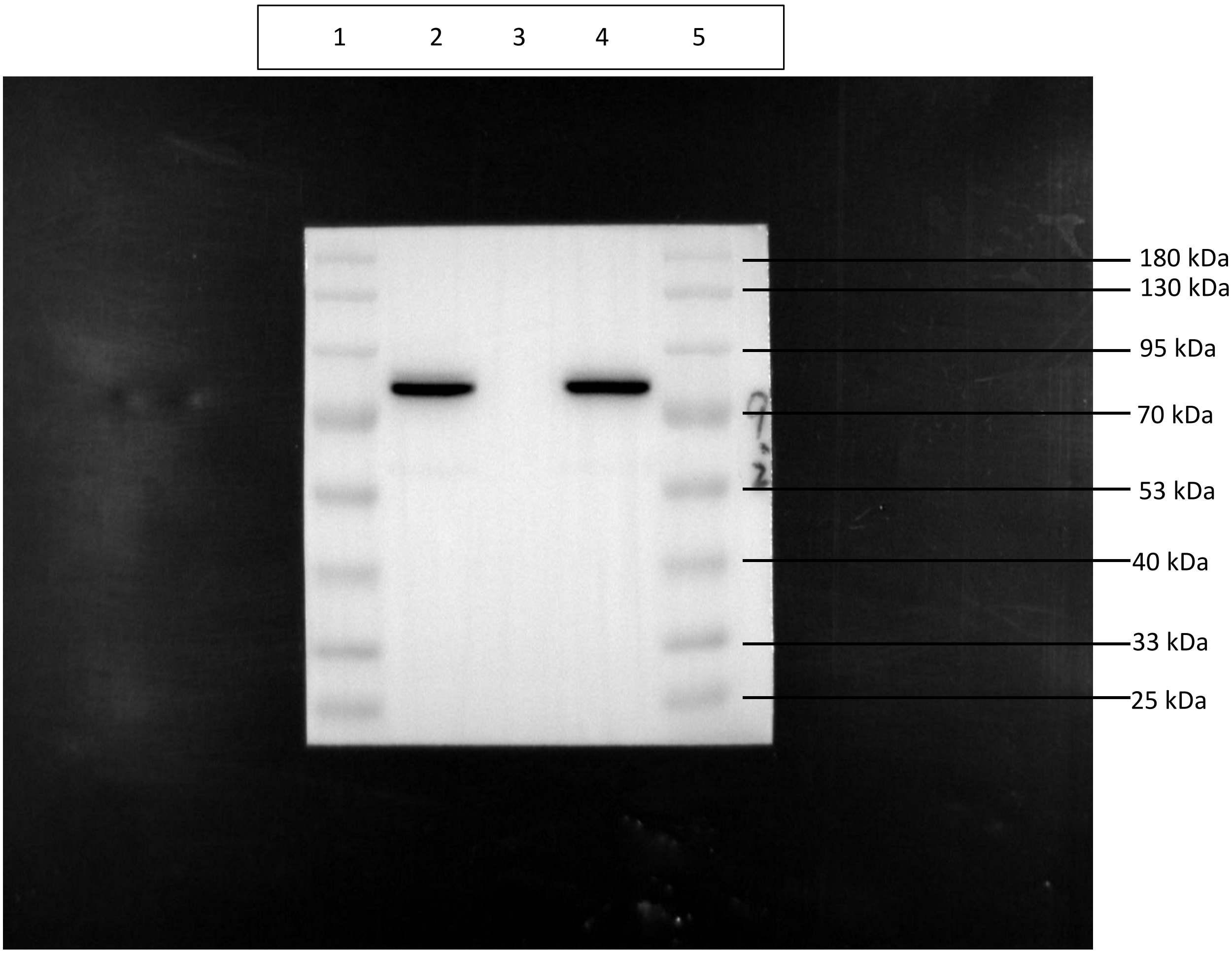

Lane1：Protein marker

Lane2：Input

Lane3：IgG

Lane4：ADAMTS8

Lane5：Protein marker

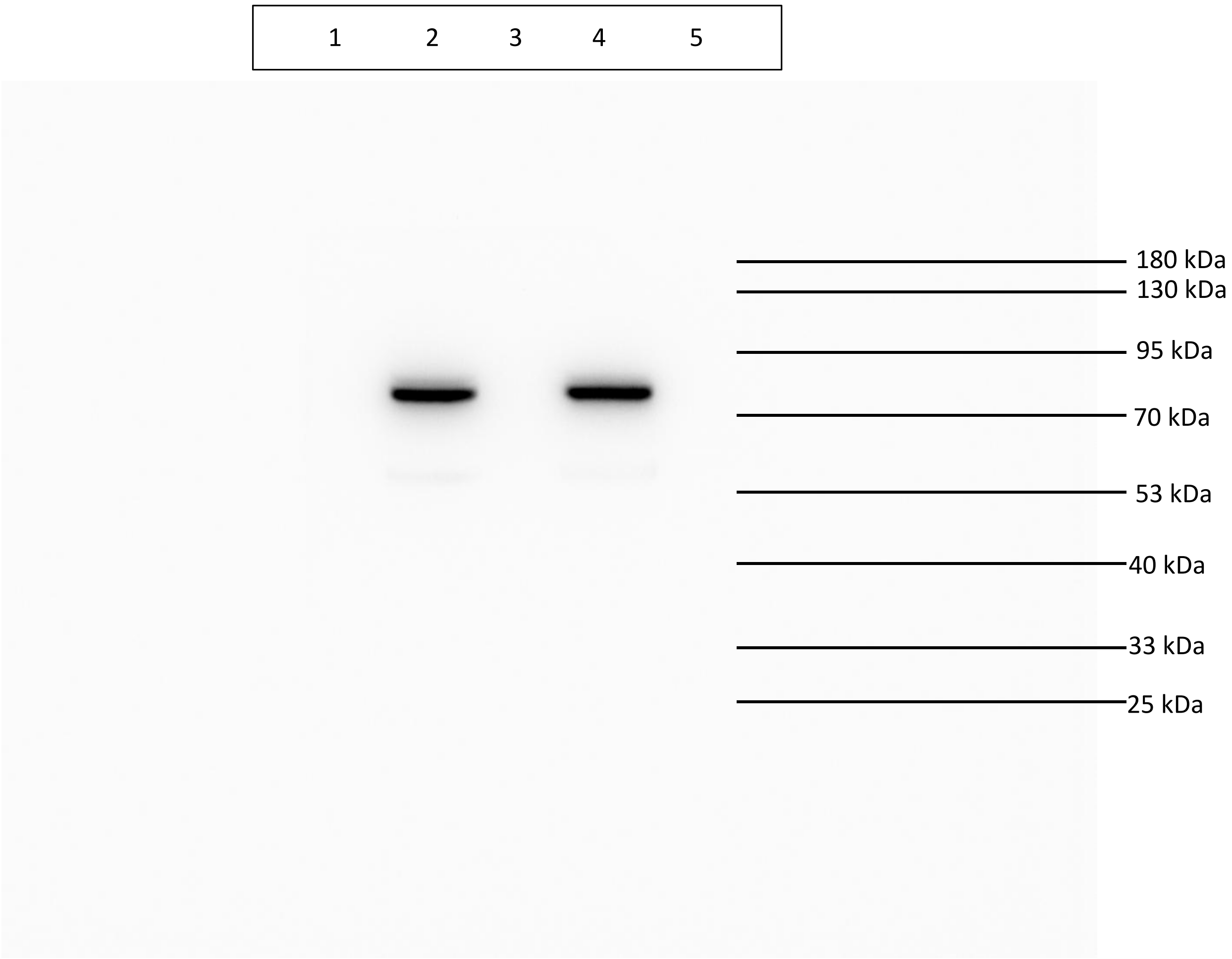

Lane1：Protein marker

Lane2：Input

Lane3：IgG

Lane4：ADAMTS8

Lane5：Protein marker

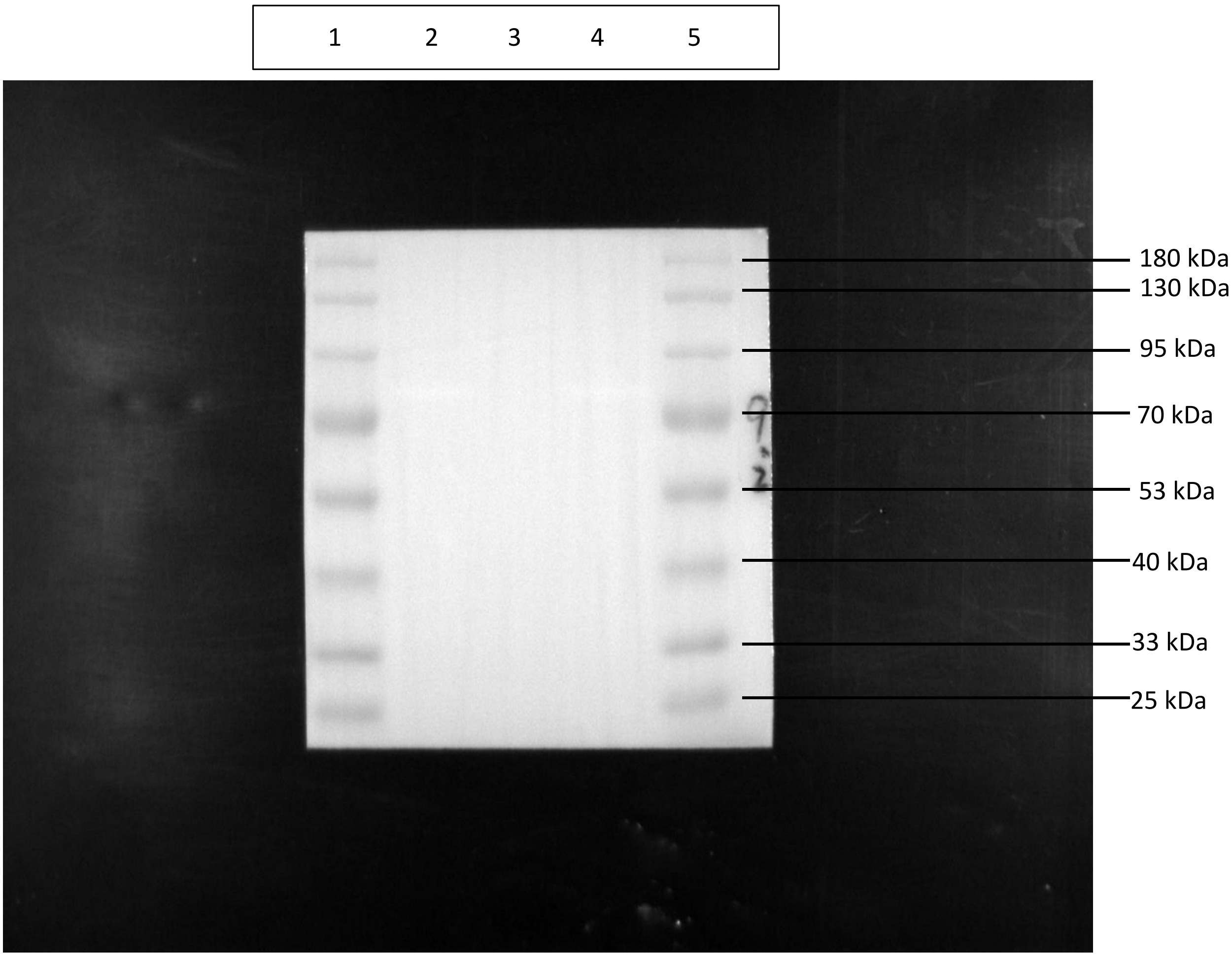

Lane1：Protein marker

Lane2：Input

Lane3：IgG

Lane4：ADAMTS8

Lane5：Protein marker

Figure 4G

DNMT3A

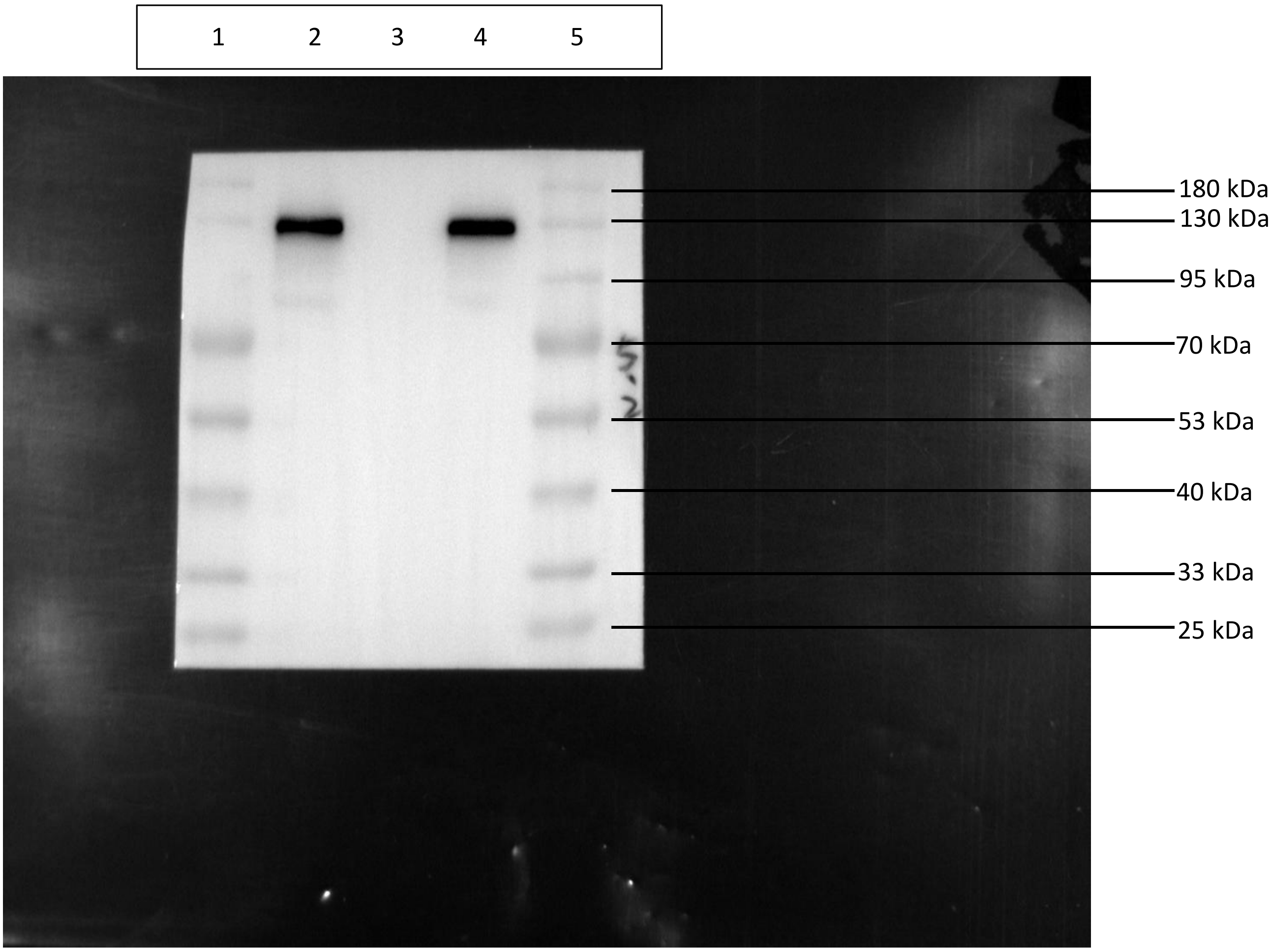

Lane1：Protein marker

Lane2：Input

Lane3：IgG

Lane4：ADAMTS8

Lane5：Protein marker

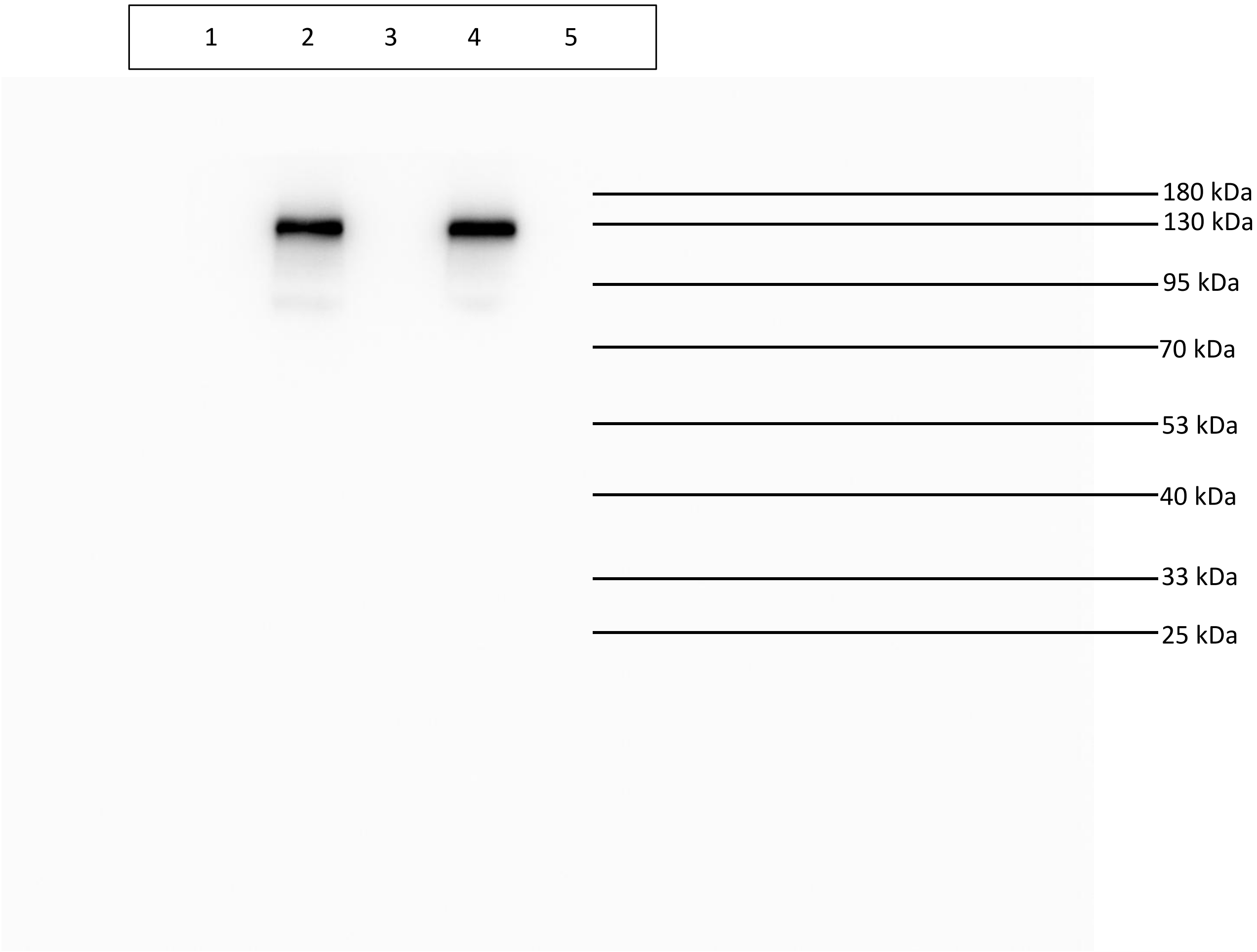

Lane1：Protein marker

Lane2：Input

Lane3：IgG

Lane4：ADAMTS8

Lane5：Protein marker

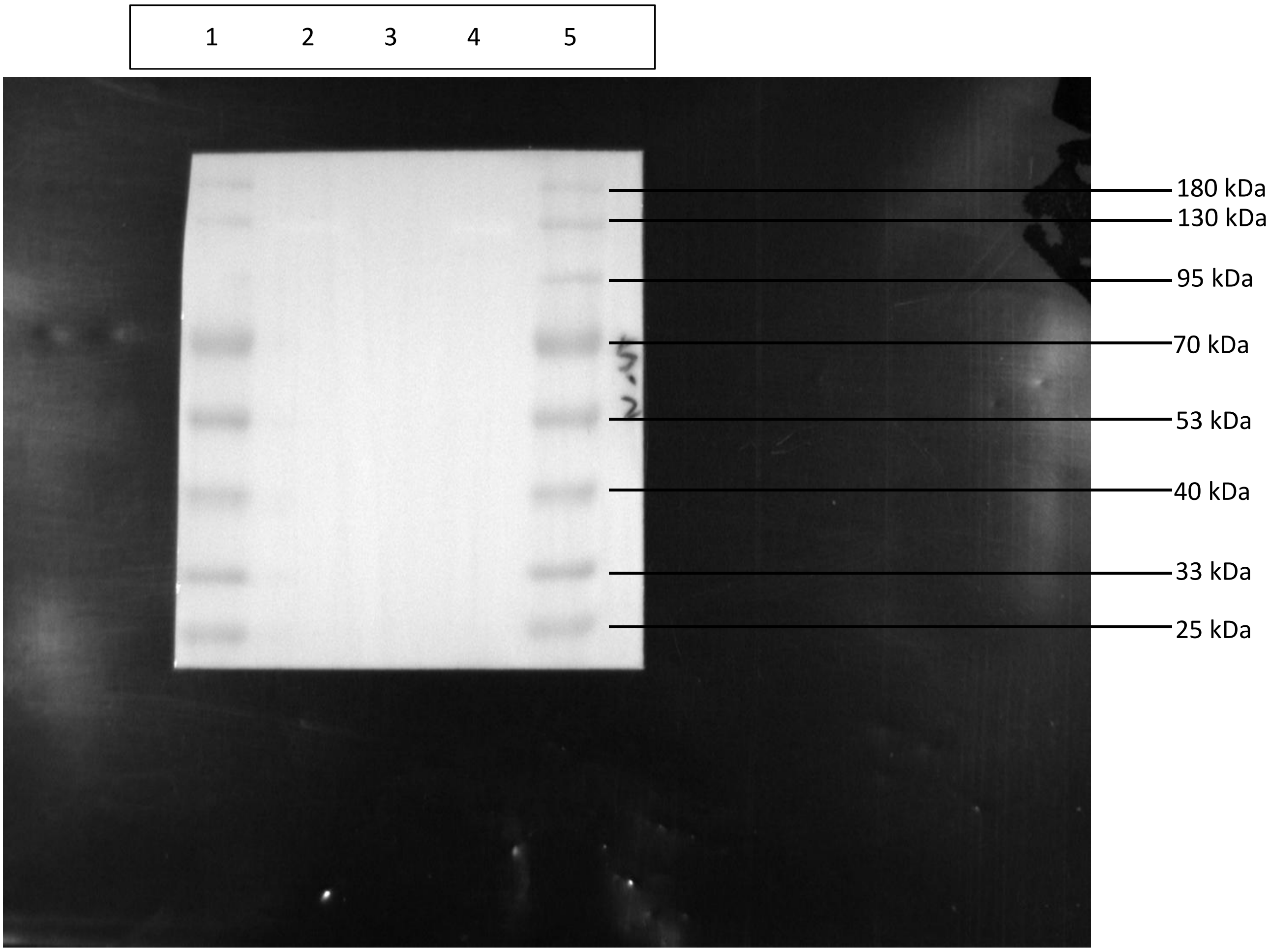

Lane1：Protein marker

Lane2：Input

Lane3：IgG

Lane4：ADAMTS8

Lane5：Protein marker

Figure 6D

DNMT3A

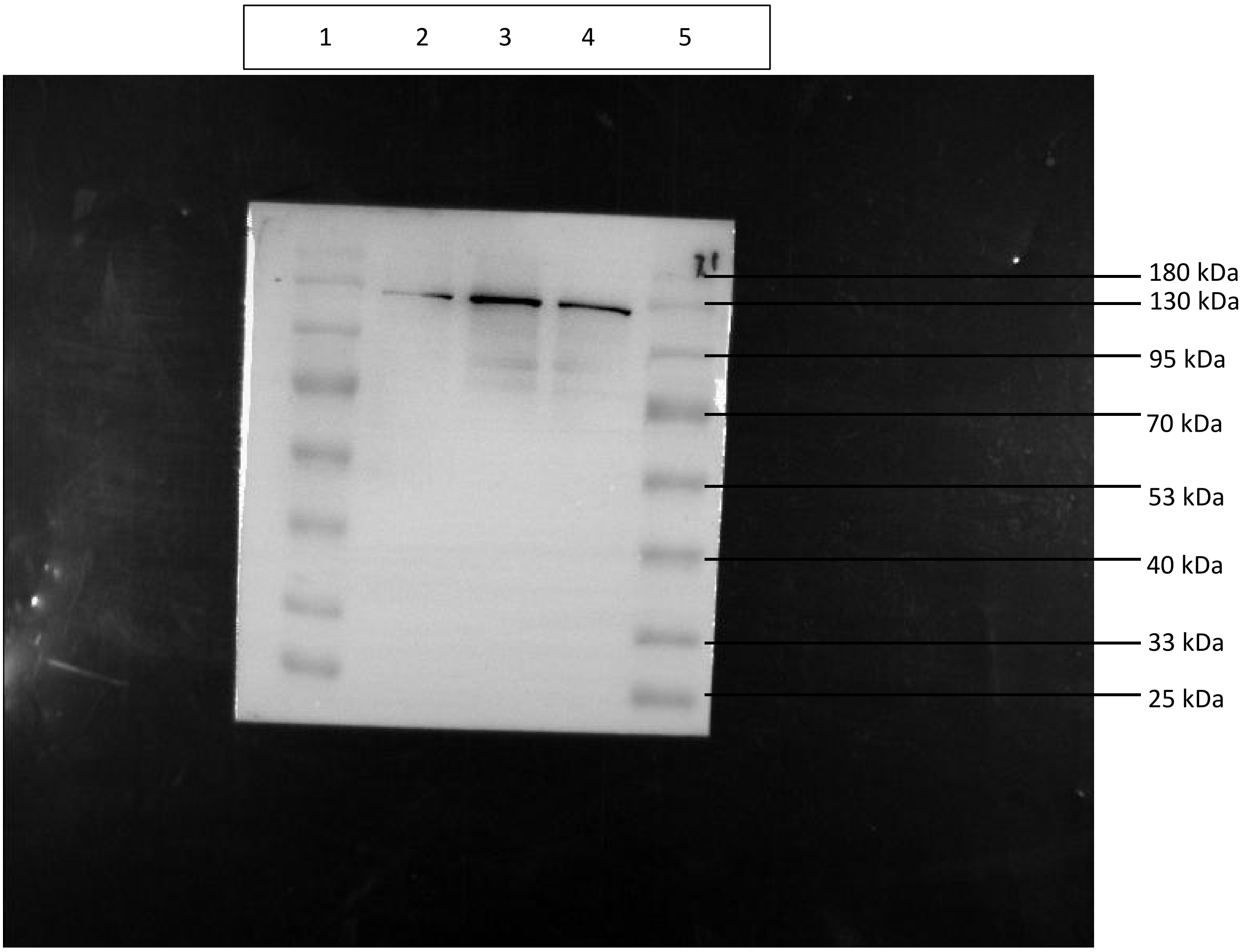

Lane1：Protein marker

Lane2：Vector

Lane3：DNMT3A

Lane4：DNMT3A+DAC

Lane5：Protein marker

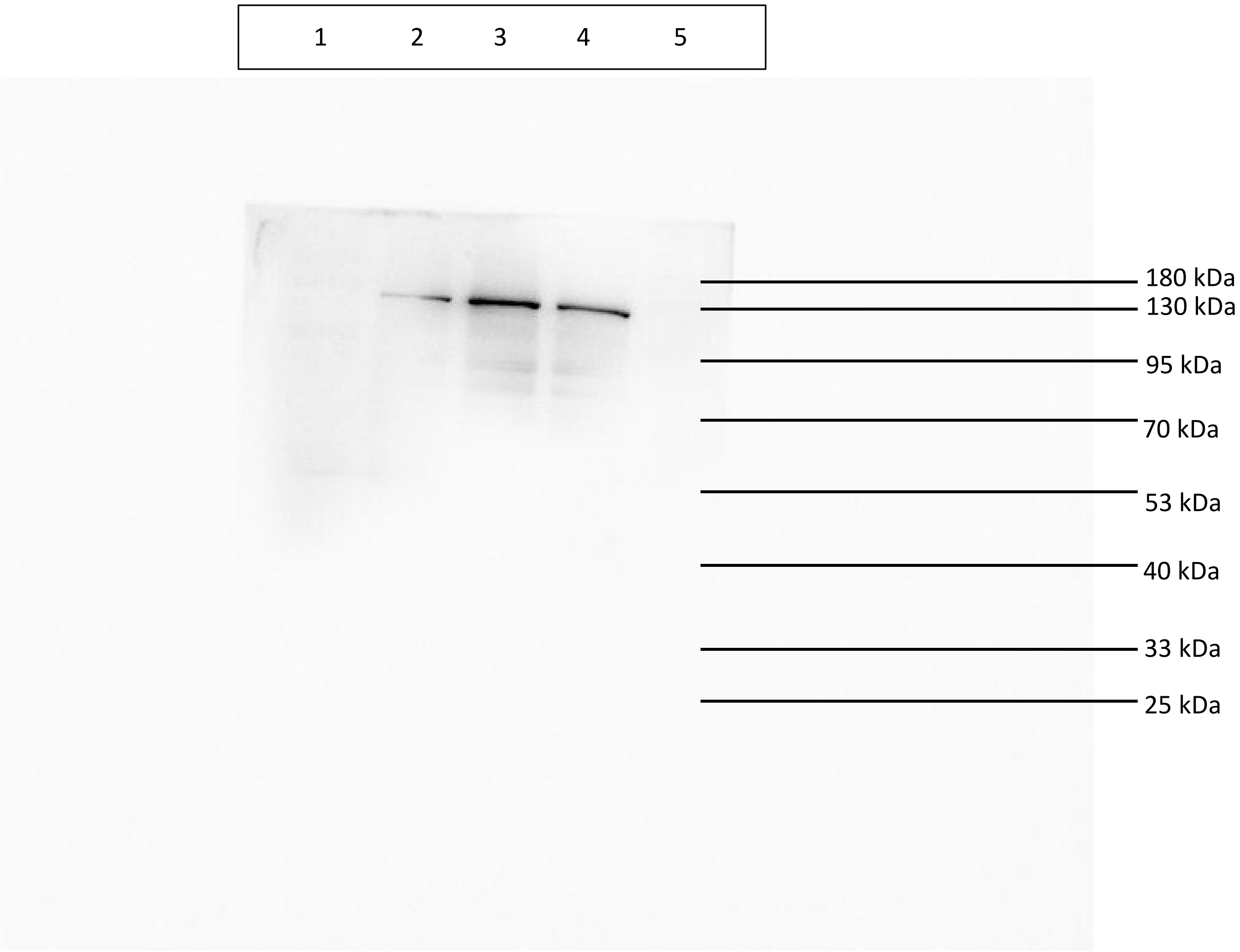

Lane1：Protein marker

Lane2：Vector

Lane3：DNMT3A

Lane4：DNMT3A+DAC

Lane5：Protein marker

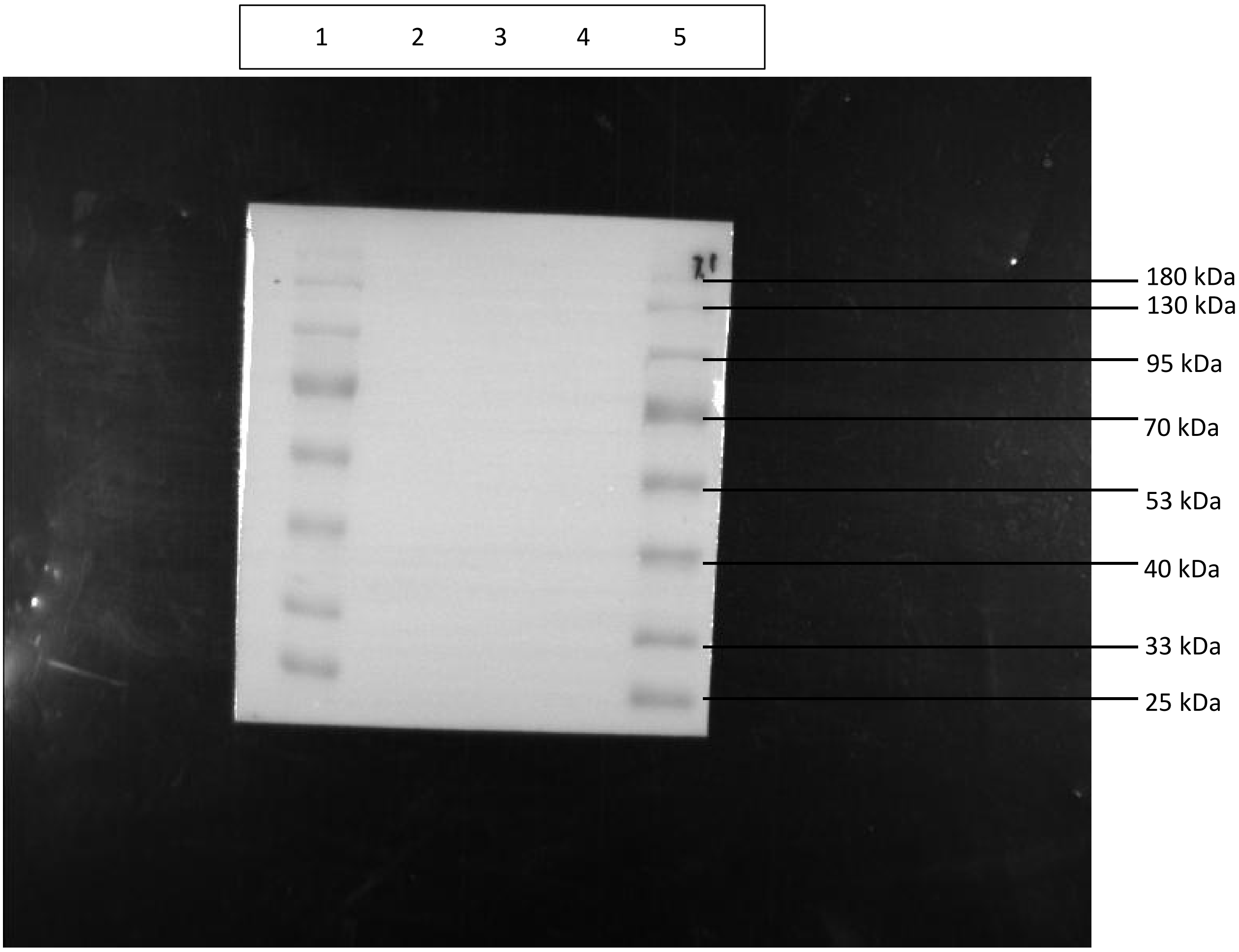

Lane1：Protein marker

Lane2：Vector

Lane3：DNMT3A

Lane4：DNMT3A+DAC

Lane5：Protein marker

Figure 6D

ADAMTS8

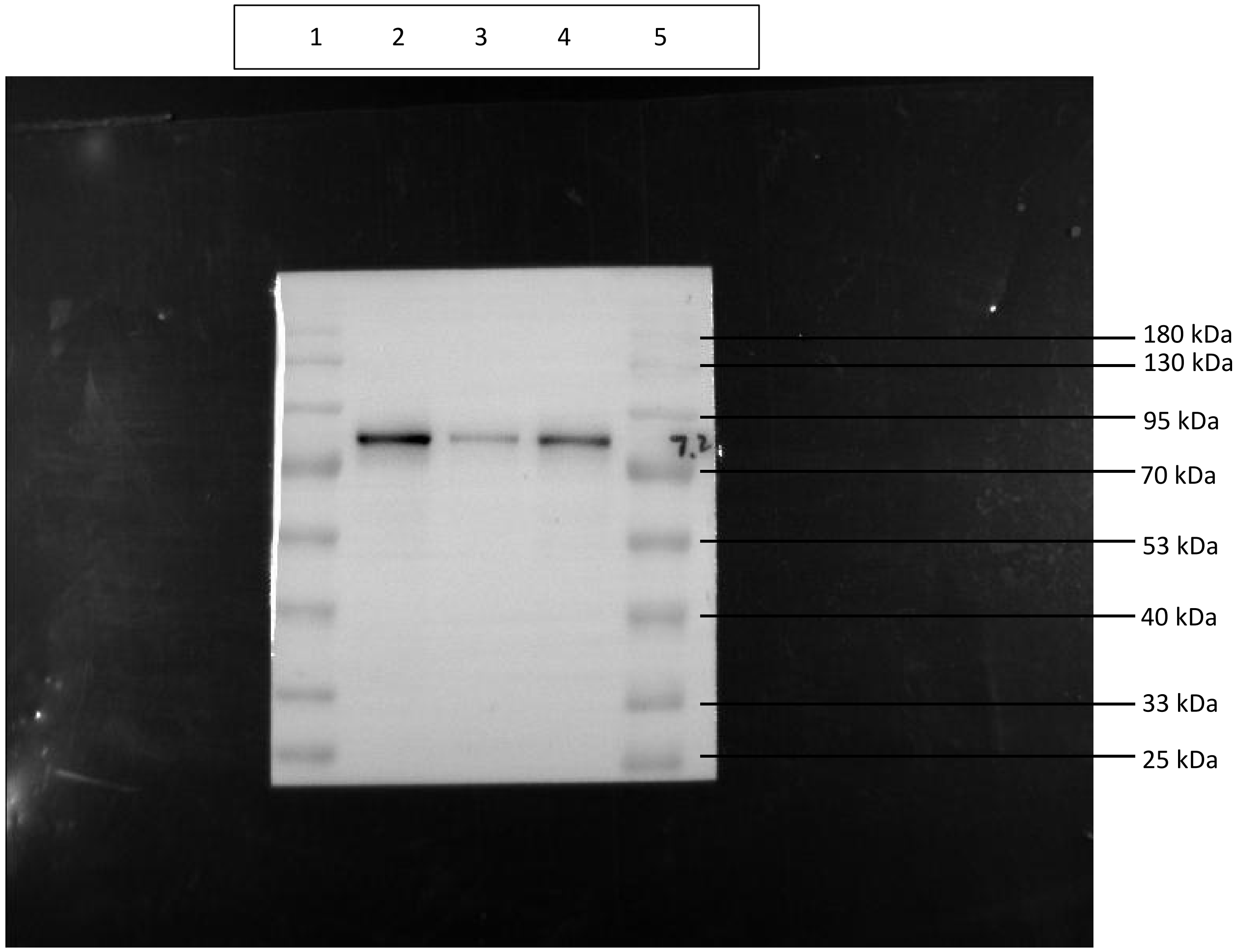

Lane1：Protein marker

Lane2：Vector

Lane3：DNMT3A

Lane4：DNMT3A+DAC

Lane5：Protein marker

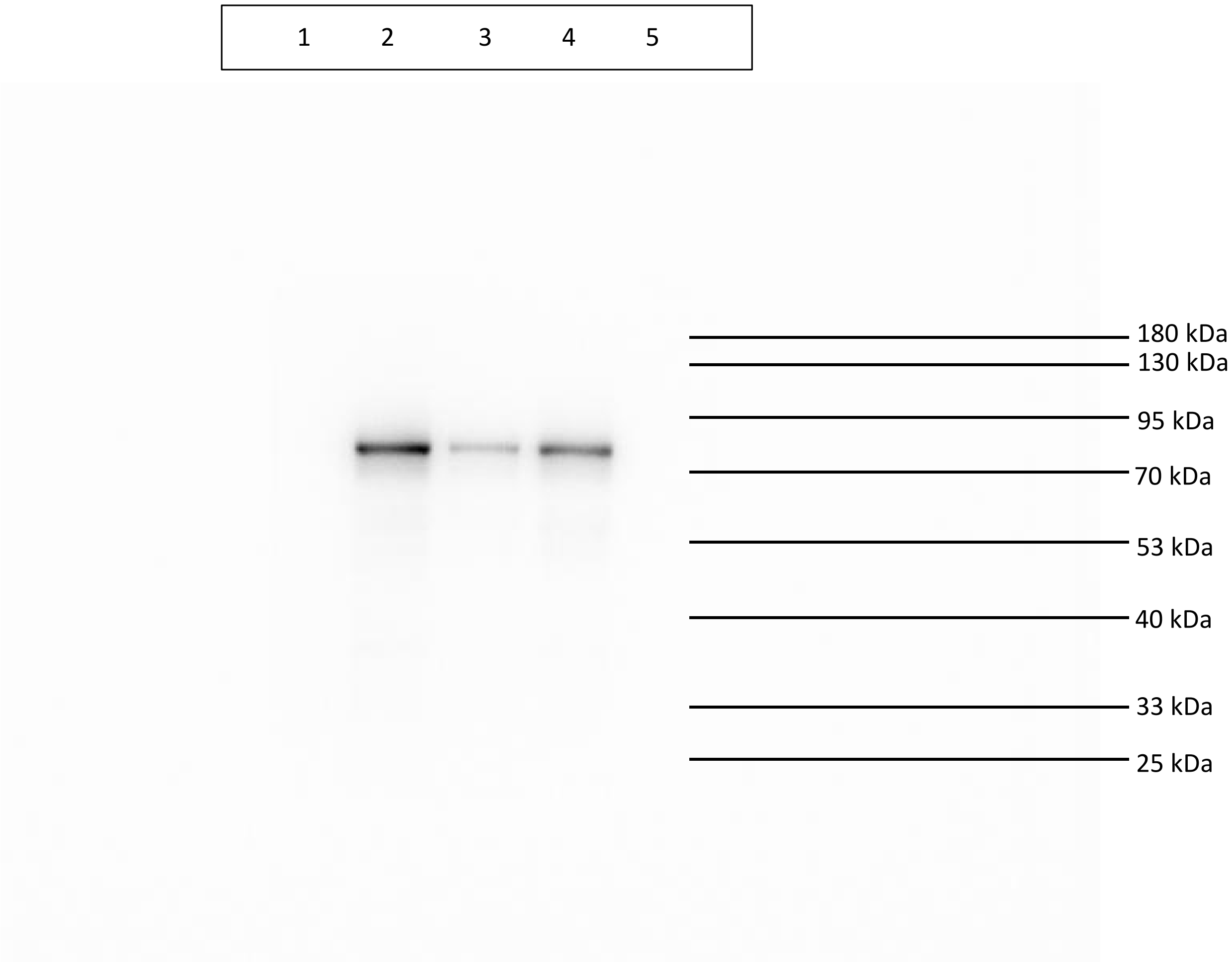

Lane1：Protein marker

Lane2：Vector

Lane3：DNMT3A

Lane4：DNMT3A+DAC

Lane5：Protein marker

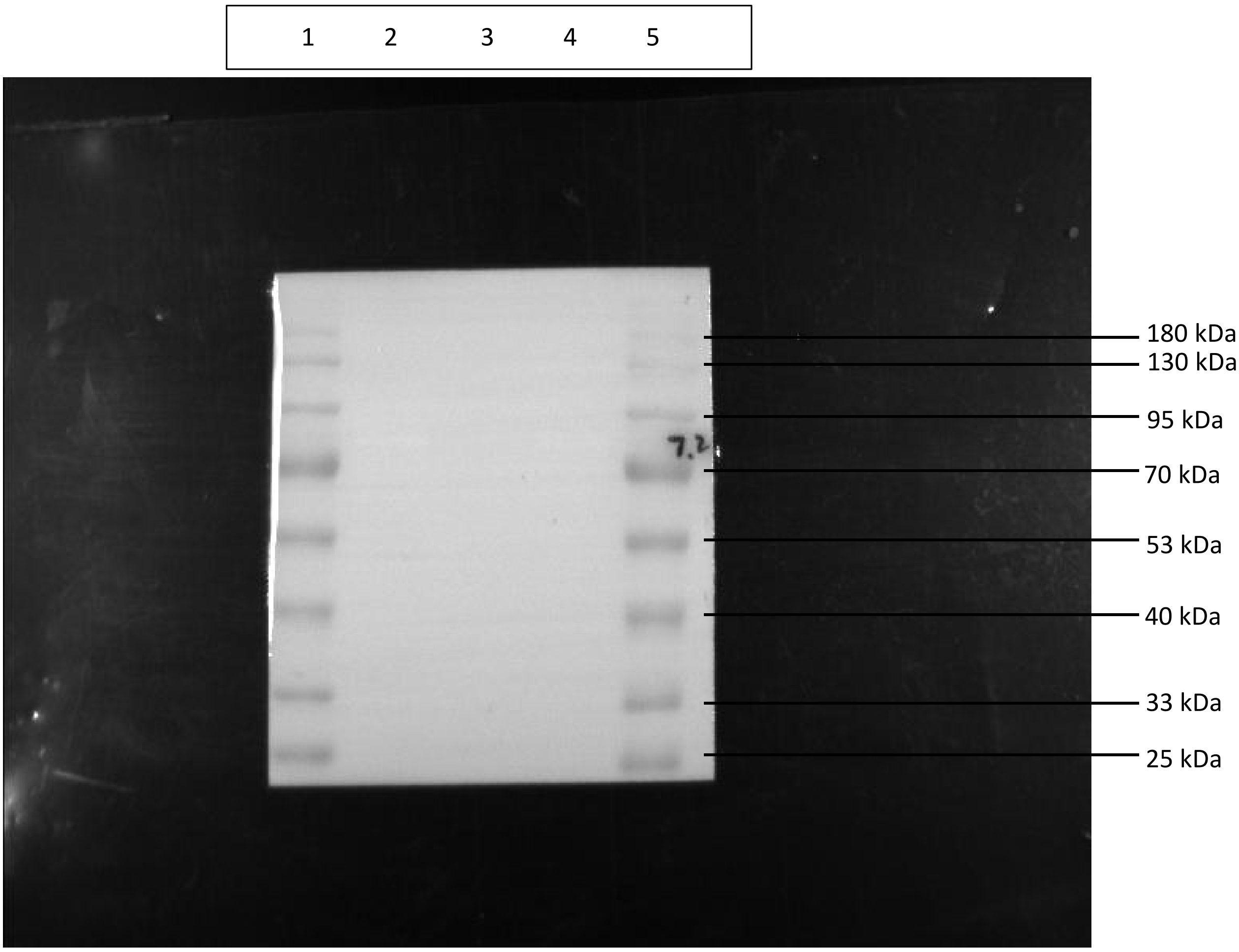

Lane1：Protein marker

Lane2：Vector

Lane3：DNMT3A

Lane4：DNMT3A+DAC

Lane5：Protein marker

Figure 6D

β-actin

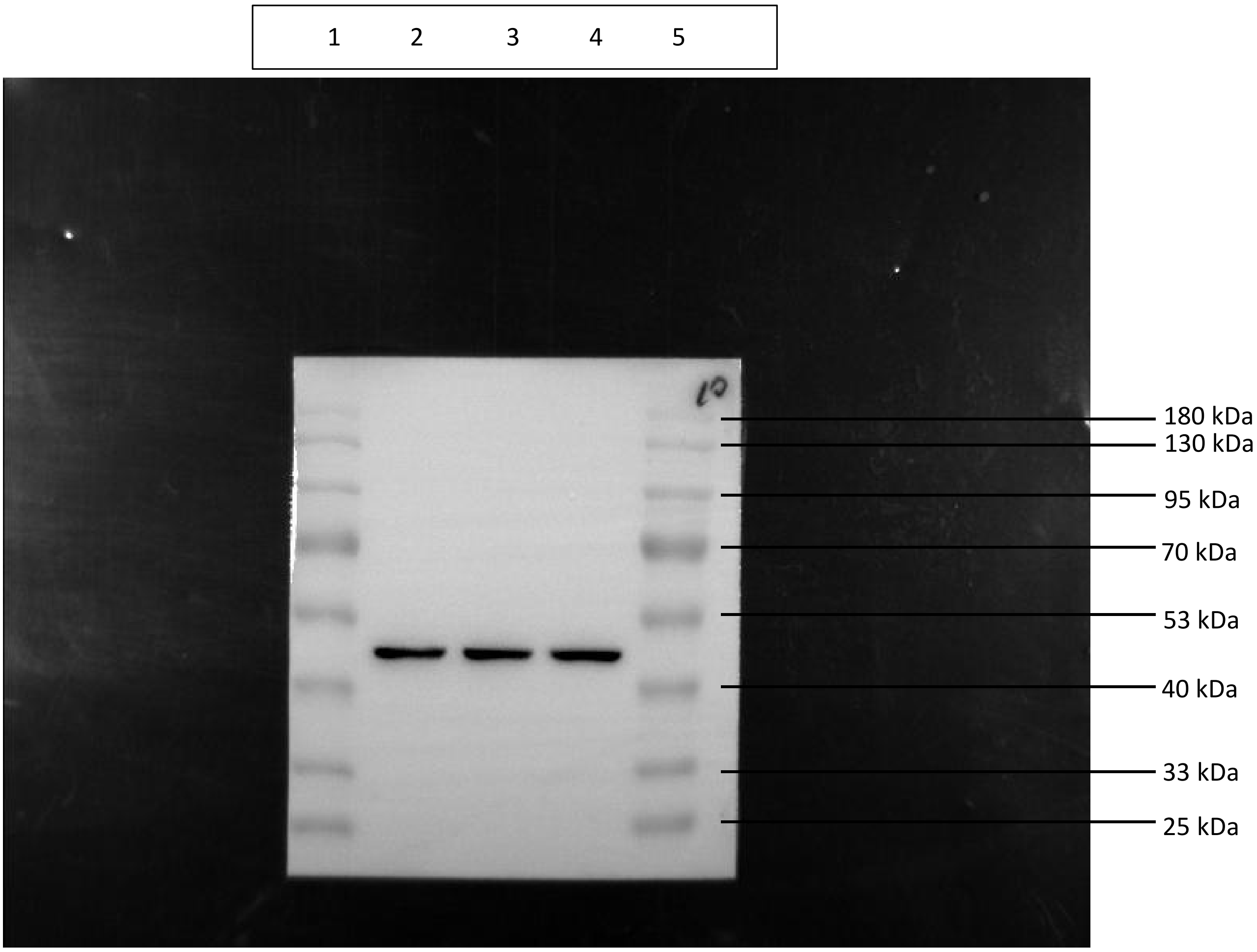

Lane1：Protein marker

Lane2：Vector

Lane3：DNMT3A

Lane4：DNMT3A+DAC

Lane5：Protein marker

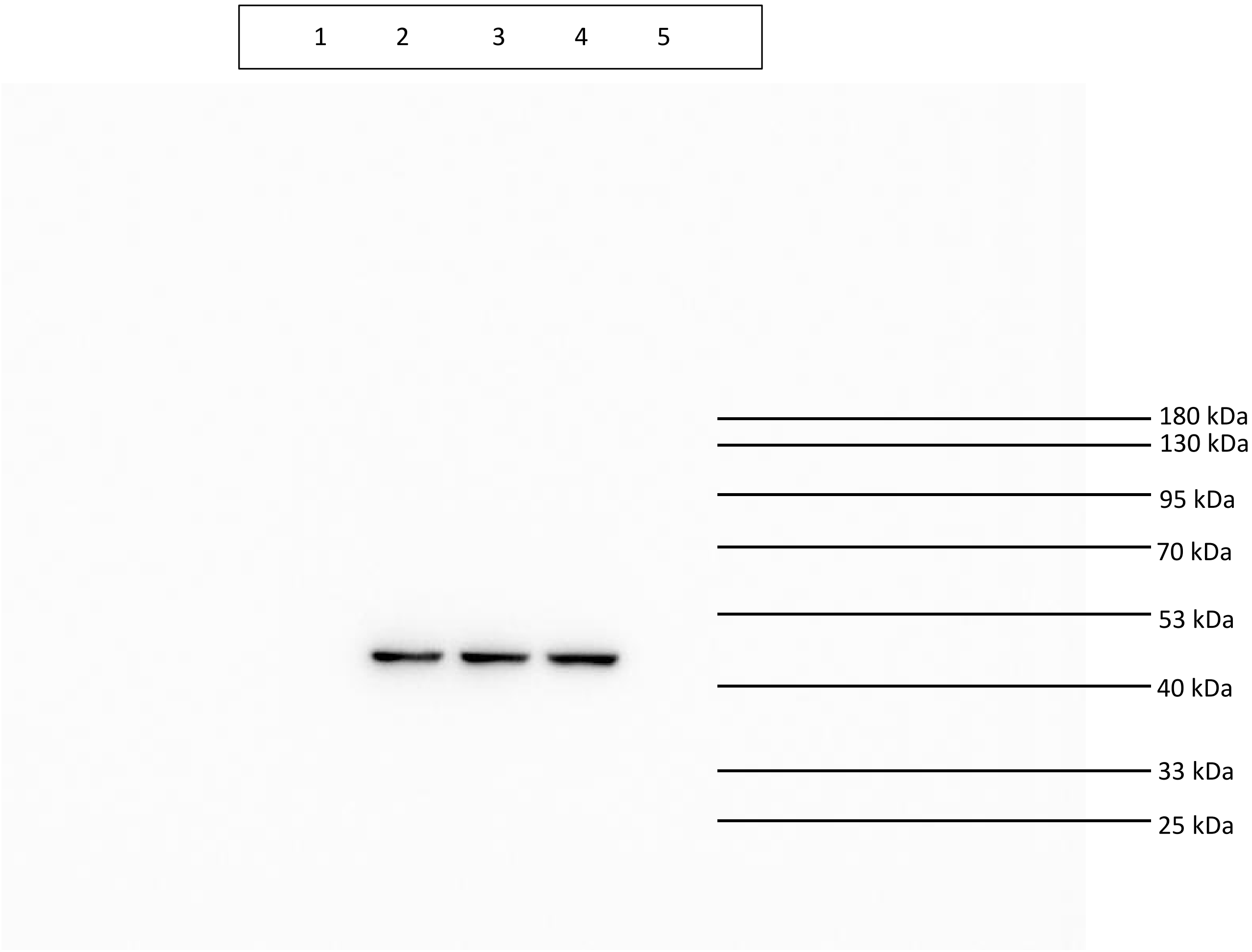

Lane1：Protein marker

Lane2：Vector

Lane3：DNMT3A

Lane4：DNMT3A+DAC

Lane5：Protein marker

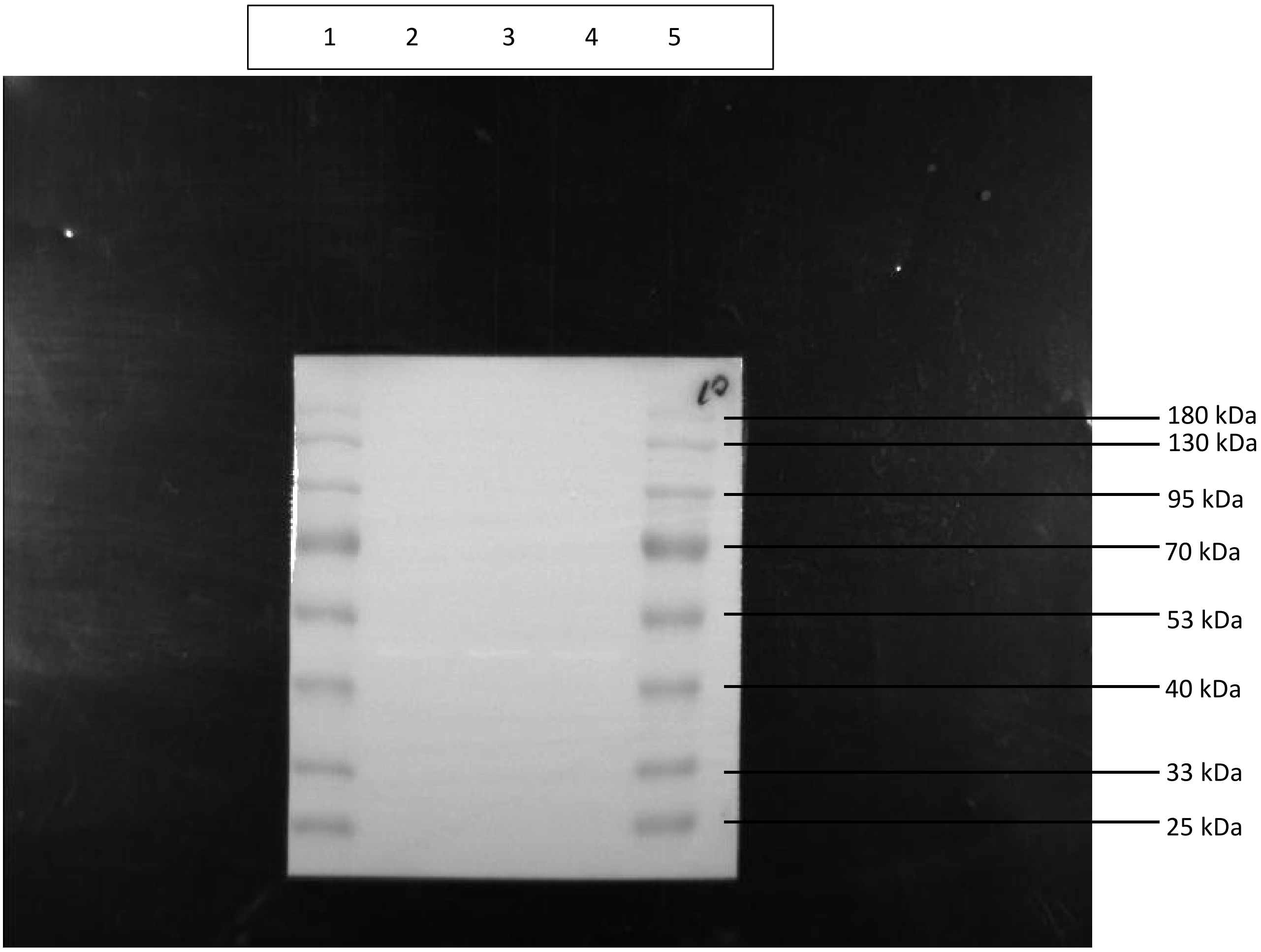

Lane1：Protein marker

Lane2：Vector

Lane3：DNMT3A

Lane4：DNMT3A+DAC

Lane5：Protein marker

Figure 6E

EGFR

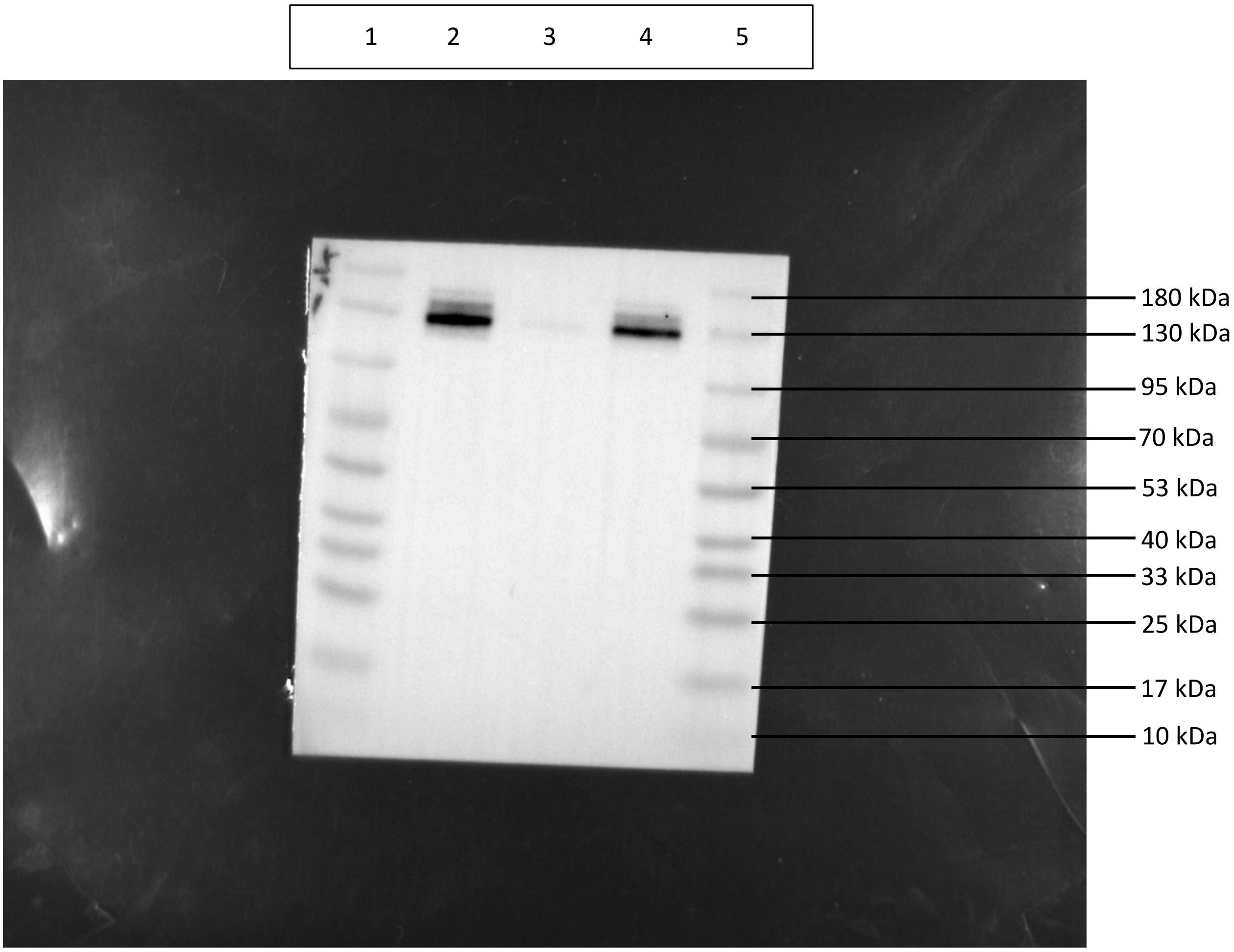

Lane1： Protein marker

Lane2： Input

Lane3： IgG

Lane4： Anti-ADAMTS8

Lane5： Protein marker

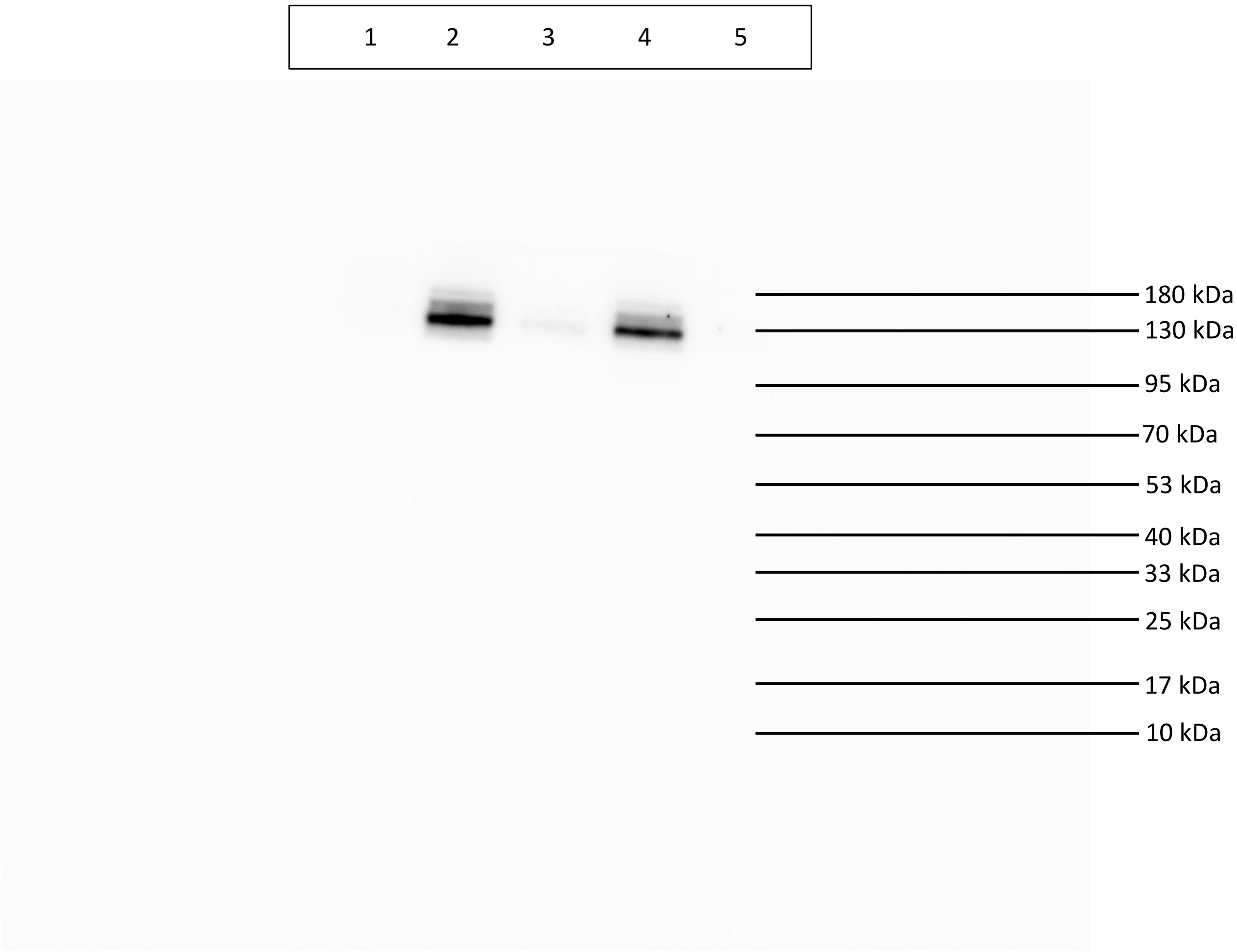

Lane1：Protein marker

Lane2：Input

Lane3：IgG

Lane4：Anti-ADAMTS8

Lane5：Protein marker

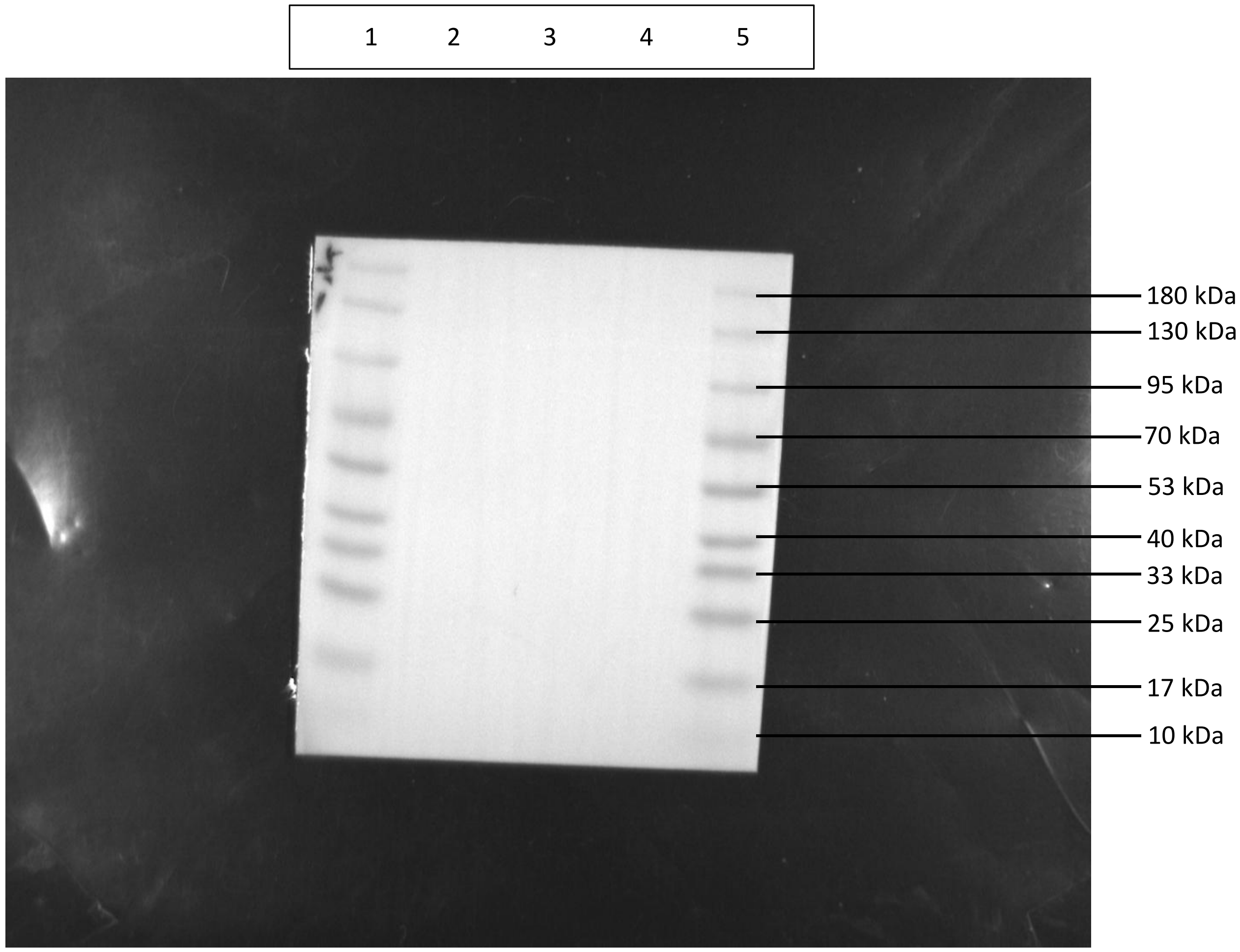

Lane1：Protein marker

Lane2：Input

Lane3：IgG

Lane4：Anti-ADAMTS8

Lane5：Protein marker

Figure 6E

ADAMTS8

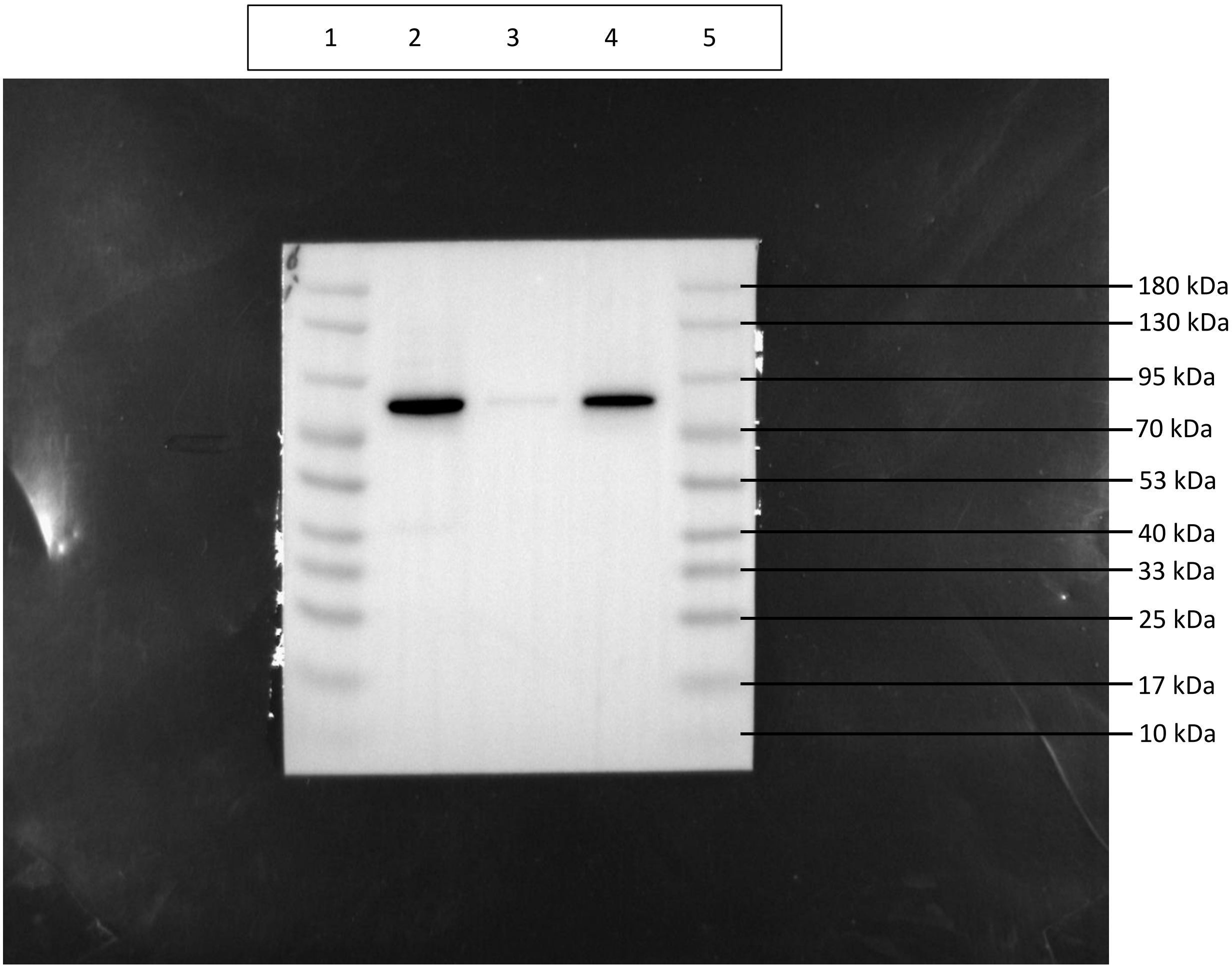

Lane1：Protein marker

Lane2：Input

Lane3：IgG

Lane4：Anti-ADAMTS8

Lane5：Protein marker

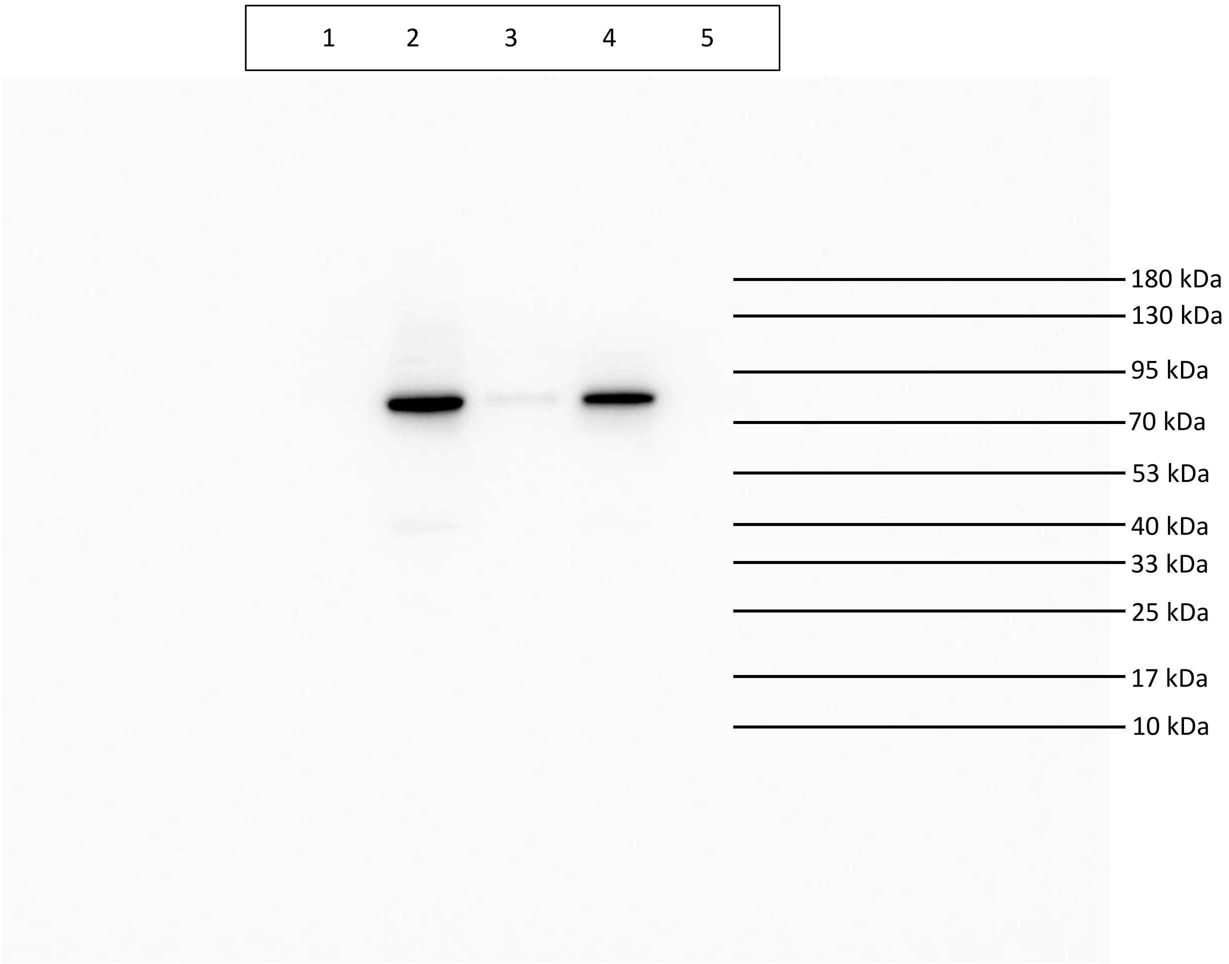

Lane1：Protein marker

Lane2：Input

Lane3：IgG

Lane4：Anti-ADAMTS8

Lane5：Protein marker

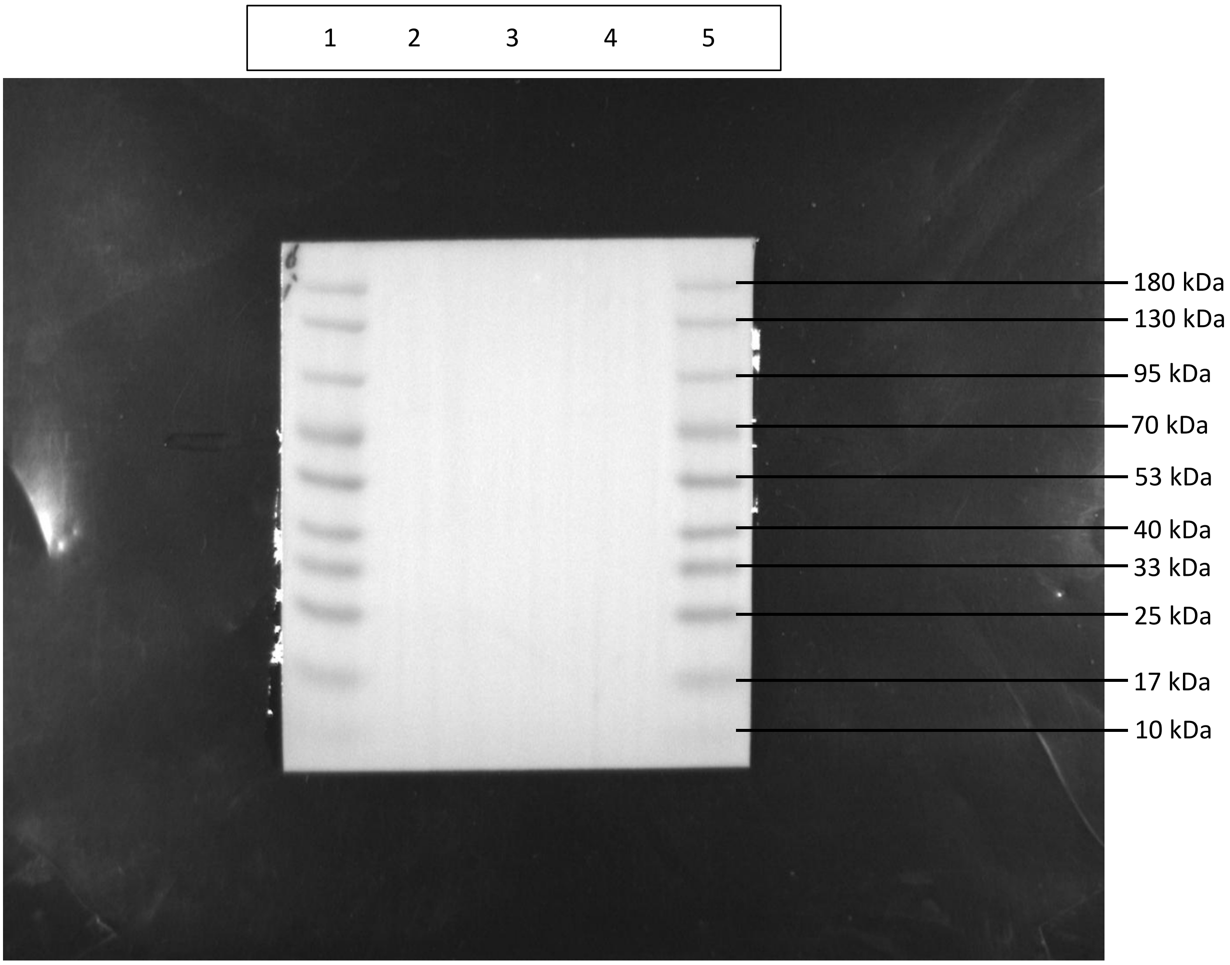

Lane1：Protein marker

Lane2：Input

Lane3：IgG

Lane4：Anti-ADAMTS8

Lane5：Protein marker

Figure 6E

EGFR

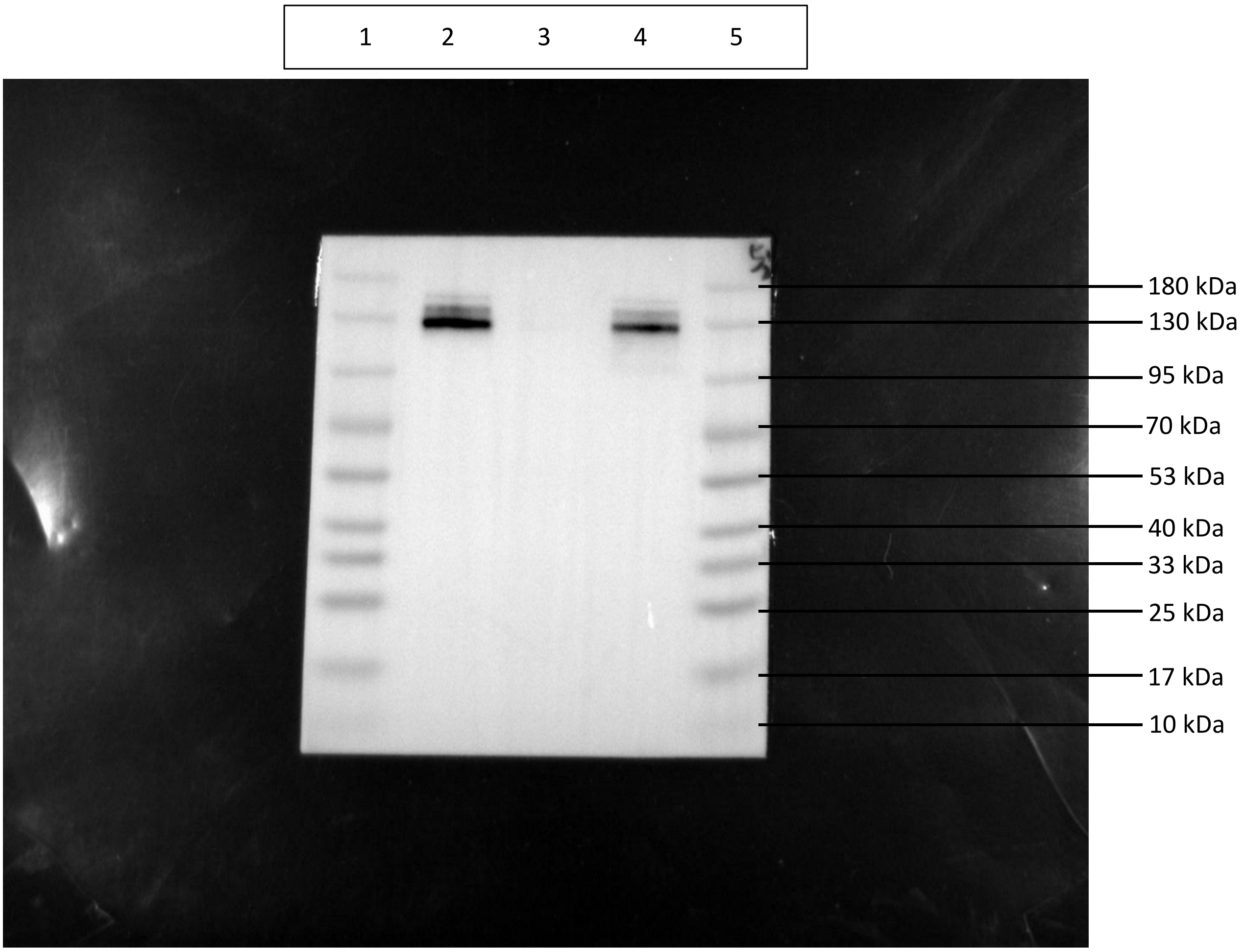

Lane1：Protein marker

Lane2：Input

Lane3：IgG

Lane4：Anti-EGFR

Lane5：Protein marker

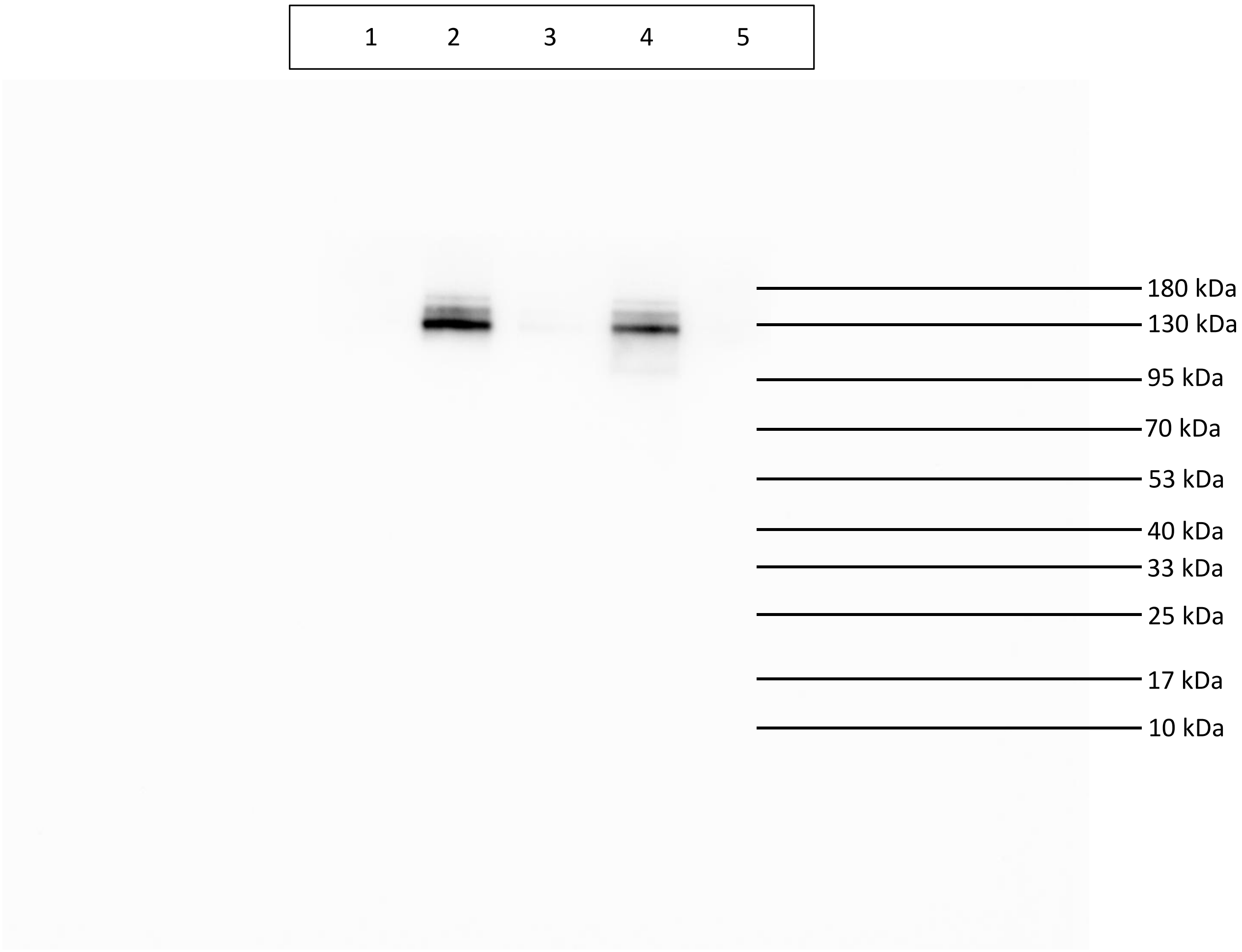

Lane1：Protein marker

Lane2：Input

Lane3：IgG

Lane4：Anti-EGFR

Lane5：Protein marker

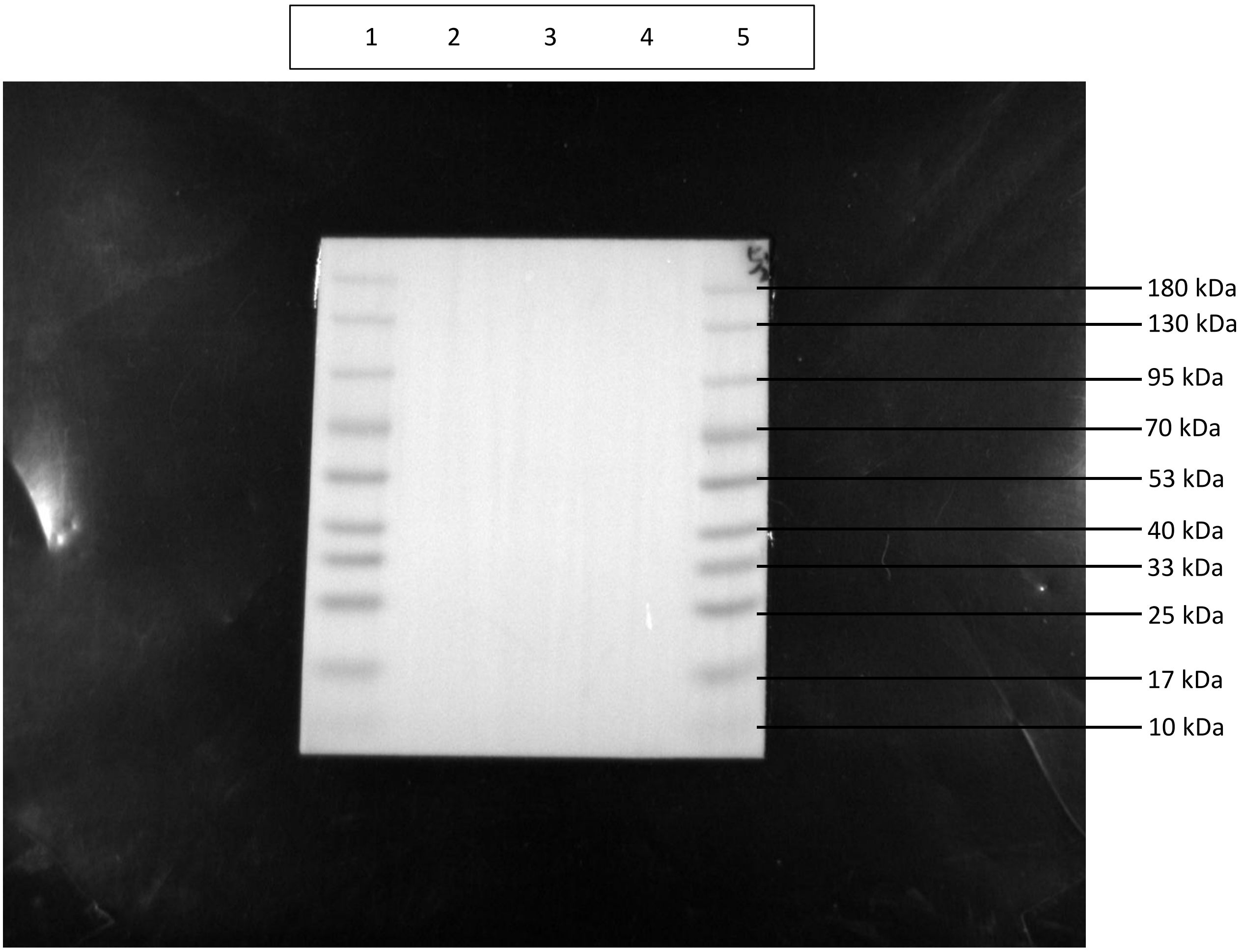

Lane1：Protein marker

Lane2：Input

Lane3：IgG

Lane4：Anti-EGFR

Lane5：Protein marker

Figure 6E

ADAMTS8

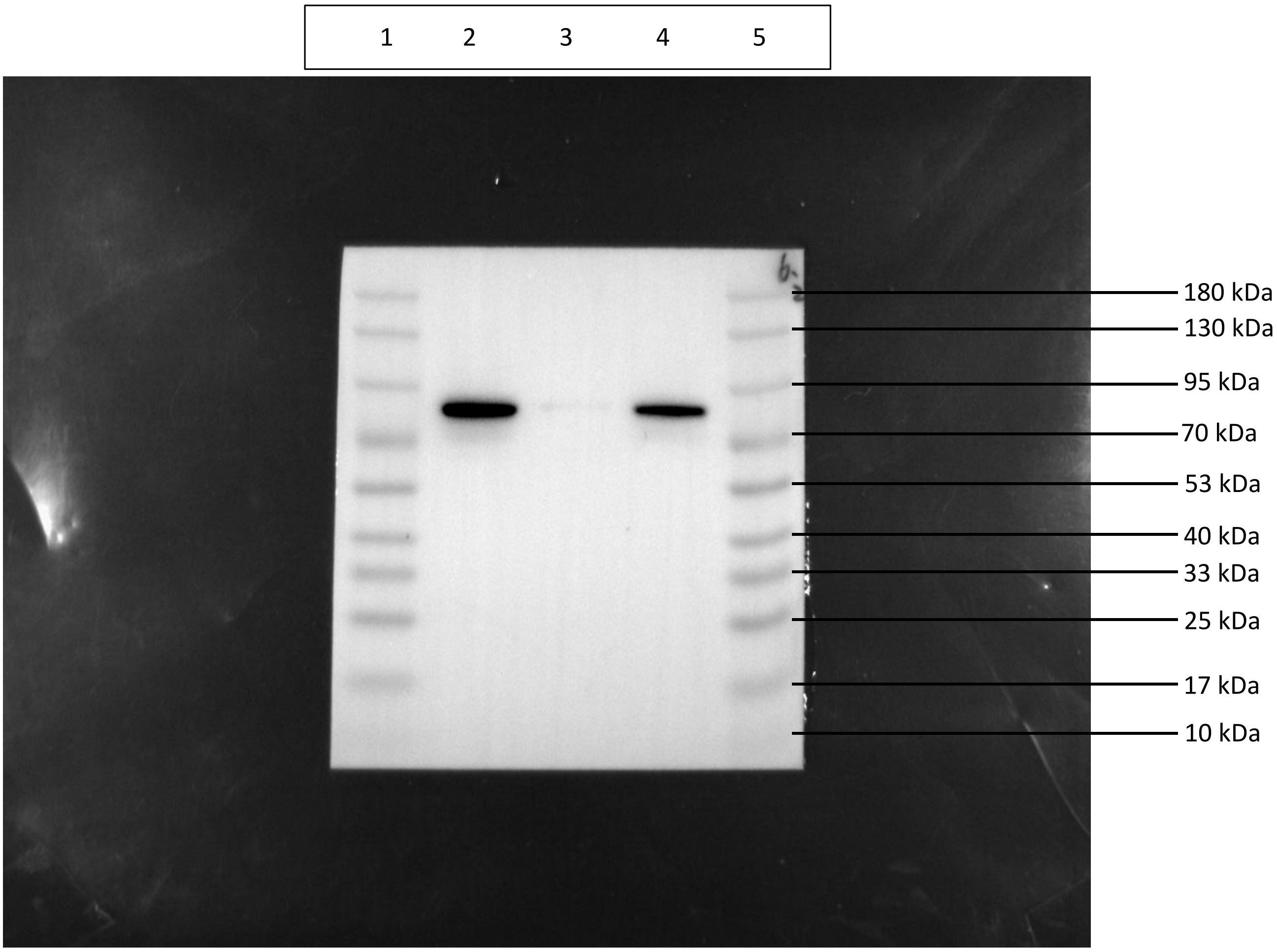

Lane1：Protein marker

Lane2：Input

Lane3：IgG

Lane4：Anti-EGFR

Lane5：Protein marker

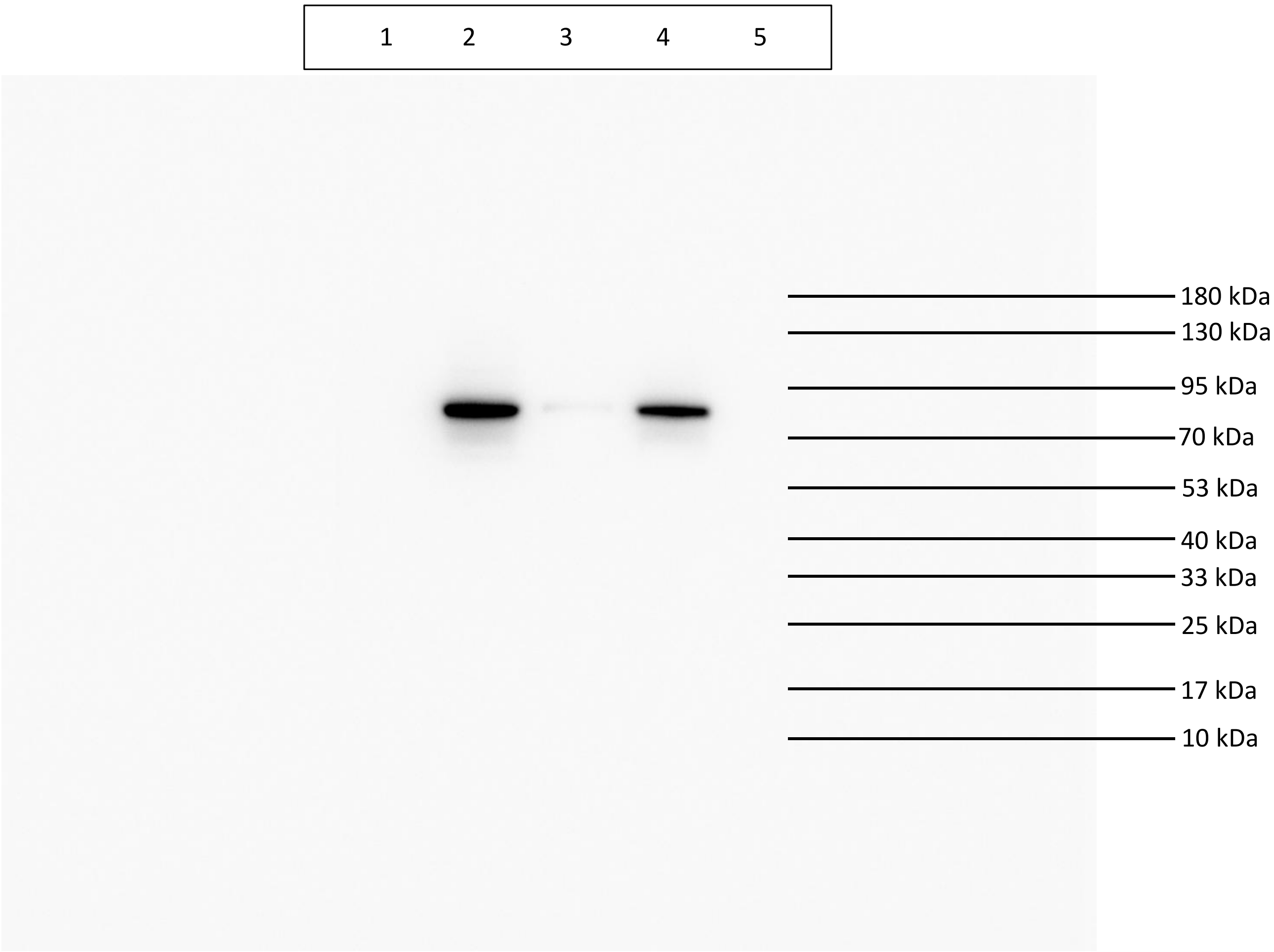

Lane1：Protein marker

Lane2：Input

Lane3：IgG

Lane4：Anti-EGFR

Lane5：Protein marker

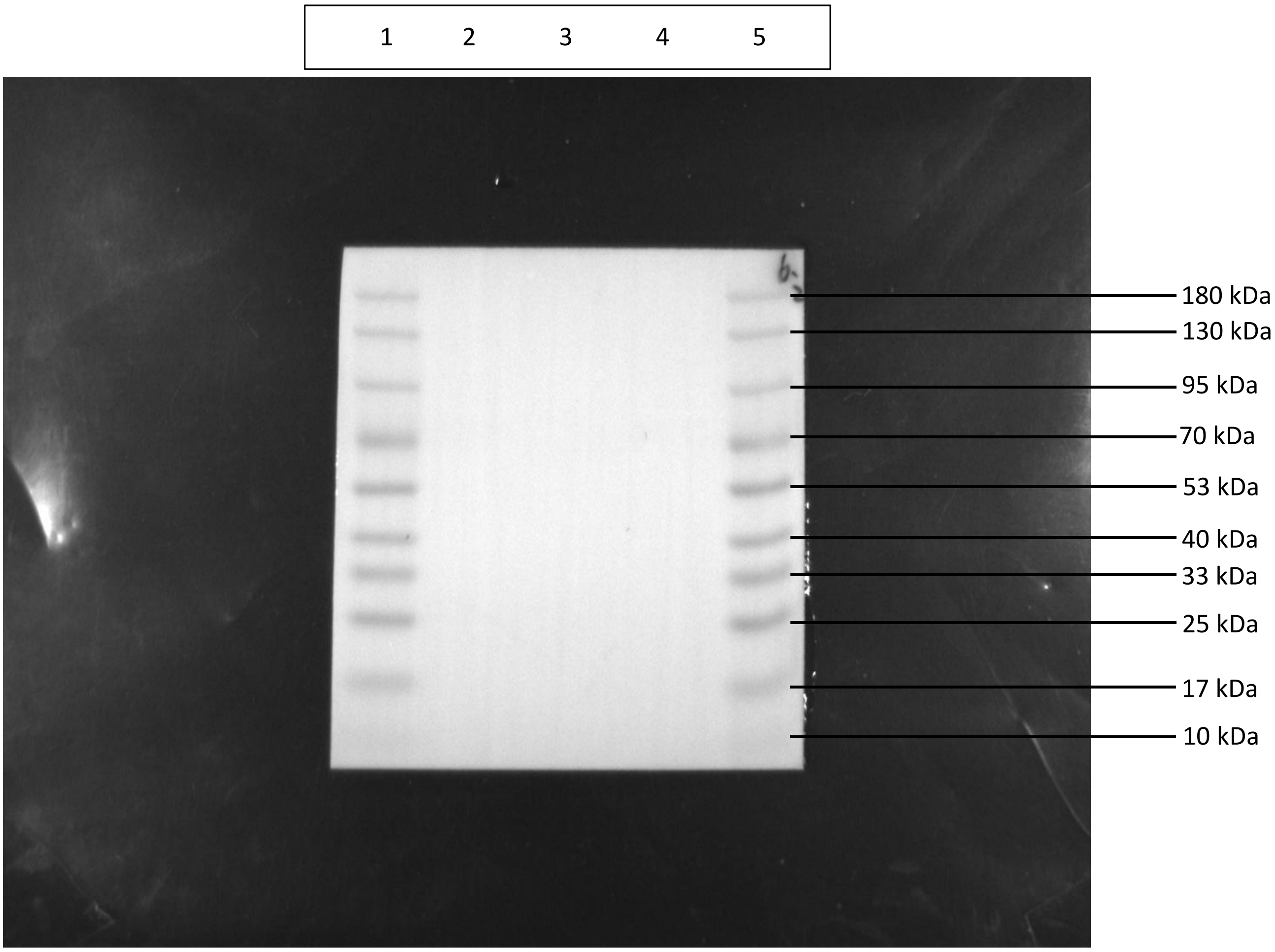

Lane1：Protein marker

Lane2：Input

Lane3：IgG

Lane4：Anti-EGFR

Lane5：Protein marker

Figure 6G

ADAMTS8

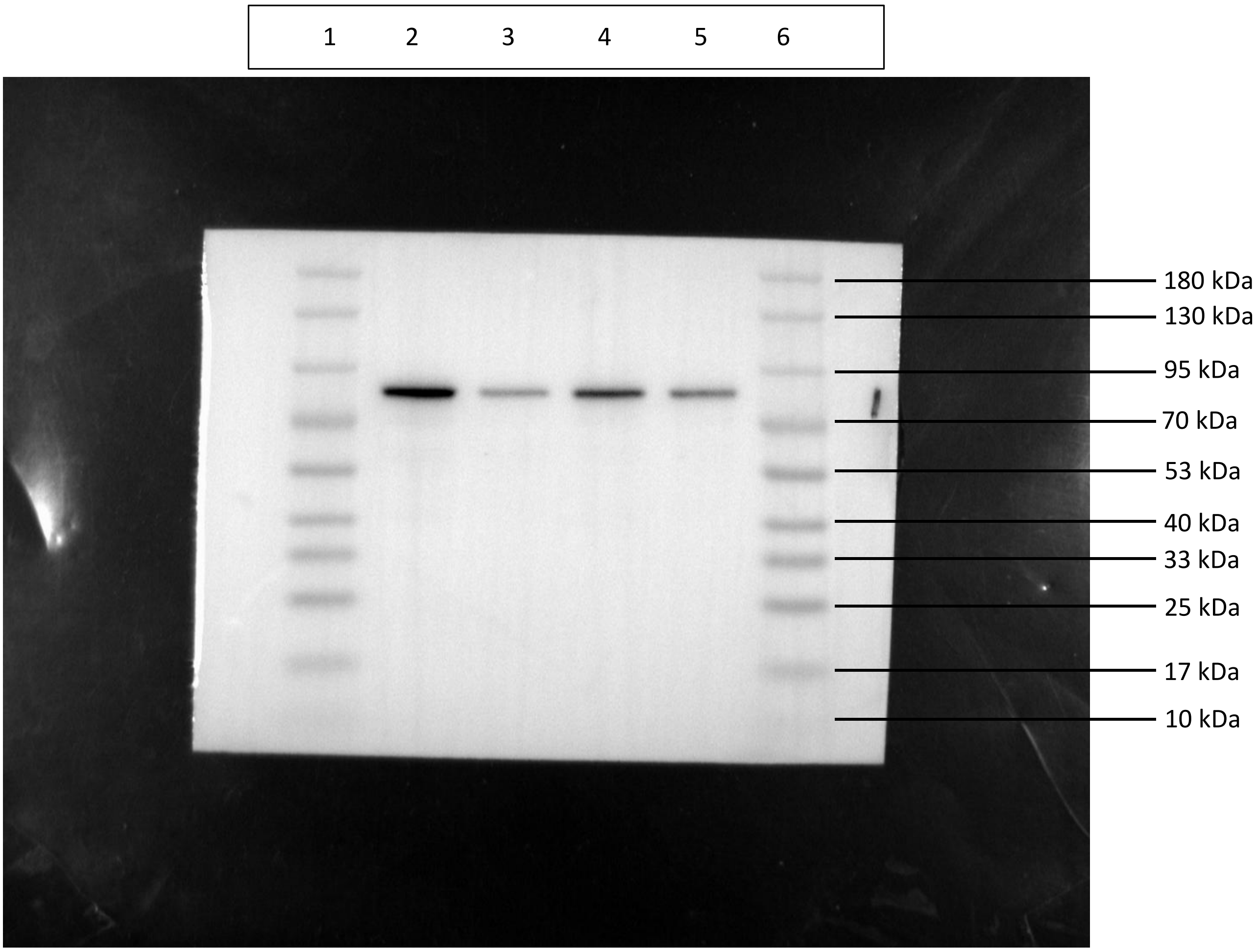

Lane1：Protein marker

Lane2：NC

Lane3：si-ADAMTS8-1

Lane4：si-ADAMTS8-2

Lane5：si-ADAMTS8-3

Lane6：Protein marker

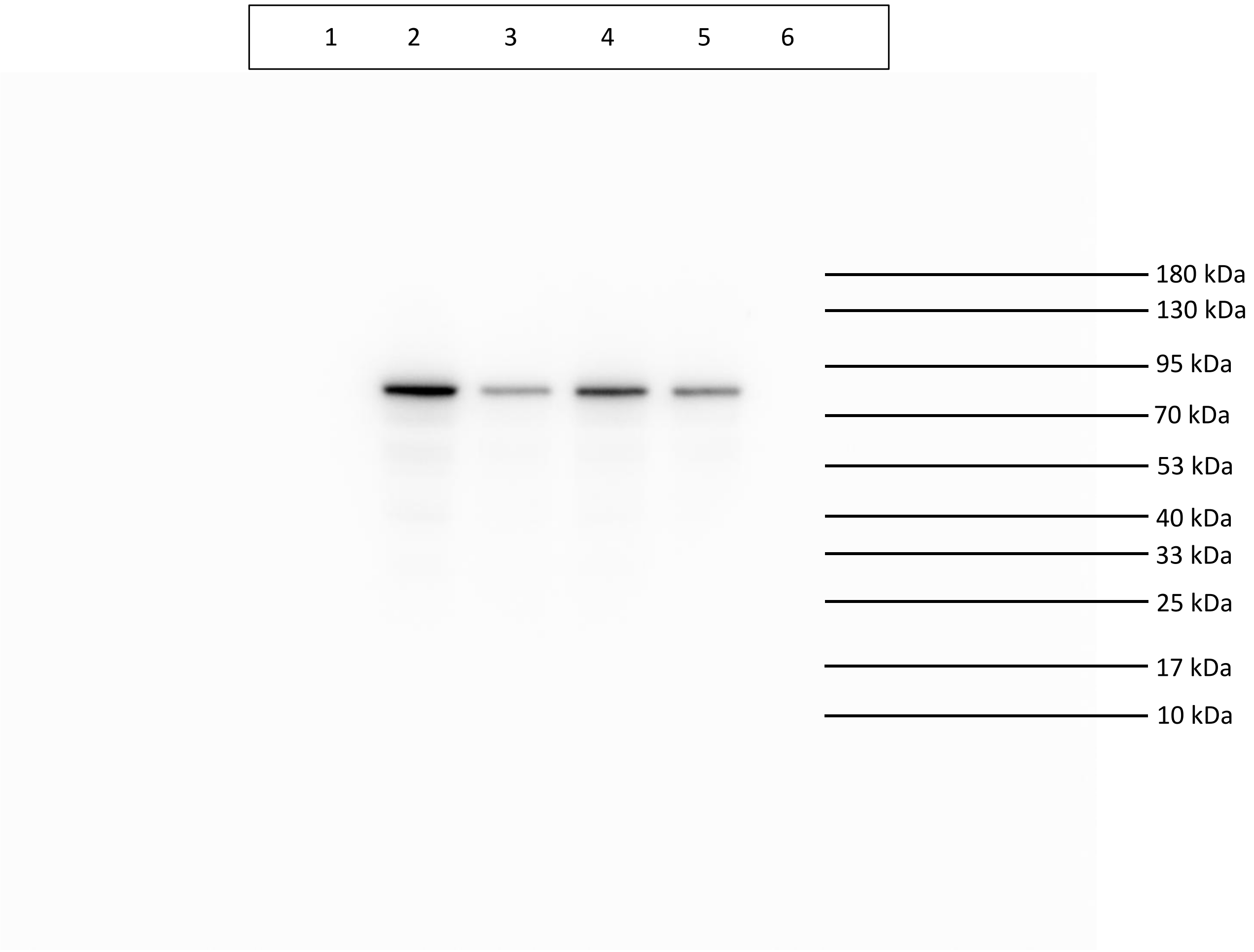

Lane1：Protein marker

Lane2：NC

Lane3：si-ADAMTS8-1

Lane4：si-ADAMTS8-2

Lane5：si-ADAMTS8-3

Lane6：Protein marker

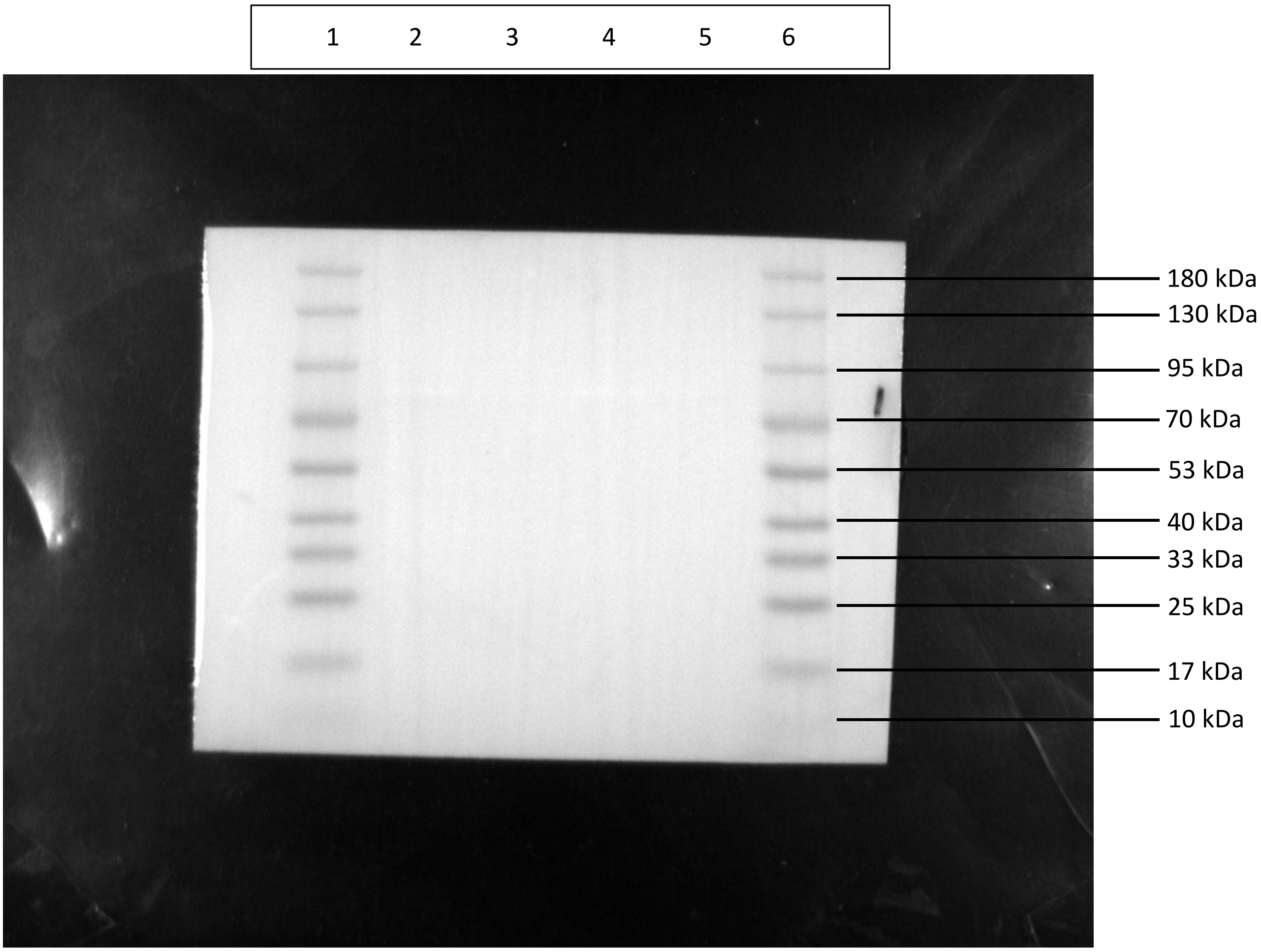

Lane1：Protein marker

Lane2：NC

Lane3：si-ADAMTS8-1

Lane4：si-ADAMTS8-2

Lane5：si-ADAMTS8-3

Lane6：Protein marker

β-actin

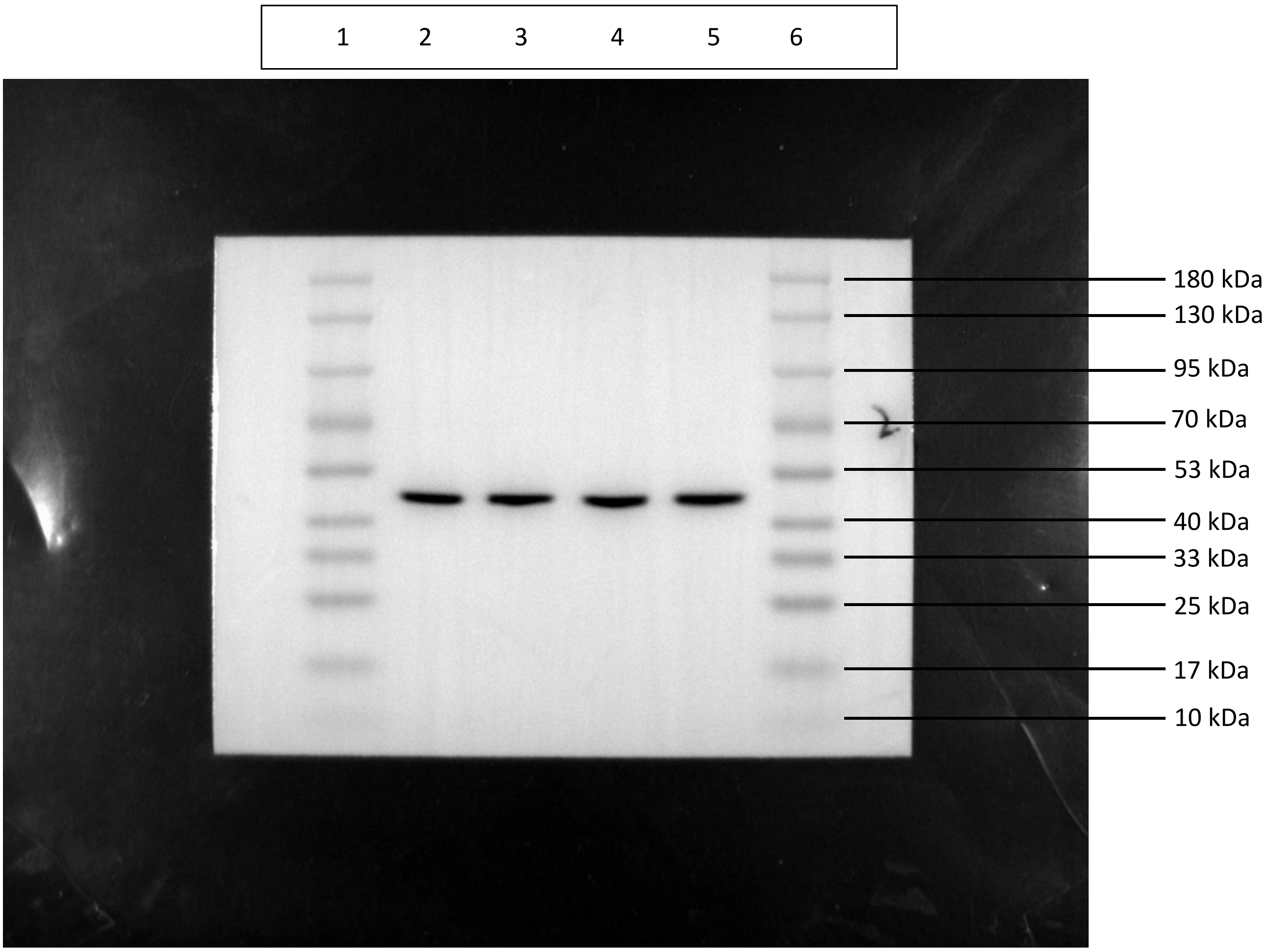

Lane1：Protein marker

Lane2：NC

Lane3：si-ADAMTS8-1

Lane4：si-ADAMTS8-2

Lane5：si-ADAMTS8-3

Lane6：Protein marker

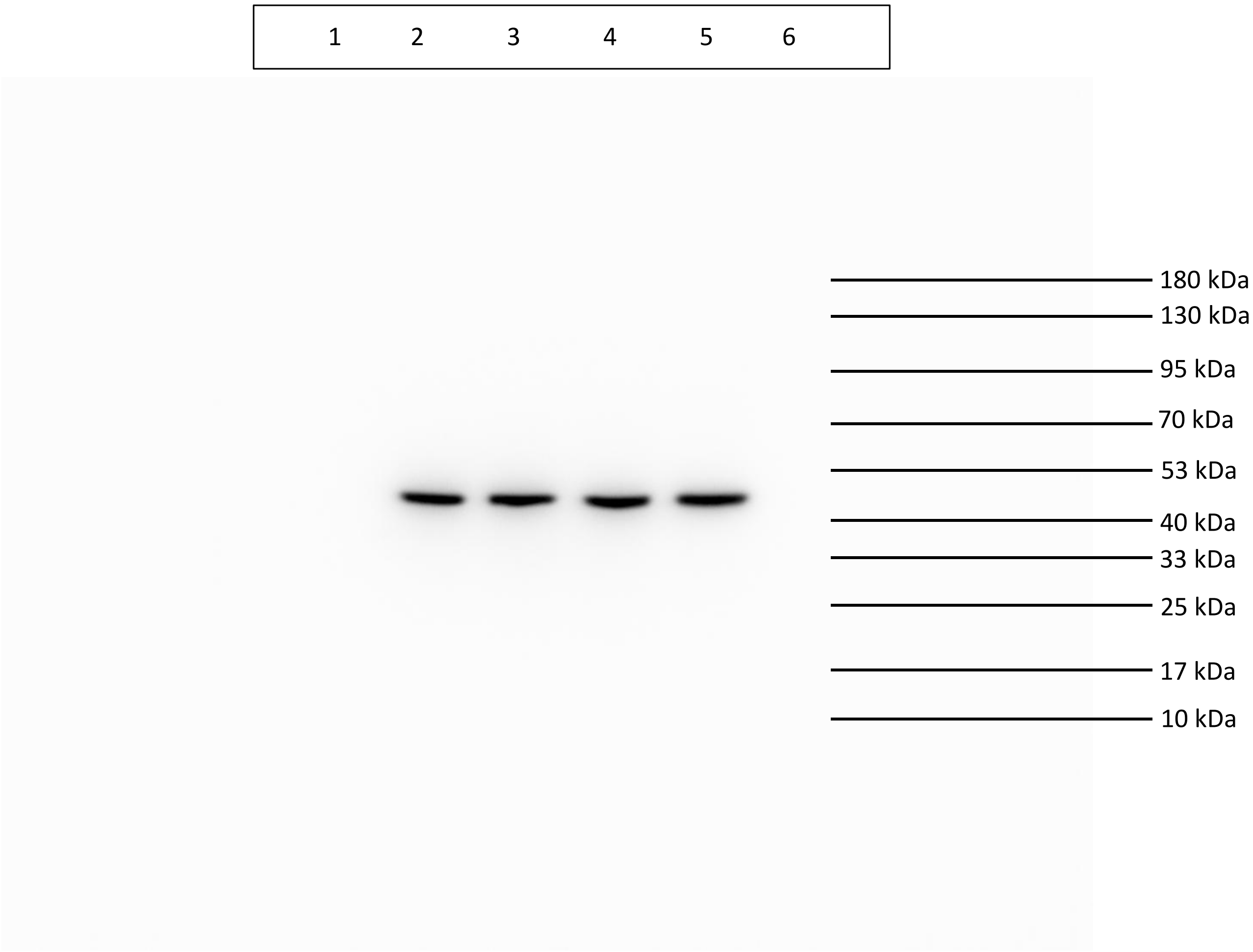

Lane1：Protein marker
Lane2：NC
Lane3：si-ADAMTS8-1
Lane4：si-ADAMTS8-2
Lane5：si-ADAMTS8-3
Lane6：Protein marker

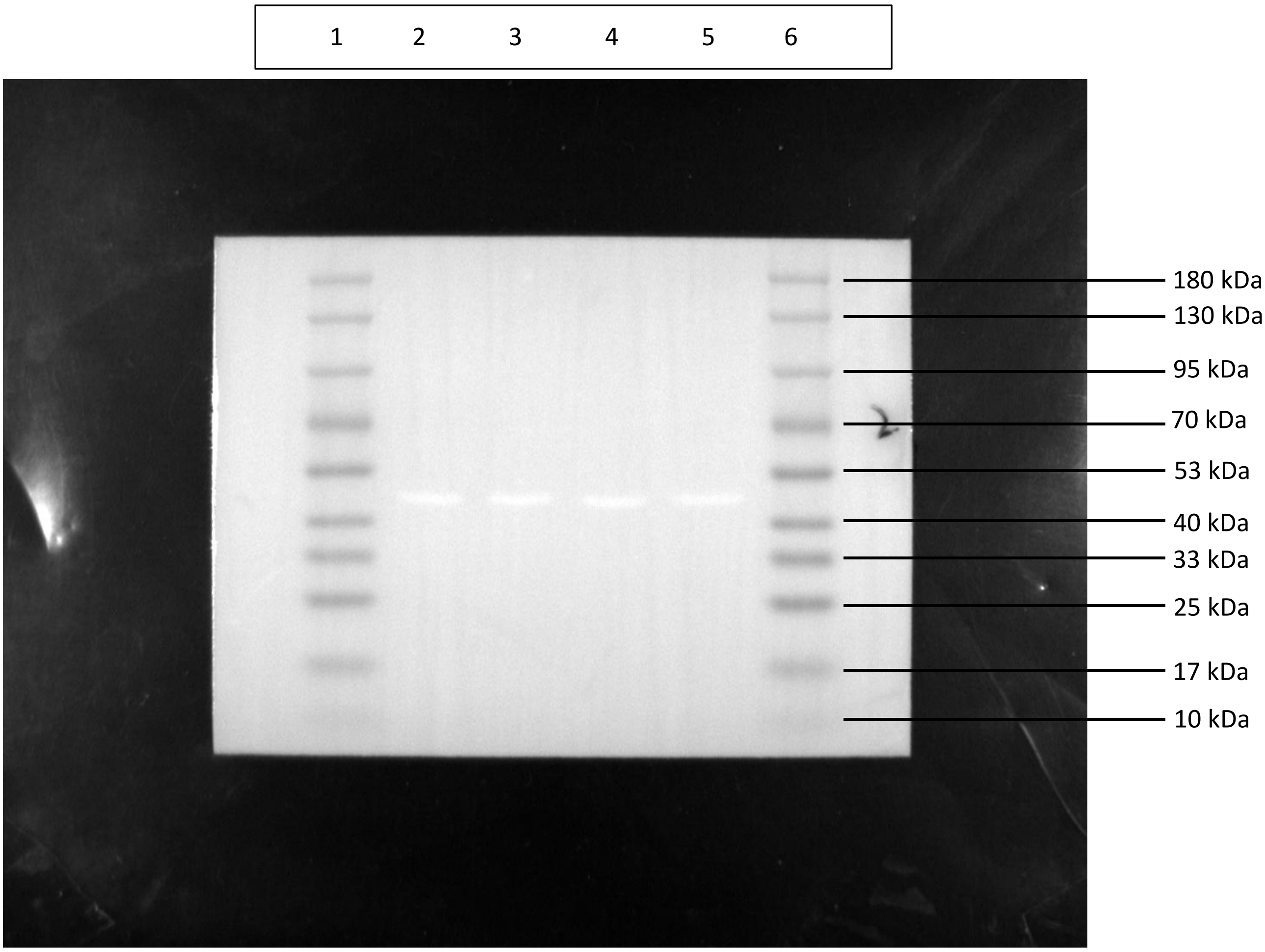

Lane1：Protein marker

Lane2：NC

Lane3：si-ADAMTS8-1

Lane4：si-ADAMTS8-2

Lane5：si-ADAMTS8-3

Lane6：Protein marker

Figure 6H

EGFR

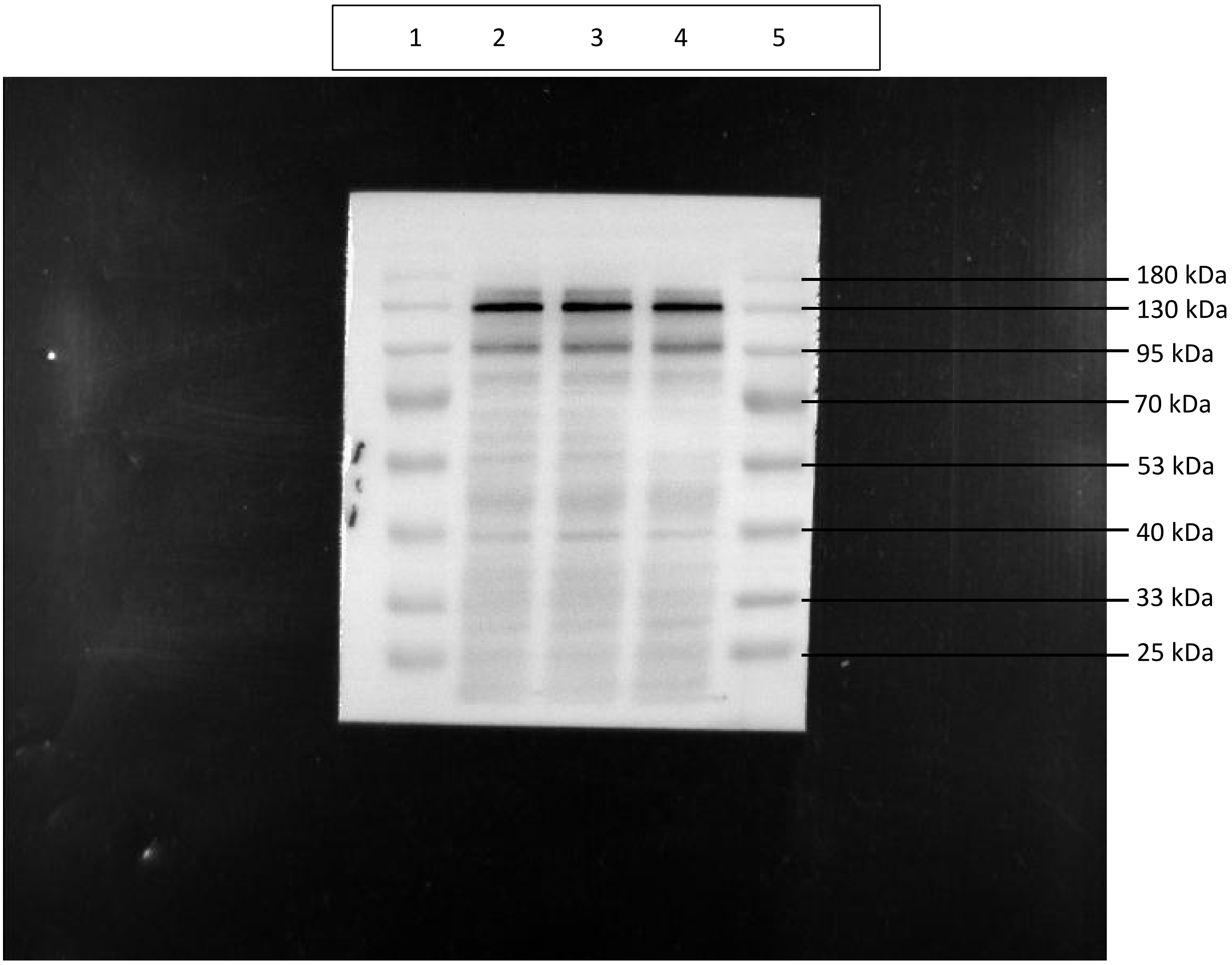

Lane1：Protein marker

Lane2：si-NC

Lane3：si-DNMT3A

Lane4：si-ADAMTS8

Lane5：Protein marker

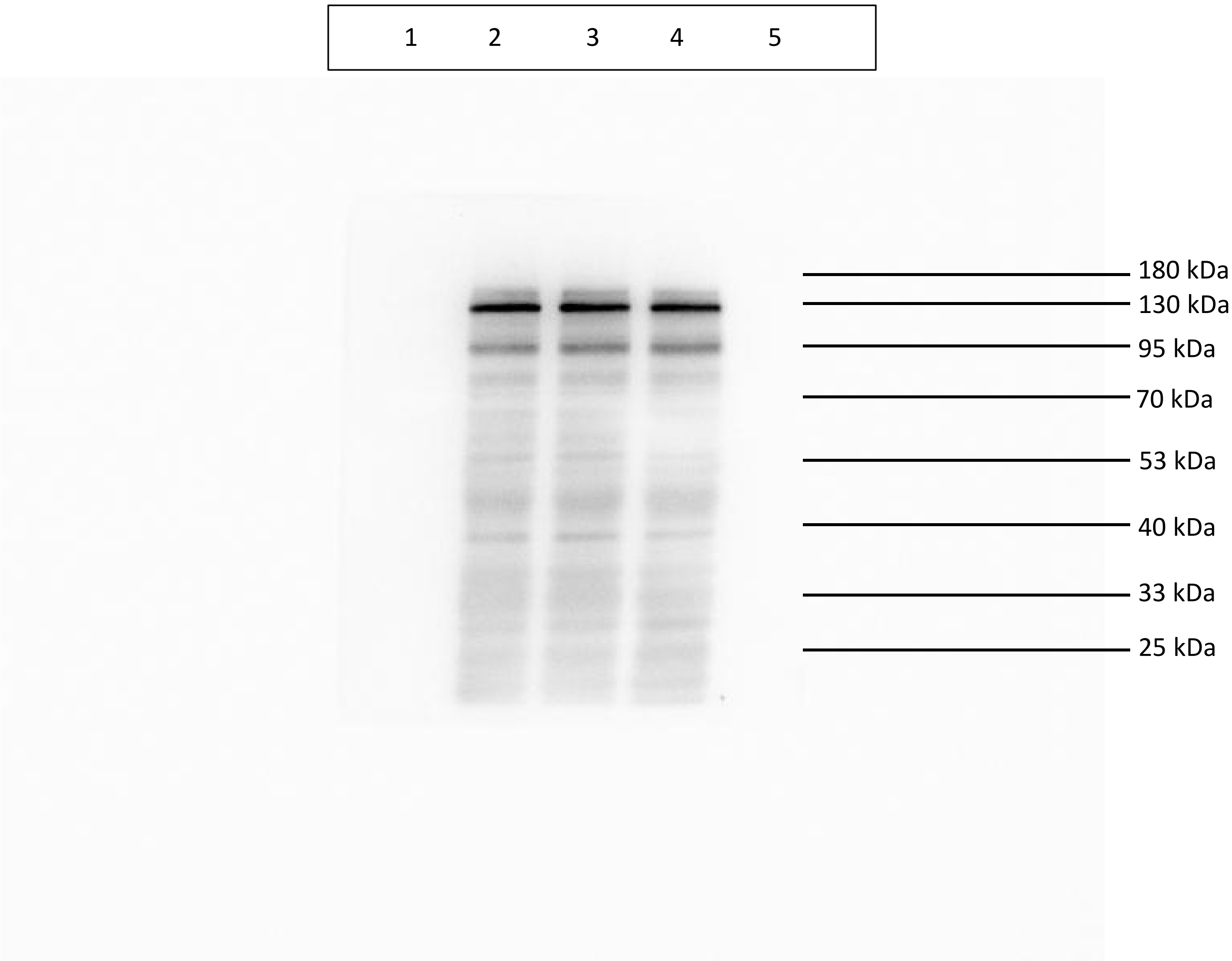

Lane1：Protein marker

Lane2：si-NC

Lane3：si-DNMT3A

Lane4：si-ADAMTS8

Lane5：Protein marker

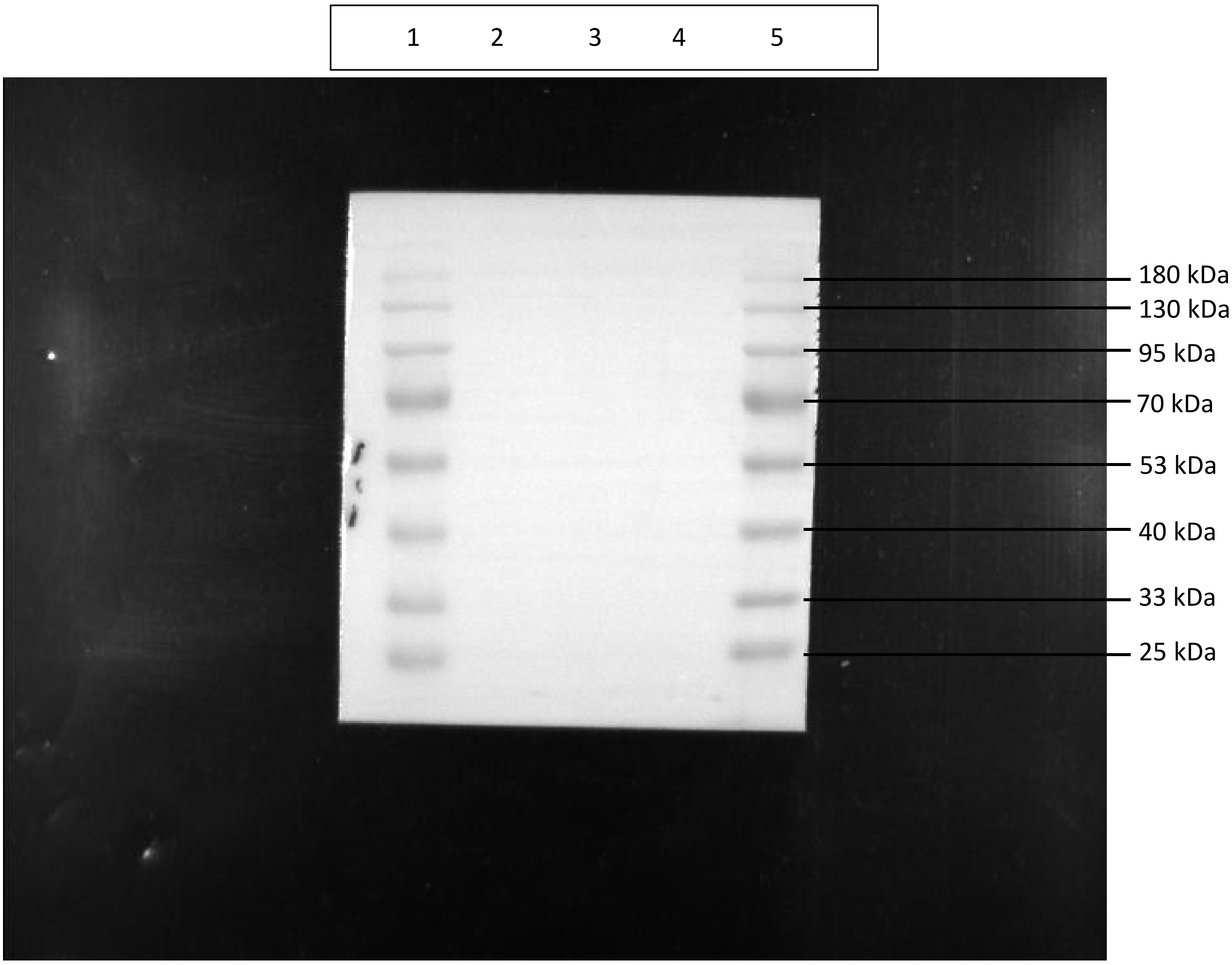

Lane1：Protein marker

Lane2：si-NC

Lane3：si-DNMT3A

Lane4：si-ADAMTS8

Lane5：Protein marker

Figure 6H

p-EGFR

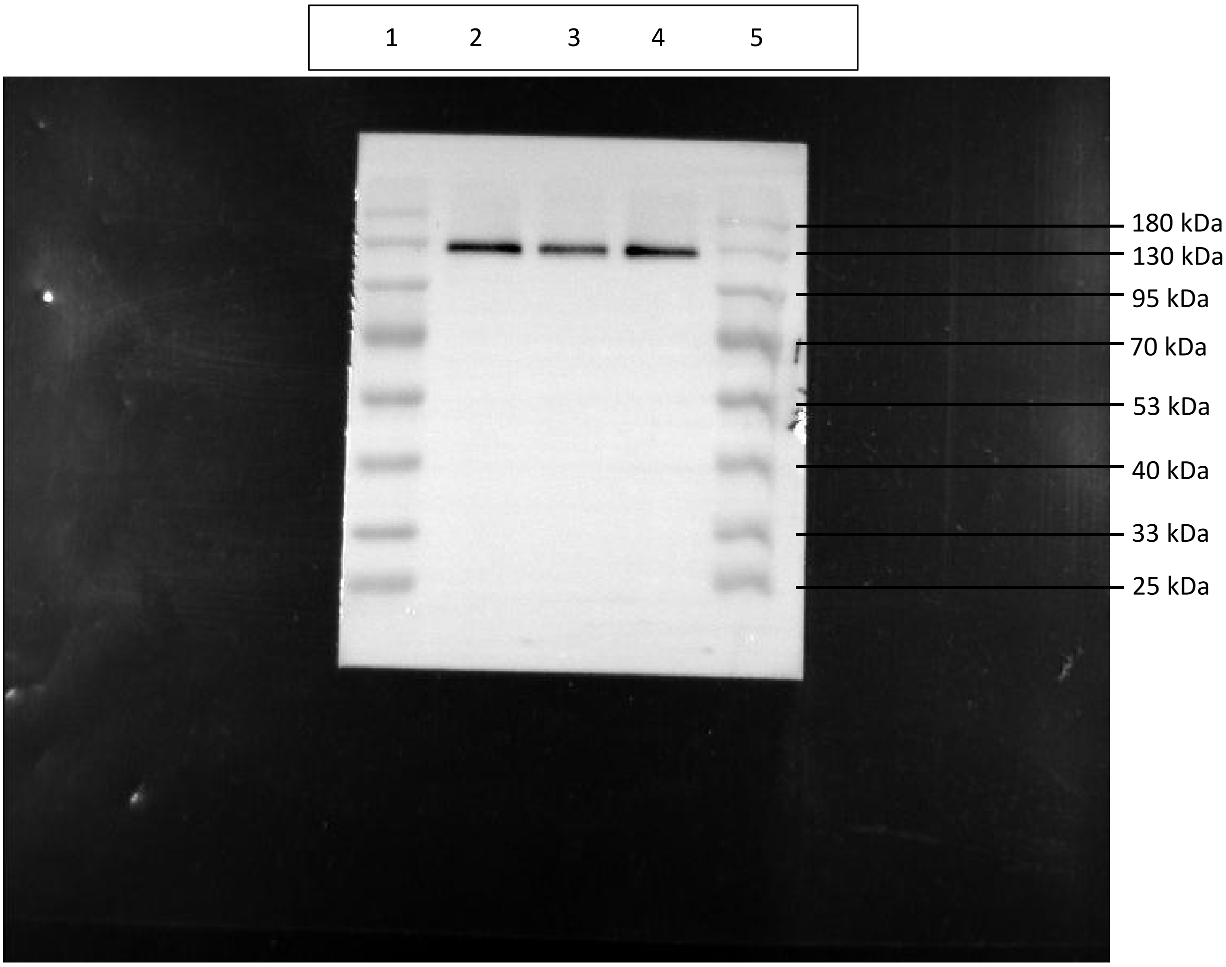

Lane1：Protein marker

Lane2：si-NC

Lane3：si-DNMT3A

Lane4：si-ADAMTS8

Lane5：Protein marker

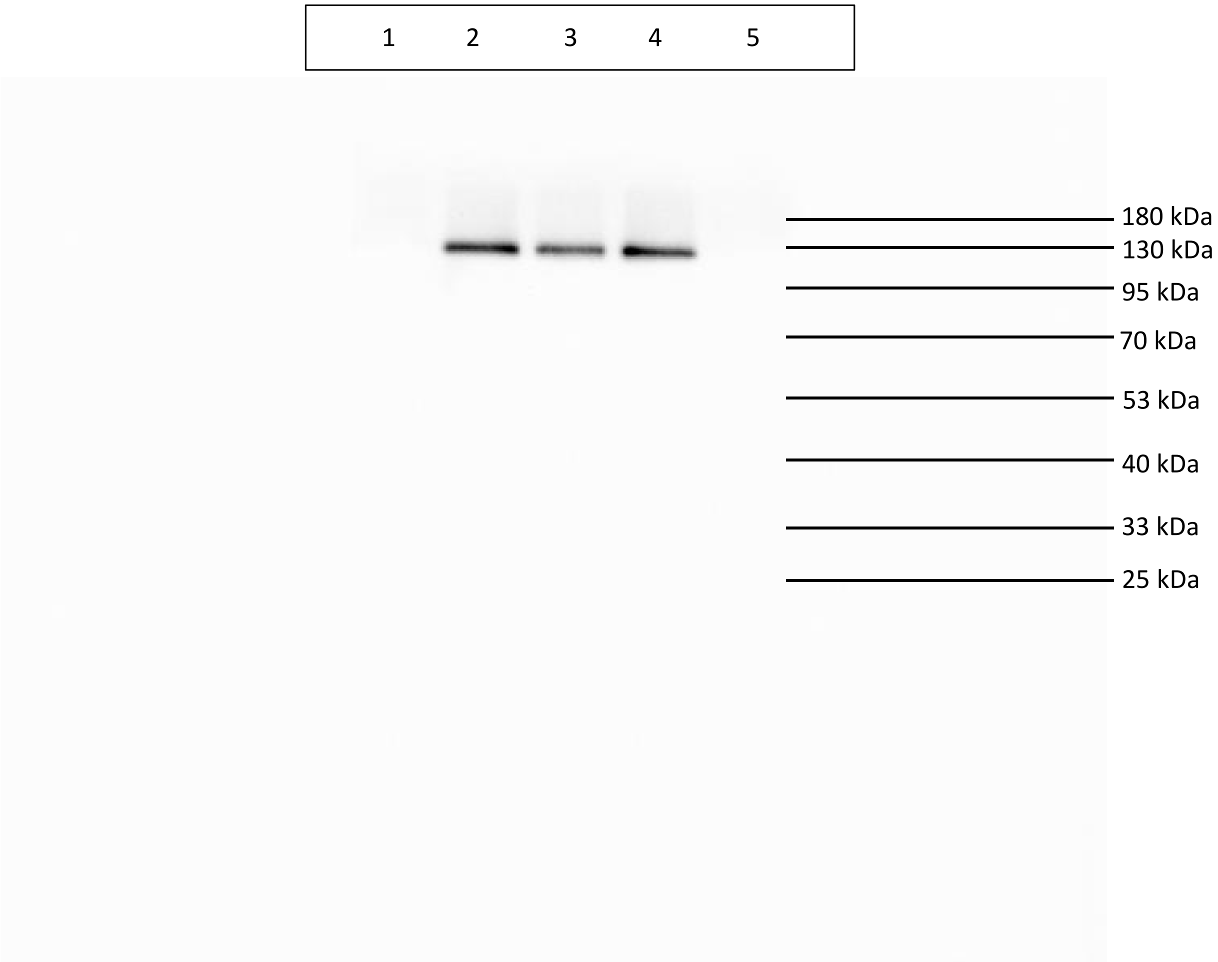

Lane1：Protein marker

Lane2：si-NC

Lane3：si-DNMT3A

Lane4：si-ADAMTS8

Lane5：Protein marker

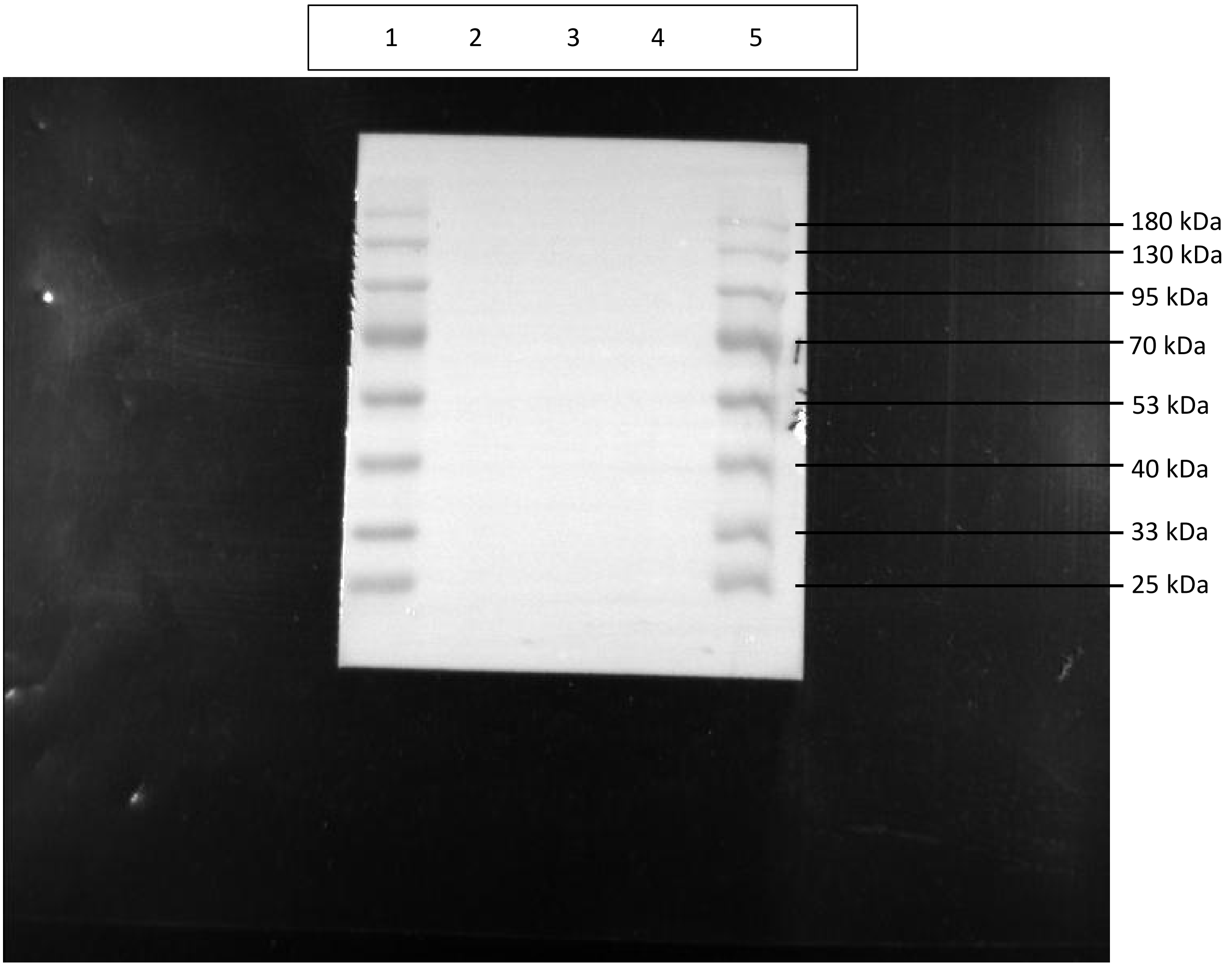

Lane1：Protein marker

Lane2：si-NC

Lane3：si-DNMT3A

Lane4：si-ADAMTS8

Lane5：Protein marker

Figure 6H

ERK1/2

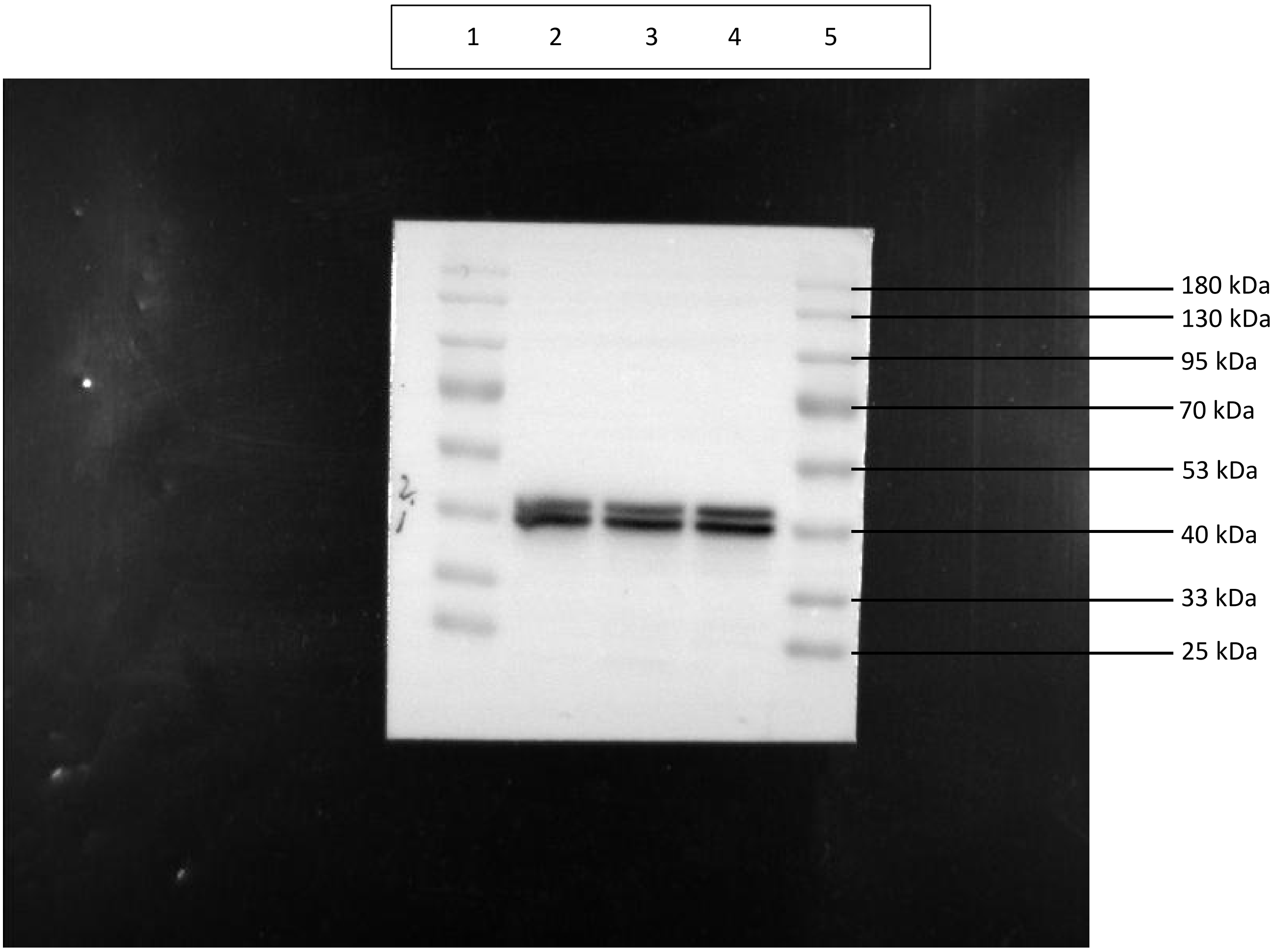

Lane1：Protein marker

Lane2：si-NC

Lane3：si-DNMT3A

Lane4：si-ADAMTS8

Lane5：Protein marker

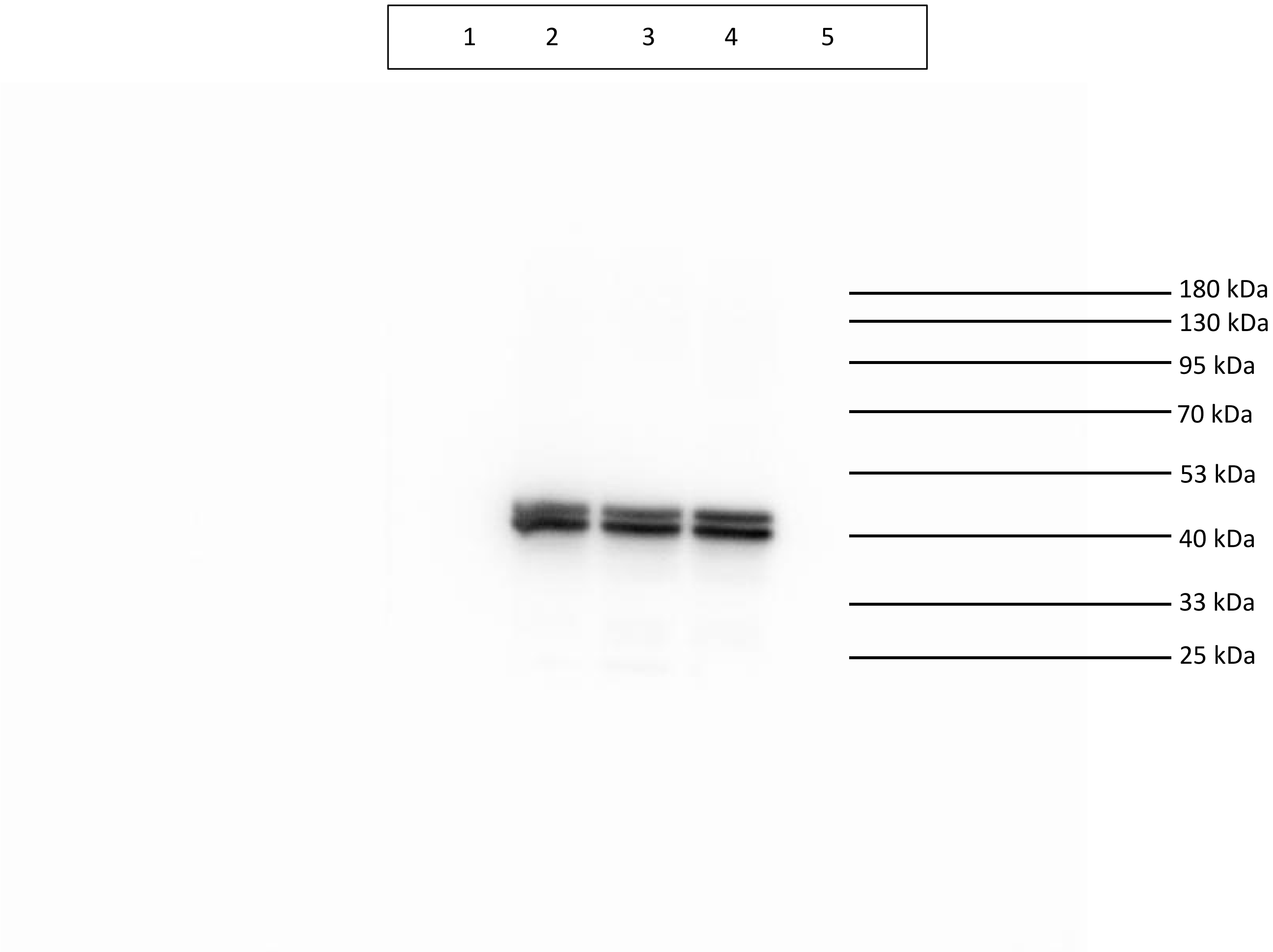

Lane1：Protein marker

Lane2：si-NC

Lane3：si-DNMT3A

Lane4：si-ADAMTS8

Lane5：Protein marker

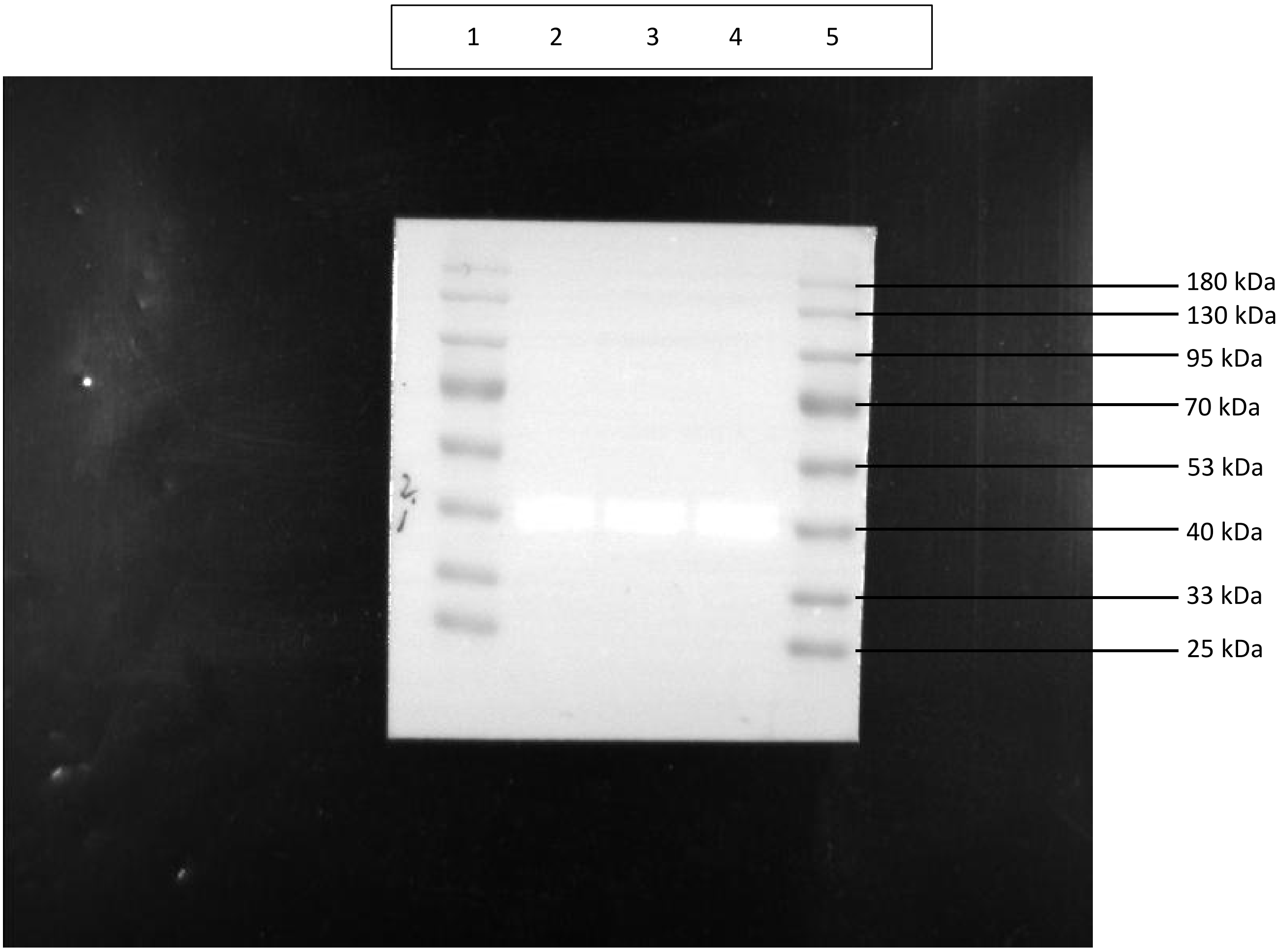

Lane1：Protein marker

Lane2：si-NC

Lane3：si-DNMT3A

Lane4：si-ADAMTS8

Lane5：Protein marker

Figure 6H

p-ERK1/2

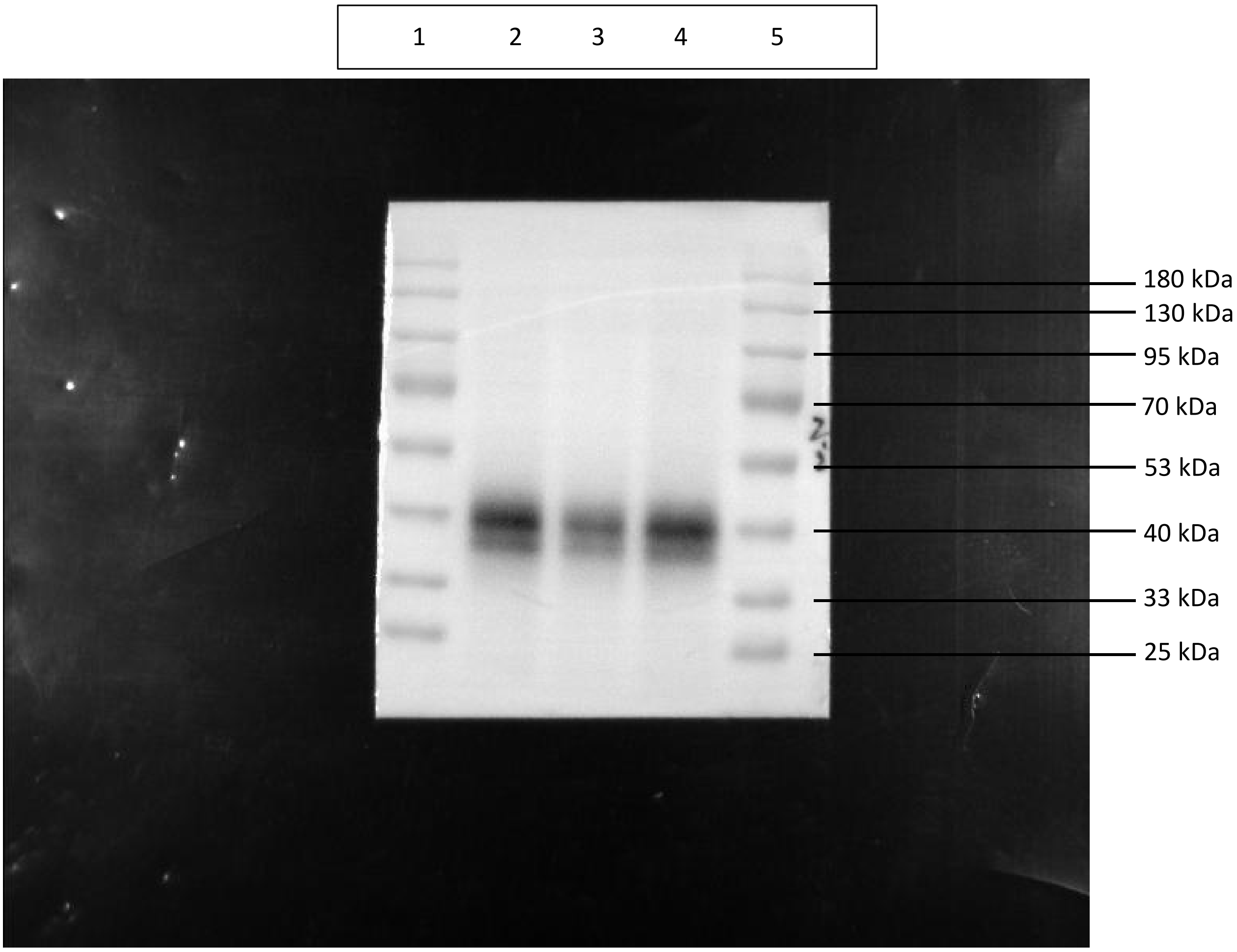

Lane1：Protein marker

Lane2：si-NC

Lane3：si-DNMT3A

Lane4：si-ADAMTS8

Lane5：Protein marker

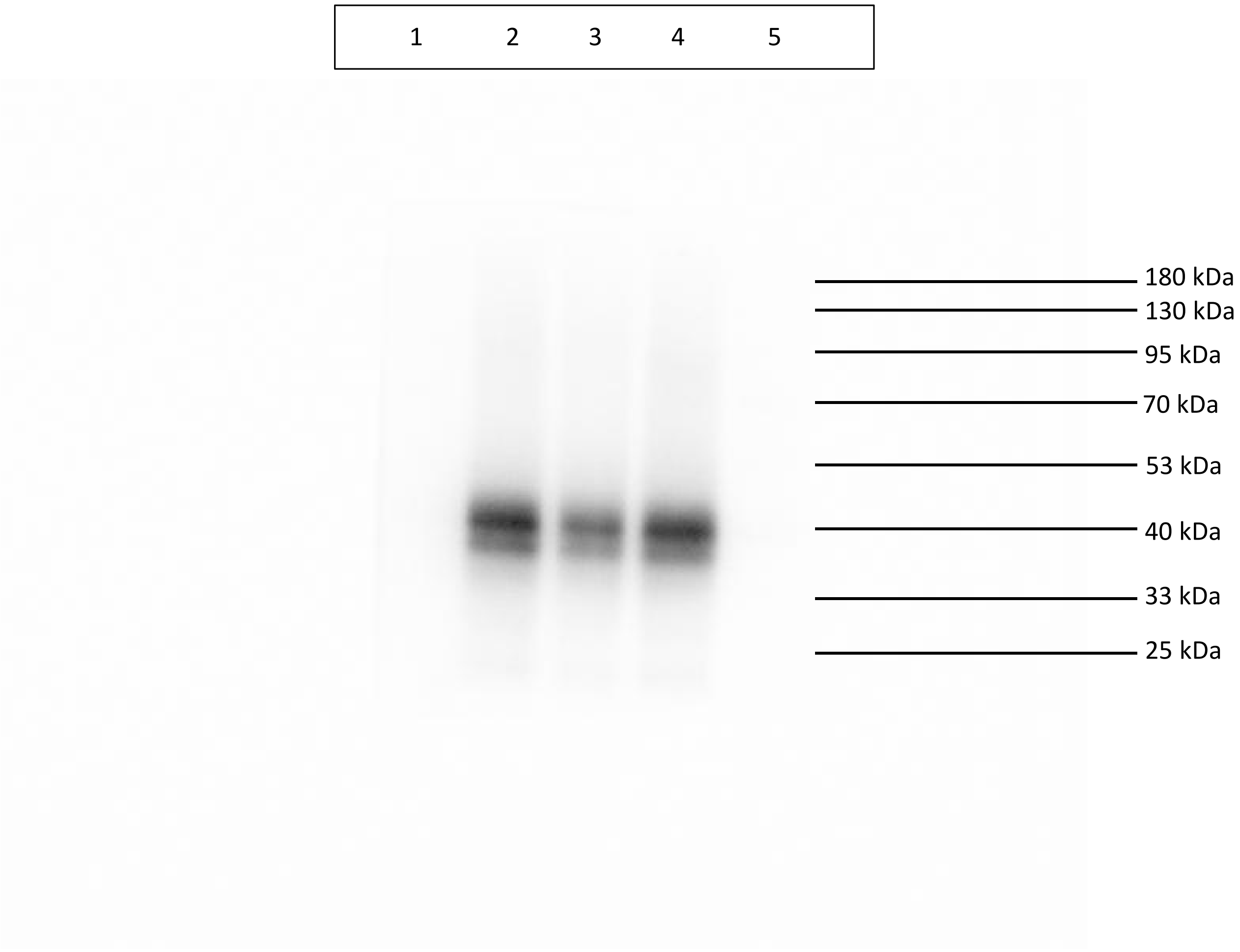

Lane1：Protein marker

Lane2：si-NC

Lane3：si-DNMT3A

Lane4：si-ADAMTS8

Lane5：Protein marker

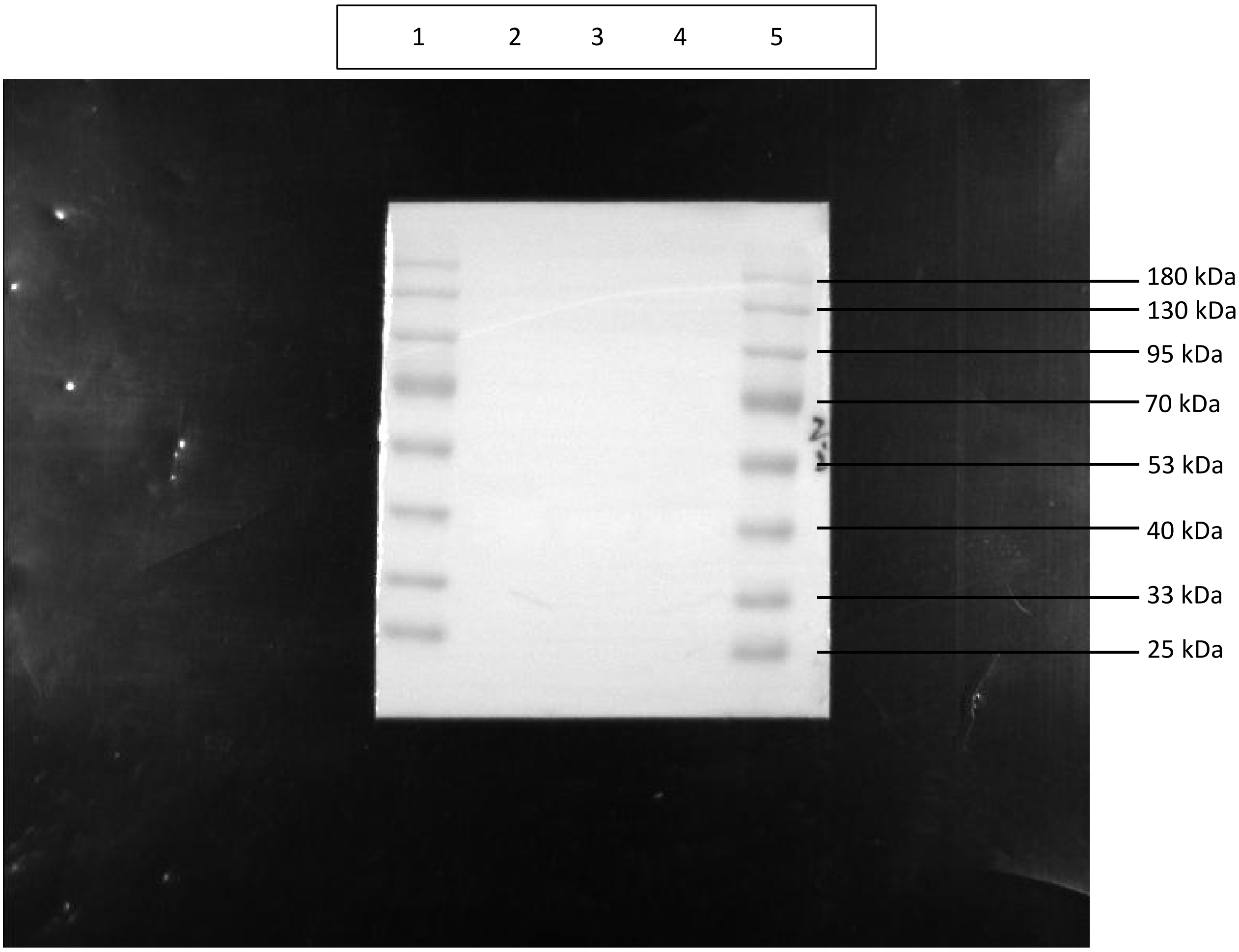

Lane1：Protein marker

Lane2：si-NC

Lane3：si-DNMT3A

Lane4：si-ADAMTS8

Lane5：Protein marker

Figure 6H

MEK1/2

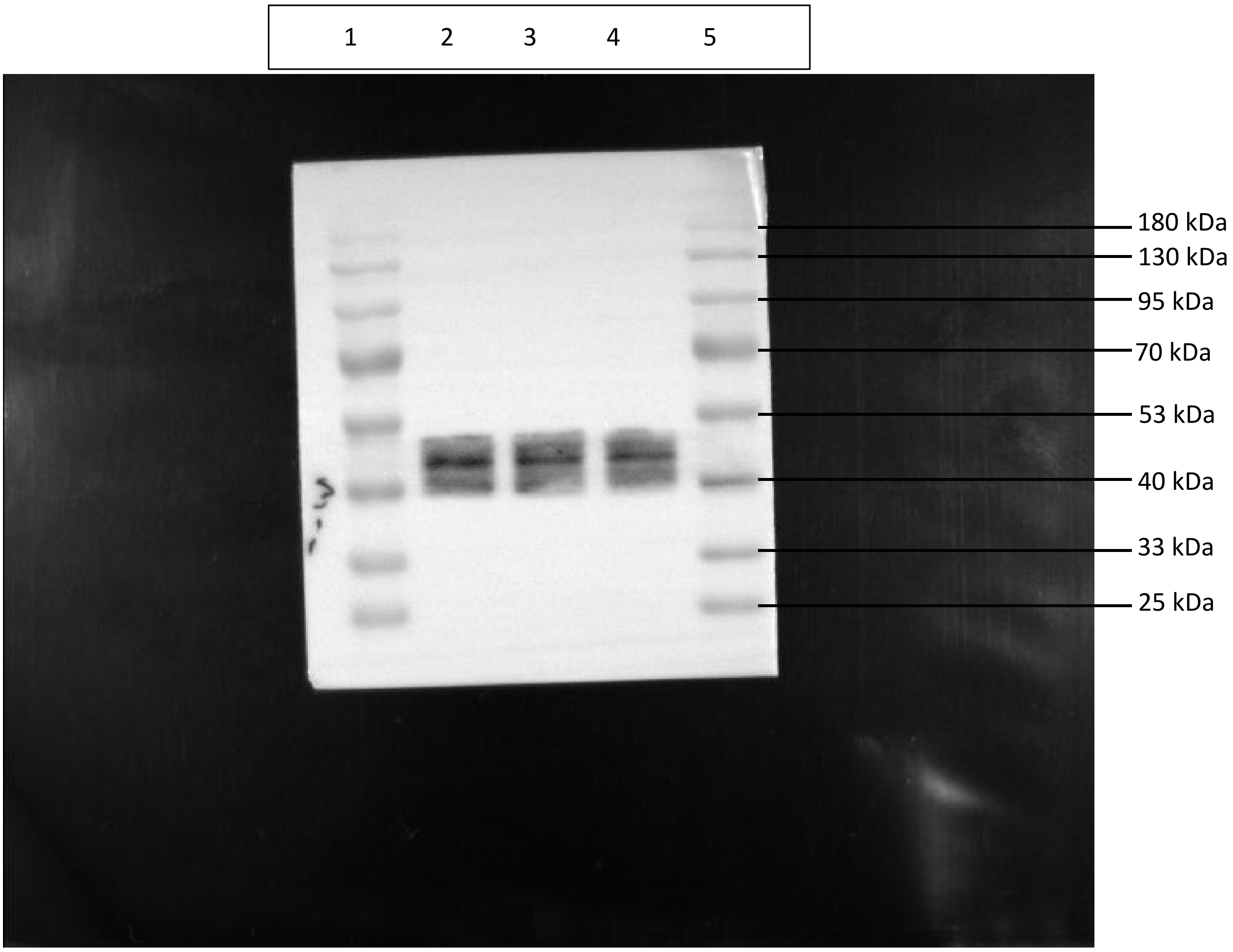

Lane1：Protein marker

Lane2：si-NC

Lane3：si-DNMT3A

Lane4：si-ADAMTS8

Lane5：Protein marker

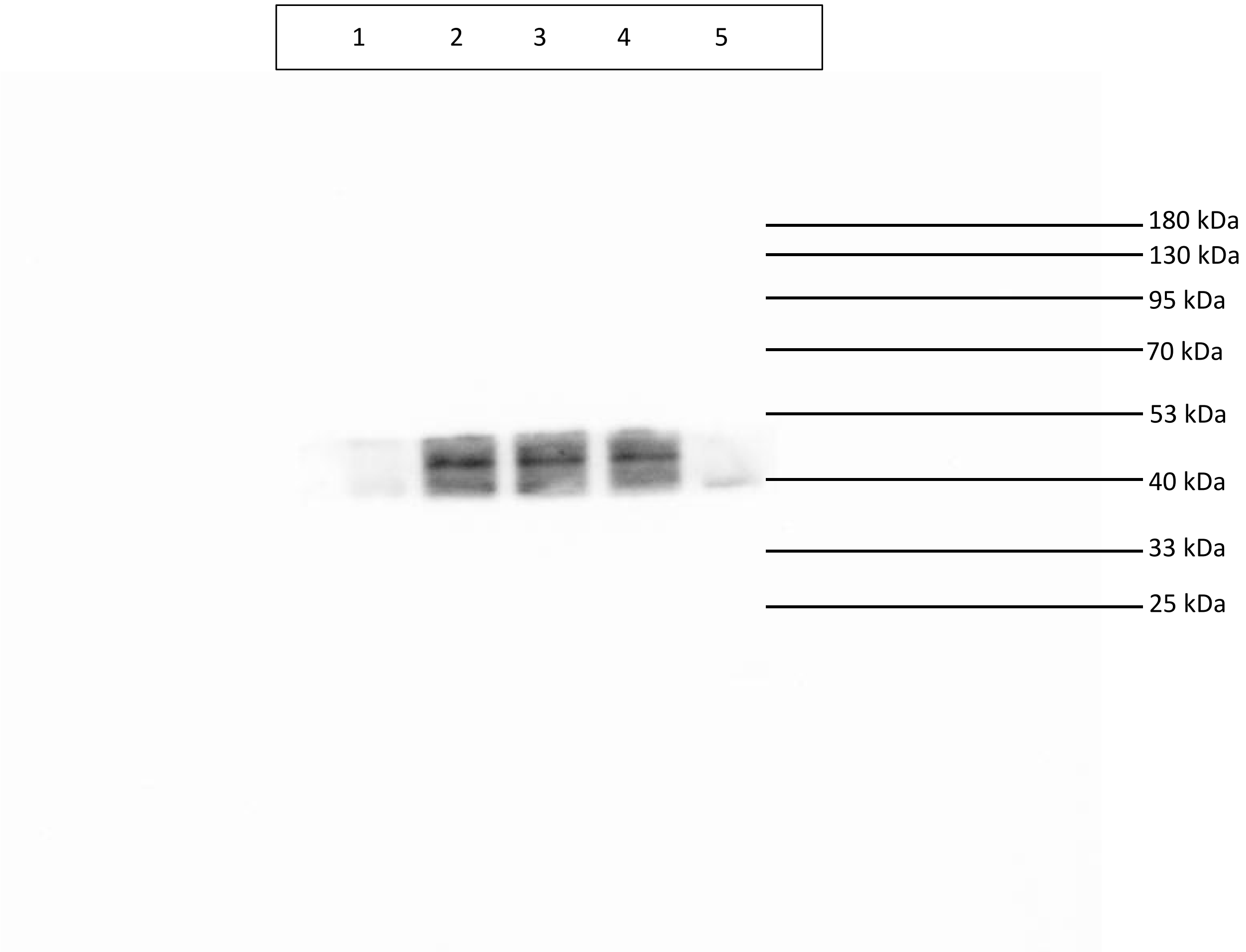

Lane1：Protein marker

Lane2：si-NC

Lane3：si-DNMT3A

Lane4：si-ADAMTS8

Lane5：Protein marker

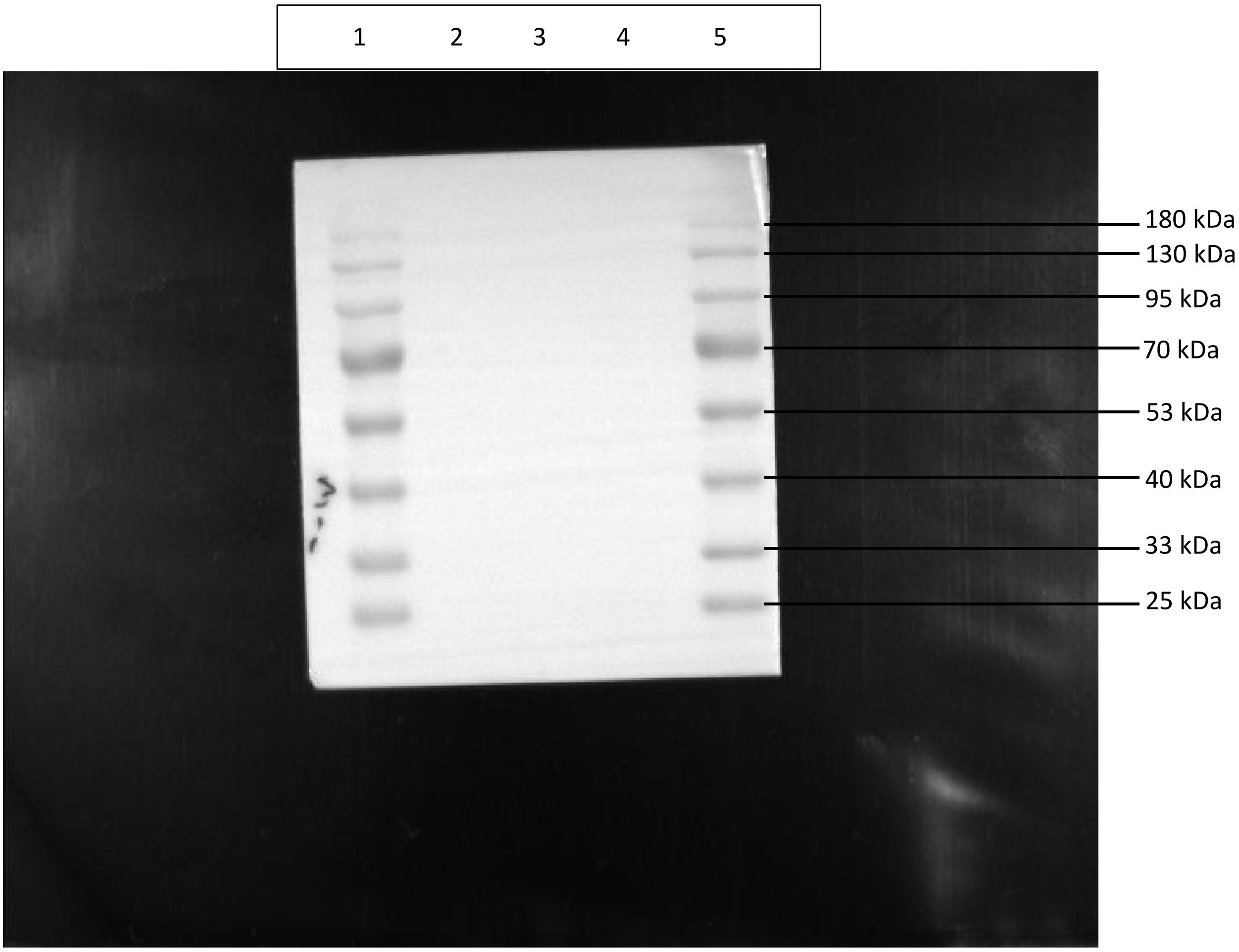

Lane1：Protein marker

Lane2：si-NC

Lane3：si-DNMT3A

Lane4：si-ADAMTS8

Lane5：Protein marker

Figure 6H

p-MEK1/2

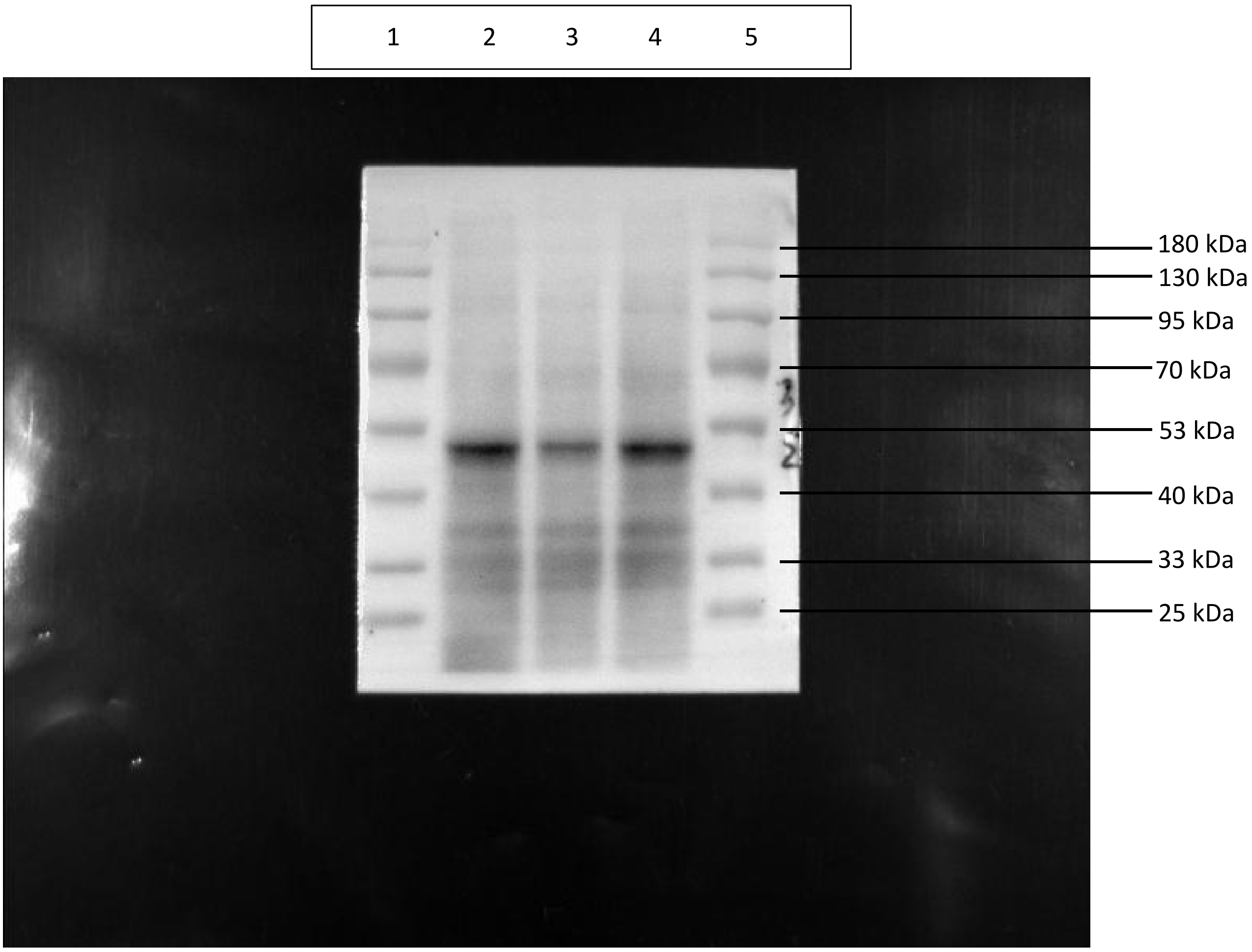

Lane1：Protein marker

Lane2：si-NC

Lane3：si-DNMT3A

Lane4：si-ADAMTS8

Lane5：Protein marker

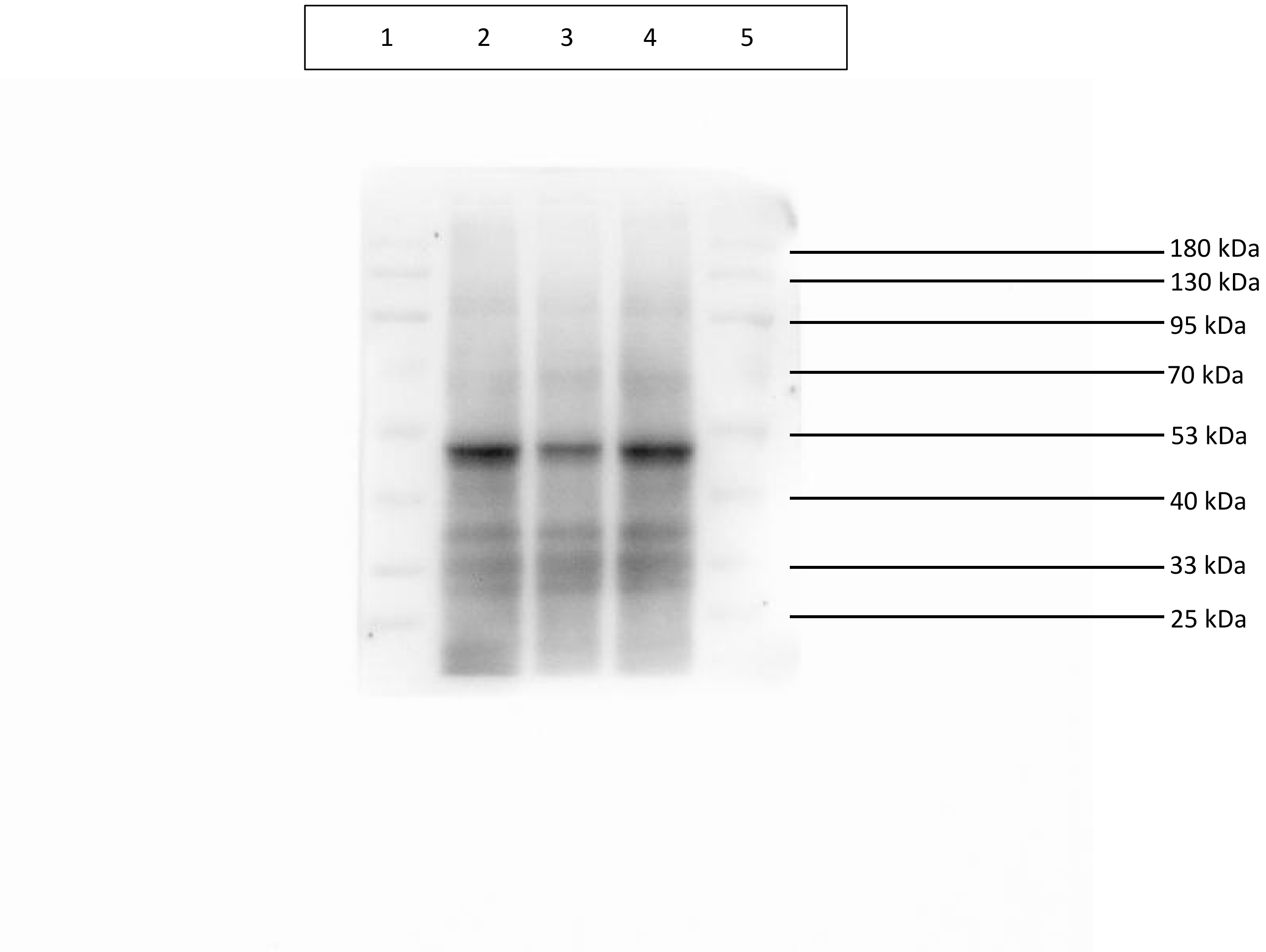

Lane1：Protein marker

Lane2：si-NC

Lane3：si-DNMT3A

Lane4：si-ADAMTS8

Lane5：Protein marker

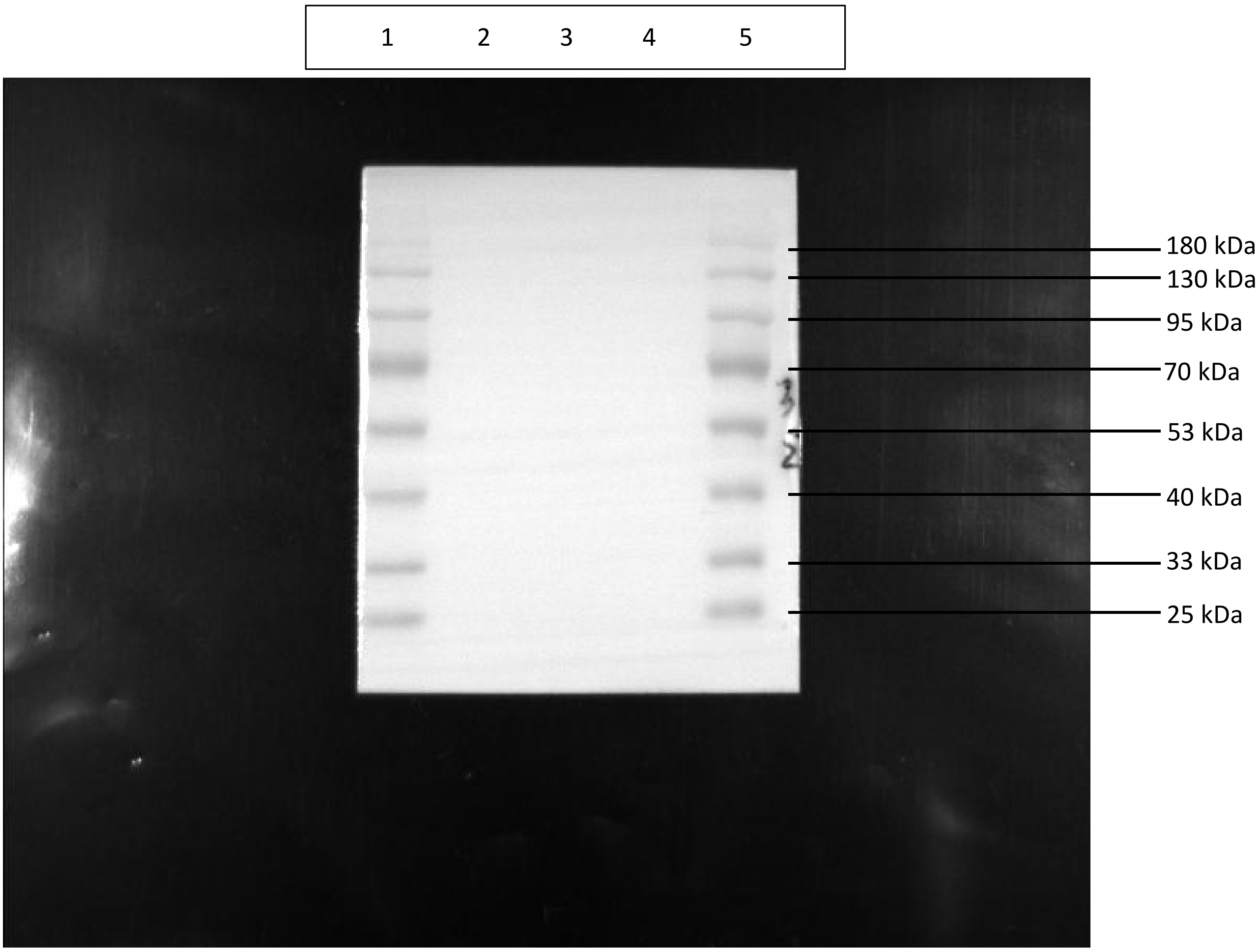

Lane1：Protein marker

Lane2：si-NC

Lane3：si-DNMT3A

Lane4：si-ADAMTS8

Lane5：Protein marker

Figure 6H

β-actin

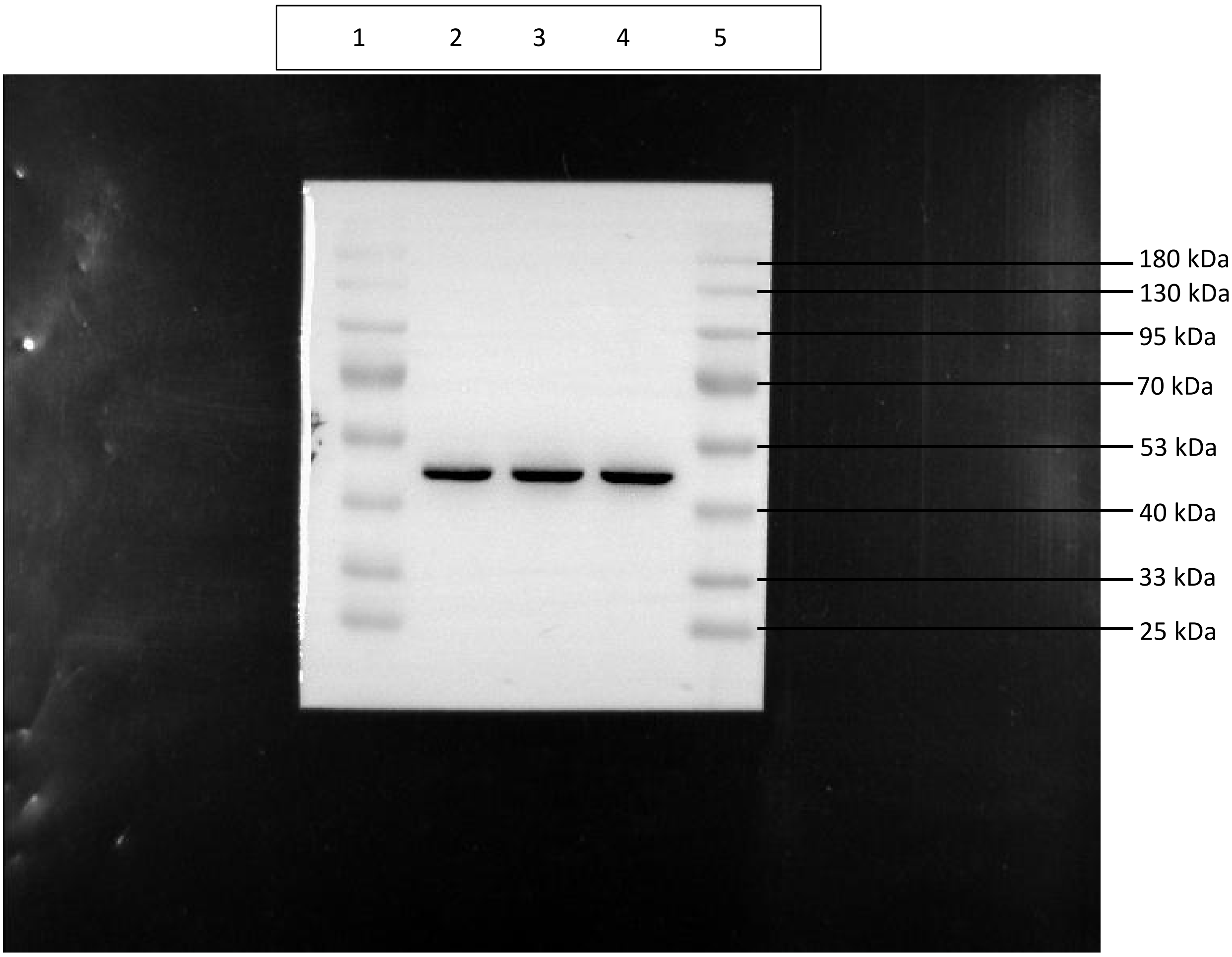

Lane1：Protein marker

Lane2：si-NC

Lane3：si-DNMT3A

Lane4：si-ADAMTS8

Lane5：Protein marker

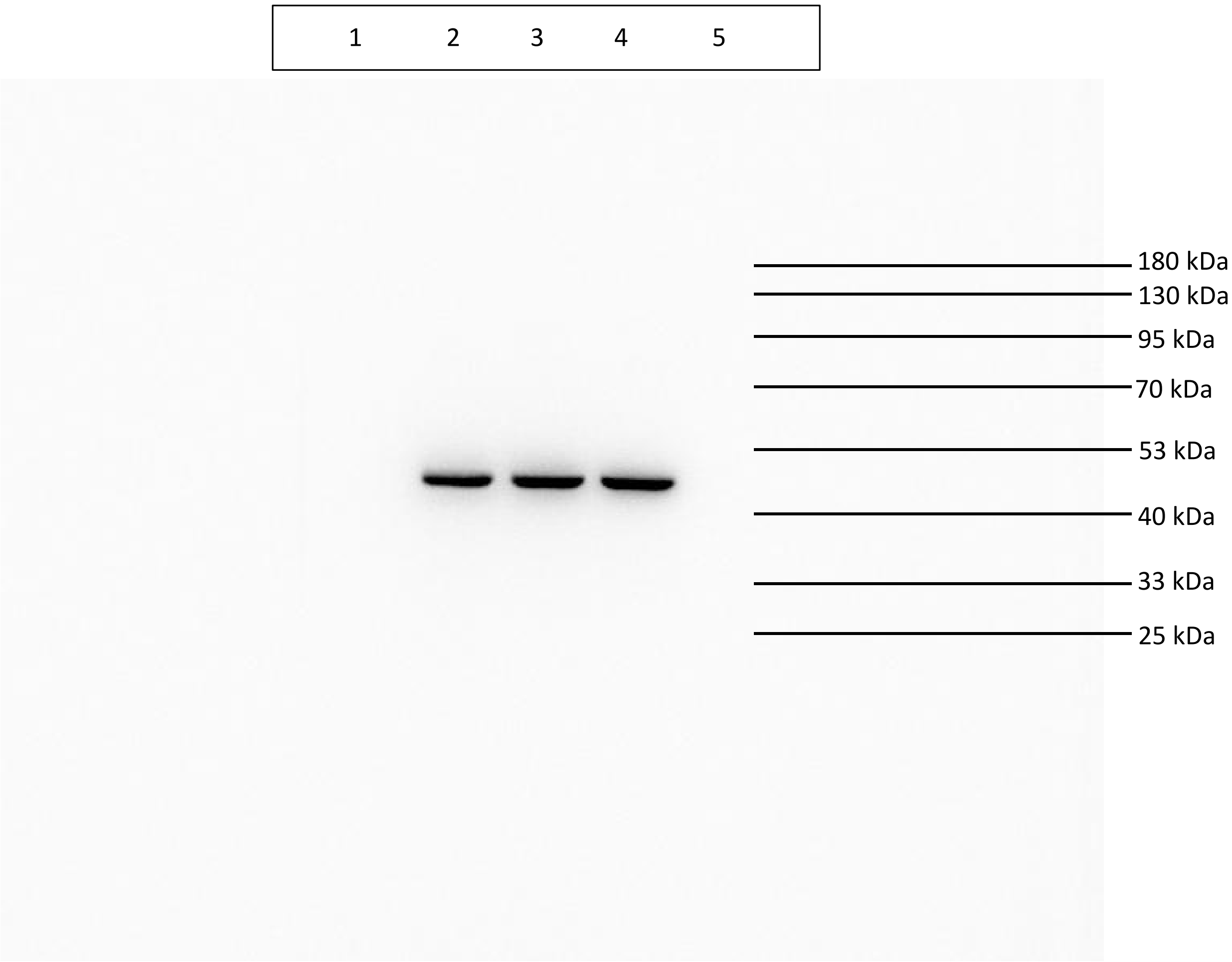

Lane1：Protein marker

Lane2：si-NC

Lane3：si-DNMT3A

Lane4：si-ADAMTS8

Lane5：Protein marker

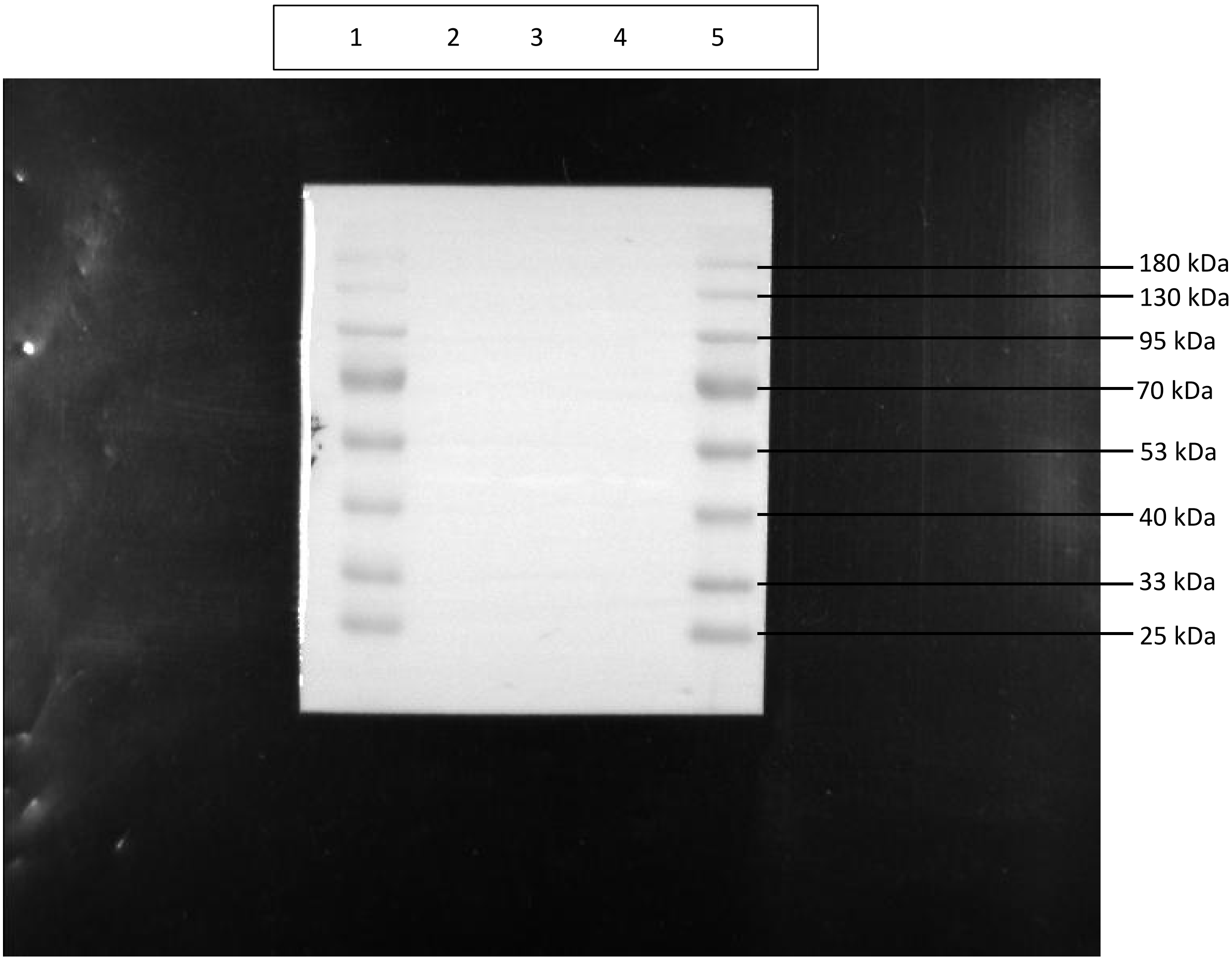

Lane1：Protein marker

Lane2：si-NC

Lane3：si-DNMT3A

Lane4：si-ADAMTS8

Lane5：Protein marker
